# Supplementary material for: Modeling pKa of the Brønsted Bases as an Approach to the Gibbs Energy of the Proton in Acetonitrile
Source: Int J Mol Sci. 2022 Sep 12;23(18):10576. doi: 10.3390/ijms231810576 (PMC9502073; doi:10.3390/ijms231810576)
Supplement: Supplementary file 1 [file ijms-23-10576-s001.zip › ijms-1912687-supplementary.pdf]

Supplementary material for

Modeling pK_a of the Brønsted Bases as an Approach to the Gibbs Energy of Proton in Acetonitrile

Zoran Glasovac*, Borislav Kovačević*

S1. Computational approach 2

S2. GB values of tricyclohexylphosphine (8) [1] 3

S3. Calculation of the pK_a 4

S4. Calculated gas phase energies for a P-bases 1-10 6

S5. N-Bases 10

S6. P-Bases 29

S7. C-Bases 40

S8. Cartesian coordinates 42

S1. Computational approach

a) General procedure

All calculations were performed using Gaussian09 program package with default optimization and convergence algorithms and parameters. All structures were identified as minima and their nature was verified by vibrational analysis (NImag = 0 in all cases). Gibbs corrections were used as obtained from calculations without correction for the low energy vibrational modes. Solvation energies were calculated as single point energy corrections to the gas phase Gibbs energies. Initial geometries for optimizations were taken either from literature (previous calculations or X-ray structure data) or by optimization of several conformers built upon the geometry of the most similar known structures. Employed computational models are as follows:

M1 = B3LYP/6-311+G(2df,p)//B3LYP/6-31G(d)
M2 = B3LYP(GD3)/6-311+G(2df,p)// B3LYP(GD3)/6-31G(d)
M3 = M06-2X/aug-cc-pVTZ//M06-2X/cc-pVDZ
M4 = M06-2X(GD3)/6-311++G(3df,2pd)//M06-2X/cc- pVDZ,
M5 = MP2(fc)/6-311+G(2df,p)/M06-2X/cc-pVDZ
M6 = MP2(fc)/6-311+G(2df,p)//B3LYP/6-31G(d)
M7 = B2PLYP/6-311+G(2df,p)// B3LYP/6-31G(d)
CPCM = CPCM(ACN)/B3LYP/6-31G(d)
IPCM = IPCM(ACN)/B3LYP/6-311+G(d,p)
SMD1 = SMD(ACN)/M06-2X/6-31G(d)
SMD2 = SMD(ACN)/M06-2X/6-31+G(d,p)

b) The gas–phase calculations

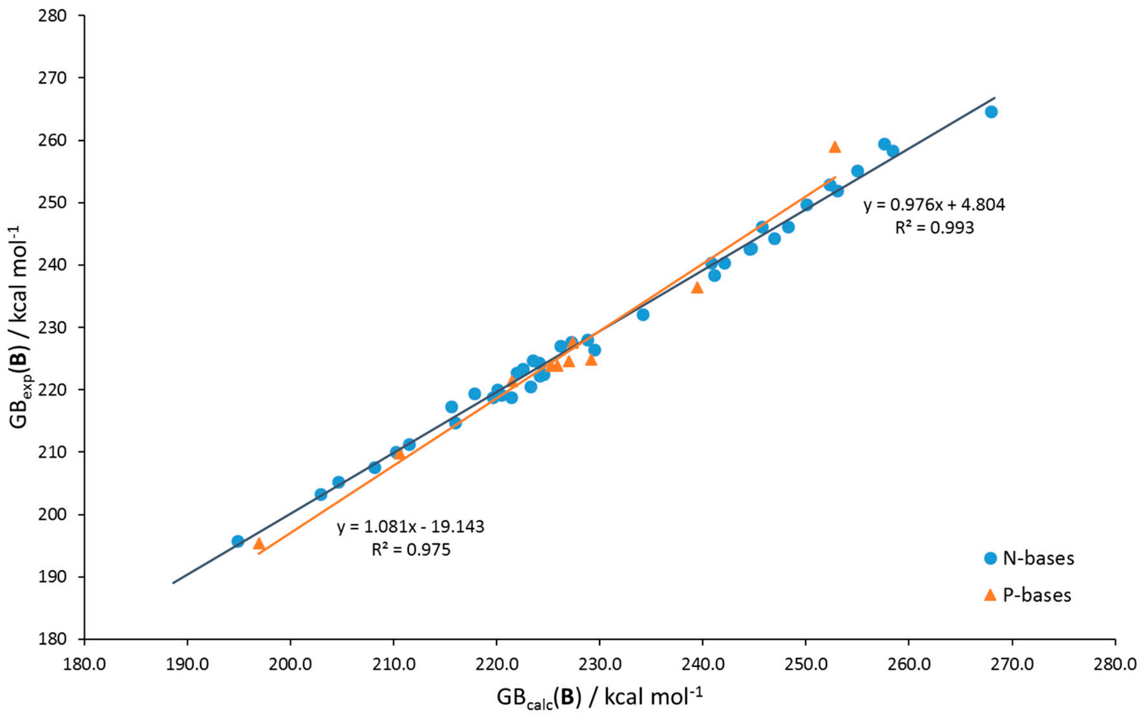

Figure S1. Quality of the correlation lines obtained for N-bases and P-bases using B3LYP/6-311+G(2df,p)//B3LYP/6-31G(d) (**M1**) level of theory.

S2. GB values of tricyclohexylphosphine (8) [1]

PA(**8**) was calculated according the equations (S1-S2).

$$GB = PA - T\Delta S_p \quad (S1)$$

$$\Delta S_p = S(\mathbf{B}) + S(H^+) - S(\mathbf{B}H^+) \quad (S2)$$

Taking into account entropy of the proton ($S(H^+)$ = 26.04 cal mol^{-1} K^{-1}, and assuming the negligible difference in entropies of **8** and **8**H$^+$ (Equations S1 and S2, $T\Delta S_p \approx 7.8$ kcal mol^{-1}), we obtain GB$_{lit}$(**8**) = 241 kcal mol^{-1}.

*Our suggested values for GB(**8**) and PA(**8**)*

Authors measured PA(**8**) = 249 ± 3 kcal mol^{-1} by bracketing method against 1,3-bis(dimethylamino)-propane and 1,4-bis(dimethylamino)butane. Both of these diamines can form intramolecular hydrogen bonds upon protonation. Therefore, protonation entropies for these two reference bases are significantly different than for **8**. In such cases, usage of GBs instead of PAs will most likely give more reliable results.
By using the available GBs for the 1,3-bis(dimethylamino)propane (235.5 kcal mol^{-1}) and 1,4-bis(dimethylamino)butane (237.3 kcal mol^{-1}), we can calculate GB(**8**) = 236 ± 3 kcal mol^{-1}.
After correction for the entropy of the proton, we obtain PA(**8**) = 244 ± 3 kcal mol^{-1}

S3. Calculation of the pK_a

Typical approaches to the calculation of pK_a are based on (a) calculation of the Gibbs energy of the proton transfer ($\Delta G_{PT,sol}$(**B** – **R**)) from the unknown base (**B**) to the reference base (**R**) (Eq (S3)) or (b) using Gibbs energy of dissociation ($\Delta G_{a,sol}$(**B**)) in the solution which can, in turn, be converted to a pK_a employing Equation (S4).

$$pK_a(\mathbf{B}H^+) - pK_a(\mathbf{R}H^+) = (\Delta G_{PT,sol}(\mathbf{B} - \mathbf{R}))/2.303RT \quad (S3)$$

$$pK_a(\mathbf{B}H^+) = \Delta G_{a,sol}(\mathbf{B}H^+)/2.303RT \quad (S4)$$

The shortcoming of the first approach is a necessity for a reference system with a highly reliable experimental pK_a and a highly accurate computational method for its treatment. The second approach demands an accurate experimental value for $G(H^+)$ in the solvent of interest. Both approaches are also sensitive to a choice of the solvation model since they usually suffer from systematic errors, as it is shown for CSM models in a work by Klamt et al.[2,3] Although the implicit solvation models have been improved significantly over time, the possibility of systematic errors even in the most modern CSM approaches like SMD[4] and CosmoTherm should not be disregarded.

When a sufficient number of the bases have reliable experimental pK_a, a linear correlation against the calculated $\Delta G_{p,sol}$ can be used to avoid both of the problematic points mentioned above. This approach was initially employed by Perakylla[5] and Kovačević and Maksić[6] using proton affinities in particular solvents as a measure of $\Delta G_{p,sol}$, and later on by Glasovac et al.[7] who used reduced basicity, i.e., the difference in the Gibbs energies between the base and its conjugate acid. In this way, linear correlation can be used regardless of whether the Gibbs energy of solvation for proton is known or not. Additionally, all bases are in a position of a reference base with equal weights diminishing the probability of error due to the selection of the inappropriate base.

By separating proton from base **B**, equation (S4) could be rewritten as Eqs. (5) and (6).

$$pK_a(\mathbf{B}H^+) = \Delta G'_{a,sol}(\mathbf{B}H^+)/2.303RT + G_{sol}(H^+)/2.303RT \quad (S5)$$

$$pK_a(\mathbf{B}H^+) = [G_{sol}(\mathbf{B}) - G_{sol}(\mathbf{B}H^+)]/2.303RT + [G^\circ_{sol}(H^+) + G^{\circ\rightarrow *}]/2.303RT \quad (S6)$$

Here, $\Delta G'_{a,sol}(\mathbf{B}H^+)$ stands for the reduced basicity of the base **B** in solution and $G_{sol}(H^+)$ stands for the Gibbs energy of proton in that solution. One could easily recognize an ideal value of slope = 1/2.303RT and intercept that hides the information of the Gibbs energy of the proton in the particular solvent and the whole term should be constant regardless of the structure of the base **B**. It should also be noted that the correction for the change in standard state ($G^{\circ\rightarrow *}$)[8] for neutral and protonated species cancels each other within $\Delta G'_{a,sol}$, while the one for proton remains included in the intercept.

To obtain Gibbs energies of each species (G_{sol}(**B**) and G_{sol}(**B**H^+)) and the reduced basicities ($\Delta G'_{a,sol}$) in solution, we employed a simple thermodynamic cycle shown in Figure 2

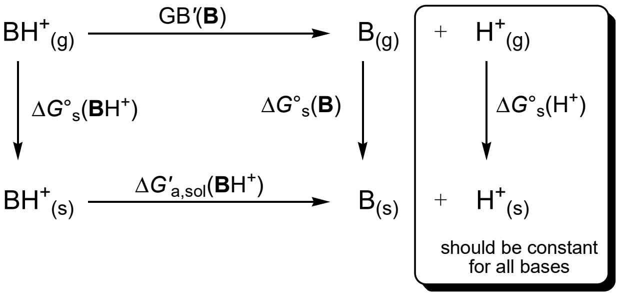

Figure S2. Simple thermodynamic cycle used for the calculations of the pK_a(**B**H^+)

Following the logic depicted in the thermodynamic cycle (Fig. S2) we can separate the gas-phase calculations from the solvation energies (Eq. S7). By combining Eqs (S6) and (S7) we obtain Eq. (S8)

$$G_{sol}(\mathbf{B}/\mathbf{B}\mathrm{H}^+) = G_g(\mathbf{B}/\mathbf{B}\mathrm{H}^+) + \Delta G_s(\mathbf{B}/\mathbf{B}\mathrm{H}^+) \quad \text{(S7)}$$

$$\mathrm{p}K_a(\mathbf{B}\mathrm{H}^+) = \{[G_g(\mathbf{B}) + \Delta G^\circ_s(\mathbf{B})] - [G_g(\mathbf{B}\mathrm{H}^+) + \Delta G^\circ_s(\mathbf{B}\mathrm{H}^+)]\}/2.303RT + [G^\circ_{sol}(\mathrm{H}^+) + G^{\circ\rightarrow *}]/2.303RT \quad \text{(S8)}$$

where G_g(**X**) stands for Gibbs energy of the species **X** in the gas phase, ΔG°_s(**X**) stands for solvation Gibbs energy of the species X at the standard state of 101325 Pa, $G^{\circ\rightarrow *}$ stands for the correction for the standard state change to 1 mol dm^{-3} and $G^\circ_{sol}(H^+)$ stands for the Gibbs energy of proton in solution assuming standard state of 101325 Pa.

Reorganization of the Eq. (S8) goes as follows:

$$\mathrm{p}K_a(\mathbf{B}\mathrm{H}^+) = \{[G_g(\mathbf{B}) - G_g(\mathbf{B}\mathrm{H}^+)] + [\Delta G^\circ_s(\mathbf{B}) - \Delta G^\circ_s(\mathbf{B}\mathrm{H}^+)]\}/2.303RT + [G^\circ_{sol}(\mathrm{H}^+) + G^{\circ\rightarrow *}]/2.303RT \quad \text{(S9)}$$

$$\mathrm{p}K_a(\mathbf{B}\mathrm{H}^+) = [\mathrm{GB}'(\mathbf{B}) + \Delta(\Delta G_s(\mathbf{B}/\mathbf{B}\mathrm{H}^+))]/2.303RT + [G^\circ_{sol}(\mathrm{H}^+) + G^{\circ\rightarrow *}]/2.303RT \quad \text{(S10)}$$

Equation (S10) is rewritten form of Eq. (S5) which indicate components that could be separately calculated at different levels of theory - GB' and $\Delta(\Delta G_s$(**B/B**H^+). The more general form of Eq. (S5) is given in Eq. (S11) and it includes empirical coefficients c_1 and c_2 which compensates for the systematic error in calculation of solvation energies.

$$\mathrm{p}K_a(\mathbf{B}\mathrm{H}^+) = c_1 \times [(\Delta G'_{a,sol}(\mathbf{B}\mathrm{H}^+)/2.303RT] + c_2 \times [G_{sol}(\mathrm{H}^+)/2.303RT] \quad \text{(S11)}$$

Equations (S12) - (S15) for the reduced gas-phase basicity (GB'(**B**)), reduced basicity of the base **B** in solution ($\Delta G'_{a,sol}$(**B**H^+)) and Gibbs energy of proton in a solution ($G_{sol}(H^+)$) were also used.

$$\mathrm{GB}'(\mathbf{B}) = G_g(\mathbf{B}) - G_g(\mathbf{B}\mathrm{H}^+) \quad \text{(S12)}$$

$$\Delta(\Delta G_s(\mathbf{B}/\mathbf{B}\mathrm{H}^+) = \Delta G^\circ_s(\mathbf{B}) - \Delta G^\circ_s(\mathbf{B}\mathrm{H}^+) \quad \text{(S13)}$$

$$\Delta G'_{a,sol}(\mathbf{B}\mathrm{H}^+) = \mathrm{GB}'(\mathbf{B}) + \Delta(\Delta G_s(\mathbf{B}/\mathbf{B}\mathrm{H}^+)) \quad \text{(S14)}$$

$$G_{sol}(\mathrm{H}^+) = G^\circ_{sol}(\mathrm{H}^+) + G^{\circ\rightarrow *} \quad \text{(S15)}$$

Equation (S11) shows linear relationship between pK_a(**B**H^+) and $\Delta G'_{a,sol}$(**B**H^+) which can be written in its general form as Eq. (3) given in the main text.

S4. Calculated gas phase energies for a P-bases 1-10

Table S1. Gas phase electronic energies (E_{el}), Gibbs corrections (G_{corr}), and Gibbs free energies (G) calculated using different theoretical approaches.

Base	E_{el} / a.u.	G_{corr} / a.u.	G / a.u.	GB_{calc} / kcal mol^{-1}	GB_{exp} / kcal mol^{-1}
B3LYP/6-311+G(2df,p)//B3LYP/6-31G(d) (M1)					
**1**	-382.50731	0.03034	-382.47697	196.9	195.4
**1**H^+	-382.84205	0.04127	-382.80078		
**2**	-421.84000	0.05727	-421.78273	210.6	209.8
**2**H^+	-422.19648	0.06814	-422.12834		
**3**	-461.17450	0.08521	-461.08929	221.5	221.4
**3**H^+	-461.54819	0.09589	-461.45230		
**4**	-579.14443	0.16397	-578.98046	227.4	227.5
**4**H^+	-579.52731	0.17448	-579.35283		
**5**	-652.96331	0.13185	-652.83146	225.3	223.9
**5**H^+	-653.34245	0.14192	-653.20053		
**6**	-844.75309	0.18009	-844.57300	227.0	224.6
**6**H^+	-845.13550	0.19079	-844.94471		
**7**	-1036.54287	0.22744	-1036.31543	229.2	224.8
**7**H^+	-1036.92860	0.23791	-1036.69069		
**8**	-1047.41477	0.43760	-1046.97717	239.4	236.4
**8**H^+	-1047.81676	0.44801	-1047.36875		
**9**	-577.93879	0.14903	-577.78976	225.8	223.9
**9**H^+	-578.31930	0.15962	-578.15968		
**10**	-916.14504	0.27265	-915.87239	252.8	259.0
**10**H^+	-916.57364	0.28833	-916.28531		

Table S1. (contd.)

Base	E_{el} / a.u.	G_{corr} / a.u.	G / a.u.	GB_{calc} / kcal mol^{-1}	GB_{exp} / kcal mol^{-1}
B3LYP(GD3)/6-311+G(2df,p)//B3LYP(GD3)/6-31+G (d) (M2)					
**1**	-382.50729	0.03018	-382.47711	196.9	195.4
**1**H$^+$	-382.84203	0.04113	-382.80090		
**2**	-421.83997	0.05696	-421.78301	210.5	209.8
**2**H$^+$	-422.19646	0.06794	-422.12852		
**3**	-461.18005	0.08473	-461.09532	221.8	221.4
**3**H$^+$	-461.55432	0.09551	-461.45881		
**4**	-579.14441	0.16326	-578.98115	227.2	227.5
**4**H$^+$	-579.52727	0.17399	-579.35328		
**5**	-652.97638	0.13137	-652.84501	225.7	223.9
**5**H$^+$	-653.35620	0.14154	-653.21466		
**6**	-844.77457	0.17967	-844.59490	227.8	224.6
**6**H$^+$	-845.15813	0.19024	-844.96789		
**7**	-1036.57384	0.22775	-1036.34609	230.4	224.8
**7**H$^+$	-1036.96057	0.23727	-1036.72330		
**8**	-1047.47463	0.43879	-1047.03584	240.3	236.4
**8**H$^+$	-1047.87792	0.44906	-1047.42886		
**9**	-577.93869	0.14827	-577.79042	225.6	223.9
**9**H$^+$	-578.31926	0.15933	-578.15993		
**10**	-916.18739	0.27264	-915.91475	251.1	259.0
**10**H$^+$	-916.61350	0.28852	-916.32498		
M06-2X/aug-cc-pVTZ//M06-2X/cc-pVDZ (M3)					
**1**	-382.43816	0.02993	-382.40823	194.5	195.4
**1**H$^+$	-382.76887	0.04068	-382.72819		
**2**	-421.75231	0.05651	-421.69580	207.5	209.8
**2**H$^+$	-422.10380	0.06726	-422.03654		
**3**	-461.06938	0.08412	-460.98526	217.4	221.4
**3**H$^+$	-461.43691	0.09514	-461.34177		
**4**	-578.98567	0.16245	-578.82322	223.3	227.5
**4**H$^+$	-579.36272	0.17366	-579.18906		
**5**	-652.79431	0.13174	-652.66257	220.3	223.9
**5**H$^+$	-653.16551	0.14188	-653.02363		
**6**	-844.52054	0.18079	-844.33975	221.9	224.6
**6**H$^+$	-844.89439	0.19103	-844.70336		
**7**	-1036.24683	0.22922	-1036.01761	223.5	224.8
**7**H$^+$	-1036.62320	0.23947	-1036.38373		
**8**	-1047.08788	0.43724	-1046.65064	235.3	236.4
**8**H$^+$	-1047.48264	0.44703	-1047.03561		
**9**	-577.79273	0.14789	-577.64484	221.8	223.9
**9**H$^+$	-578.16689	0.15866	-578.00823		
**10**	-915.89858	0.27256	-915.62602	249.2	259.0
**10**H$^+$	-916.32011	0.28698	-916.03313		

Table S1. (contd.)

Base	E_{el} / a.u.	G_{corr} / a.u.	G / a.u.	GB_{calc} / kcal mol^{-1}	GB_{exp} / kcal mol^{-1}
M06-2X(GD3)/6-311++G(3df,2pd)//M06-2X/cc-pVDZ (M4)					
**1**	-382.43362	0.02993	-382.40369	195.2	195.4
**1**H^+	-382.76539	0.04068	-382.72471		
**2**	-421.74678	0.05651	-421.69027	208.3	209.8
**2**H^+	-422.09954	0.06726	-422.03228		
**3**	-461.06299	0.08412	-460.97887	218.4	221.4
**3**H^+	-461.43200	0.09514	-461.33686		
**4**	-578.97582	0.16245	-578.81337	224.2	227.5
**4**H^+	-579.35433	0.17366	-579.18067		
**5**	-652.78081	0.13174	-652.64907	221.2	223.9
**5**H^+	-653.15346	0.14188	-653.01158		
**6**	-844.50025	0.18079	-844.31946	222.7	224.6
**6**H^+	-844.87545	0.19103	-844.68442		
**7**	-1036.22014	0.22922	-1035.99092	224.3	224.8
**7**H^+	-1036.59784	0.23947	-1036.35837		
**8**	-1047.06524	0.43724	-1046.62800	236.2	236.4
**8**H^+	-1047.46142	0.44703	-1047.01439		
**9**	-577.78289	0.14789	-577.63500	222.7	223.9
**9**H^+	-578.15854	0.15866	-577.99988		
**10**	-915.88349	0.27256	-915.61093	251.6	259.0
**10**H^+	-916.30882	0.28698	-916.02184		
MP2(fc)/6-311+G(2df,p)//M06-2X/cc-pVDZ (M5)					
**1**	-381.85792	0.02993	-381.82799	196.6	195.4
**1**H^+	-382.19194	0.04068	-382.15126		
**2**	-421.07969	0.05651	-421.02318	209.2	209.8
**2**H^+	-421.43381	0.06726	-421.36655		
**3**	-460.30508	0.08412	-460.22096	218.7	221.4
**3**H^+	-460.67466	0.09514	-460.57952		
**4**	-577.94279	0.16245	-577.78034	223.7	227.5
**4**H^+	-578.32050	0.17366	-578.14684		
**5**	-651.61876	0.13174	-651.48702	221.3	223.9
**5**H^+	-651.99155	0.14188	-651.84967		
**6**	-842.93611	0.18079	-842.75532	221.9	224.6
**6**H^+	-843.30996	0.19103	-843.11893		
**7**	-1034.25579	0.22922	-1034.02657	222.5	224.8
**7**H^+	-1034.63061	0.23947	-1034.39114		
**8**	-1044.97750	0.43724	-1044.54026	234.7	236.4
**8**H^+	-1045.37126	0.44703	-1044.92423		
**9**	-576.76307	0.14789	-576.61518	222.3	223.9
**9**H^+	-577.13810	0.15866	-576.97944		
**10**	-914.19662	0.27256	-913.92406	258.8	259.0
**10**H^+	-914.63341	0.28698	-914.34643		

Table S1. (contd.)

Base	E_{el} / a.u.	G_{corr} / a.u.	G / a.u.	GB_{calc} / kcal mol^{-1}	GB_{exp} / kcal mol^{-1}
MP2(fc)/6-311+G(2df,p)//B3LYP/6-31G(d) (M6)					
**1**	-381.85797	0.03034	-381.82763	196.5	195.4
**1**H$^+$	-382.19199	0.04127	-382.15072		
**2**	-421.07959	0.05727	-421.02232	209.1	209.8
**2**H$^+$	-421.43367	0.06814	-421.36553		
**3**	-460.30475	0.08522	-460.21953	218.9	221.4
**3**H$^+$	-460.67433	0.09590	-460.57843		
**4**	-577.94230	0.16397	-577.77833	223.9	227.5
**4**H$^+$	-578.31969	0.17448	-578.14521		
**5**	-651.61867	0.13227	-651.48640	221.1	223.9
**5**H$^+$	-651.99120	0.14249	-651.84871		
**6**	-842.93530	0.18009	-842.75521	221.8	224.6
**6**H$^+$	-843.30944	0.19079	-843.11865		
**7**	-1034.25425	0.22744	-1034.02681	222.8	224.8
**7**H$^+$	-1034.62975	0.23790	-1034.39185		
**8**	-1044.97492	0.43777	-1044.53715	234.3	236.4
**8**H$^+$	-1045.36861	0.44801	-1044.92060		
**9**	-576.76245	0.14903	-576.61342	222.4	223.9
**9**H$^+$	-577.13745	0.15962	-576.97783		
**10**	-914.19524	0.27265	-913.92259	258.1	259.0
**10**H$^+$	-914.63222	0.28833	-914.34389		
B2PLYP/6-311+G(2df,p)//B3LYP(d) (M7)					
**1**	-382.19725	0.02993	-382.16732	199.7	195.4
**1**H$^+$	-382.53627	0.04068	-382.49559		
**2**	-421.43267	0.05651	-421.37616	213.3	209.8
**2**H$^+$	-421.79341	0.06726	-421.72615		
**3**	-460.66987	0.08412	-460.58575	224.1	221.4
**3**H$^+$	-461.04801	0.09514	-460.95287		
**4**	-578.34890	0.16245	-578.18645	229.7	227.5
**4**H$^+$	-578.73620	0.17366	-578.56254		
**5**	-652.04490	0.13174	-651.91316	227.6	223.9
**5**H$^+$	-652.42782	0.14188	-652.28594		
**6**	-843.42000	0.18079	-843.23921	229.9	224.6
**6**H$^+$	-843.80654	0.19103	-843.61551		
**7**	-1034.79548	0.22922	-1034.56626	232.0	224.8
**7**H$^+$	-1035.18538	0.23947	-1034.94591		
**8**	-1045.51502	0.43724	-1045.07778	242.6	236.4
**8**H$^+$	-1045.92149	0.44703	-1045.47446		
**9**	-577.16266	0.14789	-577.01477	228.5	223.9
**9**H$^+$	-577.54761	0.15866	-577.38895		
**10**	-914.65765	0.27256	-914.38509	257.0	259.0
**10**H$^+$	-915.09165	0.28698	-914.80467		

S5. N-Bases

Phosphazenes

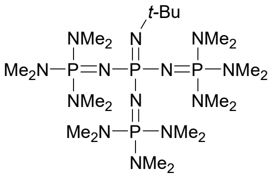

P4*tBu

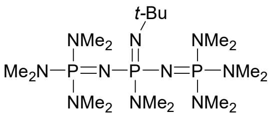

P3*tBu

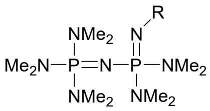

P2*R
(R = Me, Et, tBu)

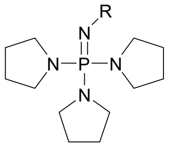

P1*R_pyrr
(R = H, Et)

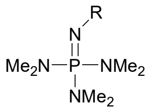

P1*R
(R = H, Me, Ph, tBu)

Guanidines:

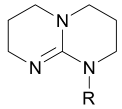

TBD (R=H)
MTBD (R = Me)

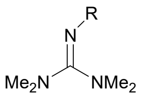

TMG*R
(R = H, Me, Ph)

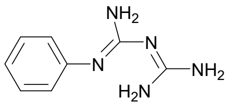

BGV

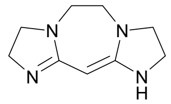

MTTT

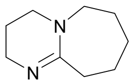

DBU

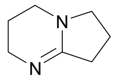

DBN

Amines:

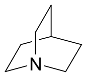

QQ

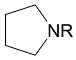

pyr*R
(R = H, Me)

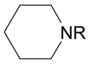

pp*R
(R = H, Me)

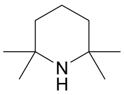

TMP

NH_3, NMe_3, NEt_3, $EtNH_2$, $BnNH_2$, iPr_2NH,

$PrNH_2$, PrNHMe, $PrNMe_2$, Ph_2NH

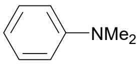

DMAn

**Pyridines**		**Anilines**	
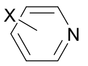		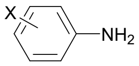	
**1P**	(X = H)	**1An**	(X = H)
**2P**	(X = 2-Me)	**2An**	(X = 2-Me)
**3P**	(X = 2,6-*di*-Me)	**3An**	(X = 2-Cl)
**4P**	(X = 2,4,6-*tri*-Me)	**4An**	(X = 2,6-*di*-Cl)
**5P**	(X = 4-OMe)	**5An**	(X = 4-Br)
**6P**	(X = 2-NH_2)	**6An**	(X = 4-OMe)
**7P**	(X = 3-NH_2)	**7An**	(X = 4-CF_3)
**8P**	(X = 4-NH_2)	**8An**	(X = 2-NO_2)
**9P**	(X = 4-NMe_2)	**9An**	(X = 3-NO_2)
**10P**	(X = 2,6-*di*-NH_2)	**10An**	(X = 4-NO_2)
**11P**	(X = 2,3-*di*-NH_2)		

Scheme S1. Structures of the N-Bases.

Table S2. Experimental and calculated $^{ACN}pK_a(\mathbf{B}H^+)$ values for a set of nitrogen bases.

Base **B**	$pK_a(\mathbf{B}H^+)$ exp.	M1 IPCM	M1 CPCM	M1 SMD1	M3 SMD2	M5 SMD2	M6 IPCM	M6 CPCM	M6 SMD1
**P4*tBu**	42.70	42.7	40.9	41.1	40.0	39.6	42.8	40.5	40.9
**P3*tBu**	38.60	38.3	37.1	37.2	38.7	39.0	38.4	36.9	37.1
**P2*tBu**	33.49	31.8	30.7	30.7	32.3	31.7	32.3	30.9	31.0
**P2*Et**	32.94	32.4	31.1	31.3	31.3	31.2	33.1	31.5	31.9
**P2*Me**	32.72	32.5	31.3	31.4	31.7	31.8	33.3	32.0	32.2
**P1*Ph**	21.25	21.7	20.9	20.9	21.6	21.1	22.1	21.3	21.3
**P1*tBu**	26.98	26.6	26.9	27.0	28.1	27.2	26.9	27.1	27.2
**P1*et_pyr**	28.88	27.7	30.5	25.7	28.8	28.5	28.6	31.7	26.5
**P1*H_pyr**	27.01	26.2	25.9	25.9	27.9	27.9	26.5	26.1	26.2
**P1*Me**	27.52	27.4	27.4	27.5	27.8	27.5	27.8	27.7	28.0
**P1*H**	25.85	26.0	26.0	26.2	26.2	26.2	26.1	26.1	26.3
**MTTT**	26.95	27.8	27.4	27.8	26.5	25.3	26.5	25.8	26.2
**BGV**	19.66	20.8	19.2	19.4	21.2	21.0	21.9	20.3	20.6
**TMG*Ph**	20.84	21.3	21.0	21.1	21.1	20.5	20.8	20.5	20.5
**MTBD**	25.49	25.8	26.1	26.3	25.5	24.8	25.5	25.6	25.9
**TBD**	26.03	25.9	26.2	26.5	25.7	25.0	25.5	25.7	26.0
**TMG*H**	23.30	23.8	24.0	24.3	23.5	23.3	23.6	23.7	24.0
**TMG*Me**	25.00	25.0	25.2	25.5	24.9	24.7	25.1	25.3	25.6
**DBU**	24.34	24.1	24.8	25.1	24.2	23.7	23.8	24.4	24.7
**DBN**	23.79	23.9	24.1	24.3	23.4	23.0	23.6	23.7	24.0
**pyr*Me**	18.42	17.6	19.3	19.2	19.2	19.4	18.3	20.2	20.2
**pyr*H**	19.56	18.4	19.1	19.2	18.8	19.5	19.0	19.9	20.1
**pp*H**	19.29	17.9	19.2	19.2	19.2	19.7	18.5	20.0	20.1
**pp*Me**	18.25	17.4	19.2	19.1	19.3	19.6	18.1	20.1	20.1
**TMP**	18.64	18.1	20.3	20.4	20.3	20.3	17.9	20.3	20.4
**QQ**	19.51	19.0	20.3	20.1	19.4	19.5	19.3	20.8	20.6
$\mathbf{NMe_3}$	17.62	17.2	18.6	18.6	18.8	19.5	18.5	20.1	20.2
$\mathbf{NEt_3}$	18.82	17.9	19.7	19.7	20.1	20.2	18.6	20.6	20.7
$\mathbf{EtNH_2}$	18.40	17.2	17.6	17.7	17.6	18.6	18.2	18.8	19.0
$\mathbf{NH_3}$	16.50	17.2	14.1	14.1	14.5	16.2	19.0	15.9	16.0
$\mathbf{iPr_2NH}$	18.81	17.5	19.6	19.6	19.9	20.2	17.9	20.2	20.3
$\mathbf{BnNH_2}$	16.91	15.1	16.0	16.3	16.0	16.9	15.6	16.8	17.0
$\mathbf{PrNH_2}$	18.43	16.6	17.5	17.6	17.6	18.6	17.6	18.7	18.9
**PrNHMe**	18.92	17.3	18.8	18.9	18.9	19.7	18.2	20.0	20.1
$\mathbf{PrNMe_2}$	18.30	17.2	19.0	19.0	19.3	19.8	18.2	20.3	20.3
**DMAn**	11.43	11.8	12.5	12.4	13.1	14.1	12.9	13.9	13.8
$\mathbf{Ph_2NH}$	5.97	6.1	5.8	5.4	6.1	6.4	6.2	6.1	5.6
**1P**	12.53	14.5	13.9	14.0	13.1	12.4	13.3	12.6	12.6
**2P**	13.32	15.4	15.1	15.1	14.4	13.5	13.9	13.5	13.5
**3P**	14.13	16.3	16.2	16.2	15.3	14.1	14.6	14.4	14.3
**4P**	14.98	16.9	16.9	17.1	16.1	14.6	15.0	14.9	14.9
**5P**	14.23	16.3	15.8	16.1	14.9	14.0	14.6	13.9	14.1
**6P**	14.47	16.5	15.6	15.7	14.9	13.4	14.6	13.4	13.4
**7P**	14.17	16.4	15.2	15.4	14.1	14.0	15.5	14.0	14.1

**8P**	17.62	19.3	18.1	18.4	17.3	16.0	17.6	16.2	16.4
**9P**	17.95	19.3	18.9	19.2	17.8	16.9	18.0	17.4	17.7
**10P**	14.77	17.8	16.7	16.9	16.1	14.1	15.4	13.9	14.0
**11P**	15.24	16.7	15.5	15.7	14.8	13.4	14.6	13.1	13.2
**1An**	10.62	10.1	9.7	9.6	10.0	10.5	10.4	10.1	10.0
**2An**	10.50	9.9	10.0	9.9	10.4	10.5	9.7	10.0	9.8
**3An**	7.86	7.4	6.5	6.3	6.9	7.2	7.4	6.6	6.4
**4An**	5.06	5.1	4.1	3.9	4.4	4.4	5.1	4.1	3.8
**5An**	9.43	8.7	8.8	8.9	9.1	9.6	8.9	9.1	9.2
**6An**	11.86	11.3	11.3	11.4	11.4	11.5	11.0	11.1	11.1
**7An**	8.03	7.4	6.8	6.9	7.6	8.3	7.9	7.4	7.5
**8An**	4.80	3.7	2.4	2.2	2.1	3.2	4.8	3.7	3.3
**9An**	7.68	7.0	6.8	6.8	7.2	7.6	7.1	7.1	7.1
**10An**	6.22	5.3	5.0	5.1	5.9	7.4	6.9	6.9	7.1
a		0.606	0.630	0.545	0.618	0.679	0.601	0.665	0.698
b		-154.9	-161.5	-133.5	-157.5	-173.7	-146.8	-169.6	-178.4
R^2	-	0.979	0.976	0.979	0.986	0.984	0.992	0.979	0.980
MUE		0.9	1.0	0.9	0.7	0.7	0.5	0.9	0.9
RMS	-	1.1	1.2	1.2	0.9	1.0	0.7	1.1	1.1

Table S3. Electronic energies (E_{el}), Gibbs corrections (G_{corr}) and total Gibbs energies (G_{tot}) calculated for a set of the nitrogen bases. All energies are given in atomic units (a.u.).

Base B	E_{el}(**B**)	G_{corr} (**B**)	G_{tot}(**B**)	E_{el}(**B**H$^+$)	G_{corr} (**B**H$^+$)	G_{tot}(**B**H$^+$)
	M1					
**P4*tBu**	-2954.17693	0.82141	-2953.35552	-2954.66395	0.83244	-2953.83150
**P3*tBu**	-2288.76464	0.65785	-2288.10678	-2289.23745	0.67031	-2288.56714
**P2*tBu**	-1623.34276	0.49408	-1622.84868	-1623.79297	0.50885	-1623.28411
**P2*Et**	-1544.69601	0.43914	-1544.25687	-1545.14691	0.45300	-1544.69391
**P2*Me**	-1505.36691	0.41344	-1504.95347	-1505.81758	0.42654	-1505.39104
**P1*et_pyr**	-1111.59233	0.38352	-1111.20880	-1112.02810	0.39879	-1111.62931
**P1*H_pyr**	-1032.95410	0.32980	-1032.62430	-1033.38613	0.34551	-1033.04062
**P1*Ph**	-1031.74541	0.30030	-1031.44511	-1032.16024	0.31349	-1031.84676
**P1*tBu**	-957.91503	0.33213	-957.58290	-958.34217	0.34722	-957.99495
**P1*Me**	-839.93516	0.25147	-839.68370	-840.36315	0.26622	-840.09693
**P1*H**	-800.62645	0.22576	-800.40069	-801.04826	0.23907	-800.80920
**MTTT**	-609.86562	0.22523	-609.64039	-610.30145	0.23922	-610.06223
**BGV**	-624.78756	0.18329	-624.60427	-625.19040	0.19589	-624.99451
**MTBD**	-478.28205	0.19930	-478.08275	-478.70091	0.21251	-478.48839
**TBD**	-438.96590	0.17343	-438.79247	-439.38247	0.18646	-439.19601
**TMG*Ph**	-593.83149	0.22777	-593.60372	-594.24024	0.24281	-593.99743
**TMG*Me**	-402.02603	0.18014	-401.84589	-402.44137	0.19575	-402.24562
**TMG*H**	-362.71605	0.15483	-362.56122	-363.12429	0.16879	-362.95551
**DBU**	-462.23730	0.21171	-462.02559	-462.65262	0.22715	-462.42547
**DBN**	-383.59143	0.15617	-383.43526	-384.00208	0.17101	-383.83108
**pyr*Me**	-251.97654	0.12809	-251.84845	-252.35512	0.14209	-252.21304
**pyr*H**	-212.65440	0.10196	-212.55244	-213.02934	0.11694	-212.91240
**pp*Me**	-291.30901	0.15733	-291.15168	-291.69045	0.17263	-291.51782
**pp*H**	-251.98853	0.13138	-251.85714	-252.36432	0.14649	-252.21784
**TMP**	-409.29046	0.23710	-409.05336	-409.67965	0.25168	-409.42797
**QQ**	-329.41043	0.16554	-329.24490	-329.79763	0.18067	-329.61697
**NMe_3**	-174.53473	0.09490	-174.43984	-174.90715	0.11016	-174.79699
**NEt_3**	-292.51282	0.17417	-292.33865	-292.89851	0.18939	-292.70911
**$EtNH_2$**	-135.22607	0.06780	-135.15827	-135.58590	0.08264	-135.50326
**NH_3**	-56.58411	0.01649	-56.56762	-56.92070	0.03255	-56.88815
**iPr_2NH**	-292.52540	0.17235	-292.35306	-292.90771	0.18748	-292.72023
**$BnNH_2$**	-327.01883	0.11481	-326.90402	-327.38374	0.12946	-327.25428
**$PrNH_2$**	-174.55190	0.09422	-174.45768	-174.91382	0.10911	-174.80472
**PrNHMe**	-213.86903	0.12016	-213.74887	-214.24166	0.13544	-214.10621
**$PrNMe_2$**	-253.18657	0.14652	-253.04005	-253.56540	0.16172	-253.40368
**Ph_2NH**	-518.81762	0.16154	-518.65608	-519.17255	0.17604	-518.99651
**DMAn**	-366.33330	0.14082	-366.19248	-366.70168	0.15567	-366.54601
**1P**	-248.36486	0.06164	-248.30322	-248.73311	0.07576	-248.65735
**2P**	-287.69768	0.08620	-287.61148	-288.07286	0.10009	-287.97277
**3P**	-327.03039	0.11013	-326.92026	-327.41193	0.12388	-327.28805
**4P**	-366.36112	0.13466	-366.22646	-366.74885	0.14915	-366.59969

**5P**	-362.93152	0.09111	-362.84041	-363.31276	0.10506	-363.20769
**6P**	-303.75450	0.07689	-303.67762	-304.13035	0.08992	-304.04043
**7P**	-303.74379	0.07653	-303.66726	-304.12284	0.08976	-304.03307
**8P**	-303.74870	0.07680	-303.67190	-304.13856	0.09101	-304.04755
**9P**	-382.38031	0.12895	-382.25136	-382.77797	0.14342	-382.63456
**10P**	-359.14391	0.09222	-359.05169	-359.52277	0.10194	-359.42083
**11P**	-359.13272	0.09288	-359.03984	-359.51257	0.10601	-359.40656
**1An**	-287.70290	0.08823	-287.61466	-288.04931	0.10131	-287.94800
**2An**	-327.03193	0.11448	-326.91745	-327.38130	0.12780	-327.25350
**3An**	-747.33145	0.07674	-747.25471	-747.67321	0.09018	-747.58303
**4An**	-1206.95877	0.06522	-1206.89355	-1207.29607	0.07867	-1207.21740
**5An**	-2861.24415	0.07497	-2861.16917	-2861.58441	0.08756	-2861.49685
**6An**	-402.26284	0.11717	-402.14567	-402.61782	0.13044	-402.48738
**7An**	-624.87244	0.08657	-624.78587	-625.20449	0.09925	-625.10524
**8An**	-492.28436	0.08675	-492.19760	-492.61752	0.10089	-492.51664
**9An**	-492.28038	0.08677	-492.19361	-492.60779	0.09940	-492.50839
**10An**	-492.28434	0.08695	-492.19739	-492.60679	0.09878	-492.50801

Table S3. (contd.)

Base B	E_{el}(**B**)	G_{corr} (**B**)	G_{tot}(**B**)	E_{el}(**B**H^+)	G_{corr} (**B**H^+)	G_{tot}(**B**H^+)
	M3					
**P4*tBu**	-2953.46520	0.81835	-2952.64685	-2953.94727	0.83352	-2953.11375
**P3*tBu**	-2288.19294	0.65855	-2287.53439	-2288.66037	0.66845	-2287.99192
**P2*tBu**	-1622.91265	0.49277	-1622.41988	-1623.36101	0.50691	-1622.85410
**P2*Et**	-1544.29175	0.43650	-1543.85525	-1544.73949	0.45210	-1544.28739
**P2*Me**	-1504.98085	0.41057	-1504.57027	-1505.42838	0.42518	-1505.00320
**P1*et_pyr**	-1111.25950	0.38352	-1110.87597	-1111.69441	0.39738	-1111.29702
**P1*H_pyr**	-1032.65655	0.33215	-1032.32440	-1033.08623	0.34467	-1032.74156
**P1*Ph**	-1031.44311	0.29974	-1031.14337	-1031.85737	0.31346	-1031.54391
**P1*tBu**	-957.62817	0.33216	-957.29601	-958.05493	0.34595	-957.70898
**P1*Me**	-839.69623	0.25034	-839.44589	-840.12230	0.26472	-839.85758
**P1*H**	-800.40515	0.22508	-800.18007	-800.82353	0.23740	-800.58612
**MTTT**	-609.63974	0.22562	-609.41412	-610.07029	0.23900	-609.83130
**BGV**	-624.55104	0.18471	-624.36633	-624.95433	0.19595	-624.75839
**MTBD**	-478.09642	0.19921	-477.89722	-478.51183	0.21234	-478.29949
**TBD**	-438.79683	0.17355	-438.62327	-439.20987	0.18642	-439.02345
**TMG*Ph**	-593.59794	0.22824	-593.36970	-594.00339	0.24290	-593.76049
**TMG*Me**	-401.85637	0.17972	-401.67665	-402.26849	0.19563	-402.07286
**TMG*H**	-362.56490	0.15463	-362.41027	-362.96901	0.16811	-362.80090
**DBU**	-462.05221	0.21166	-461.84054	-462.46373	0.22655	-462.23718
**DBN**	-383.43813	0.15601	-383.28212	-383.84523	0.17072	-383.67451
**pyr*Me**	-251.86242	0.12773	-251.73469	-252.23949	0.14219	-252.09730
**pyr*H**	-212.55811	0.10151	-212.45661	-212.93034	0.11661	-212.81373
**pp*Me**	-291.17896	0.15680	-291.02216	-291.55892	0.17214	-291.38678
**pp*H**	-251.87560	0.13110	-251.74450	-252.24910	0.14598	-252.10312
**TMP**	-409.11417	0.23582	-408.87835	-409.50057	0.25049	-409.25008
**QQ**	-329.27542	0.16425	-329.11118	-329.66031	0.18161	-329.47870
**NMe_3**	-174.44649	0.09326	-174.35323	-174.81766	0.10859	-174.70907
**NEt_3**	-292.37011	0.17231	-292.19780	-292.75472	0.18772	-292.56700
**$EtNH_2$**	-135.15515	0.06762	-135.08753	-135.51258	0.08204	-135.43054
**NH_3**	-56.55252	0.01534	-56.53717	-56.88663	0.02949	-56.85714
**iPr_2NH**	-292.38439	0.17271	-292.21168	-292.76449	0.18762	-292.57687
**$BnNH_2$**	-326.88318	0.11457	-326.76861	-327.24478	0.12937	-327.11541
**$PrNH_2$**	-174.46259	0.09391	-174.36868	-174.82204	0.10832	-174.71372
**PrNHMe**	-213.76092	0.11966	-213.64125	-214.13142	0.13477	-213.99665
**$PrNMe_2$**	-253.06204	0.14572	-252.91632	-253.43952	0.16102	-253.27851
**Ph_2NH**	-518.61669	0.16153	-518.45516	-518.96916	0.17625	-518.79291
**DMAn**	-366.18076	0.14120	-366.03957	-366.54737	0.15570	-366.39167
**1P**	-248.26466	0.06197	-248.20269	-248.62838	0.07598	-248.55240
**2P**	-287.57875	0.08647	-287.49228	-287.94946	0.09995	-287.84951
**3P**	-326.89277	0.11057	-326.78220	-327.26993	0.12451	-327.14543
**4P**	-366.20502	0.13448	-366.07054	-366.58830	0.14875	-366.43955
**5P**	-362.79403	0.09162	-362.70241	-363.16991	0.10546	-363.06444

**6P**	-303.63624	0.07726	-303.55899	-304.00746	0.09034	-303.91712
**7P**	-303.62548	0.07681	-303.54867	-303.99870	0.09004	-303.90866
**8P**	-303.63063	0.07717	-303.55347	-304.01564	0.09148	-303.92416
**9P**	-382.22740	0.12899	-382.09841	-382.62025	0.14402	-382.47623
**10P**	-359.00792	0.09258	-358.91534	-359.38179	0.10249	-359.27930
**11P**	-358.99773	0.09349	-358.90424	-359.37215	0.10635	-359.26580
**1An**	-287.58523	0.08854	-287.49669	-287.92881	0.10129	-287.82752
**2An**	-326.89712	0.11465	-326.78248	-327.24360	0.12741	-327.11619
**3An**	-747.19850	0.07725	-747.12125	-747.53730	0.08978	-747.44752
**4An**	-1206.81055	0.06585	-1206.74470	-1207.14530	0.07862	-1207.06668
**5An**	-2861.22978	0.07520	-2861.15458	-2861.56702	0.08783	-2861.47919
**6An**	-402.10788	0.11792	-401.98996	-402.45942	0.13123	-402.32819
**7An**	-624.67684	0.08724	-624.58960	-625.00631	0.09974	-624.90656
**8An**	-492.10326	0.08741	-492.01585	-492.43383	0.10087	-492.33296
**9An**	-492.10002	0.08764	-492.01238	-492.42475	0.10045	-492.32429
**10An**	-492.10334	0.08786	-492.01548	-492.42393	0.10000	-492.32393

Table S3. (contd.)

Base B	E_{el}(**B**)	G_{corr} (**B**)	G_{tot}(**B**)	E_{el}(**B**H^+)	G_{corr} (**B**H^+)	G_{tot}(**B**H^+)
	M5					
**P4*tBu**	-2948.26575	0.81835	-2947.44741	-2948.74075	0.83352	-2947.90723
**P3*tBu**	-2284.12272	0.65855	-2283.46417	-2284.58490	0.66845	-2283.91646
**P2*tBu**	-1619.97582	0.49277	-1619.48305	-1620.41805	0.50691	-1619.91114
**P2*Et**	-1541.53683	0.43650	-1541.10033	-1541.97965	0.45210	-1541.52755
**P2*Me**	-1502.31712	0.41057	-1501.90655	-1502.76029	0.42518	-1502.33511
**P1*et_pyr**	-1109.12351	0.38352	-1108.73999	-1109.55351	0.39738	-1109.15613
**P1*H_pyr**	-1030.70518	0.33215	-1030.37304	-1031.13096	0.34467	-1030.78629
**P1*Ph**	-1029.50127	0.29974	-1029.20153	-1029.91161	0.31346	-1029.59816
**P1*tBu**	-955.82366	0.33216	-955.49150	-956.24420	0.34595	-955.89825
**P1*Me**	-838.16549	0.25034	-837.91515	-838.58676	0.26472	-838.32204
**P1*H**	-798.96741	0.22508	-798.74233	-799.38201	0.23740	-799.14461
**MTTT**	-608.35456	0.22562	-608.12894	-608.77872	0.23900	-608.53972
**BGV**	-623.27067	0.18471	-623.08596	-623.67066	0.19595	-623.47471
**MTBD**	-477.07327	0.19921	-476.87407	-477.48337	0.21234	-477.27103
**TBD**	-437.86596	0.17355	-437.69241	-438.27375	0.18642	-438.08733
**TMG*Ph**	-592.32365	0.22824	-592.09541	-592.72483	0.24290	-592.48193
**TMG*Me**	-400.99224	0.17972	-400.81252	-401.40039	0.19563	-401.20476
**TMG*H**	-361.79382	0.15463	-361.63919	-362.19438	0.16811	-362.02627
**DBU**	-461.03795	0.21166	-460.82629	-461.44504	0.22655	-461.21849
**DBN**	-382.60998	0.15601	-382.45397	-383.01285	0.17072	-382.84213
**pyr*Me**	-251.29621	0.12773	-251.16848	-251.67137	0.14219	-251.52918
**pyr*H**	-212.08418	0.10151	-211.98267	-212.45568	0.11661	-212.33907
**pp*Me**	-290.51980	0.15680	-290.36300	-290.89791	0.17214	-290.72577
**pp*H**	-251.30877	0.13110	-251.17767	-251.68111	0.14598	-251.53513
**TMP**	-408.18282	0.23582	-407.94701	-408.56656	0.25049	-408.31607
**QQ**	-328.53844	0.16425	-328.37420	-328.92115	0.18161	-328.73955
**NMe_3**	-174.05274	0.09326	-173.95948	-174.42337	0.10859	-174.31478
**NEt_3**	-291.69859	0.17231	-291.52628	-292.08088	0.18772	-291.89316
**$EtNH_2$**	-134.85554	0.06762	-134.78792	-135.21319	0.08204	-135.13115
**NH_3**	-56.44128	0.01534	-56.42594	-56.77786	0.02949	-56.74837
**iPr_2NH**	-291.71371	0.17271	-291.54101	-292.09210	0.18762	-291.90448
**$BnNH_2$**	-326.17003	0.11457	-326.05546	-326.53206	0.12937	-326.40269
**$PrNH_2$**	-174.06943	0.09391	-173.97552	-174.42920	0.10832	-174.32088
**PrNHMe**	-213.27386	0.11966	-213.15420	-213.64394	0.13477	-213.50917
**$PrNMe_2$**	-252.48241	0.14572	-252.33669	-252.85870	0.16102	-252.69768
**Ph_2NH**	-517.48830	0.16153	-517.32677	-517.84156	0.17625	-517.66532
**DMAn**	-365.37214	0.14120	-365.23094	-365.73987	0.15570	-365.58417
**1P**	-247.73638	0.06197	-247.67441	-248.09716	0.07598	-248.02119
**2P**	-286.95852	0.08647	-286.87205	-287.32535	0.09995	-287.22540
**3P**	-326.18063	0.11057	-326.07006	-326.55311	0.12451	-326.42861
**4P**	-365.40089	0.13448	-365.26641	-365.77870	0.14875	-365.62995
**5P**	-362.05867	0.09162	-361.96705	-362.43063	0.10546	-362.32517

**6P**	-303.00616	0.07726	-302.92890	-303.37198	0.09034	-303.28164
**7P**	-302.99694	0.07681	-302.92013	-303.36853	0.09004	-303.27849
**8P**	-303.00018	0.07717	-302.92301	-303.37997	0.09148	-303.28849
**9P**	-381.41003	0.12899	-381.28104	-381.79869	0.14402	-381.65467
**10P**	-358.27549	0.09258	-358.18291	-358.64267	0.10249	-358.54018
**11P**	-358.26881	0.09349	-358.17532	-358.63806	0.10635	-358.53171
**1An**	-286.96313	0.08854	-286.87459	-287.30711	0.10129	-287.20582
**2An**	-326.18379	0.11465	-326.06915	-326.52978	0.12741	-326.40237
**3An**	-746.07556	0.07725	-745.99831	-746.41483	0.08978	-746.32506
**4An**	-1205.18713	0.06585	-1205.12128	-1205.52217	0.07862	-1205.44355
**5An**	-2858.90348	0.07520	-2858.82828	-2859.24134	0.08783	-2859.15351
**6An**	-401.28021	0.11792	-401.16229	-401.63099	0.13123	-401.49976
**7An**	-623.59062	0.08724	-623.50338	-623.92151	0.09974	-623.82177
**8An**	-491.17898	0.08741	-491.09157	-491.51284	0.10087	-491.41197
**9An**	-491.17924	0.08764	-491.09160	-491.50468	0.10045	-491.40423
**10An**	-491.17971	0.08786	-491.09185	-491.50401	0.10000	-491.40401

Table S3. (contd.)

Base B	E_{el}(**B**)	G_{corr} (**B**)	G_{tot}(**B**)	E_{el}(**B**H$^+$)	G_{corr} (**B**H$^+$)	G_{tot}(**B**H$^+$)
	M6					
**P4*tBu**	-2948.26100	0.82141	-2947.43959	-2948.73631	0.83244	-2947.90386
**P3*tBu**	-2284.11981	0.65785	-2283.46196	-2284.58225	0.67031	-2283.91194
**P2*tBu**	-1619.97402	0.49408	-1619.47994	-1620.41628	0.50885	-1619.90743
**P2*Et**	-1541.53607	0.43914	-1541.09693	-1541.97942	0.45300	-1541.52642
**P2*Me**	-1502.31680	0.41344	-1501.90337	-1502.76049	0.42654	-1502.33395
**P1*et_pyr**	-1109.12169	0.38352	-1108.73816	-1109.55196	0.39879	-1109.15317
**P1*H_pyr**	-1030.70432	0.32980	-1030.37453	-1031.12967	0.34551	-1030.78416
**P1*Ph**	-1029.50071	0.30030	-1029.20041	-1029.91024	0.31349	-1029.59675
**P1*tBu**	-955.82334	0.33213	-955.49121	-956.24350	0.34722	-955.89628
**P1*Me**	-838.16527	0.25147	-837.91380	-838.58647	0.26622	-838.32026
**P1*H**	-798.96749	0.22576	-798.74173	-799.38210	0.23907	-799.14304
**MTTT**	-608.35367	0.22523	-608.12844	-608.77790	0.23922	-608.53868
**BGV**	-623.27022	0.18329	-623.08693	-623.66996	0.19589	-623.47407
**MTBD**	-477.07239	0.19930	-476.87309	-477.48273	0.21251	-477.27022
**TBD**	-437.86532	0.17343	-437.69189	-438.27313	0.18646	-438.08667
**TMG*Ph**	-592.32251	0.22777	-592.09474	-592.72374	0.24281	-592.48093
**TMG*Me**	-400.99170	0.18014	-400.81156	-401.39994	0.19575	-401.20419
**TMG*H**	-361.79336	0.15483	-361.63853	-362.19400	0.16879	-362.02521
**DBU**	-461.03680	0.21171	-460.82509	-461.44413	0.22715	-461.21698
**DBN**	-382.60945	0.15617	-382.45328	-383.01229	0.17101	-382.84129
**pyr*Me**	-251.29585	0.12809	-251.16777	-251.67095	0.14209	-251.52886
**pyr*H**	-212.08412	0.10196	-211.98216	-212.45527	0.11694	-212.33832
**pp*Me**	-290.51935	0.15733	-290.36202	-290.89732	0.17263	-290.72468
**pp*H**	-251.30858	0.13138	-251.17719	-251.68061	0.14649	-251.53413
**TMP**	-408.18211	0.23710	-407.94501	-408.56532	0.25168	-408.31364
**QQ**	-328.53810	0.16554	-328.37257	-328.92050	0.18067	-328.73984
**NMe_3**	-174.05266	0.09490	-173.95776	-174.42322	0.11016	-174.31306
**NEt_3**	-291.69827	0.17417	-291.52410	-292.08040	0.18939	-291.89101
**$EtNH_2$**	-134.85571	0.06780	-134.78791	-135.21311	0.08264	-135.13046
**NH_3**	-56.44162	0.01649	-56.42513	-56.77788	0.03255	-56.74533
**iPr_2NH**	-291.71309	0.17235	-291.54074	-292.09113	0.18748	-291.90365
**$BnNH_2$**	-326.17010	0.11481	-326.05529	-326.53173	0.12946	-326.40227
**$PrNH_2$**	-174.06958	0.09422	-173.97536	-174.42905	0.10911	-174.31994
**PrNHMe**	-213.27392	0.12016	-213.15376	-213.64376	0.13544	-213.50832
**$PrNMe_2$**	-252.48213	0.14652	-252.33561	-252.85829	0.16172	-252.69657
**Ph_2NH**	-517.48794	0.16154	-517.32641	-517.84096	0.17604	-517.66492
**DMAn**	-365.37201	0.14082	-365.23119	-365.73954	0.15567	-365.58387
**1P**	-247.73648	0.06164	-247.67484	-248.09713	0.07576	-248.02137
**2P**	-286.95860	0.08620	-286.87240	-287.32531	0.10009	-287.22523
**3P**	-326.18069	0.11013	-326.07056	-326.55303	0.12388	-326.42915
**4P**	-365.40095	0.13466	-365.26629	-365.77860	0.14915	-365.62945
**5P**	-362.05867	0.09111	-361.96756	-362.43045	0.10506	-362.32539

**6P**	-303.00620	0.07689	-302.92932	-303.37193	0.08992	-303.28200
**7P**	-302.99703	0.07653	-302.92051	-303.36846	0.08976	-303.27870
**8P**	-303.00023	0.07680	-302.92343	-303.37988	0.09101	-303.28887
**9P**	-381.41008	0.12895	-381.28113	-381.79854	0.14342	-381.65512
**10P**	-358.27550	0.09222	-358.18328	-358.64252	0.10194	-358.54058
**11P**	-358.26900	0.09288	-358.17612	-358.63823	0.10601	-358.53222
**1An**	-286.96317	0.08823	-286.87494	-287.30698	0.10131	-287.20567
**2An**	-326.18381	0.11448	-326.06933	-326.52960	0.12780	-326.40180
**3An**	-746.07521	0.07674	-745.99847	-746.41445	0.09018	-746.32427
**4An**	-1205.18643	0.06522	-1205.12122	-1205.52160	0.07867	-1205.44293
**5An**	-2858.90330	0.07497	-2858.82833	-2859.24113	0.08756	-2859.15356
**6An**	-401.28017	0.11717	-401.16300	-401.63074	0.13044	-401.50030
**7An**	-623.59021	0.08657	-623.50364	-623.92099	0.09925	-623.82174
**8An**	-491.17929	0.08675	-491.09254	-491.51360	0.10089	-491.41271
**9An**	-491.18000	0.08677	-491.09323	-491.50531	0.09940	-491.40591
**10An**	-491.18011	0.08695	-491.09316	-491.50473	0.09878	-491.40595

Table S4. Reduced GBs, solvation energies ($\Delta G°_s$) and the reduced Gibbs energy of dissociation in solution ($\Delta G'_{a,sol}$(**B**H$^+$)) calculated for a set of the nitrogen bases. All energies are given in kcal mol^{-1} (1 kcal = 4.184 kJ)

Base	GB' (M1)	GB' (M6)	$\Delta G°_s$(**B**)	$\Delta G°_s$(**B**H$^+$)	$\Delta(\Delta G°_s)$	$\Delta G'_{a,sol}$(**B**H$^+$) (M1)	$\Delta G'_{a,sol}$(**B**H$^+$) (M6)
IPCM//M1 and IPCM//M6							
**P4*tBu**	298.7	291.3	-2.3	-26.6	-24.3	323.0	315.6
**P3*tBu**	288.9	282.4	-2.5	-28.5	-26.0	314.9	308.4
**P2*tBu**	273.2	268.3	-1.8	-31.6	-29.8	303.1	298.1
**P2*Et**	274.2	269.5	-2.3	-32.2	-30.0	304.2	299.5
**P2*Me**	274.6	270.2	-3.0	-32.7	-29.7	304.3	299.9
**P1*et_pyr**	263.9	260.4	-2.8	-34.4	-31.6	295.5	292.0
**P1*H_pyr**	261.2	257.0	-5.0	-36.5	-31.5	292.7	288.5
**P1*Ph**	252.0	248.7	-4.3	-36.8	-32.5	284.5	281.2
**P1*tBu**	258.6	254.2	-1.3	-36.2	-35.0	293.5	289.2
**P1*Me**	259.3	255.1	-2.7	-38.3	-35.6	294.9	290.7
**P1*H**	256.3	251.8	-4.0	-40.0	-36.0	292.4	287.8
**MTTT**	264.7	257.4	-7.2	-38.2	-31.1	295.8	288.5
**BGV**	244.9	242.9	-6.5	-44.5	-38.0	282.9	280.9
**MTBD**	254.5	249.2	-3.3	-40.9	-37.6	292.1	286.8
**TBD**	253.2	247.7	-4.6	-43.6	-39.1	292.3	286.8
**TMG*Ph**	247.1	242.3	-3.6	-40.2	-36.7	283.7	279.0
**TMG*Me**	250.8	246.4	-2.6	-42.4	-39.8	290.7	286.2
**TMG*H**	247.4	242.6	-3.6	-44.7	-41.0	288.4	283.7
**DBU**	250.9	245.9	-3.9	-41.9	-38.1	289.0	284.0
**DBN**	248.4	243.5	-4.0	-44.2	-40.1	288.5	283.6
**pyr*Me**	228.8	226.6	-1.6	-49.9	-48.3	277.1	274.9
**pyr*H**	225.9	223.5	-2.2	-54.8	-52.5	278.4	276.0
**pp*Me**	229.8	227.6	-1.0	-48.0	-47.0	276.7	274.6
**pp*H**	226.3	224.0	-1.5	-52.6	-51.2	277.5	275.1
**TMP**	235.1	231.3	-1.1	-44.0	-42.9	278.0	274.2
**QQ**	233.5	230.5	-1.7	-47.8	-46.1	279.6	276.6
**NMe_3**	224.1	223.0	-1.2	-53.4	-52.2	276.3	275.2
**NEt_3**	232.5	230.2	-0.7	-45.8	-45.1	277.5	275.3
**$EtNH_2$**	216.5	215.0	-2.2	-62.0	-59.7	276.2	274.7
**NH_3**	201.1	200.9	-4.4	-79.5	-75.1	276.3	276.1
**iPr_2NH**	230.4	227.7	-1.2	-47.7	-46.5	276.9	274.2
**$BnNH_2$**	219.8	217.7	-3.4	-56.1	-52.7	272.5	270.4
**$PrNH_2$**	217.8	216.2	-2.5	-59.9	-57.4	275.2	273.6
**PrNHMe**	224.2	222.5	-1.6	-53.9	-52.2	276.4	274.7
**$PrNMe_2$**	228.2	226.5	-1.1	-49.2	-48.1	276.3	274.6
**Ph_2NH**	213.6	212.4	-3.4	-45.7	-42.3	255.9	254.7
**DMAn**	221.8	221.3	-2.5	-47.1	-44.6	266.4	265.9
**1P**	222.2	217.5	-3.0	-52.1	-49.1	271.3	266.6
**2P**	226.7	221.4	-2.6	-48.8	-46.2	272.9	267.6
**3P**	230.8	225.0	-2.3	-46.1	-43.8	274.5	268.8

**4P**	234.2	227.9	-2.5	-44.0	-41.4	275.6	269.3
**5P**	230.5	224.5	-4.1	-48.3	-44.2	274.7	268.7
**6P**	227.7	221.3	-4.2	-51.5	-47.3	275.0	268.6
**7P**	229.5	224.8	-5.3	-50.6	-45.3	274.9	270.1
**8P**	235.7	229.3	-5.7	-50.1	-44.4	280.1	273.7
**9P**	240.5	234.7	-4.8	-44.4	-39.6	280.0	274.3
**10P**	231.6	224.2	-5.5	-51.2	-45.7	277.4	270.0
**11P**	230.1	223.5	-5.1	-50.4	-45.3	275.4	268.8
**1An**	209.2	207.5	-3.6	-57.7	-54.1	263.3	261.7
**2An**	210.9	208.6	-3.4	-55.3	-52.0	262.8	260.6
**3An**	206.0	204.4	-3.1	-55.5	-52.3	258.3	256.8
**4An**	203.2	201.9	-2.4	-53.3	-50.9	254.1	252.8
**5An**	205.6	204.1	-3.9	-59.0	-55.0	260.7	259.1
**6An**	214.4	211.7	-4.7	-55.7	-51.1	265.5	262.7
**7An**	200.4	199.6	-4.8	-62.7	-57.9	258.4	257.6
**8An**	200.2	200.9	-6.3	-57.7	-51.4	251.6	252.3
**9An**	197.5	196.2	-7.0	-67.0	-60.0	257.5	256.2
**10An**	194.9	196.3	-8.5	-68.1	-59.6	254.5	255.9

Table S4. (contd.)

Base	GB' (M1)	GB' (M6)	$\Delta G°_s$(**B**)	$\Delta G°_s$(**B**H$^+$)	$\Delta(\Delta G°_s)$	$\Delta G'_{a,sol}$(**B**H$^+$) (M1)	$\Delta G'_{a,sol}$(**B**H$^+$) (M6)
CPCM//M1 and CPCM//M6							
**P4*tBu**	298.7	291.3	-18.1	-42.7	-24.6	323.3	315.9
**P3*tBu**	288.9	282.4	-14.0	-42.1	-28.1	316.9	310.4
**P2*tBu**	273.2	268.3	-10.2	-43.4	-33.2	306.4	301.5
**P2*Et**	274.2	269.5	-10.7	-43.5	-32.8	307.1	302.3
**P2*Me**	274.6	270.2	-10.5	-43.3	-32.9	307.4	303.0
**P1*et_pyr**	263.9	260.4	-12.6	-54.8	-42.2	306.1	302.6
**P1*H_pyr**	261.2	257.0	-13.6	-50.8	-37.3	298.5	294.3
**P1*Ph**	252.0	248.7	-11.5	-49.7	-38.3	290.3	287.0
**P1*tBu**	258.6	254.2	-6.6	-48.1	-41.6	300.1	295.7
**P1*Me**	259.3	255.1	-7.0	-48.7	-41.7	301.0	296.7
**P1*H**	256.3	251.8	-7.9	-50.3	-42.4	298.7	294.2
**MTTT**	264.7	257.4	-16.6	-52.9	-36.4	301.1	293.8
**BGV**	244.9	242.9	-20.0	-62.7	-42.6	287.5	285.6
**MTBD**	254.5	249.2	-11.5	-55.8	-44.3	298.8	293.5
**TBD**	253.2	247.7	-12.6	-58.5	-45.8	299.1	293.6
**TMG*Ph**	247.1	242.3	-11.4	-54.8	-43.4	290.5	285.8
**TMG*Me**	250.8	246.4	-7.9	-54.5	-46.6	297.4	293.0
**TMG*H**	247.4	242.6	-9.2	-57.1	-47.9	295.3	290.6
**DBU**	250.9	245.9	-10.2	-56.0	-45.8	296.7	291.7
**DBN**	248.4	243.5	-9.6	-56.8	-47.2	295.5	290.7
**pyr*Me**	228.8	226.6	-4.0	-62.8	-58.8	287.6	285.4
**pyr*H**	225.9	223.5	-5.2	-66.7	-61.5	287.4	285.0
**pp*Me**	229.8	227.6	-4.4	-62.1	-57.6	287.4	285.2
**pp*H**	226.3	224.0	-4.9	-66.0	-61.1	287.4	285.1
**TMP**	235.1	231.3	-5.5	-59.7	-54.2	289.3	285.5
**QQ**	233.5	230.5	-5.5	-61.3	-55.8	289.3	286.3
**NMe_3**	224.1	223.0	-2.9	-65.1	-62.3	286.4	285.2
**NEt_3**	232.5	230.2	-4.0	-59.8	-55.8	288.2	286.0
**$EtNH_2$**	216.5	215.0	-4.2	-72.5	-68.3	284.8	283.3
**NH_3**	201.1	200.9	-4.1	-82.0	-77.9	279.1	278.9
**iPr_2NH**	230.4	227.7	-4.5	-62.2	-57.7	288.1	285.4
**$BnNH_2$**	219.8	217.7	-8.3	-70.7	-62.5	282.3	280.2
**$PrNH_2$**	217.8	216.2	-4.8	-71.7	-66.9	284.6	283.1
**PrNHMe**	224.2	222.5	-4.2	-66.8	-62.6	286.8	285.0
**$PrNMe_2$**	228.2	226.5	-3.7	-62.7	-58.9	287.1	285.4
**Ph_2NH**	213.6	212.4	-11.2	-63.0	-51.7	265.4	264.1
**DMAn**	221.8	221.3	-7.3	-61.9	-54.6	276.5	275.9
**1P**	222.2	217.5	-5.9	-62.4	-56.5	278.7	273.9
**2P**	226.7	221.4	-6.1	-60.0	-53.9	280.7	275.3
**3P**	230.8	225.0	-6.2	-57.9	-51.7	282.5	276.7
**4P**	234.2	227.9	-6.6	-56.0	-49.5	283.7	277.3
**5P**	230.5	224.5	-7.1	-58.4	-51.4	281.8	275.9

**6P**	227.7	221.3	-9.1	-62.9	-53.8	281.5	275.1
**7P**	229.5	224.8	-9.5	-60.7	-51.2	280.8	276.0
**8P**	235.7	229.3	-10.1	-60.0	-50.0	285.7	279.3
**9P**	240.5	234.7	-9.0	-55.5	-46.5	286.9	281.2
**10P**	231.6	224.2	-12.3	-64.0	-51.7	283.4	275.9
**11P**	230.1	223.5	-10.8	-62.0	-51.2	281.3	274.7
**1An**	209.2	207.5	-8.5	-71.1	-62.6	271.7	270.1
**2An**	210.9	208.6	-8.7	-70.1	-61.4	272.3	270.0
**3An**	206.0	204.4	-8.2	-68.7	-60.5	266.5	264.9
**4An**	203.2	201.9	-7.5	-66.8	-59.3	262.5	261.2
**5An**	205.6	204.1	-9.8	-74.5	-64.6	270.2	268.7
**6An**	214.4	211.7	-9.1	-69.1	-60.0	274.4	271.6
**7An**	200.4	199.6	-8.4	-75.0	-66.5	266.9	266.1
**8An**	200.2	200.9	-8.7	-68.3	-59.6	259.8	260.5
**9An**	197.5	196.2	-10.2	-79.7	-69.5	267.0	265.7
**10An**	194.9	196.3	-11.6	-80.7	-69.1	264.0	265.4

Table S4. (contd.)

Base	GB' (M1)	GB' (M6)	$\Delta G°_s$(**B**)	$\Delta G°_s$(**B**H^+)	$\Delta(\Delta G°_s)$	$\Delta G'_{a,sol}$(**B**H^+) (M1)	$\Delta G'_{a,sol}$(**B**H^+) (M6)
SMD1//M1 and SMD1//M6							
**P4*tBu**	298.7	291.3	-20.7	-43.7	-23.0	321.6	314.3
**P3*tBu**	288.9	282.4	-15.8	-42.4	-26.5	315.4	308.9
**P2*tBu**	273.2	268.3	-11.3	-43.3	-31.9	305.2	300.2
**P2*Et**	274.2	269.5	-11.8	-43.6	-31.9	306.1	301.4
**P2*Me**	274.6	270.2	-11.5	-43.2	-31.7	306.3	301.9
**P1*et_pyr**	263.9	260.4	-15.9	-49.1	-33.2	297.1	293.7
**P1*H_pyr**	261.2	257.0	-14.5	-50.7	-36.3	297.5	293.3
**P1*Ph**	252.0	248.7	-12.3	-49.9	-37.5	289.6	286.2
**P1*tBu**	258.6	254.2	-7.2	-47.8	-40.6	299.2	294.8
**P1*Me**	259.3	255.1	-7.6	-48.4	-40.8	300.1	295.8
**P1*H**	256.3	251.8	-8.6	-50.1	-41.6	297.9	293.4
**MTTT**	264.7	257.4	-17.4	-53.2	-35.9	300.6	293.3
**BGV**	244.9	242.9	-21.2	-63.6	-42.4	287.2	285.3
**MTBD**	254.5	249.2	-12.1	-55.7	-43.6	298.2	292.8
**TBD**	253.2	247.7	-13.2	-58.5	-45.2	298.5	293.0
**TMG*Ph**	247.1	242.3	-12.2	-55.0	-42.8	289.9	285.2
**TMG*Me**	250.8	246.4	-8.3	-54.3	-46.0	296.8	292.4
**TMG*H**	247.4	242.6	-9.6	-57.0	-47.5	294.9	290.1
**DBU**	250.9	245.9	-10.5	-55.8	-45.2	296.2	291.1
**DBN**	248.4	243.5	-10.0	-56.6	-46.6	295.0	290.1
**pyr*Me**	228.8	226.6	-4.3	-62.5	-58.1	286.9	284.7
**pyr*H**	225.9	223.5	-5.5	-66.5	-61.0	286.9	284.5
**pp*Me**	229.8	227.6	-4.7	-61.7	-57.0	286.7	284.6
**pp*H**	226.3	224.0	-5.2	-65.7	-60.6	286.9	284.5
**TMP**	235.1	231.3	-5.8	-59.4	-53.6	288.7	284.9
**QQ**	233.5	230.5	-6.2	-61.0	-54.8	288.2	285.2
**NMe_3**	224.1	223.0	-3.1	-64.9	-61.8	285.9	284.7
**NEt_3**	232.5	230.2	-4.4	-59.5	-55.1	287.6	285.4
**$EtNH_2$**	216.5	215.0	-4.5	-72.4	-68.0	284.4	282.9
**NH_3**	201.1	200.9	-4.4	-82.0	-77.7	278.8	278.6
**iPr_2NH**	230.4	227.7	-4.7	-61.9	-57.1	287.5	284.8
**$BnNH_2$**	219.8	217.7	-8.9	-71.3	-62.4	282.2	280.1
**$PrNH_2$**	217.8	216.2	-5.1	-71.6	-66.5	284.3	282.8
**PrNHMe**	224.2	222.5	-4.5	-66.5	-62.1	286.3	284.6
**$PrNMe_2$**	228.2	226.5	-4.0	-62.4	-58.4	286.5	284.9
**Ph_2NH**	213.6	212.4	-12.3	-63.7	-51.4	265.0	263.8
**DMAn**	221.8	221.3	-8.0	-62.1	-54.2	276.0	275.5
**1P**	222.2	217.5	-6.4	-62.7	-56.3	278.6	273.8
**2P**	226.7	221.4	-6.5	-60.2	-53.7	280.4	275.1
**3P**	230.8	225.0	-6.7	-58.0	-51.3	282.0	276.3
**4P**	234.2	227.9	-7.0	-56.3	-49.3	283.5	277.1
**5P**	230.5	224.5	-7.5	-58.9	-51.4	281.9	276.0

**6P**	227.7	221.3	-9.8	-63.4	-53.6	281.3	274.9
**7P**	229.5	224.8	-10.1	-61.3	-51.2	280.8	276.0
**8P**	235.7	229.3	-10.6	-60.5	-49.9	285.6	279.2
**9P**	240.5	234.7	-9.5	-55.9	-46.4	286.9	281.1
**10P**	231.6	224.2	-13.1	-64.6	-51.5	283.2	275.7
**11P**	230.1	223.5	-11.5	-62.6	-51.2	281.3	274.6
**1An**	209.2	207.5	-9.3	-71.8	-62.5	271.7	270.1
**2An**	210.9	208.6	-9.5	-70.6	-61.2	272.0	269.8
**3An**	206.0	204.4	-8.9	-69.3	-60.4	266.4	264.9
**4An**	203.2	201.9	-8.0	-67.3	-59.3	262.5	261.2
**5An**	205.6	204.1	-10.5	-75.3	-64.8	270.4	268.9
**6An**	214.4	211.7	-10.0	-70.0	-60.0	274.4	271.7
**7An**	200.4	199.6	-9.1	-76.0	-66.8	267.3	266.5
**8An**	200.2	200.9	-9.2	-68.8	-59.6	259.8	260.5
**9An**	197.5	196.2	-10.8	-80.5	-69.7	267.2	265.9
**10An**	194.9	196.3	-11.9	-81.5	-69.6	264.5	265.9

Table S4. (contd.)

Base	GB' (M1)	GB' (M6)	$\Delta G°_s$(**B**)	$\Delta G°_s$(**B**H$^+$)	$\Delta(\Delta G°_s)$	$\Delta G'_{a,sol}$(**B**H$^+$) (M1)	$\Delta G'_{a,sol}$(**B**H$^+$) (M6)
SMD2//M3 and SMD2/M5							
**P4*tBu**	293.0	288.5	-16.7	-43.2	-26.4	319.4	315.0
**P3*tBu**	287.1	283.8	-13.6	-43.8	-30.2	317.3	314.0
**P2*tBu**	272.5	268.6	-10.8	-45.2	-34.4	306.9	303.0
**P2*Et**	271.2	268.1	-11.1	-45.3	-34.2	305.4	302.3
**P2*Me**	271.7	268.9	-10.9	-45.2	-34.3	306.0	303.2
**P1*et_pyr**	264.2	261.1	-13.2	-50.3	-37.1	301.3	298.2
**P1*H_pyr**	261.8	259.3	-14.1	-52.0	-38.0	299.7	297.3
**P1*Ph**	251.3	248.9	-12.9	-51.1	-38.2	289.6	287.1
**P1*tBu**	259.1	255.2	-8.1	-49.1	-41.0	300.1	296.2
**P1*Me**	258.3	255.3	-8.5	-49.8	-41.3	299.7	296.7
**P1*H**	254.8	252.4	-9.5	-51.7	-42.3	297.1	294.7
**MTTT**	261.8	257.8	-18.7	-54.4	-35.7	297.5	293.4
**BGV**	246.0	243.9	-22.0	-65.0	-42.9	289.0	286.9
**MTBD**	252.4	249.1	-12.8	-56.4	-43.6	296.0	292.6
**TBD**	251.1	247.8	-14.1	-59.2	-45.1	296.2	292.9
**TMG*Ph**	245.2	242.5	-12.6	-56.1	-43.6	288.8	286.1
**TMG*Me**	248.6	246.1	-8.8	-55.2	-46.3	294.9	292.5
**TMG*H**	245.1	242.9	-10.4	-57.9	-47.5	292.7	290.4
**DBU**	248.9	246.1	-11.6	-56.5	-44.9	293.8	291.0
**DBN**	246.2	243.6	-11.0	-57.2	-46.3	292.5	289.9
**pyr*Me**	227.5	226.3	-4.9	-63.1	-58.2	285.8	284.6
**pyr*H**	224.1	223.6	-6.2	-67.1	-61.0	285.1	284.6
**pp*Me**	228.8	227.6	-5.3	-62.4	-57.1	285.9	284.8
**pp*H**	225.0	224.3	-5.7	-66.4	-60.7	285.7	285.0
**TMP**	233.3	231.6	-6.4	-60.6	-54.3	287.5	285.8
**QQ**	230.6	229.3	-6.4	-61.8	-55.4	286.0	284.7
**NMe_3**	223.3	223.0	-3.7	-65.5	-61.8	285.1	284.7
**NEt_3**	231.7	230.2	-4.9	-60.4	-55.5	287.2	285.7
**$EtNH_2$**	215.2	215.4	-5.0	-73.0	-68.0	283.2	283.3
**NH_3**	200.8	202.3	-5.0	-82.3	-77.3	278.1	279.7
**iPr_2NH**	229.2	228.1	-5.4	-63.0	-57.6	286.8	285.7
**$BnNH_2$**	217.6	217.9	-9.7	-72.6	-62.9	280.5	280.8
**$PrNH_2$**	216.5	216.7	-5.7	-72.3	-66.6	283.1	283.3
**PrNHMe**	223.0	222.7	-5.1	-67.3	-62.3	285.3	285.0
**$PrNMe_2$**	227.3	226.5	-4.6	-63.2	-58.6	285.9	285.2
**Ph_2NH**	211.9	212.4	-12.6	-65.1	-52.5	264.5	265.0
**DMAn**	220.9	221.7	-8.3	-63.2	-54.9	275.9	276.6
**1P**	219.4	217.6	-7.0	-63.4	-56.4	275.9	274.0
**2P**	224.2	221.7	-7.1	-61.0	-53.9	278.0	275.6
**3P**	227.9	225.0	-7.3	-58.8	-51.5	279.4	276.5
**4P**	231.6	228.1	-7.8	-57.0	-49.2	280.8	277.3
**5P**	227.2	224.7	-8.3	-59.9	-51.6	278.8	276.3

**6P**	224.7	221.3	-10.4	-64.4	-54.1	278.8	275.4
**7P**	225.9	224.9	-10.9	-62.4	-51.5	277.4	276.4
**8P**	232.6	229.3	-11.5	-61.5	-50.1	282.7	279.4
**9P**	237.1	234.5	-10.4	-56.7	-46.3	283.4	280.8
**10P**	228.4	224.2	-13.8	-66.1	-52.4	280.7	276.5
**11P**	226.9	223.6	-12.2	-63.9	-51.8	278.6	275.4
**1An**	207.6	207.9	-9.6	-72.8	-63.2	270.8	271.1
**2An**	209.4	209.1	-9.8	-71.8	-62.1	271.5	271.2
**3An**	204.7	205.0	-9.1	-70.2	-61.1	265.8	266.1
**4An**	202.0	202.2	-8.1	-67.9	-59.7	261.8	262.0
**5An**	203.7	204.1	-10.9	-76.5	-65.6	269.3	269.7
**6An**	212.2	211.8	-10.5	-71.4	-60.9	273.1	272.6
**7An**	198.9	199.8	-9.9	-78.0	-68.0	266.9	267.8
**8An**	199.0	201.1	-9.9	-68.9	-59.1	258.1	260.1
**9An**	195.7	196.2	-11.5	-82.1	-70.6	266.3	266.7
**10An**	193.6	195.9	-12.6	-83.2	-70.6	264.2	266.5

S6. P-Bases

Table S5. Electronic energies (E_{el}), Gibbs corrections (G_{corr}) and total Gibbs energies (G_{tot}) calculated for a set of the phosphorus bases. All energies are given in atomic units (a.u.).

Base B	E_{el}(**B**)	G_{corr} (**B**)	G_{tot}(**B**)	E_{el}(**B**H$^+$)	G_{corr} (**B**H$^+$)	G_{tot}(**B**H$^+$)
	M1					
**3**	-461.17450	0.08521	-461.08929	-461.54819	0.09589	-461.45230
**4**	-579.14443	0.16397	-578.98046	-579.52729	0.17448	-579.35282
**5**	-652.96331	0.13185	-652.83146	-653.34245	0.14192	-653.20053
**6**	-844.75309	0.18009	-844.57299	-845.13550	0.19079	-844.94471
**7**	-1036.54287	0.22744	-1036.31543	-1036.92860	0.23791	-1036.69070
**8**	-1047.41477	0.43760	-1046.97717	-1047.81676	0.44801	-1047.36875
**10**	-916.14504	0.27265	-915.87240	-916.57364	0.28833	-916.28531
**11**	-500.48278	0.10934	-500.37344	-500.82663	0.12042	-500.70621
**12**	-657.79043	0.21590	-657.57452	-658.16065	0.22639	-657.93426
**13**	-815.09803	0.32221	-814.77582	-815.48724	0.33489	-815.15236
**14**	-815.05627	0.33028	-814.72599	-815.45743	0.34012	-815.11731
**15**	-592.76650	0.10670	-592.65980	-593.12928	0.11733	-593.01195
**16**	-805.42039	0.15235	-805.26804	-805.78798	0.16349	-805.62450
**17**	-1334.36506	0.19946	-1334.16560	-1334.74048	0.20947	-1334.53102
**18**	-2415.42464	0.19192	-2415.23272	-2415.79843	0.20203	-2415.59640
**19**	-1154.53144	0.30050	-1154.23094	-1154.92599	0.31164	-1154.61435
**20**	-1380.23435	0.31604	-1379.91830	-1380.63609	0.32580	-1380.31030
**21**	-1135.81667	0.21908	-1135.59759	-1136.19988	0.22960	-1135.97028
**22**	-1235.09008	0.20977	-1234.88031	-1235.47100	0.22075	-1235.25025
**23**	-1334.36303	0.19963	-1334.16340	-1334.74182	0.21111	-1334.53071
**24**	-1235.08494	0.21017	-1234.87477	-1235.46788	0.22087	-1235.24701
**25**	-1433.62781	0.19155	-1433.43626	-1434.00585	0.20237	-1433.80348
**26**	-1632.17107	0.17350	-1631.99757	-1632.54484	0.18513	-1632.35971
**27**	-3794.26302	0.15953	-3794.10350	-3794.64443	0.17141	-3794.47302
**28**	-1497.59603	0.35934	-1497.23669	-1497.99023	0.37152	-1497.61871
**29**	-921.13712	0.16950	-920.96762	-921.51606	0.17993	-921.33612
**30**	-1688.29395	0.36164	-1687.93232	-1688.68275	0.37179	-1688.31096
**31**	-1727.61935	0.38781	-1727.23154	-1728.00766	0.39817	-1727.60949
**32**	-1766.94560	0.41409	-1766.53150	-1767.33303	0.42569	-1766.90734
**33**	-2071.87708	0.45706	-2071.42001	-2072.27491	0.47020	-2071.80471
**34**	-2379.24467	0.54635	-2378.69832	-2379.64446	0.55725	-2379.08721
**35**	-2741.49845	0.69033	-2740.80812	-2741.98122	0.70440	-2741.27681
**36**	-3438.47239	1.00760	-3437.46479	-3438.97128	1.02323	-3437.94805
**37**	-1427.72930	0.47176	-1427.25754	-1428.18869	0.48614	-1427.70255
**38**	-1651.30612	0.63011	-1650.67601	-1651.76130	0.64250	-1651.11742
**39**	-2128.39876	0.82235	-2127.57641	-2128.87974	0.83889	-2128.04085

Table S5. (contd.)

Base B	E_{el}(**B**)	G_{corr} (**B**)	G_{tot}(**B**)	E_{el}(**B**H$^+$)	G_{corr} (**B**H$^+$)	G_{tot}(**B**H$^+$)
	M2					
**3**	-461.18005	0.08473	-461.09532	-461.55432	0.09551	-461.45881
**4**	-579.15922	0.16396	-578.99527	-579.54346	0.17458	-579.36888
**5**	-652.97638	0.13134	-652.84504	-653.35620	0.14154	-653.21466
**6**	-844.77457	0.17967	-844.59490	-845.15813	0.19024	-844.96789
**7**	-1036.57384	0.22775	-1036.34609	-1036.96057	0.23727	-1036.72330
**8**	-1047.47463	0.43879	-1047.03584	-1047.87792	0.44906	-1047.42886
**10**	-916.18739	0.27264	-915.91475	-916.61350	0.28852	-916.32498
**11**	-500.49322	0.10931	-500.38392	-500.83833	0.12045	-500.71788
**12**	-657.81256	0.21593	-657.59664	-658.18447	0.22692	-657.95756
**13**	-815.13465	0.32262	-814.81203	-815.52754	0.33547	-815.19207
**14**	-815.10477	0.33119	-814.77358	-815.50810	0.34168	-815.16641
**15**	-592.77690	0.10646	-592.67044	-593.14180	0.11730	-593.02450
**16**	-805.43803	0.15178	-805.28625	-805.80680	0.16341	-805.64339
**17**	-1334.39783	0.19902	-1334.19881	-1334.77426	0.20859	-1334.56567
**18**	-2415.46195	0.19214	-2415.26981	-2415.83661	0.20154	-2415.63507
**19**	-1154.57083	0.30042	-1154.27041	-1154.96651	0.30961	-1154.65690
**20**	-1380.27858	0.31565	-1379.96294	-1380.68133	0.32629	-1380.35505
**21**	-1135.84859	0.21874	-1135.62985	-1136.23280	0.22837	-1136.00443
**22**	-1235.12307	0.20944	-1234.91363	-1235.50500	0.21976	-1235.28524
**23**	-1334.39696	0.19974	-1334.19722	-1334.77692	0.21089	-1334.56604
**24**	-1235.11781	0.21136	-1234.90645	-1235.50237	0.22080	-1235.28157
**25**	-1433.66378	0.19126	-1433.47252	-1434.04205	0.20090	-1433.84115
**26**	-1632.20852	0.17277	-1632.03575	-1632.58315	0.18309	-1632.40006
**27**	-3794.32124	0.15943	-3794.16181	-3794.70535	0.17158	-3794.53377
**28**	-1497.65148	0.35926	-1497.29222	-1498.04853	0.37148	-1497.67705
**29**	-921.15329	0.16917	-920.98412	-921.53299	0.17945	-921.35354
**30**	-1688.34572	0.36156	-1687.98416	-1688.73550	0.37136	-1688.36414
**31**	-1727.67305	0.38779	-1727.28527	-1728.06273	0.39878	-1727.66395
**32**	-1767.00168	0.41417	-1766.58750	-1767.39047	0.42407	-1766.96640
**33**	-2071.95637	0.45835	-2071.49802	-2072.35793	0.46920	-2071.88873
**34**	-2379.34810	0.54806	-2378.80004	-2379.75164	0.55913	-2379.19251
**35**	-2741.62779	0.69020	-2740.93760	-2742.11220	0.70621	-2741.40599
**36**	-3438.67256	1.01390	-3437.65866	-3439.17446	1.02755	-3438.14691
**37**	-1427.80065	0.47065	-1427.33000	-1428.26152	0.48603	-1427.77549
**38**	-1651.40605	0.63203	-1650.77402	-1651.86724	0.64816	-1651.21908
**39**	-2128.53949	0.82924	-2127.71025	-2129.02086	0.84511	-2128.17575

Table S5. (contd.)

Base B	E_{el}(**B**)	G_{corr} (**B**)	G_{tot}(**B**)	E_{el}(**B**H$^+$)	G_{corr} (**B**H$^+$)	G_{tot}(**B**H$^+$)
	M3					
**3**	-461.06938	0.08412	-460.98526	-461.43691	0.09514	-461.34177
**4**	-578.98568	0.16200	-578.82368	-579.36272	0.17376	-579.18896
**5**	-652.79431	0.13174	-652.66257	-653.16551	0.14188	-653.02363
**6**	-844.52054	0.18079	-844.33975	-844.89439	0.19103	-844.70336
**7**	-1036.24683	0.22922	-1036.01761	-1036.62320	0.23947	-1036.38373
**8**	-1047.08788	0.43724	-1046.65064	-1047.48264	0.44703	-1047.03561
**10**	-915.89858	0.27256	-915.62602	-916.32011	0.28698	-916.03313
**11**	-500.36025	0.10869	-500.25156	-500.70014	0.11948	-500.58066
**12**	-657.59695	0.21475	-657.38220	-657.96205	0.22537	-657.73668
**13**	-814.83529	0.32030	-814.51500	-815.22135	0.33403	-814.88732
**14**	-814.81211	0.32821	-814.48390	-815.20686	0.33821	-814.86865
**15**	-592.62189	0.10568	-592.51621	-592.98077	0.11718	-592.86359
**16**	-805.20356	0.15223	-805.05133	-805.56325	0.16403	-805.39922
**17**	-1334.00457	0.20156	-1333.80302	-1334.37068	0.21174	-1334.15893
**18**	-2415.08019	0.19404	-2414.88615	-2415.44413	0.20387	-2415.24026
**19**	-1154.17982	0.30167	-1153.87815	-1154.56497	0.31119	-1154.25378
**20**	-1379.82647	0.31796	-1379.50850	-1380.21753	0.32871	-1379.88882
**21**	-1135.49966	0.22044	-1135.27922	-1135.87344	0.23047	-1135.64297
**22**	-1234.75232	0.21194	-1234.54039	-1235.12360	0.22155	-1234.90205
**23**	-1334.00432	0.20196	-1333.80237	-1334.37366	0.21258	-1334.16108
**24**	-1234.74579	0.21142	-1234.53437	-1235.12052	0.22203	-1234.89849
**25**	-1433.24908	0.19472	-1433.05436	-1433.61597	0.20356	-1433.41241
**26**	-1631.75017	0.17695	-1631.57322	-1632.11276	0.18832	-1631.92445
**27**	-3793.88263	0.16081	-3793.72182	-3794.25728	0.17253	-3794.08474
**28**	-1497.14746	0.36268	-1496.78478	-1497.53326	0.37414	-1497.15912
**29**	-920.94484	0.16719	-920.77765	-921.31739	0.17768	-921.13971
**30**	-1687.84889	0.36327	-1687.48562	-1688.22862	0.37327	-1687.85534
**31**	-1727.15537	0.38938	-1726.76599	-1727.53524	0.40079	-1727.13445
**32**	-1766.46317	0.41652	-1766.04666	-1766.84212	0.42544	-1766.41669
**33**	-2071.31176	0.46171	-2070.85005	-2071.70251	0.47302	-2071.22949
**34**	-2378.58422	0.55351	-2378.03071	-2378.97629	0.56379	-2378.41250
**35**	-2740.85474	0.69147	-2740.16327	-2741.32851	0.70067	-2740.62784
**36**	-3437.61465	1.01203	-3436.60262	-3438.10492	1.02638	-3437.07855
**37**	-1427.28144	0.47159	-1426.80985	-1427.73224	0.48664	-1427.24560
**38**	-1650.76158	0.63312	-1650.12846	-1651.21264	0.64807	-1650.56457
**39**	-2127.68698	0.82863	-2126.85835	-2128.15495	0.83706	-2127.31789

Table S5. (contd.)

Base B	E_{el}(**B**)	G_{corr} (**B**)	G_{tot}(**B**)	E_{el}(**B**H$^+$)	G_{corr} (**B**H$^+$)	G_{tot}(**B**H$^+$)
	M5					
**3**	-460.30508	0.08412	-460.22097	-460.67466	0.09514	-460.57952
**4**	-577.94280	0.16200	-577.78080	-578.32050	0.17376	-578.14673
**5**	-651.61876	0.13174	-651.48702	-651.99155	0.14188	-651.84967
**6**	-842.93611	0.18079	-842.75532	-843.30996	0.19103	-843.11894
**7**	-1034.25579	0.22922	-1034.02657	-1034.63061	0.23947	-1034.39114
**8**	-1044.97750	0.43724	-1044.54025	-1045.37126	0.44703	-1044.92422
**10**	-914.19662	0.27256	-913.92405	-914.63341	0.28698	-914.34643
**11**	-499.50242	0.10869	-499.39373	-499.84405	0.11948	-499.72457
**12**	-656.36923	0.21475	-656.15448	-656.73491	0.22537	-656.50954
**13**	-813.24052	0.32030	-812.92023	-813.62543	0.33403	-813.29140
**14**	-813.22224	0.32821	-812.89404	-813.61533	0.33821	-813.27711
**15**	-591.59896	0.10568	-591.49328	-591.95925	0.11718	-591.84207
**16**	-803.70990	0.15223	-803.55767	-804.06969	0.16403	-803.90566
**17**	-1331.63770	0.20156	-1331.43614	-1332.00258	0.21174	-1331.79084
**18**	-2411.58708	0.19404	-2411.39304	-2411.95009	0.20387	-2411.74622
**19**	-1151.91430	0.30167	-1151.61263	-1152.29690	0.31119	-1151.98571
**20**	-1377.21632	0.31796	-1376.89835	-1377.60473	0.32871	-1377.27602
**21**	-1133.38345	0.22044	-1133.16301	-1133.75574	0.23047	-1133.52527
**22**	-1232.51075	0.21194	-1232.29881	-1232.88092	0.22155	-1232.65937
**23**	-1331.63738	0.20196	-1331.43543	-1332.00542	0.21258	-1331.79283
**24**	-1232.50429	0.21142	-1232.29287	-1232.87747	0.22203	-1232.65544
**25**	-1430.75685	0.19472	-1430.56213	-1431.12284	0.20356	-1430.91928
**26**	-1629.00803	0.17695	-1628.83108	-1629.36908	0.18832	-1629.18077
**27**	-3788.90595	0.16081	-3788.74514	-3789.27684	0.17253	-3789.10431
**28**	-1494.17678	0.36268	-1493.81410	-1494.55925	0.37414	-1494.18511
**29**	-919.42993	0.16719	-919.26274	-919.80349	0.17768	-919.62580
**30**	-1684.69886	0.36327	-1684.33559	-1685.07780	0.37327	-1684.70452
**31**	-1723.91141	0.38938	-1723.52203	-1724.29131	0.40079	-1723.89052
**32**	-1763.12540	0.41652	-1762.70888	-1763.50374	0.42544	-1763.07830
**33**	-2067.36485	0.46171	-2066.90315	-2067.75412	0.47302	-2067.28110
**34**	-2373.99391	0.55351	-2373.44040	-2374.38499	0.56379	-2373.82120
**35**	-2736.11171	0.69147	-2735.42024	-2736.58822	0.70067	-2735.88755
**36**	-3431.33790	1.01203	-3430.32587	-3431.82877	1.02638	-3430.80239
**37**	-1424.53286	0.47159	-1424.06127	-1424.98341	0.48664	-1424.49677
**38**	-1647.44719	0.63312	-1646.81407	-1647.90398	0.64807	-1647.25591
**39**	-2123.37901	0.82863	-2122.55038	-2123.85295	0.83706	-2123.01589

Table S5. (contd.)

Base B	E_{el}(**B**)	G_{corr} (**B**)	G_{tot}(**B**)	E_{el}(**B**H$^+$)	G_{corr} (**B**H$^+$)	G_{tot}(**B**H$^+$)
	M6					
**3**	-460.30472	0.08521	-460.21950	-460.67432	0.09589	-460.57843
**4**	-577.94230	0.16397	-577.77833	-578.31969	0.17448	-578.14521
**5**	-651.61833	0.13185	-651.48648	-651.99120	0.14192	-651.84928
**6**	-842.93529	0.18009	-842.75520	-843.30948	0.19079	-843.11869
**7**	-1034.25425	0.22744	-1034.02681	-1034.62975	0.23791	-1034.39185
**8**	-1044.97494	0.43760	-1044.53734	-1045.36848	0.44801	-1044.92047
**10**	-914.19524	0.27265	-913.92259	-914.63222	0.28833	-914.34389
**11**	-499.50268	0.10934	-499.39334	-499.84377	0.12042	-499.72335
**12**	-656.36864	0.21590	-656.15274	-656.73417	0.22639	-656.50778
**13**	-813.23931	0.32221	-812.91710	-813.62376	0.33489	-813.28888
**14**	-813.22039	0.33028	-812.89011	-813.61331	0.34012	-813.27318
**15**	-591.59864	0.10670	-591.49194	-591.95873	0.11733	-591.84140
**16**	-803.70931	0.15235	-803.55696	-804.06942	0.16349	-803.90593
**17**	-1331.63587	0.19946	-1331.43642	-1332.00151	0.20947	-1331.79204
**18**	-2411.58451	0.19192	-2411.39258	-2411.94846	0.20203	-2411.74643
**19**	-1151.91268	0.30050	-1151.61218	-1152.29581	0.31164	-1151.98417
**20**	-1377.21436	0.31604	-1376.89831	-1377.60372	0.32580	-1377.27792
**21**	-1133.38193	0.21908	-1133.16285	-1133.75490	0.22960	-1133.52530
**22**	-1232.50945	0.20977	-1232.29968	-1232.88007	0.22075	-1232.65932
**23**	-1331.63598	0.19963	-1331.43635	-1332.00469	0.21111	-1331.79358
**24**	-1232.50321	0.21017	-1232.29304	-1232.87610	0.22087	-1232.65523
**25**	-1430.75479	0.19155	-1430.56324	-1431.12193	0.20237	-1430.91956
**26**	-1629.00659	0.17350	-1628.83308	-1629.36859	0.18513	-1629.18346
**27**	-3788.90127	0.15953	-3788.74174	-3789.27285	0.17141	-3789.10144
**28**	-1494.17449	0.35934	-1493.81515	-1494.55801	0.37152	-1494.18650
**29**	-919.42892	0.16950	-919.25942	-919.80244	0.17993	-919.62251
**30**	-1684.69556	0.36164	-1684.33392	-1685.07517	0.37179	-1684.70338
**31**	-1723.90795	0.38781	-1723.52014	-1724.28728	0.39817	-1723.88912
**32**	-1763.12252	0.41409	-1762.70843	-1763.50119	0.42569	-1763.07550
**33**	-2067.35599	0.45706	-2066.89893	-2067.74642	0.47020	-2067.27622
**34**	-2373.97890	0.54635	-2373.43255	-2374.37032	0.55725	-2373.81307
**35**	-2736.10868	0.69033	-2735.41834	-2736.58658	0.70440	-2735.88217
**36**	-3431.32585	1.00760	-3430.31824	-3431.81789	1.02323	-3430.79466
**37**	-1424.53005	0.47176	-1424.05829	-1424.98142	0.48614	-1424.49528
**38**	-1647.44044	0.63011	-1646.81033	-1647.89343	0.64250	-1647.25093
**39**	-2123.36463	0.82235	-2122.54228	-2123.84755	0.83889	-2123.00866

Table S6. Reduced GBs, solvation energies ($\Delta G°_s$) and the reduced Gibbs energy of dissociation in solution ($\Delta G'_{a,sol}$(**B**H$^+$)) calculated for a set of the phosphorus bases. All energies are given in kcal mol^{-1} (1 kcal = 4.184 kJ)

**Base**	GB' (M1)	GB' (M6)	$\Delta G°_s$(**B**)	$\Delta G°_s$(**B**H$^+$)	$\Delta(\Delta G°_s)$	$\Delta G'_{a,sol}$(**B**H$^+$) (M1)	$\Delta G'_{a,sol}$(**B**H$^+$) (M6)
IPCM//M1 and IPCM//M6							
**3**	227.8	225.2	-1.3	-50.0	-48.68	276.48	273.9
**4**	233.7	230.2	-1.2	-44.0	-42.77	276.43	273.0
**5**	231.6	227.7	-2.2	-44.0	-41.80	273.39	269.5
**6**	233.3	228.1	-2.7	-39.2	-36.55	269.81	264.6
**7**	235.5	229.1	-3.1	-35.4	-32.33	267.82	261.4
**8**	245.7	240.4	-1.4	-34.9	-33.51	279.23	273.9
**10**	259.1	264.4	-1.6	-37.8	-36.20	295.31	300.6
**11**	208.8	207.1	-0.8	-51.6	-50.82	259.63	257.9
**12**	225.7	222.8	-1.0	-43.5	-42.46	268.20	265.2
**13**	236.3	233.3	-0.8	-38.4	-37.53	273.81	270.8
**14**	245.6	240.4	-1.1	-37.2	-36.12	281.68	276.5
**15**	221.0	219.3	-5.6	-53.8	-48.24	269.22	267.5
**16**	223.7	219.0	-2.5	-40.8	-38.27	261.96	257.3
**17**	229.3	223.2	-4.3	-40.9	-36.66	265.96	259.8
**18**	228.2	222.0	-3.2	-38.3	-35.09	263.30	257.1
**19**	240.6	233.4	-3.3	-32.5	-29.20	269.79	262.6
**20**	246.0	238.2	-5.9	-34.1	-28.13	274.11	266.3
**21**	233.9	227.4	-3.8	-35.6	-31.80	265.66	259.2
**22**	232.1	225.7	-4.4	-35.7	-31.31	263.45	257.0
**23**	230.5	224.2	-5.1	-35.8	-30.67	261.16	254.8
**24**	233.6	227.3	-4.1	-35.5	-31.35	264.94	258.6
**25**	230.4	223.6	-4.4	-35.9	-31.45	261.88	255.0
**26**	227.2	219.9	-5.4	-35.5	-30.08	257.33	249.9
**27**	231.9	225.7	-3.9	-32.3	-28.38	260.26	254.1
**28**	239.7	233.0	-4.2	-31.4	-27.24	266.96	260.3
**29**	231.2	227.8	-2.3	-45.7	-43.44	274.68	271.3
**30**	237.6	231.8	-4.6	-36.1	-31.53	269.13	263.4
**31**	237.2	231.5	-4.6	-36.3	-31.76	268.92	263.3
**32**	235.8	230.3	-4.6	-36.4	-31.75	267.59	262.1
**33**	241.4	236.8	-4.8	-30.7	-25.97	267.37	262.7
**34**	244.0	238.8	-5.5	-29.9	-24.32	268.35	263.1
**35**	294.1	291.1	-1.6	-26.8	-25.18	319.29	316.2
**36**	303.3	299.0	-3.0	-24.4	-21.39	324.64	320.3
**37**	279.2	274.2	-3.7	-29.6	-25.91	305.15	300.1
**38**	277.0	276.5	-4.5	-28.4	-23.90	300.89	300.4
**39**	291.4	292.7	-7.3	-25.7	-18.34	309.78	311.0

Table S6.(contd.)

Base	GB' (M1)	GB' (M6)	$\Delta G°_s$(**B**)	$\Delta G°_s$(**B**H^+)	$\Delta(\Delta G°_s)$	$\Delta G'_{a,sol}$(**B**H^+) (M1)	$\Delta G'_{a,sol}$(**B**H^+) (M6)
CPCM//M1 and CPCM//M6							
**3**	227.8	225.2	6.3	-43.3	-49.6	277.4	274.8
**4**	233.7	230.2	10.5	-33.5	-44.0	277.7	274.2
**5**	231.6	227.7	8.3	-34.2	-42.5	274.0	270.1
**6**	233.3	228.1	11.2	-26.2	-37.4	270.6	265.5
**7**	235.5	229.1	14.6	-18.0	-32.6	268.1	261.7
**8**	245.7	240.4	22.7	-12.4	-35.2	280.9	275.6
**10**	259.1	264.4	13.4	-22.7	-36.1	295.2	300.5
**11**	208.8	207.1	8.1	-44.8	-52.9	261.7	260.0
**12**	225.7	222.8	14.2	-31.0	-45.2	271.0	268.0
**13**	236.3	233.3	21.0	-19.2	-40.1	276.4	273.4
**14**	245.6	240.4	14.9	-21.8	-36.7	282.2	277.1
**15**	221.0	219.3	5.0	-44.6	-49.6	270.6	268.9
**16**	223.7	219.0	10.0	-29.4	-39.3	263.0	258.3
**17**	229.3	223.2	15.1	-21.0	-36.1	265.4	259.3
**18**	228.2	222.0	15.1	-21.9	-37.1	265.3	259.1
**19**	240.6	233.4	19.3	-10.5	-29.7	270.3	263.1
**20**	246.0	238.2	17.2	-10.1	-27.3	273.2	265.5
**21**	233.9	227.4	14.4	-17.6	-32.0	265.9	259.4
**22**	232.1	225.7	14.1	-17.3	-31.4	263.5	257.1
**23**	230.5	224.2	14.2	-16.6	-30.8	261.3	255.0
**24**	233.6	227.3	13.8	-18.2	-32.0	265.6	259.3
**25**	230.4	223.6	13.9	-17.5	-31.4	261.8	255.0
**26**	227.2	219.9	14.0	-16.8	-30.8	258.1	250.7
**27**	231.9	225.7	15.6	-12.9	-28.5	260.4	254.2
**28**	239.7	233.0	21.5	-6.1	-27.6	267.3	260.6
**29**	231.2	227.8	10.7	-34.5	-45.3	276.5	273.1
**30**	237.6	231.8	22.6	-10.2	-32.8	270.4	264.6
**31**	237.2	231.5	23.4	-10.2	-33.6	270.8	265.1
**32**	235.8	230.3	25.0	-9.3	-34.3	270.1	264.6
**33**	241.4	236.8	31.8	6.3	-25.6	267.0	262.3
**34**	244.0	238.8	37.5	13.1	-24.4	268.4	263.1
**35**	294.1	291.1	43.3	23.2	-20.0	314.1	311.1
**36**	303.3	299.0	58.1	42.1	-16.0	319.3	315.0
**37**	279.2	274.2	26.8	2.9	-23.9	303.1	298.1
**38**	277.0	276.5	35.1	13.4	-21.7	299.5	298.1
**39**	291.4	292.7	46.0	31.2	-14.8	306.2	307.4

Table S6.(contd.)

**Base**	GB' (M1)	GB' (M6)	$\Delta G°_s$(**B**)	$\Delta G°_s$(**B**H$^+$)	$\Delta(\Delta G°_s)$	$\Delta G'_{a,sol}$(**B**H$^+$) (M1)	$\Delta G'_{a,sol}$(**B**H$^+$) (M6)
SMD1//M1 and SMD1//M6							
**3**	227.8	225.2	-2.4	-59.7	-57.3	285.1	282.5
**4**	233.7	230.2	-4.4	-56.7	-52.3	286.0	282.5
**5**	231.6	227.7	-6.7	-57.2	-50.4	282.0	278.1
**6**	233.3	228.1	-10.5	-54.9	-44.5	277.7	272.5
**7**	235.5	229.1	-13.5	-53.0	-39.5	275.0	268.6
**8**	245.7	240.4	-9.8	-53.6	-43.8	289.5	284.2
**10**	259.1	264.4	-7.1	-50.8	-43.8	302.9	308.1
**11**	208.8	207.1	-2.5	-64.2	-61.7	270.5	268.8
**12**	225.7	222.8	-4.7	-58.3	-53.6	279.3	276.4
**13**	236.3	233.3	-6.1	-54.5	-48.4	284.7	281.7
**14**	245.6	240.4	-5.8	-51.5	-45.7	291.3	286.1
**15**	221.0	219.3	-7.9	-66.7	-58.7	279.7	278.0
**16**	223.7	219.0	-9.8	-56.7	-46.8	270.5	265.8
**17**	229.3	223.2	-11.7	-55.9	-44.2	273.5	267.3
**18**	228.2	222.0	-15.0	-60.0	-45.1	273.3	267.1
**19**	240.6	233.4	-14.4	-50.7	-36.3	276.9	269.8
**20**	246.0	238.2	-16.3	-50.5	-34.2	280.2	272.4
**21**	233.9	227.4	-13.3	-52.4	-39.0	272.9	266.4
**22**	232.1	225.7	-13.1	-51.5	-38.4	270.5	264.1
**23**	230.5	224.2	-13.0	-50.5	-37.4	267.9	261.6
**24**	233.6	227.3	-13.3	-51.2	-37.9	271.5	265.2
**25**	230.4	223.6	-12.4	-50.2	-37.8	268.2	261.4
**26**	227.2	219.9	-12.0	-47.9	-35.9	263.1	255.7
**27**	231.9	225.7	-16.9	-47.9	-31.0	262.8	256.7
**28**	239.7	233.0	-20.8	-54.8	-33.9	273.7	267.0
**29**	231.2	227.8	-5.2	-58.7	-53.5	284.7	281.3
**30**	237.6	231.8	-20.6	-60.5	-39.9	277.5	271.7
**31**	237.2	231.5	-21.4	-62.4	-40.9	278.1	272.5
**32**	235.8	230.3	-21.9	-63.2	-41.4	277.2	271.7
**33**	241.4	236.8	-25.4	-55.7	-30.4	271.8	267.1
**34**	244.0	238.8	-29.8	-59.2	-29.3	273.4	268.1
**35**	294.1	291.1	-20.3	-41.3	-21.0	315.1	312.0
**36**	303.3	299.0	-34.5	-51.4	-16.9	320.2	315.9
**37**	279.2	274.2	-22.0	-50.0	-28.0	307.3	302.2
**38**	277.0	276.5	-24.4	-49.9	-25.5	302.5	301.7
**39**	291.4	292.7	-36.2	-54.0	-17.8	309.2	310.5

Table S6.(contd.)

**Base**	GB' (M3)	GB' (M5)	$\Delta G°_s$(**B**)	$\Delta G°_s$(**B**H^+)	$\Delta(\Delta G°_s)$	$\Delta G'_{a,sol}$(**B**H^+) (M3)	$\Delta G'_{a,sol}$(**B**H^+) (M5)
SMD2//M3 and SMD2//M5							
**3**	223.7	225.0	-2.4	-60.3	-57.9	281.6	282.9
**4**	229.2	229.6	-4.4	-57.5	-53.0	282.2	282.7
**5**	226.6	227.6	-6.9	-58.5	-51.6	278.1	279.1
**6**	228.2	228.2	-10.5	-56.4	-45.9	274.1	274.1
**7**	229.7	228.8	-13.6	-54.3	-40.8	270.5	269.5
**8**	241.6	240.9	-9.9	-54.6	-44.6	286.2	285.6
**10**	255.5	265.0	-7.8	-52.3	-44.6	300.0	309.6
**11**	206.5	207.6	-2.5	-64.6	-62.1	268.6	269.7
**12**	222.4	222.8	-4.7	-59.1	-54.3	276.7	277.1
**13**	233.6	232.9	-6.1	-55.6	-49.4	283.1	282.3
**14**	241.4	240.4	-6.1	-52.8	-46.7	288.1	287.1
**15**	218.0	218.9	-8.7	-67.3	-58.6	276.6	277.5
**16**	218.3	218.4	-9.9	-58.1	-48.2	266.5	266.6
**17**	223.3	222.6	-13.0	-59.0	-46.0	269.3	268.6
**18**	222.2	221.6	-15.3	-61.0	-45.8	268.0	267.4
**19**	235.7	234.1	-14.6	-52.0	-37.4	273.2	271.6
**20**	238.7	237.0	-16.8	-52.4	-35.7	274.3	272.6
**21**	228.3	227.3	-13.8	-53.9	-40.2	268.4	267.5
**22**	226.9	226.3	-13.8	-53.4	-39.6	266.5	265.8
**23**	225.1	224.3	-13.9	-52.6	-38.7	263.8	262.9
**24**	228.5	227.5	-14.2	-53.2	-39.0	267.5	266.5
**25**	224.7	224.1	-13.6	-52.7	-39.1	263.8	263.2
**26**	220.4	219.4	-13.3	-50.8	-37.5	257.9	256.9
**27**	227.7	225.4	-17.0	-48.7	-31.8	259.5	257.2
**28**	234.9	232.8	-21.2	-56.2	-35.1	270.0	267.9
**29**	227.2	227.8	-5.2	-59.6	-54.5	281.7	282.3
**30**	232.0	231.5	-20.7	-61.9	-41.2	273.2	272.7
**31**	231.2	231.2	-21.5	-62.9	-41.4	272.6	272.6
**32**	232.2	231.8	-22.0	-64.9	-43.0	275.1	274.8
**33**	238.1	237.2	-24.9	-56.4	-31.6	269.7	268.7
**34**	239.6	239.0	-28.8	-59.9	-31.1	270.6	270.0
**35**	291.5	293.2	-16.6	-42.6	-26.0	317.5	319.3
**36**	298.6	299.0	-29.1	-49.4	-20.3	319.0	319.3
**37**	273.4	273.3	-20.4	-51.0	-30.6	304.0	303.9
**38**	273.7	277.3	-23.1	-49.8	-26.8	300.4	304.0
**39**	288.4	292.1	-31.2	-54.1	-23.0	311.3	315.1

Table S6.(contd.)

Base	GB' (M2)	$\Delta G°_s$(**B**)	$\Delta G°_s$(**B**H^+)	$\Delta(\Delta G°_s)$	$\Delta G'_{a,sol}$(**B**H^+) (M2)
SMD2//M2					
**3**	228.1	-2.3	-60.1	-57.8	285.8
**4**	234.4	-4.4	-57.2	-52.8	287.3
**5**	231.9	-7.0	-58.3	-51.3	283.2
**6**	234.1	-10.7	-56.2	-45.5	279.6
**7**	236.7	-13.8	-54.3	-40.5	277.2
**8**	246.6	-9.9	-54.3	-44.5	291.1
**10**	257.4	-7.9	-52.1	-44.2	301.6
**11**	209.6	-2.5	-64.7	-62.1	271.7
**12**	226.5	-4.7	-59.0	-54.3	280.8
**13**	238.5	-6.2	-55.5	-49.3	287.8
**14**	246.5	-6.0	-52.4	-46.4	292.9
**15**	222.2	-8.6	-67.7	-59.1	281.3
**16**	224.1	-10.1	-58.0	-48.0	272.1
**17**	230.2	-13.5	-59.7	-46.2	276.4
**18**	229.2	-15.5	-61.3	-45.8	275.0
**19**	242.5	-14.7	-52.0	-37.3	279.8
**20**	246.1	-17.1	-52.5	-35.3	281.4
**21**	235.1	-14.1	-54.2	-40.1	275.2
**22**	233.2	-14.2	-53.8	-39.5	272.7
**23**	231.4	-14.4	-53.1	-38.7	270.1
**24**	235.4	-14.6	-53.2	-38.7	274.0
**25**	231.3	-14.0	-53.1	-39.1	270.4
**26**	228.6	-14.0	-51.1	-37.1	265.7
**27**	233.4	-17.3	-49.2	-31.8	265.2
**28**	241.5	-21.5	-56.4	-34.9	276.3
**29**	231.8	-5.1	-59.4	-54.3	286.1
**30**	238.4	-20.9	-62.0	-41.1	279.5
**31**	237.6	-21.9	-63.0	-41.2	278.8
**32**	237.8	-22.3	-64.9	-42.6	280.4
**33**	245.2	-25.5	-57.3	-31.8	277.0
**34**	246.3	-30.2	-60.9	-30.7	277.0
**35**	293.9	-18.8	-42.6	-23.8	317.8
**36**	306.4	-32.2	-50.3	-18.1	324.5
**37**	279.5	-22.1	-50.7	-28.6	308.2
**38**	279.3	-23.6	-49.4	-25.8	305.0
**39**	292.1	-32.2	-52.0	-19.8	311.9

Table S7. Experimental and calculated ^{ACN}pK_a(**B**H$^+$) values for a set of phosphorus bases.[a,b]

Base **B**	pK_a(**B**H$^+$) exp.	M1 IPCM	M1 CPCM	M1 SMD1	M2 SMD2	M3 SMD2	M5 SMD2	M5 IPCM
**3**	15.5	14.5	15.1	16.3	15.2	15.9	16.5	15.3
**4**	16.6	19.5	15.4	17.0	16.3	16.3	16.3	14.5
**5**	12.7	12.4	12.6	13.8	13.0	13.2	13.9	12.6
**6**	10.0	10.0	10.0	10.3	10.1	10.2	10.4	13.0
**7**	7.6	8.6	8.1	8.1	8.2	7.5	7.2	7.5
**8**	16.1	16.4	17.8	19.8	19.4	19.3	18.3	15.8
**10**	32.9	27.5	28.6	30.6	28.0	29.7	35.0	32.4
**11**	5.2	3.3	3.3	4.4	3.7	6.1	7.3	5.7
**12**	10.6	11.6	10.3	11.6	11.0	12.2	12.5	10.0
**13**	15.7	17.0	14.4	15.9	16.7	16.9	16.1	13.3
**14**	17.0	18.1	18.8	21.2	20.9	20.7	19.4	17.1
**15**	13.5	12.6	10.0	11.9	11.5	12.1	12.7	10.9
**16**	4.2	4.6	4.3	4.5	4.0	4.5	5.2	4.7
**17**	6.6	7.3	6.1	6.8	7.5	6.6	6.6	6.2
**18**	5.4	5.5	6.0	6.7	6.3	5.6	5.7	4.6
**19**	9.1	13.1	9.8	9.6	10.3	9.5	8.6	8.9
**20**	10.1	12.9	12.0	12.3	11.5	10.4	9.4	9.9
**21**	6.1	9.1	6.5	6.4	6.5	6.0	5.8	6.3
**22**	4.6	7.0	4.7	4.5	4.5	4.5	4.7	5.3
**23**	3.0	4.8	3.0	2.3	2.4	2.5	2.7	3.8
**24**	5.2	6.6	6.2	5.2	5.6	5.3	5.1	6.1
**25**	2.5	4.5	3.4	2.6	2.6	2.5	2.8	4.0
**26**	0.7	1.4	0.6	-1.6	-1.2	-1.9	-1.5	0.4
**27**	1.7	3.4	2.3	-1.8	-1.6	-0.7	-1.4	2.9
**28**	6.6	10.4	7.5	7.0	7.4	7.1	6.1	6.8
**29**	15.6	13.3	14.5	16.0	15.4	15.9	16.1	13.8
**30**	9.4	9.5	9.9	10.1	10.0	9.6	9.4	8.7
**31**	9.8	9.4	10.2	10.6	9.4	9.1	9.4	9.0
**32**	10.2	8.4	9.6	9.9	10.7	11.0	10.8	9.7
**33**	8.0	8.3	7.3	5.5	8.0	6.9	6.7	8.8
**34**	7.8	9.0	8.4	6.8	8.0	7.6	7.5	9.3
**35**	43.4	43.9	42.8	40.5	41.1	42.8	41.7	43.6
**36**	45.4	47.6	46.7	44.6	46.6	43.9	41.8	44.3
**37**	32.7	34.2	34.5	34.2	33.3	32.7	31.0	31.7
**38**	29.5	31.3	31.8	30.4	30.8	30.0	31.1	33.1
**39**	38.8	37.4	36.9	35.8	36.3	38.2	38.8	39.6
a		0.687	0.754	0.810	0.813	0.751	0.693	0.623
b	–	-175.3	-194.0	-214.6	-217.2	-195.5	-179.7	-155.3
R^2		0.976	0.986	0.980	0.980	0.987	0.986	0.988
MUE	–	1.6	1.0	1.2	1.1	0.9	1.1	0.9
RMS		2.0	1.4	2.7	1.6	1.3	1.4	1.3

a) Computational models are as given in S1 section.

b) For the references on the experimental ^{ACN}pK_as see Table 2 in the main text.

S7. C-Bases

Table S8. Electronic energies (E_{el}), Gibbs corrections (G_{corr}) and total Gibbs energies (G_{tot}) calculated for a set of the carbon bases. All energies are given in atomic units (a.u.).

M6

Base B	E_{el}(**B**)	G_{corr} (**B**)	G_{tot}(**B**)	E_{el}(**BH**$^+$)	G_{corr} (**BH**$^+$)	G_{tot}(**BH**$^+$)
**Y1**	-1073.42172	0.25271	-1073.16901	-1073.86958	0.26642	-1073.60315
**Y2**	-1416.37987	0.34168	-1416.03819	-1416.84240	0.35638	-1416.48602
**Y3**	-1759.33330	0.42896	-1758.90434	-1759.81106	0.44400	-1759.36706
**Y4**	-1759.34844	0.42845	-1758.92000	-1759.80365	0.44105	-1759.36261
**Y5**	-1416.38587	0.34063	-1416.04524	-1416.83836	0.35330	-1416.48506
**Y6**	-1416.38312	0.34058	-1416.04255	-1416.84250	0.35491	-1416.48758
**Y7**	-1426.36542	0.48871	-1425.87671	-1426.83499	0.50545	-1426.32954
**Y8**	-1265.93974	0.32344	-1265.61630	-1266.39261	0.33840	-1266.05421
**Y9**	-1080.57821	0.39282	-1080.18538	-1081.02930	0.40671	-1080.62260
**Y10**	-1112.63251	0.27911	-1112.35340	-1113.08471	0.29464	-1112.79007
**Y11**	-1116.20954	0.34960	-1115.85994	-1116.66570	0.36412	-1116.30157
**Y12**	-1119.78722	0.41926	-1119.36796	-1120.24232	0.43380	-1119.80852
**Y13**	-1013.41976	0.31226	-1013.10750	-1013.85506	0.32411	-1013.53095
**Y14**	-1240.37792	0.39869	-1239.97923	-1240.82723	0.41243	-1240.41480
**Y15**	-1245.15995	0.41711	-1244.74284	-1245.60422	0.43157	-1245.17265
**Y16**	-1394.86953	0.46902	-1394.40051	-1395.32611	0.48414	-1394.84196
**Y17**	-1191.29558	0.35160	-1190.94398	-1191.75425	0.36606	-1191.38820
**Y18**	-1530.68136	0.36948	-1530.31188	-1531.14693	0.38405	-1530.76287
**Y19**	-1230.28490	0.36072	-1229.92419	-1230.73389	0.37550	-1230.35839
**Y20**	-2537.06423	0.59342	-2536.47081	-2537.52618	0.61030	-2536.91587
**Y21**	-1217.64281	0.31148	-1217.33133	-1218.05955	0.32374	-1217.73581
**Y22**	-1449.38384	0.41575	-1448.96809	-1449.81051	0.42835	-1449.38216
**Y23**	-1151.84764	0.30697	-1151.54068	-1152.30041	0.32207	-1151.97834
**Y24**	-1494.80605	0.39604	-1494.41002	-1495.27575	0.41258	-1494.86317
**Y25**	-1494.80970	0.39519	-1494.41451	-1495.27436	0.41094	-1494.86342
**Y26**	-1837.77463	0.48286	-1837.29177	-1838.23627	0.49819	-1837.73808
**Y27**	-861.29926	0.28976	-861.00950	-861.75177	0.30498	-861.44679
**Y28**	-2029.09360	0.53520	-2028.55840	-2029.56533	0.55183	-2029.01349
**Y29**	-2372.05465	0.62329	-2371.43135	-2372.53268	0.64056	-2371.89212
**Y30**	-1183.92805	0.37327	-1183.55478	-1184.37553	0.38918	-1183.98635
**Y31**	-1948.43513	0.59395	-1947.84118	-1948.89180	0.60955	-1948.28225

Table S9. Reduced GBs, solvation energies (ΔG_s), the reduced Gibbs energy of dissociation in solution ($\Delta G'_{a,sol}$(**B**H^+)) and $^{ACN}pK_a$ values calculated for a set of the carbon bases. All energies are given in kcal mol^{-1} (1 kcal = 4.184 kJ)

Base	$^{ACN}pK_a$ (exp)[a]	GB' (M6)	$\Delta G°_s$(**B**)	$\Delta G°_s$(**B**H^+)	$\Delta(\Delta G°_s)$	$\Delta G'_{a,sol}$(**B**H^+) (M6)	$^{ACN}pK_a$ (calc)[b]
IPCM//M6							
**Y1**	**34.4**	272.4	-4.1	-34.8	-30.67	303.1	35.4
**Y2**	**38.6**	281.0	-6.8	-32.5	-25.60	306.6	37.8
**Y3**	**42.1**	290.4	-9.9	-31.3	-21.47	311.8	41.5
**Y4**	**35.3**	277.7	-8.5	-32.5	-23.97	301.7	34.4
**Y5**	**34.9**	276.0	-6.0	-33.0	-27.06	303.0	35.3
**Y6**	**36.5**	279.3	-7.1	-33.6	-26.48	305.7	37.2
**Y7**	**33.3**	284.2	-3.1	-29.8	-26.64	310.8	(40.8)
**Y8**	**35.8**	274.8	-3.7	-32.7	-29.01	303.8	35.9
**Y9**	**38.0**	274.4	-2.9	-34.7	-31.78	306.1	37.5
**Y10**	**35.9**	274.0	-3.4	-34.0	-30.55	304.6	36.4
**Y11**	**38.4**	277.1	-2.8	-34.0	-31.20	308.3	39.0
**Y12**	**39.0**	276.5	-2.1	-33.9	-31.77	308.2	39.0
**Y13**	**31.2**	265.7	-4.5	-36.8	-32.23	297.9	31.7
**Y14**	**34.9**	273.3	-4.7	-34.0	-29.25	302.6	35.0
**Y15**	**32.4**	269.7	-5.2	-33.9	-28.67	298.4	32.0
**Y16**	**35.6**	277.0	-5.2	-32.4	-27.21	304.2	36.2
**Y17**	**38.3**	278.8	-3.9	-33.4	-29.53	308.3	39.0
**Y18**	**40.0**	283.0	-7.7	-32.9	-25.22	308.2	39.0
**Y19**	**34.5**	272.5	-2.9	-33.0	-30.05	302.5	35.0
**Y20**	**30.7**	279.3	-20.4	-37.6	-17.17	296.5	30.7
**Y21**	**24.5**	253.8	-11.8	-45.2	-33.41	287.2	24.2
**Y22**	**25.5**	259.8	-12.8	-41.5	-28.71	288.5	25.1
**Y23**	**36.9**	274.6	-2.9	-33.3	-30.32	305.0	36.7
**Y24**	**40.9**	284.4	-5.5	-31.7	-26.25	310.6	40.7
**Y25**	**39.9**	281.7	-6.1	-32.8	-26.78	308.5	39.2
**Y26**	**38.9**	280.1	-7.9	-31.6	-23.76	303.8	(35.9)
**Y27**	**37.7**	274.4	-1.0	-37.2	-36.21	310.6	(40.7)
**Y28**	**40.0**	285.6	-9.2	-30.6	-21.44	307.0	(38.1)
**Y29**	**41.6**	289.1	-12.3	-31.1	-18.78	307.9	(38.8)
**Y30**	**33.1**	270.8	-5.4	-35.5	-30.07	300.9	33.8
**Y31**	**33.3**	276.8	-8.1	-31.4	-23.32	300.1	33.2
a							0.705
b							-178.3
R^2							0.982
MUE							1.0
RMS							1.7

a) For the references on the experimental $^{ACN}pK_a$s see Table ?? in the main text.
b) values in parentheses were omitted from the regression

S8. Cartesian coordinates

a) Phosphorous bases and their conjugated acids

1

P	-0.2087012251	0.3672152106	-0.1510847884
H	-0.0918354177	0.0949587979	1.2435686253
C	1.5871464119	0.0760736783	-0.6077258046
H	-0.6901654295	-0.9413788742	-0.4487642411
H	1.6736933364	0.0413207753	-1.6982533781
H	2.1833808458	0.9241251751	-0.2566393745
H	2.0031034491	-0.8447238357	-0.1909179845

1H$^+$

P	-0.0691455676	0.2479572731	-0.0700267884
H	-0.1758571646	0.2506204365	1.3265059948
C	1.650656921	0.0863294064	-0.6237513998
H	-0.8732587381	-0.8012344648	-0.5329214276
H	1.6802831446	0.0887434556	-1.717522887
H	2.2354843225	0.926876225	-0.2381592535
H	2.0653631885	-0.8534977259	-0.2469134554
H	-0.6593231093	1.4356413888	-0.5204777912

2

P	-0.1813731444	0.3721777258	-0.1282497408
C	-0.0618414021	0.0726828364	1.7146837207
C	1.5960051916	0.0726827372	-0.6298649044
H	-0.6777555556	-0.9188342931	-0.4792451319
H	1.6588764035	0.0110684625	-1.7212766634
H	2.2064994022	0.9246981214	-0.3120450953
H	2.0191791731	-0.8411702734	-0.1994514199
H	-1.0698772956	0.0110686258	2.1377629833
H	0.4850142274	-0.8411701816	1.9701849793
H	0.4412999949	0.924698227	2.1843233015

2H$^+$

P	-0.0678209007	0.1412920797	-0.0026003468
C	-0.1188515948	0.0779708316	1.8099014764
C	1.60681246	0.1148089271	-0.7001938919
H	-0.8048067461	-0.9303987127	-0.5249228704
H	1.5487889546	0.1556393533	-1.7922387853
H	2.1709800216	0.9785849618	-0.3355217099
H	2.1165353472	-0.8054473768	-0.399149405
H	-1.1596916314	0.0980932532	2.1473093367
H	0.3570471432	-0.8431795583	2.1596623408
H	0.4121091719	0.9408006418	2.2233895643
H	-0.7362792255	1.2908775992	-0.4453627089

3

P	-0.1917944635	0.3321977554	-0.1356187943
C	-0.0480678794	0.0832560094	1.7096181569
C	1.5958204793	0.0832560094	-0.615191055
C	-0.8700120588	-1.3403930703	-0.615191055
H	1.6816831235	0.0487179927	-1.7067411375
H	2.1919045929	0.9324475009	-0.2636168937

H	2.021010648	-0.8394169193	-0.2004990121
H	-1.0485702851	0.0487179927	2.1544202616
H	0.4846375843	-0.8394169193	1.9722606114
H	0.4820941232	0.9324475009	2.1544202616
H	-0.8830325811	-1.4320213097	-1.7067411375
H	-0.2835489475	-2.1699550222	-0.2004990121
H	-1.9034755199	-1.4320213097	-0.2636168937

3H+

P	-0.0105008425	0.0181880418	-0.0074246279
C	-0.0419127302	0.0725950273	1.8069992418
C	1.689683758	0.0725950272	-0.6418479962
C	-0.9077109743	-1.4270115205	-0.6418479962
H	1.6784169027	0.0832793949	-1.7360028733
H	2.1929999562	0.9745633882	-0.2805422071
H	2.2412169963	-0.8079972487	-0.2975940058
H	-1.0772474597	0.0832793951	2.1610950412
H	0.4664974573	-0.8079972486	2.2122384356
H	0.4665017007	0.9745633883	2.1610950412
H	-0.9113304805	-1.4119119538	-1.7360028733
H	-0.4208623122	-2.3449494539	-0.2975940058
H	-1.9404965874	-1.4119119537	-0.2805422071
H	-0.6717826063	1.163561655	-0.4750214474

4

P	-0.1901806964	0.3307130014	-0.1369767218
C	0.0348368521	0.1285241262	1.717872945
C	1.5792197986	0.0316978388	-0.6951288443
C	-0.9293508659	-1.3486748789	-0.5427342242
C	1.7590257639	0.1047018203	-2.2172821577
H	2.1966558206	0.8045753665	-0.2201183125
H	1.9342717279	-0.9358176689	-0.3124650888
H	-0.9616926808	-0.0508746664	2.1407856053
H	0.6331907962	-0.7698093387	1.9266490015
C	0.6584109769	1.3571770219	2.3929915211
H	-0.8159419784	-1.4914102652	-1.6248000433
H	-0.346114216	-2.1435272949	-0.0559825549
C	-2.412882429	-1.4673934421	-0.1687583658
H	-2.8150088096	-2.443250075	-0.4661055892
H	-2.5687550395	-1.3656992069	0.9114067218
H	-3.0097656905	-0.6931338828	-0.6636064657
H	0.7285170707	1.2149400055	3.4780316788
H	1.6715993794	1.5531392689	2.0236447359
H	0.0598322941	2.2565002673	2.2099333969
H	2.8117416719	-0.0276782125	-2.4943286842
H	1.1860372427	-0.6750050652	-2.7322171397
H	1.427792011	1.0725142806	-2.6100984143

4H+

P	0.0094251834	-0.0161839944	0.0069139304
C	0.0056398343	0.0736399158	1.8355268218
C	1.7075382308	0.0343089843	-0.6755559166
C	-0.9302940557	-1.4618110399	-0.6082109714
C	1.7627525007	0.1006182367	-2.2118332238
H	2.1982286008	0.9075988262	-0.2304305911
H	2.2304024415	-0.8527433439	-0.2965260602

H	-1.0418762302	0.0010172583	2.1502659483
H	0.5113436168	-0.8291051869	2.2003848681
C	0.6585426934	1.3470943511	2.4002754478
H	-0.8428475329	-1.4498142527	-1.7007780935
H	-0.4043156584	-2.3579626	-0.2560191121
C	-2.4073160156	-1.4743631586	-0.1773919851
H	-0.6541976872	1.1318097598	-0.4603587309
H	-2.898817297	-2.3437567624	-0.6226977312
H	-2.5198400669	-1.553603872	0.9077270608
H	-2.9392908694	-0.5814489456	-0.5207041949
H	0.5687798242	1.3371786591	3.4900466082
H	1.72374464	1.4060822224	2.1594448662
H	0.1656599511	2.2540225051	2.0359490982
H	2.808089375	0.1694190846	-2.5253360316
H	1.3382262347	-0.7926059212	-2.6790898324
H	1.2421122867	0.9812972741	-2.6011321749

5

P	-0.4497047038	0.1122140169	0.0026740401
C	-0.2407061682	0.0213799516	1.8462929122
C	1.2501201128	0.6862155984	-0.5217809028
C	-0.3138902866	-1.6934096186	-0.4611465311
H	1.3088390335	0.6650601688	-1.6158182024
H	1.3998758207	1.7210180054	-0.1977704518
H	2.0626107211	0.0717014569	-0.1170296998
H	-0.2957308145	-1.7779802884	-1.5535310457
H	0.5813542387	-2.1813212006	-0.0585994923
H	-1.1968670207	-2.2288105266	-0.0979238508
C	-1.1943389	0.6682407927	2.6459308228
C	-1.1016936638	0.642220543	4.0398508989
C	-0.0491914544	-0.0341132244	4.6560245532
C	0.9094340479	-0.6837039903	3.8736300935
C	0.8125810559	-0.654881131	2.4828024304
H	-2.0163638697	1.1965704813	2.1689537689
H	-1.8511178202	1.149744755	4.6418536641
H	0.0260226821	-0.0564787627	5.740137374
H	1.7321701771	-1.2125140625	4.3485648303
H	1.5681520087	-1.1662167496	1.8909695414

5H$^+$

P	-0.0985241196	-0.1272293725	0.1314754123
C	-0.045494684	-0.0630640059	1.9205949561
C	1.3560115936	0.6360347725	-0.6513017595
C	-0.3373907552	-1.8118942671	-0.5133775737
H	1.2473303564	0.61838484	-1.7401557442
H	1.4523870253	1.670731112	-0.3103203679
H	2.2592444629	0.0848227487	-0.3726004971
H	-0.4168425769	-1.7870262684	-1.6046136592
H	0.5118518799	-2.4412204091	-0.2304061454
H	-1.2513729822	-2.2379392949	-0.0899685708
C	-1.0321799069	0.6580370567	2.6147884343
C	-0.9916715794	0.7084461778	4.0073566202
C	0.0224451139	0.0465725043	4.7019528982
C	1.0043084172	-0.6710051997	4.0110896999
C	0.9766091738	-0.7301339393	2.6207604684
H	-1.8244715983	1.175634518	2.0808810304

H	-1.7519154024	1.2643255547	4.5467499722
H	0.0496806718	0.0888151255	5.7864802017
H	1.7906006102	-1.1838277482	4.5558189719
H	1.7455711843	-1.2912171267	2.0953031932
H	-1.2173282678	0.6250267174	-0.2535152526

6

C	0.1123128968	0.3041421819	-0.0293985235
C	-0.0243895512	-0.0100353617	1.3310249432
C	1.139434913	-0.3087544993	2.0630277579
C	2.3912820179	-0.3138747354	1.4523318254
C	2.5115733156	-0.0010019272	0.095456267
C	1.3696197603	0.3110200799	-0.6404824565
P	-1.627633702	0.0534450038	2.2549637925
C	-1.8883082753	-1.7349595794	2.6828902736
C	-1.5046786344	-2.7996827184	1.8517334048
C	-1.7622103941	-4.11937516	2.221866833
C	-2.4101296072	-4.396665113	3.4286683518
C	-2.7940621983	-3.3485422551	4.2652612413
C	-2.5290514516	-2.0276808745	3.8958840873
C	-2.8742965599	0.2368914385	0.8774710169
H	-0.9925678982	-2.594656705	0.9153148839
H	-1.4567887933	-4.9332256606	1.5688786002
H	-2.6091398545	-5.4257799831	3.7163375285
H	-3.2926078879	-3.5571269135	5.2084231633
H	-2.8180547463	-1.2141911923	4.5573709575
H	1.0615747435	-0.5435744698	3.1223369807
H	3.2754402315	-0.5556514518	2.0366708075
H	3.4883383152	0.0028876241	-0.3809102304
H	1.4518246394	0.557960984	-1.6960785286
H	-0.7608868032	0.5462207998	-0.6274106689
H	-3.8713014629	0.122961658	1.314377386
H	-2.8109155304	1.2419131853	0.4472959758
H	-2.7570804822	-0.5062743553	0.0812623305

6H^+

C	0.2246829808	0.7018185661	0.0252834551
C	0.012950276	-0.0832000701	1.171887636
C	1.0992362087	-0.6978296633	1.8208686426
C	2.3870967462	-0.5228372995	1.3203945278
C	2.5961310088	0.256626546	0.1795754866
C	1.5183045767	0.8661425259	-0.4663659138
P	-1.6369003539	-0.2690399632	1.8663226877
C	-1.8998918245	-1.8797043073	2.6143254952
C	-1.653128071	-3.0497847123	1.8735423998
C	-1.9104037261	-4.2910386239	2.4494083199
C	-2.4078660502	-4.3695709323	3.7540674985
C	-2.6489450777	-3.2085029942	4.4910560136
C	-2.397330268	-1.958910816	3.9261117676
C	-2.9232836874	0.1248437209	0.6379583598
H	-1.2547164059	-2.9965669524	0.8638662904
H	-1.7207247265	-5.1970086805	1.882445898
H	-2.6045168588	-5.3407571469	4.197971106
H	-3.0293511527	-3.2731107227	5.5055272081
H	-2.5837824105	-1.0591094469	4.5063179185
H	0.9422202307	-1.3128380482	2.7019634342

H	3.2261159204	-0.9976371497	1.8191671455
H	3.6018310049	0.3876769968	-0.2081051649
H	1.6823636443	1.4686410888	-1.3541520896
H	-0.6022398167	1.1820226056	-0.4881487838
H	-3.9028516681	-0.0131668437	1.1046327671
H	-2.8332334979	1.160997198	0.3000530985
H	-2.8377372587	-0.5484114927	-0.2195456525
H	-1.828124743	0.6485816177	2.9136284483

7

P	-0.1172976661	0.2030785321	-0.0803070146
C	-0.0299231542	0.0189110665	1.7626463003
C	1.6581315874	0.0377868546	-0.5892462511
C	-0.8426892973	-1.4175618616	-0.6148332265
C	2.2794032042	-1.1716846095	-0.9365731589
C	3.6188172638	-1.1954019082	-1.3314618583
C	4.3591379063	-0.0132068522	-1.3813058487
C	3.7520407682	1.19737531	-1.0405723374
C	2.4112969537	1.2221525186	-0.6572835231
H	1.7146133026	-2.0985272018	-0.902642636
H	4.0838419032	-2.1407816907	-1.5997904408
H	5.4010457076	-0.0339213085	-1.6900270802
H	4.3191643415	2.1236858368	-1.0835438878
H	1.9401159238	2.1714484914	-0.4124739663
C	-1.3684847977	-1.4792593004	-1.9164899478
C	-1.94352527	-2.6537137269	-2.4013098869
C	-2.0198726634	-3.7836005232	-1.5841067838
C	-1.5138204522	-3.7318248756	-0.2843854623
C	-0.9274289731	-2.5590872199	0.1967042138
H	-1.3301115509	-0.5973998348	-2.5520827256
H	-2.3414651198	-2.6834015858	-3.4123984693
H	-2.4769117944	-4.6967832344	-1.956290954
H	-1.5739407008	-4.6063708835	0.3586661314
H	-0.5374167972	-2.5310457962	1.2096942915
C	-1.1469737406	0.4531540985	2.4963097991
C	-1.1688128188	0.3633574439	3.8879305995
C	-0.0635411479	-0.1462312024	4.5726211088
C	1.058261911	-0.567932287	3.8571199778
C	1.0752596341	-0.4882783599	2.4629168075
H	-2.0037009899	0.8695928788	1.9712282639
H	-2.0435316178	0.7011019841	4.4377709705
H	-0.0741598869	-0.2078890305	5.6576961503
H	1.9240602524	-0.961149007	4.3841170344
H	1.9544527791	-0.8186047153	1.9180338104

7H^{+}

P	0.078373275	-0.1651060306	0.084057467
C	0.0606959636	-0.1106300761	1.8841235754
C	1.7391813542	-0.0421761466	-0.5995929146
C	-0.8177783937	-1.5858769434	-0.5717909567
C	2.4305704831	-1.1951666387	-1.0055608491
C	3.7343852313	-1.0785845775	-1.4864386708
C	4.3417899261	0.1763179093	-1.5674803573
C	3.6510629026	1.3243264439	-1.1684119312
C	2.3500670609	1.2214881749	-0.682436869
H	1.9551046363	-2.1702615576	-0.9589437578

H	4.2716880009	-1.9669404762	-1.8034434574
H	5.3551103899	0.2619752846	-1.9481195295
H	4.1233091727	2.2990459138	-1.2403406505
H	1.8178706509	2.1182761733	-0.3754658403
C	-1.0601170842	-1.6563523303	-1.9560134347
C	-1.7550492292	-2.7447808515	-2.4754015628
C	-2.2044106853	-3.7599534114	-1.6244712108
C	-1.962376158	-3.6899854456	-0.2517700142
C	-1.2708264867	-2.6025174052	0.2826693121
H	-0.7100688956	-0.8730339453	-2.6234302867
H	-1.9479217045	-2.80090571	-3.5421716686
H	-2.7465221708	-4.6064772668	-2.0350719183
H	-2.3136268271	-4.4787376979	0.4060918043
H	-1.0905154775	-2.5462302552	1.3513965524
C	-1.0756280907	0.3930455052	2.5414981415
C	-1.1088380636	0.4156117791	3.9337936252
C	-0.0177645691	-0.0592315529	4.6673861064
C	1.1119794726	-0.5562758119	4.0136237291
C	1.1591026807	-0.5823725482	2.6204963495
H	-1.9254212117	0.7680955594	1.9766958791
H	-1.9818566425	0.8089484048	4.4450805699
H	-0.0467007098	-0.0356345155	5.7525829787
H	1.9601483173	-0.9170477882	4.5870002313
H	2.0446233291	-0.9552673296	2.1145521764
H	-0.5987787342	0.99343096	-0.3343592846

8

P	0.2399745729	-0.2873450665	0.3686923404
C	0.0995366973	0.0441954807	2.2283350464
C	2.1018185431	-0.1396809973	0.0433602942
C	-0.0768524486	-2.1597259784	0.1908673849
C	-1.3894904274	0.0246326082	2.6434581347
H	0.6258657341	-0.7369899103	2.7946395426
C	-1.5823905436	0.354161159	4.1330753059
H	-1.9367821035	0.7599738845	2.0359864542
H	-1.8384789962	-0.9508599753	2.4212733758
C	-0.9501536067	1.7046815743	4.4951960147
H	-2.652570967	0.3552462139	4.3780597418
H	-1.1234340591	-0.4378682924	4.7435422971
C	0.5287580527	1.745191293	4.0886584991
H	-1.4911782671	2.5081228992	3.9735662804
H	-1.0563926504	1.9022398231	5.5696691989
C	0.7252732588	1.406002001	2.6006326033
H	0.9571207866	2.7331915547	4.3025151665
H	1.0913993894	1.0232613147	4.6992371976
H	0.2682153517	2.1951778717	1.9848581586
H	1.7969767007	1.4132932427	2.3714625742
C	2.3869526061	-0.2881761954	-1.4678317667
H	2.3138063257	0.9106014101	0.2958820577
C	3.8571060564	0.0191224562	-1.801407689
H	2.1583040991	-1.315365125	-1.7890990979
H	1.7231878256	0.3711080404	-2.0405136886
C	4.8217603024	-0.841151196	-0.9719552475
H	4.0372489285	-0.1327212217	-2.8736768119
H	4.0553766261	1.0822216534	-1.599400322
C	4.5383891907	-0.7043305918	0.5306856221

H	4.7094891483	-1.8957352047	-1.265542699
H	5.8615903469	-0.5661936938	-1.1909416944
C	3.0677227581	-1.020705299	0.8589885321
H	5.2012337675	-1.3643258762	1.1057159836
H	4.7667566137	0.323133381	0.8511714514
H	2.8763759133	-2.0785361772	0.6312974266
H	2.8944219467	-0.8950992687	1.9350369238
C	-1.4892789951	-2.3981758639	-0.3919935461
H	0.6410071757	-2.4693117299	-0.5848823172
C	-1.7336150805	-3.8798909496	-0.7211200834
H	-2.2458073187	-2.0595441051	0.3312738675
H	-1.6298612832	-1.7829849096	-1.2886759995
C	-1.4846262749	-4.7813919819	0.4963874955
H	-2.7565298524	-4.0172884208	-1.0954442532
H	-1.060160151	-4.182000155	-1.5374182072
C	-0.0832766966	-4.5502413907	1.0785927944
H	-2.2366760137	-4.5624161276	1.2692391726
H	-1.6154833511	-5.8367856944	0.2244266943
C	0.1544626438	-3.0670717573	1.4176725848
H	0.0631152333	-5.1651324314	1.976552751
H	0.6715392553	-4.8801208618	0.3490096918
H	-0.5325517498	-2.7794458989	2.2253346947
H	1.1679739866	-2.9379425137	1.8127170685

8H^+

P	-0.0093921822	-0.1325566781	-0.6174865709
C	1.5384765801	-0.8461721807	0.0993490787
C	-1.497556321	-1.1829030317	-0.3093666738
C	-0.3109474413	1.6418573195	-0.1675560641
C	2.7906567496	-0.1280206686	-0.4647506929
H	1.4773622445	-0.6581619939	1.1803792535
C	4.0784945179	-0.7244730002	0.1308556126
H	2.8098874665	-0.243129286	-1.5577539365
H	2.7498709561	0.9466862638	-0.2566377043
C	4.1621667352	-2.2388618123	-0.0972790349
H	4.9421528052	-0.2180197415	-0.3142305949
H	4.1105628072	-0.5097063539	1.2081906867
C	2.9195178927	-2.9495201007	0.4534006088
H	4.256221699	-2.4434241322	-1.1732203878
H	5.0644656704	-2.6407790946	0.3761985182
C	1.6180408531	-2.3746445849	-0.1352622739
H	2.9609681438	-4.02315424	0.2390672745
H	2.8914545684	-2.8494070917	1.5475516823
H	1.5848960071	-2.5824214577	-1.2146250045
H	0.7587375173	-2.8810890839	0.3178762758
C	-2.6894563039	-0.7463196746	-1.1953462373
H	-1.1879040186	-2.1870912323	-0.631600051
C	-3.8896780496	-1.685427254	-0.97558932
H	-2.988175908	0.2797674856	-0.9408108375
H	-2.4023385166	-0.7447741598	-2.2542812429
C	-4.2861875737	-1.7640828058	0.5051914209
H	-4.7321534106	-1.3350238322	-1.5819714967
H	-3.6339738073	-2.688247402	-1.34507434
C	-3.0974864741	-2.1777577357	1.384025109
H	-4.6619523213	-0.7851758721	0.8356233559
H	-5.1110273516	-2.4731205305	0.6344520404

C	-1.8887430057	-1.2450378083	1.1856256301
H	-3.381404464	-2.1749603762	2.4422082215
H	-2.7997943401	-3.2077184254	1.1426668845
H	-2.1497671244	-0.2378046737	1.5383726856
H	-1.0450885659	-1.5903278482	1.796226759
C	0.2727062843	2.6211667181	-1.2155932735
H	-1.406358488	1.7329743277	-0.1977029381
C	-0.0905618082	4.0710981721	-0.8514237831
H	1.3650875234	2.5178478908	-1.25338897
H	-0.1056912568	2.378992469	-2.216190039
C	0.3542226639	4.4321725799	0.5730650962
H	0.3672025715	4.748008739	-1.5812281767
H	-1.1780084201	4.2026149535	-0.9427433561
C	-0.2120404003	3.4464640074	1.6045103321
H	1.4521943149	4.4263621439	0.6266912608
H	0.0369624218	5.451870795	0.8168698081
C	0.1579164479	1.9912727048	1.2656478868
H	0.1580600953	3.6841294715	2.6079980476
H	-1.3064047834	3.5402636554	1.6443192639
H	1.2469037327	1.8713195272	1.3364555956
H	-0.2878737467	1.3098190149	1.999886819
H	0.175333813	-0.1612560754	-2.0127552075

9

P	0.3451814338	-0.4817459476	-0.0493957068
C	0.0200290029	-0.2823256531	1.7875029585
C	-0.6025348462	1.0943992654	2.0836914621
H	-0.6509877553	-1.07832197	2.1429058921
H	0.9692180599	-0.3942694346	2.3256974377
C	-1.9718484562	1.2927389433	1.4110744557
H	-0.7109551994	1.2178326553	3.1696342759
H	0.0839519911	1.8856503237	1.7503814816
C	-1.9327456584	1.1728977348	-0.1221817887
H	-2.6772642176	0.5483303414	1.8099702751
H	-2.372256499	2.2767730239	1.6875470164
C	-1.4235787466	-0.1971755249	-0.6063016398
H	-2.9390602147	1.3493835898	-0.525227859
H	-1.2926072094	1.9668500032	-0.5324278721
H	-2.0761226897	-0.9942337676	-0.2202071825
H	-1.4593447678	-0.2509765439	-1.7012749818
C	0.4481386674	-2.3412768875	-0.1772950475
H	0.4691498771	-2.6357717097	-1.2323498277
H	1.3804292395	-2.6891361834	0.2811397289
H	-0.3933280113	-2.8477272583	0.3115289218

9H^+

P	0.0732919584	-0.6879099875	0.1051853808
C	-0.0209018409	-0.2847219512	1.8807926005
H	1.059742547	0.1346102449	-0.4630145415
C	-0.6008997727	1.1372683903	2.0778222443
H	-0.6693915546	-1.0388870649	2.3451963149
H	0.9745503402	-0.3883294743	2.3264103413
C	-1.9677353766	1.3460006201	1.4024631377
H	-0.6870854924	1.3061448555	3.156142715
H	0.1172666511	1.8799609382	1.7065369757
C	-1.9321684385	1.2092934121	-0.1298144588

H	-2.6986959281	0.6398477788	1.8202119021
H	-2.3311964415	2.3474461667	1.6543173827
C	-1.5283219935	-0.2032442334	-0.6187311248
H	-2.9204640509	1.427139944	-0.5476045646
H	-1.2470725144	1.9536504901	-0.5561898521
H	-2.2634774761	-0.9526378221	-0.2979074973
H	-1.460359666	-0.2565963953	-1.7108688561
C	0.5384998563	-2.4120376716	-0.2313278755
H	0.5948442668	-2.5778406447	-1.3116352182
H	1.5140649793	-2.6278498887	0.2152254213
H	-0.2088130529	-3.087127707	0.1964365726

10

P	0.2106313973	-0.0511029423	-1.4239965224
N	0.0034422288	1.5274536558	-0.7444318118
N	-1.1818817702	-0.8916790605	-0.8298976378
N	1.4803197055	-0.7054539691	-0.445877208
C	-2.3772724508	-0.8795316559	-1.6575181358
C	2.1781493077	-1.8788147984	-0.9461937395
C	0.7743937306	2.6151211683	-1.3250857452
H	1.2525669834	2.2679611738	-2.2486379537
H	0.1285881868	3.4677268884	-1.582449376
H	1.5719645053	2.9873915237	-0.6615830928
H	-2.1317101397	-0.4657086246	-2.6427234996
H	-2.7662715543	-1.8972203903	-1.8095060535
H	-3.1925904507	-0.268284724	-1.2373334065
H	1.8904089699	-2.0546195755	-1.9895060646
H	3.268349316	-1.7341971657	-0.9193622374
H	1.948179257	-2.7973880371	-0.3819959849
H	-0.2991150514	-2.2902624172	0.4518879751
H	-2.0506141779	-2.3868293647	0.3266715856
H	2.9662979099	-0.4848853644	0.99260233
H	2.0315935262	0.9712129511	0.6754142591
H	-1.9116870059	1.3648989402	0.0805483664
H	-1.1461382326	2.9379550382	0.2627951221
H	0.3400212936	2.0420960231	2.0397673573
H	-1.3695572487	1.8198239696	2.406053812
H	1.5923764421	0.1060600033	2.9045532094
H	1.0706796792	-1.4550715379	2.2747374417
H	-2.2967816326	-0.3638539193	1.7439983363
H	-1.3680577813	-1.636572048	2.5334802515
N	-0.2890289696	0.0736962103	1.9768392515
C	-1.3364461604	-0.893363371	1.716493005
C	-0.5536587832	1.4791999143	1.7433715029
C	-0.9556481636	1.8562143229	0.3018517283
C	1.9488075927	-0.1158948945	0.8012454846
C	-1.2106394883	-1.6812426075	0.3947551862
C	1.0849230299	-0.3810753146	2.0524032641

10H^+

C	-2.0443350552	-1.555123585	-1.7095030553
N	-1.4624726794	-0.8507814987	-0.5612413696
P	-0.0190328032	0.0272350009	-0.7145469083
H	-0.0584940406	0.0814073712	-2.1189529198
C	-1.8953441576	-1.3544622196	0.7353582479
C	-1.3409023398	-0.4126611203	1.7984698272

N	0.039015447	-0.0532266073	1.3773168837
C	1.0419787056	-1.1011336223	1.7040214403
C	2.1269973556	-1.0219486344	0.635697831
N	1.4648959125	-0.7914337403	-0.6411905027
C	2.358496146	-0.8397587265	-1.8040205488
C	0.4481994318	1.3077952839	1.8147724145
C	-0.1675569624	2.2926942081	0.8268339494
N	-0.0473690843	1.7103925465	-0.5031599074
C	-0.4645190329	2.6000230269	-1.5928692343
H	0.3745140824	3.2443134308	0.8677427487
H	-1.2186094884	2.512081231	1.0734429651
H	0.1337469463	1.4896085389	2.8476541294
H	1.5378180444	1.3624750339	1.7679922393
H	-1.52255315	2.8813662322	-1.5032872104
H	0.1431961815	3.5109513583	-1.5713881408
H	-0.3133369008	2.1201172565	-2.5616424824
H	-1.3338351847	-0.8575146098	2.7989365552
H	-1.9323628934	0.5047336609	1.8296482021
H	-2.990104857	-1.3641675355	0.7853782251
H	-1.558026614	-2.3911004303	0.8943944172
H	-1.7744644175	-2.619841899	-1.7105947334
H	-3.1357752304	-1.4707807793	-1.6731611917
H	-1.7055542532	-1.1100659992	-2.6469931142
H	3.1379334021	-0.0678645735	-1.7478189044
H	2.8408462104	-1.8219319064	-1.8510863418
H	1.7959638285	-0.6988809153	-2.7288823706
H	2.6830108882	-1.9658291436	0.6028386238
H	2.8575661626	-0.2273884388	0.8563109954
H	1.4315954297	-0.9581913989	2.7172358672
H	0.5413299706	-2.0704357959	1.6562913731

11

P	0.1596679298	-0.1075162475	-0.0775778314
H	-0.379390527	-0.311561406	1.2256072314
H	-0.7303971322	-1.045611002	-0.677073252
C	-0.8102039458	1.4421911717	-0.5449357521
H	-0.8561037274	1.4415564496	-1.6416398558
H	-1.8441472357	1.3815713899	-0.1836558825
C	-0.1592323886	2.7563131556	-0.0689344548
C	-0.903860402	3.9612461672	-0.6649452632
C	-0.1025016316	2.8566334411	1.4624221261
H	0.8732425749	2.7752354836	-0.447886159
H	-0.4281903299	4.9043271952	-0.3705573328
H	-0.919253179	3.9206537492	-1.7605763781
H	-1.9446422307	3.9911694125	-0.3168495782
H	0.3425212921	3.8083086758	1.7752406793
H	-1.1098106671	2.8015003393	1.896238775
H	0.4971976	2.0528370248	1.9044579281

11H^+

P	-0.0630921266	-0.0678416408	-0.0633403854
H	-0.2086737757	-0.3406854443	1.3020601971
H	1.3159212496	-0.0630259886	-0.3133871165
H	-0.575142729	-1.1878892977	-0.7333068742
C	-0.8607471743	1.4901937999	-0.5924801796
H	-0.8752710364	1.456261811	-1.6890589351

H	-1.901940913	1.4330743749	-0.248725519
C	-0.1625771202	2.7771489429	-0.0767296482
C	-0.9084601296	3.9861331684	-0.664599582
C	-0.1023589238	2.8419374169	1.4557662778
H	0.8618035478	2.7822248252	-0.4738637822
H	-0.4038562216	4.9111417453	-0.3700468753
H	-0.936191308	3.9548564363	-1.7589850416
H	-1.9391712205	4.0346466639	-0.2944886636
H	0.3432208549	3.7892667564	1.772307771
H	-1.1039785217	2.7844889355	1.8988730845
H	0.514975548	2.0469224946	1.8963452724

12

P	0.234767216	0.0239084665	-0.1950460373
H	1.3027896451	0.3944786173	-1.066567471
C	-0.6305162878	-1.1928971734	-1.3412654891
C	-0.8302850384	1.541306436	-0.5270499338
H	-1.0187268604	1.6260115335	-1.6052171378
H	-1.8042540309	1.3606192285	-0.0499859762
C	-0.2446171355	2.867307693	0.0029267584
C	-1.0864203524	4.0535029698	-0.4947164937
C	-0.1335196393	2.8878248374	1.53480628
H	0.7676388134	2.9807322296	-0.4140105796
H	-0.6656724242	5.0068428814	-0.1528773198
H	-1.1350434412	4.0807900193	-1.58984214
H	-2.1148531112	3.989236096	-0.1159243142
H	0.3007645256	3.832797562	1.8818827669
H	-1.125106356	2.7878294446	1.9958478785
H	0.4907366779	2.0702335284	1.9098130374
H	-1.6696077232	-1.2420140377	-0.987489634
H	-0.6666332689	-0.7993877431	-2.3665970142
C	-0.035281398	-2.6155695769	-1.3419104938
C	-0.9350113299	-3.5674963375	-2.1457232511
C	1.4030837393	-2.6443849095	-1.8800933127
H	-0.0132145787	-2.9692965088	-0.3007737269
H	-0.5459775661	-4.5925449245	-2.123270704
H	-1.9556427341	-3.5866028287	-1.7453188412
H	-0.9943434113	-3.257348398	-3.1971932325
H	1.7955014337	-3.6680357457	-1.8917998633
H	1.4442554081	-2.2604097781	-2.9081635597
H	2.0810752283	-2.0383895813	-1.2687251953

12H^+

P	0.0279460563	-0.0102081305	-0.1223235045
H	-0.0284054522	-0.2237425534	1.2620030376
H	1.3915266129	0.172961063	-0.3911009855
C	-0.5937760958	-1.5062453515	-0.9708323418
C	-0.867022947	1.5210770154	-0.5681583227
H	-0.9456183583	1.5122172664	-1.6630195439
H	-1.8860865854	1.4151397233	-0.173004196
C	-0.2152852182	2.8401524721	-0.0756414391
C	-1.0260420528	4.0185401456	-0.6386917752
C	-0.1142184331	2.9130973711	1.4547535896
H	0.797340231	2.8916485387	-0.4995749753
H	-0.5590633784	4.9656337816	-0.3517794054
H	-1.0790879327	3.989900695	-1.7324297218

H	-2.0496586623	4.0182689484	-0.2458129457
H	0.2974916059	3.8801813889	1.7579726597
H	-1.1008123967	2.813255888	1.9236706786
H	0.5453932333	2.1446516308	1.8784055107
H	-1.6868578224	-1.4694581017	-0.8771529413
H	-0.3592509372	-1.3827816696	-2.0364796825
C	-0.0422103978	-2.8533136447	-0.433734048
C	-0.7559306727	-3.9963065504	-1.173428395
C	1.4827889767	-2.9646557214	-0.5737048216
H	-0.3053372653	-2.9217881086	0.6310919325
H	-0.4260825348	-4.961898254	-0.7781934737
H	-1.8434956583	-3.9406544364	-1.055101194
H	-0.5264451437	-3.9781162848	-2.245411302
H	1.8197119625	-3.949136068	-0.2360932244
H	1.7956865003	-2.8496901273	-1.6187370971
H	2.0243107662	-2.224078926	0.0291239277

13

P	-0.2447209909	0.4172262845	-0.1861652765
C	0.0419031604	0.2348111711	1.6654985531
C	1.5054362057	0.0631749851	-0.7806084761
C	-1.0061193819	-1.2712652805	-0.5228671551
C	1.7332307645	0.2432701613	-2.2968234018
H	2.1760568937	0.7434562747	-0.2366084246
H	1.7828594279	-0.9569045483	-0.4769605797
H	-0.9226526878	-0.0368019006	2.1175203172
H	0.7176934261	-0.6157715844	1.8386148827
C	0.5870905553	1.493064445	2.3749322554
H	-0.8350347375	-1.4976359016	-1.5849277099
H	-0.453822586	-2.0306508823	0.0501906212
C	-2.5165270944	-1.3845125998	-0.2251296024
C	-2.9603786584	-2.8557260462	-0.2677558589
H	-2.6959769415	-1.0166410615	0.7967319037
C	-3.3605293113	-0.5355927527	-1.1872280236
H	-4.0281232416	-2.9539147038	-0.037128377
H	-2.795126589	-3.285177498	-1.2646106908
H	-2.4027981296	-3.4646776462	0.4539364629
H	-4.4257268299	-0.6000850255	-0.9351361914
H	-3.0669294381	0.5187047075	-1.1592151172
H	-3.2415821239	-0.8890829262	-2.2201961136
C	1.0401024792	1.1465753046	3.8022973072
H	1.473191606	1.8446631622	1.8245571711
C	-0.439857821	2.6348553304	2.3936499895
H	1.4425638264	2.0286267742	4.3149809377
H	0.1983316304	0.7706915512	4.3985677338
H	1.8181111412	0.373813424	3.8003196849
H	-0.0235325201	3.5285609446	2.873589563
H	-0.7581434883	2.90790779	1.3824105852
H	-1.3348259556	2.3408131116	2.9583959203
C	3.0942440929	-0.3416013298	-2.7066549475
H	0.9563489402	-0.3230649791	-2.8330162088
C	1.6245981237	1.7143578599	-2.7244264207
H	3.2611235783	-0.2367394088	-3.7856245198
H	3.9132850355	0.1778951955	-2.1920310106
H	3.1661499212	-1.4069295234	-2.4566943642
H	1.7499399102	1.8180768619	-3.8087786796

H	0.6552637709	2.1433103362	-2.450760889
H	2.4059010371	2.3157399232	-2.24055285

13H+

P	-0.0964473183	0.1553846908	-0.0600092351
C	-0.1306638112	0.1955123172	1.7710608983
C	1.6243769238	0.2085117082	-0.6851029961
C	-0.9781214266	-1.3170843539	-0.7000447852
C	1.8089469713	0.0958350445	-2.2211222175
H	2.0620676551	1.1465639364	-0.3197793901
H	2.1545025143	-0.6065683095	-0.1746902699
H	-1.187268948	0.2523771759	2.0634375905
H	0.2347171915	-0.7867504235	2.0988200012
C	0.6759508906	1.3286062738	2.4565566204
H	-0.9696362178	-1.2355226484	-1.7947758165
H	-0.3470916607	-2.178384526	-0.4433108255
C	-2.4262757927	-1.536017498	-0.1902568336
H	-0.761669595	1.2947001014	-0.5295511532
C	-2.9448257876	-2.8701694503	-0.750489888
H	-2.39342853	-1.6229735324	0.9043268913
C	-3.3638358229	-0.3783022797	-0.5635055111
H	-3.950083319	-3.0749492807	-0.3693058075
H	-3.0020116887	-2.8430611955	-1.8452726509
H	-2.3008426146	-3.7086859941	-0.463558483
H	-4.3833939698	-0.5969479858	-0.2320334847
H	-3.0761220821	0.5731774507	-0.0985058309
H	-3.3955286502	-0.2283601003	-1.6497185351
C	0.605676849	1.1254157174	3.9787505179
H	1.7273514319	1.2292022537	2.1542796757
C	0.1806934406	2.7278234676	2.0621713665
H	1.2088495348	1.8824147512	4.4895470407
H	-0.4246990037	1.2185166386	4.3421785323
H	0.983842127	0.140705	4.274624238
H	0.7383108819	3.4933868437	2.6100304588
H	0.3114533304	2.9435419897	0.9940544316
H	-0.8810860457	2.8559161475	2.3058795896
C	3.3139025898	0.0387804593	-2.5285918473
H	1.3643615914	-0.8532433681	-2.5502332026
C	1.1321803171	1.2460612249	-2.9810391646
H	3.4760796265	-0.0875158267	-3.6035491715
H	3.8147661317	0.9647879393	-2.221769857
H	3.8009556328	-0.7980406694	-2.0160216676
H	1.3291918807	1.1572556168	-4.0536105356
H	0.041668026	1.2578596914	-2.8585896154
H	1.5200067464	2.2187280023	-2.6541420772

14

C	-0.0011210386	0.0038554191	0.0014240102
C	-0.0017994373	-0.0014936245	1.5506112021
P	1.8719294511	-0.0018301144	2.1034374081
C	1.9349786206	0.5527654749	3.9751440591
C	1.8606642512	2.1002430606	3.977885291
C	-0.8990118572	-1.1489080732	2.054808453
C	-0.6547948842	1.3371007781	1.9605333215
C	2.5151929782	-1.8432913277	2.0116750856
C	4.0623968935	-1.768173475	2.0126110545

C	2.0495462744	-2.8174310041	3.1104931261
C	2.1395245799	-2.4458832375	0.6399565243
C	0.8587112006	-0.0106846891	4.9226991896
C	3.3224244728	0.2124763574	4.5622383847
H	2.0296833718	2.4670952523	4.9998632464
H	2.6334173487	2.5336347764	3.3339331795
H	0.8958911333	2.4889472404	3.6509405194
H	3.4234812449	0.7068755257	5.5379985627
H	3.4661996079	-0.8571762802	4.7304732608
H	4.1361101679	0.5772431391	3.9263447001
H	1.0261454276	0.3744099975	5.9391206528
H	-0.1512469916	0.2871443975	4.6303583312
H	0.8841531819	-1.1023640339	4.9793618073
H	-1.6328544725	1.4176136912	1.4669685554
H	-0.8327957962	1.416515208	3.0354208609
H	-0.0572101561	2.1977545441	1.6418400732
H	-1.0310913335	0.1335655049	-0.3589946277
H	0.5981324871	0.8329563247	-0.3898425473
H	0.3799731581	-0.9196675804	-0.4353183935
H	-1.923416599	-1.0033781164	1.6822030355
H	-0.5669188879	-2.1275744444	1.6991032926
H	-0.9522060421	-1.1885710769	3.1463145616
H	4.4723275602	-2.7709049936	1.8279146326
H	4.4256919545	-1.1063657622	1.2190991334
H	4.4790348399	-1.4177285156	2.9571629702
H	2.6776409122	-3.3951177119	0.5125282595
H	1.0742216013	-2.6681845334	0.5461106679
H	2.4327545557	-1.7912631429	-0.1877278272
H	2.4672901333	-3.8161572245	2.9168277344
H	2.387018589	-2.5179886689	4.1059587736
H	0.9613854984	-2.9194710613	3.1410894748

14H^{+}

C	-0.0028100415	0.0046127577	-0.0028342857
C	-0.0004415242	0.0006022042	1.5406120585
C	1.4473420213	-0.0027601371	2.0898673243
P	-0.904264081	-1.5425015433	2.2191143956
C	-2.8030784471	-1.3622579305	2.3860916722
C	-3.096757529	-0.3798833496	3.5463825211
C	-0.3076657573	-3.2101069221	1.4972390977
C	-0.5733923197	-4.3176362282	2.5458230063
C	-0.6673121748	1.2961521954	2.0537821193
C	-1.0325588284	-3.5216949119	0.1705663459
C	1.2171106763	-3.1891258594	1.2491481742
C	-3.4274703079	-0.8589162497	1.0677105792
C	-3.4514034462	-2.7116191144	2.7692119263
H	1.929929972	0.9237542516	1.7590314624
H	1.4697198681	-0.0058822502	3.1850175387
H	2.0553366125	-0.8290517617	1.7256598992
H	-0.060419481	2.1366967694	1.6991026531
H	-1.6770605579	1.4460091262	1.6678730488
H	-0.6868802864	1.3528240555	3.1460755452
H	0.5041584937	0.9143420157	-0.3433542256
H	0.5364660259	-0.8433937557	-0.4311327965
H	-1.0135681894	0.020308605	-0.4211039759
H	-0.4728147454	-1.6018020964	3.5573544716

H	-0.1584614422	-5.2528085769	2.153564531
H	-4.1828848991	-0.3494195621	3.6885767151
H	-0.0670046082	-4.1122115846	3.4951070308
H	-2.6564827063	-0.7174537321	4.4908504898
H	-1.6292397222	-4.4927887621	2.74425097
H	-2.7690879137	0.6403324721	3.3535250804
H	1.5051578452	-4.1911755479	0.9121033229
H	-4.5200489041	-2.5254531081	2.9240521005
H	1.5184320132	-2.4904961918	0.467021869
H	-3.3694001709	-3.4687705787	1.9874090498
H	1.7875710154	-2.9797951135	2.1587884083
H	-3.0578480973	-3.1169181719	3.7061204862
H	-0.6479599955	-4.4721536948	-0.2158655754
H	-4.5124922306	-0.7944580055	1.2057919408
H	-2.1119754839	-3.6371821305	0.292198488
H	-3.0799400876	0.1370159434	0.7840814954
H	-0.8492663923	-2.76193308	-0.5949943848
H	-3.247034173	-1.5409364457	0.2317184261

15

P	-0.2363538006	0.2951266366	-0.15020225
C	-0.0315583799	0.1025396543	1.6937143247
C	1.5607429478	0.0588315665	-0.6499082774
C	-0.9143549757	-1.3961698494	-0.5596601943
C	1.7554035766	0.1453983954	-2.1786906003
H	2.1396467044	0.8563839585	-0.1696781929
H	1.9516132276	-0.8957641008	-0.2752899514
H	-1.019612851	0.0649807509	2.1644416469
H	0.5213877052	-0.8042316589	1.9672417229
H	0.4957792043	0.972779357	2.0984999038
H	-0.9808231384	-1.5265143657	-1.6451402005
H	-0.3082497516	-2.2094837328	-0.1418674891
H	-1.9313205208	-1.4786320638	-0.1611433735
C	3.163488177	0.0136883375	-2.5684693014
H	1.1862028326	-0.641517657	-2.688551875
H	1.3757842246	1.1025979655	-2.5565973545
N	4.2830968178	-0.093416194	-2.856521538

15H⁺

P	0.0039048559	0.0526807708	-0.0671621581
C	-0.1346977482	0.2154334283	1.7360332747
C	1.7393834291	-0.0032984235	-0.601073548
C	-0.8905324356	-1.4178672193	-0.7115763518
H	1.7919384016	-0.0823293392	-1.6912861191
H	2.2496311391	0.9124530009	-0.286854795
H	2.24257285	-0.865254538	-0.1523339562
H	-1.1833376873	0.3510528785	2.0169789431
H	0.2683630852	-0.674801408	2.2286849431
H	0.4316443566	1.0927659943	2.0641645337
H	-0.8564460068	-1.3374700321	-1.80435066
H	-0.3174701331	-2.3091807709	-0.4319534037
C	-2.3550146092	-1.5572257913	-0.2231862427
H	-0.5890071696	1.1845475633	-0.6420343516
H	-2.8515028695	-2.3302283662	-0.8199724656
C	-3.0831326401	-0.2903750724	-0.3386978362
H	-2.3885332852	-1.8936292963	0.819058686

N	-3.5560095331	0.7670516212	-0.4134304927

16

C	0.07705174	0.0733973727	-0.0508420737
C	-0.0234582279	-0.0352955099	1.3449707789
C	1.1610497004	-0.1031261246	2.0979810985
C	2.4069351868	-0.0832333093	1.4731224048
C	2.4926895014	0.0259297828	0.0829317455
C	1.325384281	0.1086545228	-0.676467574
P	-1.6417637685	-0.0050561352	2.2360454666
C	-1.9278044754	-1.798063181	2.6315079263
C	-1.5569449412	-2.8542790287	1.7847223
C	-1.8172587102	-4.1760611378	2.1437268788
C	-2.4536844227	-4.4631343353	3.3547375157
C	-2.8239986815	-3.4227925078	4.2061859464
C	-2.5573896562	-2.0988111713	3.8475660868
H	-2.4554329523	0.0778849124	1.0699195907
H	-1.055186603	-2.641148408	0.8447699763
H	-1.5241974967	-4.9841833051	1.478203127
H	-2.6552656014	-5.4943085605	3.6329755685
H	-3.31457302	-3.6387959367	5.1517666309
H	-2.839614571	-1.2908821913	4.5183458319
H	1.1067048908	-0.1717201878	3.1821612068
H	3.3122307085	-0.1434953531	2.0716634572
H	3.4639403071	0.049877278	-0.4039777647
H	1.3833449127	0.1951936274	-1.7585284738
H	-0.8248476392	0.1311217272	-0.6550576176

16H^+

C	0.158129209	0.4640220877	-0.1187447548
C	0.0244955616	-0.1768061384	1.1253823184
C	1.1581616552	-0.5920985713	1.8471126236
C	2.4238822989	-0.3568273154	1.3175301766
C	2.5596475068	0.2839439192	0.0817885533
C	1.4323153534	0.6917305817	-0.6349244767
P	-1.6102326666	-0.4189928763	1.8128462056
C	-1.9193724176	-2.0228530977	2.544534859
C	-1.5302977558	-3.1914665305	1.8650836247
C	-1.8150338956	-4.4317156129	2.4293597003
C	-2.4783824902	-4.5079499574	3.6583619051
C	-2.8597896513	-3.3461924014	4.3332627944
C	-2.583072114	-2.0967754436	3.7816341987
H	-2.5153960567	-0.1173184024	0.7811637524
H	-1.0063757012	-3.1354947878	0.915043279
H	-1.5181972993	-5.3385775954	1.9121888773
H	-2.6953293286	-5.47890951	4.0931789187
H	-3.3686356111	-3.4106026383	5.2897021805
H	-2.8785291628	-1.1959069529	4.3125654516
H	1.055843386	-1.0981745161	2.8029531624
H	3.3041886176	-0.6745001653	1.867196647
H	3.5501373951	0.4623538287	-0.3256389038
H	1.5427831275	1.1826893105	-1.5964877249
H	-0.7158567864	0.7792686074	-0.6822596221
H	-1.9202410298	0.5268543463	2.8047036316

17

P	-0.120233549	0.2070712274	-0.0829659423
C	-0.0365687448	0.0236518667	1.7587998994
C	1.6548945569	0.0438230995	-0.588602694
C	-0.8430897275	-1.4125859161	-0.6188147124
C	2.2851352573	-1.1665311197	-0.9190709907
C	3.6228064835	-1.1977693933	-1.315604109
C	4.3302929511	-0.0030239492	-1.3757182831
C	3.7427065777	1.2171228059	-1.0602763317
C	2.4028037532	1.230970311	-0.6772222598
H	1.7282105488	-2.0973697485	-0.8740109531
H	4.1177915281	-2.1277135596	-1.5767445337
F	5.6232724016	-0.0267474693	-1.7585375629
H	4.3270262516	2.1289476967	-1.1288223955
H	1.9291200529	2.1823969846	-0.4479600083
C	-1.3926430428	-1.4681186416	-1.9115965947
C	-1.9692128305	-2.6372956771	-2.4044363544
C	-2.0097404241	-3.7562105625	-1.579853425
C	-1.4914032889	-3.7394218374	-0.2904187159
C	-0.9073030483	-2.5634909412	0.1824995049
H	-1.373486173	-0.5822223428	-2.5416583897
H	-2.3931829686	-2.6890560167	-3.4020003164
F	-2.5757543222	-4.8893742532	-2.0430293811
H	-1.5494939932	-4.633134301	0.3226596307
H	-0.5031485152	-2.5443589476	1.1899004196
C	-1.1432182702	0.4835173004	2.4938618386
C	-1.1750235589	0.4006967602	3.8847259827
C	-0.0729085243	-0.1334022792	4.5430987734
C	1.0472646117	-0.5854012393	3.8556053122
C	1.0571974173	-0.505138917	2.4624438825
H	-1.9918343218	0.9178819652	1.9710552295
H	-2.0263848427	0.7514813093	4.4589272044
F	-0.0884688371	-0.2080039779	5.8896003473
H	1.8892366753	-0.9890870182	4.4088640945
H	1.931377387	-0.8541159765	1.9216351673

17H⁺

P	0.0764359898	-0.1616847135	0.0814794271
C	0.0566469984	-0.10599685	1.878233229
C	1.7345319544	-0.0395884049	-0.6002364112
C	-0.8171724144	-1.5801044876	-0.5732023978
C	2.4216880946	-1.1892442136	-1.0286454465
C	3.7223958018	-1.0833961711	-1.5109856114
C	4.3175975651	0.1756060858	-1.5624565312
C	3.6558222471	1.3323456483	-1.1493234553
C	2.3585356624	1.2211745998	-0.6649414197
H	1.9434221801	-2.1633460303	-0.9977233836
H	4.275048418	-1.9522566825	-1.8512087635
F	5.5612802035	0.2803012028	-2.0284171346
H	4.1580310941	2.2911400165	-1.2171860506
H	1.8366556563	2.118416841	-0.3427518648
C	-1.0635703238	-1.6556800532	-1.9585737557
C	-1.7580030005	-2.7375812268	-2.4835301229
C	-2.1983581564	-3.7370881291	-1.6141032536
C	-1.967202304	-3.6864575982	-0.2416421461
C	-1.2742583456	-2.5982757671	0.2804864217
H	-0.7151433058	-0.8755242387	-2.6301497765

H	-1.9656792757	-2.8202123824	-3.5447654098
F	-2.8644164084	-4.7754048818	-2.1173470793
H	-2.3308138734	-4.4864601715	0.3938245721
H	-1.094752582	-2.5444157546	1.3492770663
C	-1.0806083778	0.3955926179	2.5398725869
C	-1.1216801037	0.425017065	3.9285877136
C	-0.0197112532	-0.0487743307	4.6409431447
C	1.1192855955	-0.5461994831	4.010121992
C	1.1565998778	-0.5714474924	2.6200039708
H	-1.9336406226	0.7675688499	1.9783737415
H	-1.9814820684	0.8116911547	4.4646446514
F	-0.0557627093	-0.0185382614	5.9723810323
H	1.9543073112	-0.8948253864	4.6078692919
H	2.0446472652	-0.94322895	2.1181121266
H	-0.5998150767	0.9978933744	-0.3366876209

18

P	-0.1267361898	0.2224463974	-0.0879027963
C	-0.0390652478	0.0374555639	1.7537014281
C	1.6473680864	0.0543449059	-0.5960604856
C	-0.8514921249	-1.3977712874	-0.6203518013
C	2.2704865575	-1.1551566797	-0.9384666086
C	3.6074068655	-1.1895128107	-1.337365131
C	4.3327011591	-0.0003208261	-1.390405357
C	3.7411170595	1.2185126215	-1.0593427358
C	2.4018365042	1.2368722102	-0.6741294858
H	1.7112038737	-2.0849628605	-0.9004657296
H	4.0817682015	-2.1285003507	-1.6033204161
Cl	6.0168626001	-0.0360879446	-1.8913690019
H	4.3166549747	2.1365627932	-1.1135068401
H	1.9361652589	2.1900581975	-0.4355633612
C	-1.3837936252	-1.4613715915	-1.9189132515
C	-1.9621647771	-2.6309366335	-2.4086930503
C	-2.0270819212	-3.7513514791	-1.5806523359
C	-1.5215045587	-3.7160844061	-0.2821993406
C	-0.9347651222	-2.5406224892	0.1888168639
H	-1.3512422492	-0.5825387192	-2.5584015712
H	-2.3675830178	-2.6716579494	-3.4141395605
Cl	-2.7666477836	-5.2282640485	-2.1810137179
H	-1.585051345	-4.5952602465	0.3504607642
H	-0.5431492313	-2.5190218829	1.2011517445
C	-1.155129394	0.4698678264	2.4894187735
C	-1.1840507024	0.3850006982	3.8801801297
C	-0.0711939641	-0.1232792603	4.5497457233
C	1.0577186229	-0.546146463	3.8499883895
C	1.0659006881	-0.4652536918	2.4567362718
H	-2.0153636839	0.8848675019	1.9696819147
H	-2.0518745767	0.718232126	4.4394805969
Cl	-0.0892614941	-0.2232191858	6.3042762901
H	1.9172363897	-0.9337263413	4.3869863264
H	1.9487616377	-0.7951864527	1.9179706949

18H^+

P	0.072079064	-0.1492433095	0.0761982351
C	0.0509659927	-0.0915431781	1.8740075185
C	1.7330217881	-0.0337495509	-0.603006433

C	-0.8246678119	-1.5681208003	-0.5763048168
C	2.4148198098	-1.1840017146	-1.0335159872
C	3.716448048	-1.0815444963	-1.5162827262
C	4.3325018465	0.1723270739	-1.5722115668
C	3.6616398234	1.3265709678	-1.1512239141
C	2.363244846	1.2223152233	-0.6660196988
H	1.934409776	-2.1572687418	-1.0051453605
H	4.2497302211	-1.9635971382	-1.8531913139
Cl	5.95410703	0.3019357909	-2.1805614118
H	4.152718479	2.29165426	-1.2093950773
H	1.847968814	2.1230148955	-0.3424646848
C	-1.0868135812	-1.6379794272	-1.957500044
C	-1.7797857019	-2.7230702903	-2.4798816492
C	-2.2101477664	-3.7423689971	-1.621145203
C	-1.9557689237	-3.6841813258	-0.2484390461
C	-1.2644411729	-2.5943005392	0.2744535644
H	-0.7532611649	-0.852320458	-2.6304167766
H	-1.9899249519	-2.7836137659	-3.5420021692
Cl	-3.0759408486	-5.0987349839	-2.2749035706
H	-2.2985718918	-4.4814150458	0.4017003902
H	-1.0748645377	-2.5481819035	1.3420343995
C	-1.0895576485	0.4001935904	2.5343393653
C	-1.1311327355	0.4289895706	3.9237428558
C	-0.030247173	-0.0340384975	4.6537175888
C	1.1109130452	-0.5207283535	4.0084665907
C	1.1519848439	-0.5468936846	2.6176223554
H	-1.9463942728	0.7652504756	1.9738889266
H	-2.0038618461	0.811479341	4.4413692462
Cl	-0.0806593441	0.0042334141	6.3893306532
H	1.95743585	-0.8672092044	4.5909575286
H	2.044930417	-0.9122955971	2.1195269173
H	-0.6020166085	1.0094521956	-0.3474113519

19

P	-0.1220976536	0.1996220769	-0.0894256052
C	-0.0424898767	0.023471268	1.7531315813
C	1.6547230162	0.0409554512	-0.590066172
C	-0.8379065763	-1.4244580162	-0.6211220492
C	2.2895831884	-1.1647383979	-0.9225945273
C	3.6306052358	-1.1827433522	-1.3080217205
C	4.3845463916	-0.0044709259	-1.374756086
C	3.7493860304	1.1994992367	-1.0415220988
C	2.4076852796	1.2242109107	-0.6667252965
H	1.7348872187	-2.097544511	-0.8814281504
H	4.1001540169	-2.1317171988	-1.559737998
C	5.8271871191	-0.0257181728	-1.8226745549
H	4.3106102887	2.1307212602	-1.084800186
H	1.9370161094	2.1760425663	-0.4303722304
C	-1.3618600758	-1.4996724032	-1.9226824319
C	-1.9270104215	-2.6787194478	-2.4024140277
C	-2.010819391	-3.8213806651	-1.5943364717
C	-1.4973774483	-3.742901387	-0.2944095656
C	-0.9194214128	-2.5669681414	0.1872549362
H	-1.326487379	-0.6236277751	-2.5666509831
H	-2.3184132883	-2.7107149639	-3.4172868041
C	-2.6626479109	-5.085359829	-2.1039142857

H	-1.54826667	-4.616075106	0.353044233
H	-0.5302305005	-2.5406861386	1.2007727091
C	-1.1589376554	0.4581493298	2.4869707647
C	-1.1835354272	0.3713342334	3.8769866361
C	-0.0863794586	-0.1327361227	4.5891267231
C	1.0299455478	-0.5563226082	3.857947207
C	1.0552754927	-0.4815008813	2.4644318991
H	-2.0182562853	0.8702030117	1.9624111075
H	-2.064532354	0.7089508873	4.4191357843
C	-0.0984668699	-0.186368019	6.0988341253
H	1.8945107384	-0.9533657951	4.3862847475
H	1.9379185993	-0.8186201732	1.9291163535
H	6.4099157615	0.7651427229	-1.3380067151
H	6.3035049654	-0.9855199127	-1.5958864779
H	5.9094847289	0.1295971684	-2.9069517018
H	0.6122055935	-0.9273877503	6.4794921484
H	0.1776023024	0.7837940725	6.5335652348
H	-1.0922871678	-0.4404461202	6.4839241298
H	-2.3475404742	-5.9602489296	-1.5258834613
H	-3.7570681935	-5.024455416	-2.0344387127
H	-2.417020134	-5.2678380363	-3.1561480071

19H^+

P	0.0760129407	-0.172990981	0.0765290532
C	0.0504584963	-0.1020352893	1.8727748001
C	1.7337574319	-0.0480298722	-0.6049946128
C	-0.8095844668	-1.601066779	-0.5692920497
C	2.4458222203	-1.1973840862	-0.9825968625
C	3.7506405249	-1.0760572679	-1.4542236515
C	4.3702846833	0.1770142782	-1.5683957631
C	3.6429742125	1.3175834464	-1.1883104674
C	2.3414666298	1.2156448506	-0.7116257225
H	1.9854690791	-2.17888342	-0.9185923425
H	4.2955311238	-1.9702401498	-1.7438214197
C	5.7704341944	0.3044618419	-2.1117497416
H	4.1034365023	2.2982424441	-1.2710979954
H	1.8046259209	2.1168267406	-0.4258537208
C	-0.986698223	-1.7320157356	-1.9601726166
C	-1.6835468495	-2.8211536859	-2.4658366513
C	-2.218017681	-3.8046021384	-1.6133834251
C	-2.0301049516	-3.6605733306	-0.2329131077
C	-1.3370203206	-2.5716594827	0.2937855734
H	-0.5809415806	-0.9914729982	-2.6447046368
H	-1.8184960558	-2.9135123525	-3.540207378
C	-2.9762476091	-4.976340743	-2.182536403
H	-2.4334656458	-4.4091582745	0.4429806838
H	-1.2113639171	-2.4812886047	1.3680474047
C	-1.0761074651	0.4240760881	2.5289017564
C	-1.1138997749	0.4529439498	3.9187112264
C	-0.0416991745	-0.0317226521	4.6856086607
C	1.0753161104	-0.5527407665	4.0148528515
C	1.1317935705	-0.5879178649	2.6245728895
H	-1.9204228232	0.8100876388	1.962945991
H	-1.9873229121	0.8627235933	4.4184139685
C	-0.0788652789	0.0352915316	6.1909818275
H	1.9158040768	-0.93112846	4.5900418884

H	2.0144866342	-0.9836418123	2.130675317
H	-0.6009277691	0.984344675	-0.3480533714
H	6.3260783306	1.0984837507	-1.603051378
H	6.32949428	-0.6296348676	-2.0071348956
H	5.7473438486	0.5574383785	-3.179649243
H	-3.2847806694	-5.6758455683	-1.401291258
H	-3.8768213478	-4.6394640714	-2.7098409674
H	-2.3655411257	-5.5248847637	-2.9090198607
H	0.5335236261	-0.7501947465	6.6434077647
H	0.3118692538	0.9989579364	6.5428092176
H	-1.1002660496	-0.0600603792	6.5714906678

20

P	-0.1678678725	0.3022158905	-0.1239311432
C	0.0054647833	0.1735964632	1.7144311848
C	1.5727225011	0.0865107897	-0.714587382
C	-0.9526529472	-1.3134749331	-0.5705745701
C	2.1616617654	-1.1404159817	-1.0415507604
C	3.4759069625	-1.2207868096	-1.5126012485
C	4.2315002775	-0.0522059311	-1.6600028569
C	3.6574587309	1.1877299462	-1.340018687
C	2.3480839305	1.2501500809	-0.8847982154
H	1.5889570358	-2.0573406416	-0.9370277415
H	3.8925594742	-2.1909471243	-1.7599616646
O	5.5196286987	-0.0097416978	-2.1091920393
H	4.2548654142	2.085089253	-1.469315764
H	1.912893926	2.2212945156	-0.6601585924
C	-1.5747116121	-1.4061010613	-1.8310144553
C	-2.2032321702	-2.572104819	-2.2447414246
C	-2.2459581297	-3.6871044094	-1.3936671579
C	-1.6469294217	-3.6141264546	-0.1312745434
C	-1.0076266835	-2.4350921099	0.2647924827
H	-1.568701099	-0.5452837766	-2.4958476151
H	-2.6811576143	-2.6424878822	-3.2171154451
O	-2.8966046428	-4.7819783009	-1.8843435217
H	-1.6713966322	-4.4583240475	0.5488200766
H	-0.5520648964	-2.395954657	1.2499555696
C	-1.0601440599	0.6549889384	2.4999777981
C	-1.0159371568	0.6103496888	3.8861850457
C	0.1158472601	0.0951275118	4.5365187069
C	1.193660907	-0.3738860959	3.7773769134
C	1.1271529419	-0.3325455035	2.3810506141
H	-1.9373420291	1.0750409716	2.0130404626
H	-1.8389681501	0.9793881784	4.4905772192
O	0.0692486804	0.1020587977	5.9006170583
H	2.0834836734	-0.76907734	4.254816929
H	1.9742614305	-0.6985118029	1.8083348401
C	1.1901194305	-0.3954687946	6.6134006917
H	0.9408709084	-0.2949265456	7.6714034646
H	1.3781558226	-1.4529688935	6.3845173119
H	2.0967668544	0.1853320244	6.3978058634
C	6.1488902409	-1.2311730517	-2.4612810003
H	7.1549998486	-0.9654879523	-2.7910649318
H	6.2173350455	-1.9129052736	-1.6030641249
H	5.620639638	-1.7367552877	-3.2805101029
C	-2.9919540667	-5.932396478	-1.0600134945

H	-3.5496934398	-6.6704420514	-1.6395152031
H	-2.0012850334	-6.3380742835	-0.8150115089
H	-3.532900113	-5.7185195612	-0.1286592601

20H+

P	0.098027428	-0.159313857	0.0643578692
C	0.1164397104	-0.0019882646	1.8495631021
C	1.7479187239	-0.1255330615	-0.6383092701
C	-0.857859109	-1.5675568601	-0.4994678373
C	2.280350086	-1.2458747035	-1.2922690036
C	3.5643650987	-1.2114754185	-1.8304196016
C	4.3339385945	-0.0415146642	-1.7222450255
C	3.8004632401	1.0889151981	-1.0674986331
C	2.5271861854	1.0482535958	-0.5333987155
H	1.6920135774	-2.152947809	-1.3906403916
H	3.9526466826	-2.0892272834	-2.3325180618
O	5.5782352012	0.1069810948	-2.2063425931
H	4.4109672752	1.9831978482	-1.0014652147
H	2.1359850078	1.9308046698	-0.0335282368
C	-1.3517885532	-1.594314312	-1.8222886472
C	-2.0739659277	-2.6823445585	-2.2740038355
C	-2.3243500089	-3.7750292445	-1.4175429825
C	-1.8389416618	-3.7529835787	-0.0992278065
C	-1.1137097211	-2.6530574334	0.3509399649
H	-1.1729493571	-0.7615034528	-2.49787151
H	-2.4667532169	-2.7178119514	-3.2845780272
O	-3.0365242799	-4.7785607511	-1.9565315691
H	-2.0272198442	-4.57769759	0.5773678369
H	-0.754179311	-2.6426461443	1.3756052936
C	-1.030302084	0.477202199	2.520117304
C	-1.0395504721	0.5793505408	3.8979292418
C	0.0958342753	0.2043203098	4.646607021
C	1.2404656381	-0.2724915049	3.9856511872
C	1.2449951452	-0.3701787803	2.5968042506
H	-1.9148714925	0.7740835868	1.9621878103
H	-1.9082263243	0.9528697504	4.4295894424
O	-0.0190019048	0.3458499375	5.9774643883
H	2.1254588295	-0.5592642079	4.540522051
H	2.1401278753	-0.7273230407	2.096393492
C	1.0853938787	0.0026731729	6.8191167904
H	0.7505354141	0.2065723788	7.8358515251
H	1.3391720855	-1.0590137365	6.7217523571
H	1.9614510759	0.619899032	6.5899822191
C	6.1964736346	-0.980792176	-2.8994136141
H	7.1792299082	-0.6155535096	-3.1964798988
H	6.3106341721	-1.8511663168	-2.2432718481
H	5.6216590463	-1.2563411053	-3.7907670449
C	-3.3486431785	-5.9251494142	-1.1605522747
H	-3.923894115	-6.5832395273	-1.8112994619
H	-2.4364896051	-6.4372107094	-0.8336188813
H	-3.9544939826	-5.6470924295	-0.290747963
H	-0.5754209851	0.980516133	-0.4147274197

21

P	-0.0822486278	0.2245082123	-0.0857013321
C	0.0022185863	0.0054787197	1.7533352848

C	1.6943746596	0.0023332411	-0.5705146868
C	-0.8596100237	-1.3642570834	-0.6341337902
C	2.304633505	-1.2350911241	-0.827824038
C	3.6445020433	-1.3001291779	-1.2143794143
C	4.3972561132	-0.1312660323	-1.3438831735
C	3.8021528001	1.1062058854	-1.092313907
C	2.4594018506	1.1713451663	-0.7182007312
H	1.7298473969	-2.1515043069	-0.7329679801
H	4.1007144944	-2.2664675318	-1.4141510553
H	5.4400303655	-0.1842899659	-1.6457112672
H	4.3789826095	2.0213502084	-1.1981924043
H	1.995854668	2.1397241733	-0.5440333341
C	-1.4702615731	-1.3667955991	-1.8984536843
C	-2.0793661209	-2.5188836461	-2.3964302156
C	-2.1009686049	-3.6850482514	-1.6292884322
C	-1.508180705	-3.6927147282	-0.3653717639
C	-0.8903282258	-2.5421753284	0.1282706949
H	-1.4746315588	-0.4560890324	-2.4928583168
H	-2.5459606935	-2.502363116	-3.3779466512
H	-2.582924486	-4.5811548386	-2.0113075588
H	-1.5259463084	-4.5961152591	0.2391364388
H	-0.4345171248	-2.5600840763	1.1141450885
C	-1.1867556846	0.1320548544	2.4805098646
C	-1.2466739938	0.0753807947	3.8657497292
C	-0.0605984532	-0.1100530847	4.5782584634
C	1.149168049	-0.2363980882	3.8943013111
C	1.1767353051	-0.1807543717	2.4986924353
F	-2.3409936253	0.3158431918	1.7972123447
H	-2.2072896774	0.1776937709	4.3601681623
H	-0.0863408565	-0.15407972	5.6634135422
H	2.0743197497	-0.3816187534	4.4449272054
H	2.1231921473	-0.2813331024	1.9773091718

35

$21H^+$

P	0.0983485276	-0.1548225694	0.0484410682
C	0.0664863554	-0.0700622603	1.8457362817
C	1.7897362638	-0.1031708971	-0.5697858083
C	-0.8352311266	-1.5537172635	-0.5876979243
C	2.4166478534	-1.2712815102	-1.030761168
C	3.7329401805	-1.2078102634	-1.4872528574
C	4.4158420219	0.0100294126	-1.4893763586
C	3.7890040379	1.1742625145	-1.0344973363
C	2.4760875303	1.1243779704	-0.573311587
H	1.8830819234	-2.2167882444	-1.0434079424
H	4.2206890079	-2.1082403763	-1.847290731
H	5.4384427319	0.055087836	-1.8517164561
H	4.3202406866	2.1208305953	-1.04478099
H	1.9930118407	2.0337314587	-0.224556978
C	-1.4456432048	-1.4462957056	-1.8489671893
C	-2.1397535451	-2.5361782917	-2.3688767226
C	-2.2240814129	-3.7250689886	-1.6389224658
C	-1.6182493145	-3.8301486083	-0.3847558297
C	-0.9231818065	-2.7457687471	0.1488711823
H	-1.3891238982	-0.52224655	-2.418455795
H	-2.6195998666	-2.4552546577	-3.3391301288
H	-2.7696153258	-4.5705541309	-2.0470281708

H	-1.6926975785	-4.7528196652	0.1822072935
H	-0.4665298249	-2.8250225889	1.1311623904
C	-1.1387606091	0.252784417	2.4824414899
C	-1.2491607962	0.3313804325	3.861057698
C	-0.1126095198	0.0716832401	4.6303305741
C	1.1055125463	-0.2543280014	4.0253020054
C	1.2005382575	-0.3222271054	2.6371644327
F	-2.2134779122	0.4975385246	1.7071375781
H	-2.2025991798	0.5907821091	4.3083061333
H	-0.1811975728	0.1282222648	5.7122938268
H	1.9812909236	-0.4496185272	4.635080259
H	2.1481411018	-0.5624701682	2.1655034085
H	-0.538176296	0.9978643449	-0.4358851824

22

P	-0.1067035114	0.23513609	-0.0230456974
C	0.0218624605	-0.0459290556	1.8048145142
C	1.6539042379	0.0448696065	-0.5664027788
C	-0.8557864338	-1.3603798717	-0.5973538244
C	2.3076038403	-1.1882041532	-0.7186612386
C	3.6341217193	-1.2401030214	-1.1482175303
C	4.3283752985	-0.0604812917	-1.4275900686
C	3.6897434132	1.1715037692	-1.2802212132
C	2.359924321	1.2220570306	-0.8586662312
H	1.7749798951	-2.1108223373	-0.5060824023
H	4.1261826204	-2.2023273788	-1.265380238
H	5.3609753927	-0.1027477826	-1.7641077547
H	4.2218672593	2.0926622758	-1.5027886996
H	1.8597651128	2.1828341701	-0.7637631394
C	-1.1605316488	-1.475107762	-1.9578736918
C	-1.7829607949	-2.5852453733	-2.5113638753
C	-2.1300590787	-3.6441291911	-1.6709635047
C	-1.8458247008	-3.5716581095	-0.3066232575
C	-1.2147691464	-2.4425311038	0.2201596809
F	-0.8259109149	-0.4531853676	-2.7800559895
H	-1.9854876941	-2.6037040853	-3.5772873753
H	-2.6204506982	-4.5207149703	-2.0847478472
H	-2.1122174308	-4.3945835052	0.3505599081
H	-0.9947422716	-2.4012928059	1.2822136422
C	-1.1461221474	0.0690928395	2.5673050786
C	-1.1679966833	-0.0085201173	3.9528979427
C	0.0377177793	-0.2013996428	4.6291637672
C	1.2281763889	-0.3149244505	3.9097216069
C	1.216927374	-0.2397509247	2.5150886691
F	-2.319687254	0.257171643	1.9184583485
H	-2.1147524332	0.0847022171	4.4750273143
H	0.04239295	-0.2610408294	5.7138355814
H	2.168752879	-0.4647722082	4.4321276989
H	2.1490748998	-0.3294553027	1.9669326049

22H^+

P	0.0984471342	-0.1419254255	0.0751170949
C	0.0903414497	-0.1294551093	1.8749827437
C	1.771314073	-0.0711959589	-0.5781799263
C	-0.8340799349	-1.5394583898	-0.5634270354
C	2.4874722457	-1.2528684492	-0.8299600036

C	3.7958558136	-1.1719302248	-1.3036735517
C	4.3834418514	0.0754255444	-1.5294819696
C	3.6677320175	1.2502531755	-1.2842494103
C	2.3602446779	1.1837703226	-0.8078330226
H	2.02737834	-2.2242775983	-0.6728155586
H	4.3524604393	-2.0820200371	-1.5042667307
H	5.4006569818	0.1322506282	-1.9050106173
H	4.1237664757	2.2174731836	-1.4704417042
H	1.8055831737	2.1008059749	-0.6269312664
C	-1.2235375336	-1.5228518919	-1.9084611811
C	-1.9379119544	-2.5605553319	-2.4841786103
C	-2.2688112166	-3.6570440346	-1.6850259461
C	-1.8919882507	-3.7051032037	-0.3388879696
C	-1.1795671258	-2.6497161019	0.2255408307
F	-0.8845347432	-0.4481895827	-2.6476078593
H	-2.2231662397	-2.4997651305	-3.5287951101
H	-2.8285116571	-4.4795215582	-2.1194703476
H	-2.1587142089	-4.5617415667	0.2709939735
H	-0.8965255358	-2.6804698109	1.273022041
C	-1.1059394293	0.1649696147	2.54141311
C	-1.1906571639	0.2126811815	3.9233110365
C	-0.0357546445	-0.0477709165	4.6646001408
C	1.1741991779	-0.345210981	4.0292036084
C	1.2427995753	-0.3831425674	2.6384406892
F	-2.1985065513	0.4118685231	1.7915670496
H	-2.1382789395	0.4504559769	4.3943260322
H	-0.0834340695	-0.0141385658	5.7486334691
H	2.0643685476	-0.5409587673	4.6175898422
H	2.1834012519	-0.6011455642	2.142803487
H	-0.5366390275	1.0315836427	-0.3522493276

23

P	-0.1231237149	0.2078831006	-0.0828061648
C	-0.0149897501	0.0010645867	1.7558342
C	1.6518976638	0.0151362259	-0.5798391665
C	-0.8287381345	-1.4268455451	-0.5975519801
C	2.2312517105	-1.1470738824	-1.1119640982
C	3.5581478299	-1.1628161924	-1.5463624235
C	4.3401004691	-0.0102998722	-1.4521493151
C	3.7956005199	1.1600541624	-0.9219937567
C	2.4721804881	1.1460766881	-0.5024161234
H	1.632002368	-2.0489716014	-1.1880507558
H	3.979791915	-2.0761923366	-1.9560856486
H	5.3728679061	-0.0178735686	-1.7890149843
H	4.3715490764	2.0751626532	-0.8305481991
F	1.9548142657	2.2833146343	0.0163326661
C	-1.1972455279	-1.5800785093	-1.9386209569
C	-1.8134704407	-2.7203755704	-2.436163608
C	-2.0854109151	-3.7696283552	-1.5571602512
C	-1.7338425327	-3.6599967695	-0.2107672327
C	-1.1106274106	-2.5021569453	0.2590037056
F	-0.9312983618	-0.570422337	-2.7990628423
H	-2.0694204767	-2.7684564022	-3.489577356
H	-2.5701467633	-4.6686790762	-1.9271173656
H	-1.9416140933	-4.475932431	0.4755291949
H	-0.8391031383	-2.4280968791	1.3072911148

C	-1.1686345687	0.2550212184	2.5058037262
C	-1.2006833774	0.2212530427	3.8934492738
C	-0.0236140898	-0.0767010901	4.5813001188
C	1.1505666085	-0.3377723359	3.8730769723
C	1.1501739439	-0.3011238105	2.4772684414
F	-2.3156891567	0.5392454829	1.8472099251
H	-2.1335064461	0.4283070465	4.4077142313
H	-0.0280906911	-0.1039904397	5.6672823486
H	2.067997697	-0.5723374717	4.4052545403
H	2.0681901278	-0.50723442	1.9360647696

23H+

P	0.08857776	-0.1515609037	0.0573305736
C	0.0678656123	-0.0865446885	1.852841672
C	1.7680316908	-0.1092069294	-0.5803162492
C	-0.8061791394	-1.5839048376	-0.556518973
C	2.394794722	-1.2333141584	-1.1448767581
C	3.6976000665	-1.1303272379	-1.6264709599
C	4.3783458128	0.0894790327	-1.5543574348
C	3.7713708142	1.2183175872	-0.9996620436
C	2.4773905917	1.0969365123	-0.5200861912
H	1.8610428324	-2.1761690584	-1.2119247738
H	4.1801851093	-1.9981731906	-2.06331806
H	5.3917875525	0.1665398764	-1.935774296
H	4.278077117	2.1752493426	-0.9374847661
F	1.8593229847	2.163364463	0.0245569172
C	-1.2428687379	-1.5837588268	-1.8872024138
C	-1.9452838993	-2.6451867407	-2.4344616257
C	-2.214527126	-3.7481024105	-1.6211887743
C	-1.7898021143	-3.7796437494	-0.2887694981
C	-1.0902995982	-2.7013030936	0.2473291401
F	-0.9609988909	-0.5034867547	-2.6417553044
H	-2.2688093464	-2.5970818967	-3.4685506186
H	-2.7640183769	-4.5886660985	-2.0334995355
H	-2.0096774426	-4.6413179555	0.332582007
H	-0.7721181334	-2.7187456116	1.285080933
C	-1.1167693246	0.2948052834	2.4951414524
C	-1.2145774707	0.3823956742	3.874282887
C	-0.0870250066	0.070511871	4.6369448249
C	1.1098731743	-0.3171954764	4.0254268235
C	1.1927444781	-0.3936546324	2.6373077244
F	-2.1843374578	0.5839499788	1.7253982028
H	-2.1514801761	0.6891378566	4.3264332415
H	-0.1453419706	0.1341567463	5.7190886347
H	1.9788989792	-0.5529538427	4.6304956192
H	2.1246783237	-0.6815620421	2.1608253206
H	-0.5711534098	0.9890269113	-0.412643698

24

P	-0.0820461788	0.0804672055	-0.2125296156
C	-0.0972327456	0.0624186315	1.6493249205
C	1.7230676041	-0.0503139368	-0.5974197391
C	-0.7648890079	-1.5870927614	-0.6443488673
C	2.2362047592	-0.9849104828	-1.5074120358
C	3.5819817443	-0.9496846512	-1.880458783
C	4.4359130238	0.0177371574	-1.352069111

C	3.9322596669	0.9608741205	-0.4527711884
C	2.5877425207	0.9335906227	-0.0883834894
H	1.588343879	-1.7504101492	-1.9229423049
H	3.9618302108	-1.6866235335	-2.583730332
H	5.4832955719	0.0414247064	-1.6408504422
H	4.58692614	1.7230920313	-0.0377636202
H	2.2089404995	1.6822875241	0.6038906982
C	-1.8011865488	-1.6033931275	-1.5905565819
C	-2.3966866938	-2.8054039253	-1.9808303374
C	-1.9640207465	-4.008479883	-1.424664153
C	-0.9332371059	-4.0063729027	-0.4804752824
C	-0.3362607559	-2.8074407153	-0.0929487308
H	-2.145385913	-0.6658788978	-2.0197075453
H	-3.198162365	-2.7987718859	-2.7148333985
H	-2.4269167819	-4.9455068065	-1.7232026384
H	-0.5928898548	-4.9424652898	-0.0449822798
H	0.4674019516	-2.8190562609	0.6348596743
C	-1.0628371693	0.8401217668	2.3051620796
C	-1.1632072706	0.9533627332	3.6876381701
C	-0.2534033745	0.2564129249	4.4802160377
C	0.7279547924	-0.540412541	3.8909416276
C	0.7780117061	-0.6212693991	2.504398005
F	-1.9566809918	1.5212929362	1.5601244614
H	-1.9412241744	1.5773239487	4.1133626893
H	-0.3113860658	0.3298087907	5.5620071989
H	1.4460090333	-1.1007880955	4.4797294373
F	1.7287976407	-1.4199538545	1.9708314768

24H^{+}

P	0.0611722137	-0.2191836853	0.1665766441
C	0.1085407783	-0.3014491858	1.9654151437
C	1.6586744057	0.10371816	-0.5930347226
C	-0.7850494034	-1.649463488	-0.5314520733
C	2.7284840748	-0.7991756591	-0.4529516299
C	3.9476524194	-0.5102217135	-1.0598267237
C	4.1054390124	0.666228452	-1.7993805399
C	3.0444046611	1.5628306871	-1.9360350811
C	1.8175790051	1.2879986977	-1.3334689679
H	2.6128481192	-1.7098000829	0.1250231779
H	4.7768331859	-1.2027657973	-0.9539590457
H	5.0600455283	0.8841928755	-2.2686703263
H	3.169066087	2.4763645105	-2.5087769217
H	0.99667659	1.9915659731	-1.4417417712
C	-0.4005760311	-2.1369639154	-1.7905959461
C	-1.1023822033	-3.2009697383	-2.3556873405
C	-2.1786165373	-3.7720114323	-1.6738700856
C	-2.5611616067	-3.2835840745	-0.4213682745
C	-1.867978519	-2.222844395	0.1562122595
H	0.4381594428	-1.6990332824	-2.3221590724
H	-0.8051575497	-3.5839724876	-3.3268722984
H	-2.7206625998	-4.6014647925	-2.1179230568
H	-3.3970304016	-3.7307158915	0.1074705612
H	-2.1667814961	-1.8515872533	1.1321797479
C	-0.374480836	0.7475889072	2.7647049966
C	-0.3395133062	0.7029222032	4.1503321329
C	0.2014289805	-0.426176223	4.7676039526

C	0.7019702371	-1.495180938	4.0183755595
C	0.6468909622	-1.4086549037	2.6383626816
F	-0.8883569263	1.829189171	2.15544864
H	-0.7283701601	1.5393855189	4.7197834647
H	0.2374481084	-0.4738369867	5.8511684293
H	1.1285743462	-2.3759694753	4.4845304559
F	1.1299459618	-2.4199090863	1.8890837591
H	-0.6809615429	0.9395993318	-0.0918047288

25

P	0.0515668725	0.2842342937	0.0627335727
C	0.0399185109	-0.01563043	1.8935282604
C	1.7941329476	-0.091433974	-0.453078379
C	-0.8246584955	-1.1962836057	-0.6454971804
C	2.5139472136	-1.2395572304	-0.0853904635
C	3.8093804598	-1.4393938496	-0.5588181101
C	4.4027332397	-0.5015391886	-1.4088564927
C	3.6947520046	0.6396229905	-1.7826085448
C	2.3979700381	0.8438418813	-1.3045540281
H	2.0610015353	-1.9770000021	0.568270684
H	4.3582297681	-2.3304817633	-0.26471607
H	5.4127740761	-0.6623074001	-1.7766901024
H	4.1492022544	1.3740218931	-2.4423864682
H	1.8512491977	1.7382976077	-1.5917191815
C	-1.5744879296	-1.0019389727	-1.8125271353
C	-2.2716499493	-2.0131126644	-2.4651310647
C	-2.2309950823	-3.2997535866	-1.9328859261
C	-1.4995627732	-3.559035525	-0.773496736
C	-0.8188157202	-2.511676703	-0.1674830823
F	-1.6215713421	0.2345862753	-2.3508507296
H	-2.8268522104	-1.777171904	-3.3661573005
H	-2.7681996557	-4.1055423985	-2.4237766824
H	-1.4470729995	-4.5491620341	-0.3341260614
F	-0.1138722598	-2.7994790421	0.9513401318
C	-1.0814405308	-0.4929494068	2.5840981262
C	-1.1603939359	-0.5944515584	3.9664939057
C	-0.074184011	-0.1733293537	4.7326205385
C	1.0560354998	0.3508658165	4.1085018145
C	1.079687673	0.4299495106	2.7209253963
F	-2.1651720541	-0.8728377867	1.8694038392
H	-2.0649112767	-0.9909283502	4.4145558147
H	-0.1143046553	-0.2417822779	5.8153020575
H	1.9124423741	0.7089508161	4.6692904479
F	2.1680342164	0.9897109228	2.1499681498

25H^+

P	0.1481734926	-0.2448943791	0.0304538854
C	0.018520678	-0.0452466379	1.812028561
C	1.8426256567	-0.1411554231	-0.5652852368
C	-0.7278917816	-1.6900742465	-0.5794585617
C	2.3371345591	-1.0868390123	-1.47419144
C	3.6384670771	-0.9540113201	-1.9581243796
C	4.4351548156	0.1131516558	-1.5413039836
C	3.9377253863	1.0566980439	-0.6368863938
C	2.6420027437	0.9353016695	-0.143763652
H	1.7206969792	-1.9178830758	-1.8014228338

H	4.0271518668	-1.6856126042	-2.6593834785
H	5.4474673538	0.2122178725	-1.9211765422
H	4.5596502368	1.8860900487	-0.3151581771
H	2.2637400001	1.6691297086	0.5629826233
C	-1.5916877578	-1.591399017	-1.6833015579
C	-2.3135255935	-2.6736316945	-2.1607191101
C	-2.1673688332	-3.9050996585	-1.5181016385
C	-1.3155396902	-4.0581165015	-0.4208261429
C	-0.6128802069	-2.951258845	0.0256772307
F	-1.7064385328	-0.3987765972	-2.2903835842
H	-2.9677142179	-2.5436061635	-3.0152529988
H	-2.724746596	-4.7626606063	-1.8809792052
H	-1.191153593	-5.0096451737	0.0833674177
F	0.2191389983	-3.069280379	1.0729537975
C	-1.0665546793	0.6642652129	2.3559784957
C	-1.2192592042	0.8697838223	3.7166294793
C	-0.2563220186	0.3378041499	4.5777353847
C	0.8346334653	-0.3851113636	4.0905398262
C	0.9529109004	-0.5685993476	2.7215700205
F	-1.9864821477	1.1532104813	1.5050746055
H	-2.0742164063	1.4279153728	4.0806958594
H	-0.3607128696	0.485832706	5.6477743901
H	1.5846672706	-0.807999267	4.7491531097
F	1.9895135989	-1.265426893	2.2400998083
H	-0.5794899507	0.8306264623	-0.4857915781

26

P	0.0712861931	0.1222478661	-0.228437928
C	-0.0040102091	0.0426597487	1.6275050426
C	1.8615195728	-0.117681781	-0.6331281118
C	-0.7423054246	-1.4897899336	-0.6345020561
C	2.4103831944	-1.0337836212	-1.5365144128
C	3.7506472925	-1.0335175752	-1.9108915345
C	4.6039169895	-0.0709786642	-1.3770457346
C	4.1111567573	0.8825818389	-0.4852935327
C	2.7674286818	0.835500202	-0.1497699501
F	1.617638323	-1.9696910681	-2.1023286375
H	4.0967940866	-1.7845888662	-2.6125177178
H	5.6519417828	-0.0588137452	-1.6598415955
H	4.7406795862	1.6519699692	-0.0522023054
F	2.2890950823	1.7708325839	0.7036477292
C	-1.8533761781	-1.4872228764	-1.4850079862
C	-2.5817873553	-2.6309138194	-1.7985902649
C	-2.1861034001	-3.8453507291	-1.2425668707
C	-1.0814851115	-3.9109254982	-0.3912007071
C	-0.3902347295	-2.740225497	-0.1126278209
F	-2.2483771918	-0.3225172846	-2.0340782379
H	-3.4332989553	-2.5509161279	-2.4650760922
H	-2.7404485946	-4.7497953474	-1.4745730389
H	-0.7474057846	-4.8441093242	0.0488010958
F	0.6820318267	-2.8172494918	0.6988755918
C	-1.1838830218	0.5287668061	2.2077005177
C	-1.4111856129	0.5993665084	3.5753104898
C	-0.4043901595	0.1621289686	4.4353399211
C	0.7904943278	-0.3370390199	3.9209232494
C	0.9632355172	-0.3927647037	2.5414535436

F	-2.1695024815	0.9438438327	1.3814102021
H	-2.356311433	0.9877351222	3.9381139607
H	-0.5531018352	0.2074760026	5.5098986775
H	1.5898933723	-0.6910870103	4.5627103568
F	2.1280848922	-0.8954794647	2.0919841575

26H^+

P	0.1492248229	-0.2577104596	0.111151455
C	0.052828104	-0.1712435689	1.9009924525
C	1.8222127456	-0.113170612	-0.5216909058
C	-0.761889861	-1.6369383645	-0.58731242
C	2.6545960554	-1.2152258744	-0.7701821298
C	3.9301045335	-1.0752212093	-1.2921347388
C	4.399332593	0.2105487878	-1.571993175
C	3.6136220164	1.340819699	-1.3336941868
C	2.3447684612	1.1586173608	-0.8101281761
F	2.1867660079	-2.4387510489	-0.4851919308
H	4.5337480728	-1.9584696231	-1.46690909
H	5.3975039239	0.3342888312	-1.9795035596
H	3.9675852989	2.3441029108	-1.541385966
F	1.56818492	2.2270318669	-0.5577138311
C	-1.4974771785	-1.4513305594	-1.7697493687
C	-2.2513605713	-2.4610357641	-2.3437187882
C	-2.2669401961	-3.7109285807	-1.7198898976
C	-1.5439507026	-3.9497504469	-0.5485868714
C	-0.8030329178	-2.9130893587	-0.0051209966
F	-1.4516734743	-0.240248629	-2.3524436297
H	-2.8034753643	-2.2640629166	-3.2555189111
H	-2.8500748677	-4.5150644132	-2.1569734857
H	-1.5436992664	-4.9174415646	-0.0600883422
F	-0.0939961212	-3.1218695963	1.1132850511
C	-0.9942183335	0.546370992	2.5030048543
C	-1.1092782171	0.6839818396	3.8760021028
C	-0.1470579215	0.0742798769	4.6847219825
C	0.9074307451	-0.6593882083	4.1356416795
C	0.9878930441	-0.7724766201	2.757342845
F	-1.9112335971	1.110706194	1.6974578775
H	-1.9352204893	1.2513943442	4.289368606
H	-0.2227161597	0.169147575	5.763145775
H	1.6555645342	-1.1420509248	4.7540180604
F	1.985896463	-1.479466608	2.2084433683
H	-0.5106771025	0.8809646734	-0.3435517087

27

P	0.0936032717	-0.1680172071	0.0899865556
C	0.1157941825	-0.2460862948	1.9494106862
C	1.8547358484	-0.1681489105	-0.5132756892
C	-0.7431931903	-1.7022520603	-0.5505698141
C	2.5949150933	-1.1988964246	-1.1315085582
C	3.8253118789	-0.9734796485	-1.7509308619
C	4.3826866795	0.3000054693	-1.748982624
C	3.7142846838	1.3452212247	-1.1200935616
C	2.4783077696	1.1020109209	-0.5285208609
Cl	2.0735558222	-2.875713471	-1.1091237063
H	4.3434940852	-1.8062816304	-2.2129333216
H	5.3420173704	0.474848945	-2.2267541681

H	4.136042265	2.3433797621	-1.0870109162
Cl	1.7136933021	2.4808857165	0.2595359522
C	-1.2589097954	-1.5943764753	-1.8639417866
C	-2.0826082386	-2.5518931982	-2.4481635232
C	-2.442527573	-3.6765744095	-1.713081339
C	-1.9520313348	-3.843721134	-0.4231593816
C	-1.1090484089	-2.8809854918	0.1343199951
Cl	-0.8411660708	-0.212877645	-2.8757696861
H	-2.4348918698	-2.4068817317	-3.4631213485
H	-3.0948659083	-4.4274217255	-2.1487694056
H	-2.2003740665	-4.7260172509	0.155967109
Cl	-0.4753794653	-3.2819141003	1.7223742221
C	-1.0630643369	0.21951592	2.5785796456
C	-1.1721394861	0.4274355728	3.950270201
C	-0.0698488495	0.1838497739	4.7629745391
C	1.1095367257	-0.2923652855	4.2018262368
C	1.1888634055	-0.5150465295	2.8261770881
Cl	-2.5151082348	0.534932317	1.6305135023
H	-2.109971607	0.7791401709	4.3650575742
H	-0.1333976791	0.3533368116	5.8336648892
H	1.9705956789	-0.5136822308	4.822407408
Cl	2.6957760524	-1.2299157498	2.2765449482

27H⁺

P	0.2636545556	-0.4440554818	0.197019567
C	0.1582901952	-0.2898203877	2.0078061912
C	1.9246764287	-0.2205583681	-0.5143516215
C	-0.7342697534	-1.7783106862	-0.5369703554
C	2.7061120714	-1.2474595815	-1.0880718463
C	3.9122681956	-0.9665435998	-1.7255085469
C	4.3732216771	0.3456116274	-1.7902185414
C	3.6439136846	1.3818865263	-1.2129905432
C	2.4366092298	1.0977586243	-0.5829588968
Cl	2.2372599254	-2.9246491661	-0.9908820144
H	4.4848439325	-1.7796653165	-2.1567713127
H	5.3146877011	0.5611639977	-2.2855734375
H	4.0031840224	2.4041789818	-1.2427007852
Cl	1.5828069809	2.4418236386	0.1495550627
C	-1.2994271829	-1.5478095059	-1.8145025568
C	-2.1721764706	-2.4571335091	-2.403282498
C	-2.4989402493	-3.6279495731	-1.7239781999
C	-1.9469245714	-3.8996128553	-0.4750403576
C	-1.0687093385	-2.9893069898	0.1081435176
Cl	-0.9056175932	-0.1087601039	-2.7343160319
H	-2.5827786896	-2.2450322766	-3.3838109308
H	-3.1814204917	-4.3401836913	-2.1769016576
H	-2.1828575443	-4.8180656655	0.0501106564
Cl	-0.3704262604	-3.4394977948	1.6417764899
C	-1.0189430236	0.2799533648	2.5502065979
C	-1.1432993659	0.5461045467	3.9099633518
C	-0.086213814	0.2434907225	4.7643942527
C	1.0794284068	-0.3367025846	4.2712582104
C	1.1970564768	-0.6078027309	2.9101449558
Cl	-2.396386734	0.656019326	1.5335222224
H	-2.0617543765	0.9809848958	4.2872425322
H	-0.1760158323	0.4513442629	5.8259654763

H	1.8979301768	-0.5922234042	4.9344320686
Cl	2.6595547322	-1.4010447918	2.3870841947
H	-0.4027031015	0.6985075497	-0.2371842138

28

P	-0.1949520154	0.3160386748	-0.1313611402
C	-0.110918675	0.1353189221	1.7155580323
C	1.5845026394	0.165292763	-0.6425991112
C	-0.9210859346	-1.3053000698	-0.6746982432
C	2.1116393199	-1.0245701034	-1.1145374846
C	3.4457214222	-1.1143904036	-1.5775416756
C	4.2604861565	-0.0085532831	-1.5599109524
C	3.7753868472	1.2384433486	-1.0834798091
C	2.4183542997	1.3394989878	-0.6237587174
H	1.4930752552	-1.9158718547	-1.1329273647
H	3.819769848	-2.0675481088	-1.9417981195
H	5.2884817779	-0.0706613561	-1.9095304658
C	1.9676092834	2.6064819058	-0.1593946115
C	-1.368463647	-1.4481309908	-2.0362056238
C	-1.9914536034	-2.6776019221	-2.440270602
C	-2.1679163046	-3.7168331583	-1.4882775574
C	-1.7418434582	-3.5566280825	-0.1921796841
C	-1.1151572264	-2.3529025008	0.2089595535
C	-1.2145330871	-0.4286551809	-3.0164859586
H	-2.6457400094	-4.6409310532	-1.805417647
H	-1.8769307082	-4.3540191218	0.5337767031
H	-0.7795451235	-2.2570688888	1.2363186706
C	-1.2667309667	0.4769764607	2.5038796621
C	-1.1812988952	0.4008747284	3.9357440129
C	0.0409744888	0.0071699003	4.542570144
C	1.134482203	-0.3059016346	3.7721824185
C	1.05335551	-0.2448464001	2.3608545858
C	-2.5056603686	0.8820638633	1.933567617
H	0.0939994398	-0.0415377917	5.6277832748
H	2.0683309127	-0.6072501078	4.2392827328
H	1.9303446957	-0.5049405931	1.7769452969
C	-1.6420320514	-0.6104631715	-4.31326632
H	-0.757872665	0.5143234225	-2.7327703275
H	-1.5135859826	0.1871001512	-5.0401066511
C	-2.2492156557	-1.8256493924	-4.7066399962
C	-2.4201458972	-2.8333740674	-3.785969459
H	-2.5810659799	-1.9582345314	-5.7328479933
H	-2.8889424153	-3.7713262815	-4.0749631923
C	2.8017868041	3.7025811871	-0.14366547
H	0.942198726	2.7114399198	0.1808681409
H	2.4291002893	4.6586008258	0.2141569772
C	4.1387598123	3.5953400862	-0.5919875122
C	4.609943091	2.3880193889	-1.0531740428
H	4.7879685808	4.4665512761	-0.5735022973
H	5.6351707018	2.2931868353	-1.4038451088
C	-3.5926847613	1.1868185885	2.7228560275
H	-2.5907098054	0.9611294537	0.8542971199
H	-4.5256430085	1.4961392578	2.2593713332
C	-3.5042591451	1.1037314817	4.1319055608
C	-2.3214816312	0.7210929477	4.7208828777
H	-4.368849588	1.3453993803	4.7442020045

H	-2.2394384946	0.6572302927	5.8035763927

28H+

P	0.0059030593	-0.0120110799	0.0061167895
C	-0.0393557786	0.066537522	1.8079013791
C	1.688346684	0.0687647066	-0.6401319476
C	-0.9047121219	-1.4292003861	-0.6401495142
C	2.2207927055	-1.0798673517	-1.2086266764
C	3.5256050775	-1.0796495378	-1.743680944
C	4.2760567597	0.0736726189	-1.714712358
C	3.7692704538	1.2730740294	-1.1477433018
C	2.4483907718	1.2887277649	-0.5838669654
H	1.6311902083	-1.9900499986	-1.2550838965
H	3.9243160422	-1.9887230879	-2.1819294411
H	5.2794865026	0.0823106954	-2.1322165818
C	1.9771837435	2.5001958683	-0.012364475
C	-1.3293235759	-1.4768300151	-2.0136766335
C	-2.0711784904	-2.6310747852	-2.4383339027
C	-2.340584653	-3.67241734	-1.5111173365
C	-1.9110871045	-3.5999088443	-0.2056149878
C	-1.1938877884	-2.4679181501	0.2338423732
C	-1.0651141013	-0.4603040985	-2.9691732135
H	-2.900191819	-4.5386722561	-1.8541177797
H	-2.1254359608	-4.4019280705	0.4931393215
H	-0.8767769686	-2.4142683266	1.2706753082
C	-1.248348009	0.413215772	2.5059171619
C	-1.186975463	0.491415661	3.938691788
C	0.0354070714	0.2108805583	4.6049787067
C	1.1714324748	-0.1308263259	3.9071569518
C	1.1350260373	-0.1966961655	2.4989184942
C	-2.4896320816	0.6829041739	1.8714616231
H	0.0591924743	0.2718835467	5.6898462027
H	2.0991769147	-0.3417562726	4.4289144817
H	2.0423143403	-0.4471616641	1.9581613013
C	-1.5185650049	-0.5717799519	-4.2664592197
H	-0.4882715791	0.4178867668	-2.6944600588
H	-1.304879753	0.2188150078	-4.9795514396
C	-2.2546551187	-1.7053898248	-4.680113506
C	-2.5215794935	-2.7133093106	-3.7827018746
H	-2.6037880038	-1.7772763822	-5.7054353435
H	-3.0831606943	-3.5914678338	-4.0894824965
C	2.7607517592	3.6346950133	-0.0103349314
H	0.9933654771	2.5477263235	0.4452161562
H	2.3785855173	4.5501448224	0.4313850176
C	4.0574635255	3.6188375229	-0.5727557967
C	4.5494058415	2.4596124307	-1.1265926048
H	4.6611240166	4.520964563	-0.5623111739
H	5.5462425855	2.4335361588	-1.5581933904
C	-3.6032072058	1.0243124548	2.6093103939
H	-2.5837560769	0.6093482951	0.7920185653
H	-4.5418727018	1.2266820756	2.10219436
C	-3.5381437125	1.1094167596	4.0187790459
C	-2.3538112273	0.8451465767	4.6669363271
H	-4.4244626914	1.3794256069	4.584371377
H	-2.2925277243	0.9023776847	5.7502740853
H	-0.6568011405	1.1361430798	-0.4594614198

29

P	-0.5110652895	0.2519870489	0.0127588526
C	-0.374830924	-0.0585855215	1.8497064301
C	1.2955217215	0.0961899179	-0.4827914428
C	-1.1026537237	-1.4343850035	-0.5312009672
H	1.3025747077	-0.0314949908	-1.5733477641
C	2.1347543132	1.3224712852	-0.0928503014
H	1.7302353723	-0.817251412	-0.0531688077
H	-1.3789736043	-0.1733802105	2.272345624
H	0.2093803044	-0.9572628952	2.0841085158
H	0.0905700685	0.8018855493	2.3416600795
H	-1.1059402762	-1.4865824847	-1.6254498586
H	-0.4781836585	-2.2487691003	-0.1433972814
H	-2.1317780656	-1.5896792501	-0.1896789547
P	3.9411299991	1.1667741941	-0.5890875241
H	1.6998198618	2.235958912	-0.5221621519
H	2.1280565723	1.4499124092	0.9977303507
C	4.5337050842	2.8518936402	-0.0422950743
C	3.804590807	1.4806199933	-2.4254590841
H	4.8086967239	1.5949219227	-2.8483153381
H	3.3379145886	0.6215618968	-2.9186771186
H	3.2213745215	2.3804063174	-2.6580843094
H	5.5627158325	3.0073596369	-0.3840832494
H	3.9094198274	3.6672878454	-0.428270043
H	4.5375903769	2.9020444651	1.0520453583

29H^{+}

P	-0.5562866883	0.203737421	0.1174876841
C	-0.3615573645	-0.2355807863	1.918791552
C	1.2552271112	0.1048791316	-0.4376782493
C	-1.1307043746	-1.4332482102	-0.5556505479
H	1.232126412	-0.0101289231	-1.5277450662
C	2.0083353857	1.3907941492	-0.0510605733
H	1.7505972896	-0.7805316151	-0.0175795887
H	-1.3583207332	-0.3773969645	2.3494955861
H	0.2168661997	-1.1535727835	2.0782346138
H	0.1068523988	0.5869263664	2.470427933
H	-1.1657356367	-1.3958720655	-1.6493247694
H	-0.5002937093	-2.2739889325	-0.2437064367
H	-2.1507301185	-1.6156850506	-0.2022127025
P	3.7380763851	1.4483270892	-0.6396032115
H	1.5026005002	2.2790617475	-0.4519722367
H	2.0522121279	1.5161576201	1.0381669297
C	4.6093343893	2.9276002868	-0.0390560072
H	4.4054532505	0.3316963345	-0.1123700025
C	3.8502971881	1.3312396489	-2.4492928838
H	4.9001647082	1.3358997604	-2.7573043473
H	3.3820873602	0.4058508773	-2.7954097361
H	3.3392221287	2.1845962124	-2.9060669371
H	5.6521113162	2.9095518442	-0.3700670669
H	4.1242690994	3.8266459254	-0.4318418389
H	4.5823745461	2.9568438009	1.0543438756

30

C	0.063482612	0.0501056894	-0.0349998808

C	-0.0888335688	0.0345094412	1.4921970628
P	1.858282631	0.2883265536	-0.5419381214
H	-0.3461578447	-0.8714674426	-0.4674499843
H	-0.5019787242	0.8915217234	-0.4534742637
P	-1.8815621118	-0.2406075627	2.0073551036
H	0.4891504002	-0.7945671391	1.9158574646
H	0.3075703842	0.957242384	1.9318542852
C	1.7698688529	0.1476953949	-2.3876812051
C	-1.7234812752	-0.1004369973	3.8508084535
C	-2.5256409931	0.7531499281	4.6242783629
C	-2.4396238409	0.748618424	6.0181661211
C	-1.5520548302	-0.1088618959	6.6679694968
C	-0.7570834271	-0.9730442902	5.9121223485
C	-0.8490629317	-0.9747985685	4.5209150463
H	-3.2202854107	1.430943716	4.1380426031
H	-3.0688860331	1.4213856552	6.5956632837
H	-1.4839532377	-0.109304695	7.7525657186
H	-0.0665056636	-1.6519605264	6.4061067521
H	-0.2344546446	-1.6707728052	3.9548076684
C	2.9861306405	0.1821370996	-3.0941249169
C	3.0108650859	0.1376823613	-4.4860957855
C	1.8152120313	0.0762197734	-5.2070586605
C	0.6011917143	0.0552957873	-4.5223696934
C	0.5777447659	0.0880878162	-3.1249471652
H	3.9234593496	0.2420792424	-2.5452761998
H	3.9632591914	0.1583078602	-5.009704904
H	1.8318421094	0.0493569866	-6.2933669338
H	-0.3348754352	0.0098087939	-5.0734169029
H	-0.3815557635	0.0657862411	-2.6179208425
C	2.5585351289	-1.3690685677	-0.0788030946
C	-2.6394125525	1.3826124339	1.5275720719
C	-3.8188855775	1.3496730032	0.7685610549
C	-4.4325829659	2.5304957944	0.3423539928
C	-3.870675592	3.7643974577	0.6686581509
C	-2.6959261979	3.8144319316	1.4245899564
C	-2.0872706343	2.6344524637	1.8506655089
H	-4.2571499009	0.3889952105	0.5095077177
H	-5.34643913	2.4844512308	-0.244254752
H	-4.3440073209	4.6848526006	0.3369063584
H	-2.2549960246	4.7739599802	1.6825692166
H	-1.1810757415	2.6893396883	2.4482145622
C	2.2404390488	-2.5612523762	-0.7499592229
C	2.7825877936	-3.7774409336	-0.3361923634
C	3.6512678581	-3.8231406657	0.75783874
C	3.9774574019	-2.6469921528	1.4329262824
C	3.4374946771	-1.4284134232	1.0132791166
H	1.5737911185	-2.5348832448	-1.6078464007
H	2.5287743865	-4.6911397091	-0.8678121268
H	4.0745784312	-4.7716780893	1.0779988038
H	4.6569712843	-2.6744286155	2.2809441252
H	3.704618477	-0.5116609658	1.5336039901

30H^+

C	-0.5467746467	-0.3929330093	-0.4146086565
C	0.6991251623	0.4405293784	-0.0824388689
P	-2.016494877	0.0551655772	0.5820496213

H	-0.8295127299	-0.2909254033	-1.4699370256
H	-0.3625433095	-1.4606743974	-0.243296835
P	2.1362096443	-0.0825260792	-1.210984267
H	0.5117522006	1.5023580571	-0.2715988566
H	0.9699236227	0.3364779192	0.9745809826
C	-3.3750056631	-1.1030098385	0.3139800538
C	3.4540292876	1.0310836199	-0.5450595526
C	4.6515437251	0.5676038273	0.0192927056
C	5.6416861346	1.4679888757	0.4196503247
C	5.4520234959	2.840796419	0.2639915322
C	4.2699707958	3.3133889095	-0.3108479782
C	3.286194475	2.4161431108	-0.7223591979
H	4.8201883705	-0.496865241	0.144780048
H	6.5638357765	1.0911297625	0.8533814077
H	6.2235800469	3.5383108321	0.5769393321
H	4.119670294	4.3803894476	-0.4506471321
H	2.3869476978	2.8017227727	-1.1991390839
C	-4.644131319	-0.6427376014	-0.0696951873
C	-5.6754511936	-1.5596796583	-0.272487592
C	-5.446500059	-2.9241444553	-0.0894915108
C	-4.1837937014	-3.3828656686	0.2979630032
C	-3.1446401762	-2.4786163672	0.4993388853
H	-4.8282812381	0.4177246255	-0.2075214426
H	-6.6572783816	-1.2054685887	-0.5706106387
H	-6.2531730468	-3.633767547	-0.2466692865
H	-4.00880613	-4.4442623622	0.4432645911
H	-2.1684829287	-2.8479196965	0.803519136
C	-2.4938103778	1.7714335691	0.3454221315
C	2.5088349193	-1.7453745038	-0.4912017409
C	2.5931422675	-2.8247737495	-1.3852248067
C	2.8431412649	-4.1194176741	-0.9227278977
C	3.0083595048	-4.3520187774	0.4424597359
C	2.9270698857	-3.2875736376	1.3455232511
C	2.6805143723	-1.9954298966	0.8827213583
H	2.4680407718	-2.647559657	-2.4504656231
H	2.9124246803	-4.9417757543	-1.6291391594
H	3.2064055302	-5.3571264075	0.8041015458
H	3.066488603	-3.4642891731	2.4086931189
H	2.6485997221	-1.1744754828	1.5949493249
C	-2.6558840315	2.2893639403	-0.9522219887
C	-3.0309331982	3.620636343	-1.1187357812
C	-3.2429130865	4.4342191451	-0.0018674973
C	-3.0817665562	3.9214193739	1.2867660185
C	-2.7065848123	2.5908372244	1.466627868
H	-2.4922183101	1.6659422162	-1.827247249
H	-3.1544278282	4.0234733875	-2.1191297729
H	-3.5324784518	5.4718266975	-0.1378725457
H	-3.2438596404	4.5559872427	2.1523427735
H	-2.5786425019	2.1994413117	2.472164914
H	-1.6206460564	-0.0316979586	1.9274825105

31

P	-2.8485891037	-0.5104760709	-1.1785318101
C	-4.1698256685	-1.4374212013	-0.2671451578
C	-2.8759186729	1.1293816629	-0.3054003525
C	-1.2753499616	-1.2496563361	-0.465826989

H	-1.2664708053	-1.166050062	0.6291712414
H	-1.2838871327	-2.319303244	-0.7140088846
C	-0.0147866574	-0.5973810046	-1.0561186129
C	1.2739949673	-1.2140330302	-0.4888462014
H	-0.0274312271	-0.7057870396	-2.1477096504
H	-0.0237129689	0.4797943394	-0.84835951
P	2.8129489361	-0.4044547392	-1.2002439689
H	1.3050705497	-1.0606010025	0.5979793905
H	1.2855495714	-2.2973132931	-0.6688644263
C	4.1758661845	-1.3609486771	-0.3861720906
C	2.8168288933	-1.1334808934	-2.9093725519
C	-3.9361950918	-2.4632281407	0.6613112986
C	-5.0004197072	-3.1446515195	1.2590099169
C	-6.3165559779	-2.8090701448	0.9445108449
C	-6.5651686428	-1.790932606	0.0203191401
C	-5.5034850846	-1.1212234882	-0.5839200046
H	-2.9215173526	-2.7409549172	0.9291935168
H	-4.7961254239	-3.937151584	1.9747298462
H	-7.1430033889	-3.33875293	1.41087931
H	-7.5873794343	-1.5244838107	-0.2359873524
H	-5.7096922994	-0.3405906177	-1.3129520021
C	-3.1538367503	1.2787545399	1.0630030263
C	-3.135432544	2.539082891	1.6596480127
C	-2.8357681713	3.6723399634	0.8987401528
C	-2.5599474903	3.5392259184	-0.4620558622
C	-2.5863543171	2.2767134807	-1.0595692208
H	-3.3975810212	0.4050985055	1.6617398938
H	-3.3563212493	2.6383665263	2.7195474411
H	-2.8224891385	4.6540277112	1.3650910354
H	-2.3311630401	4.4167259463	-1.0613627161
H	-2.3852862567	2.1793531467	-2.1238223865
C	3.1240841223	-2.4761968246	-3.1827694849
C	3.0835214787	-2.9641288931	-4.488528037
C	2.7320111503	-2.1182459986	-5.5439842403
C	2.4270434158	-0.7815271004	-5.2875547142
C	2.4755148192	-0.2927338316	-3.9797848282
H	3.4080600062	-3.1401223917	-2.3705712954
H	3.3272891762	-4.0054606335	-4.6840311757
H	2.7013002766	-2.49978823	-6.5612626797
H	2.1582211664	-0.1163494236	-6.1041308511
H	2.2509625145	0.7535495678	-3.7863853713
C	5.4942610256	-0.9892770477	-0.7066323189
C	6.5848201329	-1.623759503	-0.1159815983
C	6.3812897279	-2.6351853642	0.8264541687
C	5.0808982724	-3.0058089488	1.1660057001
C	3.9875920144	-2.3769994159	0.5631153932
H	5.6655408887	-0.1929596267	-1.4276880378
H	7.5944414508	-1.323392962	-0.384391432
H	7.2303738782	-3.1258806525	1.2948015858
H	4.9116167019	-3.7897703513	1.9000300023
H	2.9859912589	-2.6894206477	0.8418158983

31H^+

P	2.7841408158	0.5835756747	-0.7003366226
C	3.1133797661	0.8273298357	1.1106353488
C	4.1479203002	-0.5438519056	-1.2307864682

C	1.3204855303	-0.6044294819	-0.5916651162
H	1.197972184	-1.0647147188	-1.5801979904
H	1.5141712984	-1.4082552957	0.1297651886
C	0.0421314119	0.1510786164	-0.2014218429
C	-1.1655091422	-0.8002709922	-0.0893764743
H	0.1980616911	0.6584436459	0.7593020505
H	-0.1665568876	0.9307068907	-0.9430250034
P	-2.7130814954	0.0471056145	0.4036056165
H	-1.3675934239	-1.3118398052	-1.0389559919
H	-0.9850247753	-1.5849348359	0.6558968274
C	-4.025393248	-1.1342633418	0.7778729111
C	-3.2034791377	1.3002756642	-0.7863720844
C	2.7551311349	2.0623899479	1.6744859402
C	2.9475197454	2.3098895984	3.0362145912
C	3.5142165755	1.3280194625	3.8491099815
C	3.8880657659	0.0994021784	3.2971781589
C	3.689021828	-0.1495484386	1.9394538742
H	2.3408582292	2.8416642665	1.0385178184
H	2.6712631101	3.2734970572	3.4560457389
H	3.6774582734	1.5220344805	4.9056460232
H	4.3436486236	-0.6620291093	3.9247472007
H	4.0025336862	-1.1005941717	1.5172118541
C	4.0891699691	-1.9465286772	-1.2370510085
C	5.1749341537	-2.7036323805	-1.6828574682
C	6.3384866328	-2.0717785694	-2.1224487009
C	6.4101277732	-0.6772230587	-2.1239228071
C	5.3210809181	0.0783926663	-1.6921725178
H	3.1975900636	-2.4648617398	-0.8962634564
H	5.110837513	-3.7885071921	-1.683617148
H	7.1825574689	-2.6621861943	-2.4674430652
H	7.3099889962	-0.1773518097	-2.4712451038
H	5.3794887422	1.164097052	-1.7162871314
C	-3.535730363	2.5887353618	-0.3350030086
C	-3.9231028924	3.5618548717	-1.2548666437
C	-3.9784500811	3.2553344566	-2.6160024852
C	-3.6476321148	1.9741475183	-3.0668105644
C	-3.2590352761	0.9929126336	-2.1576709989
H	-3.4901378975	2.8356014212	0.7221954508
H	-4.1765094158	4.5586645371	-0.9080300196
H	-4.2770842437	4.0175044134	-3.3293205041
H	-3.6879080472	1.7405131815	-4.1260549005
H	-3.0014914419	0.0008242338	-2.5188089184
C	-5.2612448937	-1.0654972534	0.1164339719
C	-6.2573222459	-1.9924894542	0.4235822456
C	-6.0268715654	-2.9765270816	1.3858806866
C	-4.7978333733	-3.0416672208	2.0496033284
C	-3.7938674155	-2.1253268192	1.7493271915
H	-5.4478754946	-0.2975001399	-0.6272231908
H	-7.2134485274	-1.9415729574	-0.0878308086
H	-6.8064626366	-3.6940452923	1.6232954561
H	-4.6224441552	-3.8047714106	2.8014027528
H	-2.8447840756	-2.1830046723	2.2766015315
H	-2.4104749339	0.7541047388	1.5795833053

32

C	-1.8840640358	-0.5998506549	0.0501639978

C	-0.6366685955	0.2169618835	0.4197363748
P	-3.3771765954	-0.1198934699	1.0823444521
H	-2.1011988284	-0.493379472	-1.0210067203
H	-1.6915767277	-1.6639478654	0.2411079391
C	0.5993529821	-0.1945683552	-0.3954848452
H	-0.8357329458	1.2854183254	0.2623129663
H	-0.4241322036	0.0960176799	1.4908456031
C	1.8467567588	0.622212806	-0.0258910917
H	0.7983995645	-1.2630292921	-0.2380800738
H	0.3868137666	-0.0736093455	-1.4665931914
P	3.3399183503	0.1422053116	-1.0579957331
H	1.6542973876	1.686308407	-0.2168674938
H	2.0638888884	0.5157753242	1.0452819988
C	4.6822203237	1.1898966648	-0.3266195929
C	-4.7193235024	-1.1681983513	0.3515701869
C	5.9857515312	1.0025739678	-0.8219132904
C	7.0469056894	1.782659785	-0.3684530121
C	6.8256092556	2.7841082961	0.5809414742
C	5.5376036487	2.9915625954	1.0718840598
C	4.4754680649	2.2000836254	0.6247928108
H	6.1694692381	0.234620065	-1.5701661166
H	8.0465266673	1.6144420979	-0.761008609
H	7.6506615257	3.3990476334	0.9305283444
H	5.3538569022	3.7690490538	1.8092981448
H	3.4841310816	2.3795888077	1.0285964956
C	-6.0225157476	-0.9821188735	0.848207123
C	-7.0835610494	-1.7625960424	0.3951559324
C	-6.8624466324	-2.7632183574	-0.5551485661
C	-5.5747515225	-2.9694546031	-1.0474269093
C	-4.5127490681	-2.1775677566	-0.6007522851
H	-6.2060386552	-0.2148621164	1.5972232309
H	-8.082935888	-1.5953552678	0.7887526296
H	-7.6873993233	-3.378474836	-0.9044140691
H	-5.3911632059	-3.74630943	-1.7855459325
H	-3.5216486109	-2.3561205445	-1.0055549776
C	3.7344435588	-1.5212609678	-0.3301236276
C	-3.7722144528	1.5432361905	0.3539759935
C	-4.2829839063	1.7234877372	-0.9418846393
C	-4.5349904731	3.0027103853	-1.4365906504
C	-4.2793589014	4.1251865018	-0.6441425683
C	-3.7750029733	3.9613061964	0.6459364188
C	-3.5285629128	2.6784857587	1.1416898917
H	-4.496082842	0.8570719811	-1.5624124391
H	-4.9329758466	3.1250130004	-2.4408914412
H	-4.4779376089	5.1216254398	-1.0302555206
H	-3.5797477934	4.8294306535	1.2703619481
H	-3.1483704864	2.554725133	2.1529691248
C	3.4910667766	-2.6561646561	-1.1184398555
C	3.7371033753	-3.9392090178	-0.6230918134
C	4.2407422691	-4.1036792672	0.6672013102
C	4.4960688009	-2.9815703336	1.4602540132
C	4.2444916379	-1.7021072815	0.9659301852
H	3.1113906701	-2.531919485	-2.1298523428
H	3.5420974629	-4.8070663291	-1.2479660659
H	4.4389823576	-5.1003078245	1.0529994263
H	4.8934802402	-3.1043250082	2.4647263688

H	4.4573745591	-0.8359755027	1.5869320292

32H+

C	-1.8263424031	-0.6562743206	-0.240640507
C	-0.5850733394	0.1622256287	0.1671050451
P	-3.3558650535	-0.1241618547	0.6150043562
H	-2.0168127248	-0.5781071366	-1.3182098225
H	-1.6762308434	-1.7191582444	-0.0159810986
C	0.6728315194	-0.3295927154	-0.5687902768
H	-0.7521263115	1.223053634	-0.0553008963
H	-0.4294312475	0.0844660039	1.2515252517
C	1.9226718045	0.4779633388	-0.1975404909
H	0.8372902147	-1.3899932963	-0.336094226
H	0.5063348563	-0.2701187624	-1.6528448014
P	3.4379168487	-0.0855741175	-1.1748061624
H	1.7733320611	1.5360020185	-0.4465823692
H	2.1067348183	0.4301613141	0.8829154901
C	4.6964816994	1.0862980198	-0.4840647957
C	-4.7673588825	-1.1660752202	0.1942752039
C	5.8053413745	0.6952769366	0.2801872697
C	6.7448423059	1.6381235554	0.704874021
C	6.5922327702	2.9846660056	0.3759217742
C	5.4991538103	3.3852851426	-0.3959810414
C	4.5685311089	2.4432817903	-0.8308112304
H	5.9437922677	-0.347806495	0.5453958667
H	7.5984686972	1.3150128371	1.2948478238
H	7.3241046994	3.7156701938	0.7079415736
H	5.3786496772	4.4298937128	-0.6706742247
H	3.740416727	2.7681689837	-1.4581169618
C	-6.0677279799	-0.6432253815	0.3085486001
C	-7.164287362	-1.4558857777	0.0286020007
C	-6.9720296311	-2.7824558593	-0.3642126157
C	-5.6812052771	-3.3037814507	-0.4778627418
C	-4.5759822518	-2.5021834709	-0.1993363828
H	-6.2242212574	0.3889942726	0.6059517122
H	-8.1679163261	-1.0514382187	0.1149931207
H	-7.8296633943	-3.4110928482	-0.5838680836
H	-5.5326554813	-4.3340521818	-0.7855523869
H	-3.5800691038	-2.9234571373	-0.2928875113
C	3.8120502781	-1.6998527753	-0.3478439363
C	-3.701636845	1.621563782	0.3612575236
C	-4.1122497086	2.0809079908	-0.9032563603
C	-4.3386934721	3.440887826	-1.0988564044
C	-4.1596096246	4.3408083226	-0.0435486405
C	-3.7546052743	3.8857651534	1.2124816113
C	-3.5232327254	2.5264532047	1.4208823843
H	-4.2646540222	1.386346209	-1.7251415461
H	-4.6565943156	3.7987844331	-2.0731012289
H	-4.3394923029	5.3998483618	-0.2018543163
H	-3.6204080601	4.5856280317	2.0312359771
H	-3.2099241779	2.1784419739	2.4013794905
C	4.1233176874	-2.7872562389	-1.179284491
C	4.4006742081	-4.0463592324	-0.6414973374
C	4.3650757899	-4.2375945062	0.7396589324
C	4.0554110394	-3.1657524716	1.5816047937
C	3.7824910769	-1.9086006459	1.0424402485

H	4.1501247225	-2.6433920487	-2.2565565743
H	4.6451120221	-4.8743567954	-1.3012474978
H	4.5817588574	-5.2154537825	1.1609835043
H	4.035292179	-3.3086240241	2.6589676112
H	3.5639183773	-1.0812389293	1.7130258333
H	-3.0772230997	-0.232307738	1.9891869407

33

C	-2.1895806151	3.0452555884	-1.5958390673
C	-2.9639635764	2.0363268935	-0.9953778851
C	-4.3494898486	2.0384927176	-1.213463434
C	-4.9425169728	3.0174322503	-2.0153939686
C	-4.1610510931	4.0039045772	-2.6169075111
C	-2.7796308568	4.012921466	-2.4070825743
P	-2.0674014864	0.8467627846	0.1102896582
C	-3.4502502381	-0.1429940975	0.8515835256
C	-3.918598586	0.2644958491	2.1116006142
C	-4.9541887792	-0.4208551414	2.7477662711
C	-5.5287481161	-1.5387423839	2.1400532984
C	-5.0652251168	-1.962471226	0.893001612
C	-4.0363980616	-1.2689406648	0.2521922347
C	-1.3343778245	-0.3418819495	-1.1191358798
C	-0.2530343949	-1.1557979898	-0.7069237505
C	0.3412746104	-2.0275931243	-1.6298108082
C	-0.1076280327	-2.0981227244	-2.9484959884
C	-1.16790252	-1.2925086796	-3.3593595694
C	-1.7714079678	-0.4227912134	-2.4500239863
C	0.2530360766	-1.1557925247	0.7069353676
C	-0.3412721434	-2.0275813334	1.6298289085
C	0.1076306199	-2.0981007826	2.9485145913
C	1.1679044414	-1.2924827944	3.359372161
C	1.7714090993	-0.4227715427	2.4500301118
C	1.3343788204	-0.3418725011	1.119141428
H	-0.3727783846	-2.7763581563	3.6487030879
P	2.0674014106	0.8467637607	-0.1102929403
C	3.4502511023	-0.1429973562	-0.8515794027
C	3.9185991817	0.2644837218	-2.1115994589
C	4.9541900303	-0.4208710367	-2.7477599888
C	5.5287503087	-1.5387532975	-2.1400387409
C	5.0652275867	-1.9624733659	-0.8929839703
C	4.0363998709	-1.2689389941	-0.2521797775
H	0.3727815855	-2.7763848691	-3.6486794417
C	2.9639623581	2.0363369101	0.9953658002
C	4.3494886213	2.038505642	1.2134513779
C	4.9425148073	3.0174516758	2.0153746703
C	4.1610479865	4.0039277342	2.6168808709
C	2.7796277484	4.0129417773	2.4070558238
C	2.189578434	3.0452693327	1.5958194756
H	1.5268884701	-1.3369025595	4.3844682502
H	2.591137952	0.2072092176	2.7813163195
H	-2.5911373428	0.2071863921	-2.7813148997
H	-1.5268864604	-1.3369363366	-4.3844553474
H	3.461221577	1.1218590666	-2.5998185771
H	5.3044333705	-0.0884304909	-3.7216134481
H	6.3294806918	-2.0799067334	-2.6374663889
H	5.5065237502	-2.8338375082	-0.4153874366

H	3.6857651	-1.6066322592	0.7186334824
H	4.9707399482	1.2750384093	0.7555552298
H	6.0185340558	3.0046255693	2.1708951796
H	4.6242296104	4.7639532115	3.2407319056
H	2.1625603667	4.7803214268	2.8675638302
H	1.1152518119	3.0692458888	1.4262483639
H	-3.4612217039	1.1218751952	2.599813383
H	-5.3044323397	-0.0884077328	3.7216173081
H	-6.3294779854	-2.079892863	2.6374849907
H	-5.5065205506	-2.8338392724	0.4154115275
H	-3.6857630719	-1.6066407663	-0.718618568
H	-4.9707404488	1.2750283015	-0.7555616042
H	-6.0185362141	3.0046039848	-2.1709143488
H	-4.6242334461	4.7639249951	-3.2407641681
H	-2.1625642057	4.7802982759	-2.8675962912
H	-1.1152540101	3.0692344065	-1.4262681668
H	1.1725225119	-2.6476232122	-1.3059858619
H	-1.1725195223	-2.6476145386	1.3060085912

33H+

C	1.4343631101	3.1145131543	1.3216793765
C	2.4635093836	2.3463008048	0.7471827563
C	3.7890224915	2.8048382371	0.7623371026
C	4.0837909168	4.0322368144	1.3570207698
C	3.0644170677	4.7969576558	1.9260196118
C	1.7429722743	4.3396360406	1.9072888563
P	2.032148325	0.7676433262	-0.0193845038
C	3.4310486768	0.0609430108	-0.9142537147
C	3.6874447085	0.493440347	-2.2263561325
C	4.7831531916	-0.0180303009	-2.9183992092
C	5.6196781914	-0.956728897	-2.3085482778
C	5.3627750679	-1.3899924959	-1.0058891638
C	4.268121934	-0.8862830314	-0.3042254065
C	1.334814673	-0.3686574053	1.1941435949
C	0.3390121866	-1.3001501996	0.8180276924
C	-0.1296750432	-2.1809323407	1.8002776686
C	0.3688264525	-2.1474164585	3.1037117528
C	1.3467579914	-1.2197663566	3.4620954386
C	1.8259334448	-0.3236281191	2.5090179325
C	-0.1893892368	-1.3992203988	-0.583181985
C	0.3437365068	-2.392771884	-1.4163722446
C	-0.1123196355	-2.554056851	-2.7253544745
C	-1.1154671203	-1.7174412405	-3.2097073745
C	-1.661252535	-0.7303762381	-2.3863948596
C	-1.2222053147	-0.5542306563	-1.0653841948
H	0.3135098011	-3.3286248754	-3.3563513143
P	-1.8710108696	0.8064753809	0.0298006307
C	-3.0192900356	-0.0981857959	1.1594990245
C	-3.2955156446	0.4908423726	2.4036554327
C	-4.1688356312	-0.1214405958	3.3032977785
C	-4.7678735923	-1.3393419489	2.9765789922
C	-4.4941039539	-1.939972474	1.7456973922
C	-3.6271462969	-1.3238218041	0.8412887978
H	-0.0150220171	-2.8451011142	3.8420887771
C	-2.9978484112	1.7530413497	-1.0863460016
C	-4.3505882756	1.4394806349	-1.2934081773

C	-5.1374387003	2.2250255478	-2.1370662069
C	-4.5855366286	3.3308241936	-2.787035052
C	-3.2430633392	3.6563090208	-2.5848437233
C	-2.4591881859	2.8780037853	-1.7331335475
H	-1.4826578801	-1.8339502975	-4.2254918132
H	-2.4476676565	-0.0916962092	-2.7746899075
H	2.5711986578	0.4145215846	2.7907555474
H	1.7289496117	-1.1855256679	4.4774964382
H	-2.8249890518	1.4343164626	2.6699129398
H	-4.3771767147	0.3496282336	4.2598904923
H	-5.4448187986	-1.8191315577	3.6777865824
H	-4.9606650595	-2.886410226	1.4858563949
H	-3.4253275915	-1.7975874156	-0.1153751222
H	-4.7939696377	0.5867347558	-0.7887796036
H	-6.1840099202	1.9733858631	-2.2847853957
H	-5.2014499739	3.9414017599	-3.4411751345
H	-2.8108862635	4.5219944657	-3.0791176479
H	-1.4205158463	3.1526787818	-1.5611565687
H	3.0368280597	1.2183577757	-2.70875686
H	4.9800896909	0.3113267691	-3.9339191511
H	6.4709149513	-1.3550925182	-2.8525445248
H	6.0104248682	-2.1244198406	-0.5370556774
H	4.0630908897	-1.2349453103	0.7036073027
H	4.5823390375	2.2153852858	0.3137194235
H	5.1087649576	4.3897566297	1.3717081495
H	3.2982479013	5.7532494664	2.3845423159
H	0.9521438047	4.938739682	2.3482692924
H	0.4072814734	2.7573012334	1.3061079926
H	-0.9044056817	-2.8927375752	1.534733926
H	1.1227610862	-3.0444984371	-1.0296882629
H	1.0480711582	1.0168201116	-0.9821359427

34

C	-0.4853010877	0.4043323189	-0.0147717629
C	-0.6178270179	0.1063529237	1.4553530516
C	0.7738274292	0.8885802822	-0.5111429336
C	0.9233480054	1.1632251837	-1.9102399419
C	-0.181670222	0.9503433214	-2.7722635802
C	-1.3757700881	0.4854828049	-2.2795408725
C	-1.5455757585	0.193015543	-0.8966720724
C	1.8947579651	1.1132101937	0.337860718
C	2.1681337394	1.6414796987	-2.3991826729
H	-0.0707163687	1.1633796682	-3.8332171662
H	-2.2140131411	0.3436891957	-2.9541148623
P	-3.1781105832	-0.4065490157	-0.2306274448
C	-1.1275099378	1.0528112859	2.3447138889
C	-1.2325439728	0.7301451822	3.7272982032
C	-0.8210694171	-0.4864458832	4.2124182017
C	-0.2837734149	-1.4683787104	3.3425903967
C	-0.1795790063	-1.1726223357	1.9436894634
P	-1.717827679	2.6932810087	1.6892211692
H	-1.6550833464	1.4623370993	4.4078572578
H	-0.9052819833	-0.7119098362	5.2732633596
C	0.1498246196	-2.732468189	3.8235818337
C	0.3589829125	-2.174342318	1.0868050473
C	0.6674555651	-3.6754517275	2.9660704483

C	0.7705532288	-3.3905320735	1.5839013555
H	0.4413644237	-1.9711885859	0.0250265589
H	1.1762330407	-4.1392992489	0.9090555267
H	0.9964184825	-4.6391558993	3.345626315
H	0.0634209979	-2.940391491	4.8876209496
C	3.0885238284	1.5777614477	-0.1668414303
C	3.2305503965	1.8445652502	-1.5491752712
H	2.2641956372	1.8449674315	-3.4632511209
H	4.1784132933	2.2104491186	-1.9347785354
H	3.9286628092	1.7431356382	0.5020856331
H	1.7997652504	0.9158797758	1.3996840064
C	-4.3575836271	0.2578638697	-1.4992465242
C	-3.1398140013	-2.2210070414	-0.6130885307
C	-2.9735939444	3.168707042	2.9694397834
C	-0.2968361492	3.8244742876	2.0649502999
C	-3.9283842301	-3.0520196492	0.1988971052
C	-3.9706745875	-4.4310609339	-0.0121682165
C	-3.2102786459	-5.0043000303	-1.0322261801
C	-2.4121295127	-4.1917152222	-1.840332795
C	-2.3783344777	-2.8116629083	-1.6344103724
H	-4.5072871911	-2.6132806634	1.0080826764
H	-4.5885805817	-5.0571037636	0.6263499382
H	-3.2345583828	-6.0788903676	-1.1938770735
H	-1.8147347397	-4.6326479356	-2.6345679801
H	-1.7526941007	-2.1920723108	-2.2700018064
C	-5.0709503885	-0.5332262238	-2.4112868254
C	-6.0045396812	0.0443080966	-3.2765271051
C	-6.2353577517	1.4192918437	-3.2482556644
C	-5.5312938168	2.2177774507	-2.3427278691
C	-4.6094859576	1.6420693684	-1.4705130267
H	-4.8993165998	-1.6044415236	-2.4498301009
H	-6.5487347041	-0.5855134792	-3.9761122983
H	-6.9609955716	1.8665256527	-3.9226527475
H	-5.7062932908	3.2902001727	-2.3101127301
H	-4.0744570029	2.2713947831	-0.7621632234
C	-0.1700122145	4.9654275649	1.2563078606
C	0.8636419437	5.8803139801	1.4627721729
C	1.7988607462	5.6595663347	2.4746846611
C	1.6923021638	4.5230711544	3.2792889052
C	0.6524634238	3.6140434282	3.0780453963
H	-0.8840475401	5.1321319192	0.4533342262
H	0.943695634	6.7582592335	0.8269684047
H	2.6098506798	6.3658210019	2.6326840198
H	2.419751739	4.3436232692	4.0671514656
H	0.5822983535	2.7343095816	3.7108456732
C	-2.8160201808	4.2200913735	3.8839330426
C	-3.8507682123	4.5639389799	4.7584826535
C	-5.054613581	3.8604033644	4.7371698003
C	-5.2237648002	2.8113971582	3.8292952149
C	-4.1980215669	2.4754459051	2.9478654239
H	-1.8832484165	4.7744221093	3.9171151725
H	-3.7103188171	5.3828486922	5.4598410355
H	-5.857592986	4.12872919	5.4187889674
H	-6.1595633689	2.2588401098	3.8020735527
H	-4.3440231258	1.6639046828	2.2378299006

34H+

C	-0.4508258731	0.4420608689	0.0180586908
C	-0.5764851625	0.0041547178	1.4525254196
C	0.8227857384	0.933289453	-0.4320600001
C	0.9649216029	1.3616973	-1.793631356
C	-0.1565422134	1.2839300236	-2.6565091552
C	-1.3635576926	0.8052141804	-2.2071374877
C	-1.534832161	0.3657397648	-0.8640327601
C	1.9635883915	1.0146262713	0.4178359424
C	2.2198677116	1.8497354956	-2.2462301658
H	-0.0481929634	1.6066577814	-3.6891663763
H	-2.2069067869	0.7593006392	-2.8875116164
P	-3.1976027013	-0.2193838856	-0.2429634167
C	-1.0574208942	0.8666482684	2.4409017643
C	-1.1668137503	0.460208729	3.8012402247
C	-0.7699528507	-0.799037899	4.1742093282
C	-0.2589693531	-1.7123960828	3.2167862139
C	-0.1643976137	-1.312920552	1.8400099929
P	-1.555250153	2.550259436	2.0412573612
H	-1.5788613793	1.1395670809	4.5412649552
H	-0.8505288332	-1.1116507617	5.2116506683
C	0.1598930395	-3.0153965268	3.5927140601
C	0.351521698	-2.2471170914	0.8987236605
C	0.6557002978	-3.8914063406	2.6548145342
C	0.7509026678	-3.5019600315	1.2972849663
H	0.4250652247	-1.959775038	-0.1433102056
H	1.1412905301	-4.2017351206	0.5646245692
H	0.9758437149	-4.8853125328	2.9532581043
H	0.0832066107	-3.3078147645	4.6364878464
C	3.1668822193	1.4898958702	-0.0532726275
C	3.300223884	1.9131384356	-1.3973584994
H	2.3108116633	2.1680319313	-3.2815630435
H	4.2570137672	2.2821027623	-1.7549292364
H	4.0252989268	1.5353311371	0.6109178304
H	1.8848942814	0.6863222477	1.4486797295
C	-4.355388516	0.3600612643	-1.5648180675
C	-3.1149940853	-2.0465428917	-0.4981949755
C	-3.0147136042	3.0275164348	2.9984881207
C	-0.2246709734	3.754746912	2.23838209
C	-3.8861098925	-2.8468814738	0.3592485093
C	-3.9037703468	-4.2348611985	0.2144754473
C	-3.1412332929	-4.8422209865	-0.7841033047
C	-2.3625416179	-4.0575285704	-1.6380707112
C	-2.3485600216	-2.669262117	-1.4972666236
H	-4.4754041433	-2.3803640627	1.1448618518
H	-4.509173902	-4.8403588722	0.8831984642
H	-3.1521312097	-5.9227049952	-0.8967867093
H	-1.7686255069	-4.5265054546	-2.4180616437
H	-1.7401846309	-2.0699518189	-2.1689921343
C	-4.7762296192	-0.419303174	-2.6535453916
C	-5.683559316	0.0925693112	-3.5835347834
C	-6.1815738183	1.3887290215	-3.4430673666
C	-5.7772725844	2.1719631215	-2.3599690276
C	-4.8808088154	1.6564127186	-1.4247744619
H	-4.4026152791	-1.4311166872	-2.774231045
H	-6.0021316108	-0.5252721785	-4.4186270327

H	-6.8883700708	1.7831207927	-4.1675838779
H	-6.1702662073	3.1775110887	-2.2368591685
H	-4.5952359898	2.2657851728	-0.5692803218
C	-0.1610915151	4.8515067953	1.3635915016
C	0.8385092674	5.8084990516	1.5297020853
C	1.7709509255	5.6737136441	2.5609159181
C	1.7112049443	4.5799860062	3.4281690088
C	0.7172207564	3.6159300186	3.2699397187
H	-0.8797350593	4.9580466047	0.555179944
H	0.8921331715	6.6543228553	0.8512556225
H	2.549599178	6.4203465964	2.6856355526
H	2.4416490004	4.4742527181	4.2244003734
H	0.6819935778	2.7591482493	3.9364960308
C	-3.0274857646	4.2115986232	3.750529902
C	-4.1778364909	4.565220008	4.4565334005
C	-5.3075350339	3.7472481529	4.40888558
C	-5.2944506324	2.5685828677	3.6567820775
C	-4.1517733587	2.2002412537	2.9507968617
H	-2.1519783066	4.8518100565	3.7869713698
H	-4.1890553516	5.4797247123	5.041552412
H	-6.2013475683	4.0274410937	4.9585011094
H	-6.1752526952	1.9347695973	3.620291078
H	-4.1409243522	1.2838246201	2.3656769171
H	-1.8876097886	2.5891849074	0.6827072306

35

C	0.012457908	-0.1055925096	0.0642808285
N	-0.0282851409	0.0350336099	1.5232286092
C	1.3154951357	-0.0412633534	2.1051307837
P	-1.3141246799	-0.7460284358	2.3352589746
N	-2.6510729035	-0.0586349114	1.5301821436
C	-2.8416210113	1.3816532732	1.4286200551
N	-1.4953441542	-2.2934254862	2.3465239963
P	-0.3405770935	-3.5436877501	2.1132007681
N	-1.3292262568	-4.9453462652	2.2274530041
P	-2.3118919004	-5.5605475605	1.1858407839
N	-2.1867389759	-7.2574083227	1.2748208107
C	-3.0438552961	-8.1414111319	0.496880733
N	0.4526256657	-3.560798855	3.6365863487
P	1.6191711877	-4.4536965004	4.1556220439
N	2.6804534112	-3.4751959898	5.0613776754
C	3.8642750114	-4.0016718319	5.7262641107
N	-1.1112245834	-0.1153826656	3.9057225243
C	-1.5695135954	-0.9055412676	5.0465195524
N	1.04993104	-5.7881827754	5.0529681345
C	-0.2918649998	-5.7203176379	5.6290072745
N	2.7299575251	-5.23149875	3.1134865706
C	2.2830207548	-6.4107260983	2.3655345831
N	-3.913766135	-5.0207883728	1.4156109574
C	-4.3026738456	-4.5389292139	2.7395837281
N	-2.1511512644	-5.3211983021	-0.499754981
C	-2.4750264526	-4.0009667291	-1.049980344
C	-1.022990207	1.3122473447	4.187035918
C	1.9411927364	-6.6932236553	5.7685004957
C	-5.0386946036	-5.5075336202	0.6259824058
C	3.5659111527	-4.3592176964	2.2820114812

C	-0.9621151292	-5.8885853433	-1.1420725609
C	-3.8886057543	-0.8201616359	1.4072766054
C	2.2029278503	-2.2184849111	5.6255997332
C	-1.6355590939	-7.883758432	2.4706092075
H	-3.6601387077	-1.8849663889	1.4430998383
H	1.3438383101	-1.8716088533	5.0517436895
H	-0.9870307812	-7.172997628	2.9822096005
H	-2.5985104608	-0.6444904781	5.3462932748
H	-0.2840953495	-5.3127416981	6.6541485682
H	-4.7245768015	-5.3426422308	3.3666152701
H	-0.9116313051	-0.7133992866	5.9051754799
H	-0.7154054604	-6.7328763573	5.6731233568
H	-5.0714630389	-3.7623144127	2.6285845125
H	-1.5195538126	-1.9646681948	4.796173567
H	-0.9259035583	-5.1005879776	4.995972844
H	-3.435538946	-4.1049702064	3.236176411
H	-0.3585205358	1.4756016657	5.0471682216
H	1.4907180246	-7.6947284881	5.8066774136
H	-5.7865368308	-4.708736591	0.5238756615
H	-2.0011070939	1.7555036826	4.4362015845
H	2.1261590092	-6.3738566321	6.8075933613
H	-5.5403310052	-6.3721973407	1.0911600276
H	-0.6048915762	1.8454836745	3.3316303368
H	2.8990338217	-6.7740068853	5.2517152669
H	-4.7085631006	-5.7915064369	-0.374665619
H	-4.6071858361	-0.5799403883	2.2091350866
H	1.9111460299	-2.3228725768	6.6842813116
H	-2.4247019203	-8.2150309196	3.1665227911
H	-4.3684179873	-0.5827216495	0.4468852671
H	3.0028038748	-1.4656520093	5.5732749183
H	-1.050796649	-8.7696291445	2.1833985819
H	-3.4471823784	1.7926402117	2.2536055238
H	3.6778725506	-4.2718978482	6.7788191139
H	-3.9458530975	-8.4543849812	1.0486308675
H	-3.3635139245	1.6133096598	0.4897149031
H	4.6557976692	-3.2390101307	5.7174994872
H	-2.4861568702	-9.0513239319	0.2341530481
H	-1.8773656961	1.8922736012	1.4110716743
H	4.2373665701	-4.8800209498	5.1972316542
H	-3.3503694959	-7.655452899	-0.4308429077
H	-0.9974513162	-0.049376257	-0.3442653304
H	3.8941056193	-3.4931888484	2.858641814
H	-0.7378436892	-6.8673025309	-0.7159868018
H	0.6105255667	0.7117488553	-0.3597661992
H	4.451585313	-4.9179741136	1.9524230651
H	-1.1560777663	-6.007953165	-2.216155519
H	0.4643823552	-1.061605938	-0.2418961929
H	3.0256908825	-4.005102502	1.3907246439
H	-0.081063604	-5.2398509002	-1.019727433
H	1.8157981897	-0.9909139884	1.86415067
H	1.720810574	-6.1355999302	1.4609785045
H	-1.6367998704	-3.2966793187	-0.9436849019
H	1.9235085017	0.7825113708	1.7079269011
H	3.16204064	-6.9957969704	2.0635170736
H	-2.7078292844	-4.105485379	-2.1182687752
H	1.2626456807	0.0508991318	3.1896694402

H	1.642222272	-7.0326392575	2.9897414768
H	-3.3411403032	-3.5806573412	-0.5393883878

35H^{+}

C	-0.0817163679	-0.0710083551	0.0354756231
N	-0.0848456341	-0.0423010734	1.5027030334
P	1.3696375676	-0.1674016181	2.3421474987
N	0.8410283512	-0.4963859386	3.9114826482
C	-0.1020990392	-1.5854156523	4.1913426111
C	-1.1877082353	0.7631385799	2.0410760874
N	2.130783594	-1.4552139001	1.571895352
C	3.5881207215	-1.5198978083	1.4519764689
N	2.4142252768	1.0241315928	2.3346971632
P	2.3137986683	2.6384025593	2.3779392265
N	1.4973605326	3.15197641	3.6773308298
P	1.1012029249	4.5711274478	4.2630556099
N	0.6056656132	5.817544053	3.2436207221
C	-0.7013404865	5.734239288	2.5812367907
C	1.4806660511	-2.7634209724	1.4676643618
N	3.7718482809	3.3314249294	2.2729971185
P	4.9744592147	3.2642263728	1.2436336924
N	4.6901074367	3.1292805627	-0.4105590508
C	4.1158849178	4.2666165619	-1.1394223816
N	5.7659582505	4.7449222818	1.3576586183
C	5.6525005677	5.5735978185	2.5590218801
N	5.9481614536	1.9188074079	1.5478562545
C	7.1717474196	1.6736265412	0.7760279795
C	6.9705348283	5.0265269595	0.5729656456
N	-0.259228949	4.3120974525	5.2164155648
C	-0.6006675825	2.9820889116	5.7225731533
N	2.4055534396	5.2777764343	5.0703349562
C	2.2830978215	6.5814477625	5.7326953831
C	-0.8638412098	5.4005274472	5.9875526643
C	1.7312767087	-0.2179500589	5.043564745
C	3.4066188559	4.4148706799	5.7070305593
C	1.5864390646	6.5604802667	2.4434355095
C	6.0192525841	1.3722620502	2.9074488404
C	4.3102622466	1.8413903711	-1.0031022415
H	4.0050336929	-0.5147113354	1.4941312706
H	-0.1515327429	2.2211190301	5.0861867236
H	4.718662067	5.3501571959	3.0730151368
H	2.3800543351	-1.0755491803	5.2774304438
H	3.1235403639	4.1449296049	6.7356586288
H	6.7937874361	1.8681854619	3.5119253501
H	1.1205244574	-0.0043580779	5.9283994624
H	4.3601879532	4.9537128695	5.7484151899
H	6.2698543425	0.3070111739	2.8449504886
H	2.3458583509	0.6540252158	4.828327929
H	3.5466587962	3.5097913393	5.119127654
H	5.0545363645	1.4726336713	3.4015233353
H	-0.7149277541	-1.3139751	5.0592554464
H	3.2529242834	7.0921134628	5.6985244762
H	7.3458873147	0.5927283997	0.715705765
H	0.4144081669	-2.5278899971	4.4240116607
H	1.9905500706	6.4822930043	6.7880608245
H	8.0546692444	2.1325970332	1.2442743951

H	-0.7667066735	-1.7489723492	3.3422570073
H	1.5501103423	7.2062771326	5.2213049212
H	7.0713436181	2.0594212266	-0.2390730355
H	4.0354999887	-2.1306318657	2.2506541966
H	-0.2523359339	2.8400504227	6.7566448062
H	6.4937672615	5.4074413777	3.2489054491
H	3.8475611912	-1.9797154571	0.4898690251
H	-1.6918031147	2.8642366353	5.7141579463
H	5.6602716936	6.6311375987	2.2659821004
H	1.7752329907	-3.437690715	2.284974428
H	-0.5065531185	5.4165439099	7.0274120423
H	7.8898844044	4.8150751971	1.1379795631
H	1.7708543029	-3.2343912627	0.5207400235
H	-1.9522599493	5.2666293118	6.0071849971
H	6.9802999854	6.088473044	0.2989344302
H	0.3951826185	-2.6559533874	1.4720826825
H	-0.6514439368	6.3659445994	5.526415238
H	6.9783871471	4.4402192142	-0.3467100793
H	0.7485204623	-0.6744285715	-0.3310536943
H	-1.408837464	5.1868504526	3.2035800953
H	4.4135640329	5.205985762	-0.673988379
H	-1.0211217546	-0.5118651278	-0.3178480534
H	-1.0860908336	6.7476023579	2.4181594331
H	4.4809901138	4.2538593426	-2.172967993
H	0.0010576191	0.9409273302	-0.3885126744
H	-0.6295787311	5.2350559308	1.603233878
H	3.0170568892	4.2161599343	-1.1646376931
H	-1.1510528455	1.7973226601	1.6682371281
H	1.7378669478	6.0973191339	1.457549939
H	3.2175435338	1.7234225269	-1.0437546583
H	-2.141787965	0.3216746587	1.7295245568
H	1.2219125527	7.5828322476	2.2885975378
H	4.6978374684	1.7883309563	-2.0274417464
H	-1.1473094694	0.786080706	3.1292233624
H	2.5458811725	6.6017519958	2.9565849728
H	4.7256817349	1.0176067698	-0.4245668757
H	1.5762279003	3.0421064379	1.2347843505

36

C	0.0659856829	-0.7044759163	-0.1252520355
C	0.066881982	-0.7472135283	1.4156154218
N	1.4669397223	-0.4827865519	1.7904863962
C	2.3259581051	-0.3434237872	0.5987998446
C	1.5160328792	-1.0505342193	-0.4939191131
P	1.9635359686	-0.096929189	3.3619716967
N	0.9414274194	1.0852813416	4.0113176875
C	-0.4296217208	0.8885791841	4.5179469791
C	-1.1711942937	2.168145181	4.1075192019
C	-0.0665037531	3.231571407	4.1924565843
C	1.1679080139	2.4982194868	3.643443696
N	3.4301016698	0.4087494556	3.4740467862
P	4.9698693704	0.1154939702	2.771161829
N	5.0057015487	-1.6012751295	2.6617946385
P	6.0987487779	-2.7084366683	2.5714827025
N	7.3719998319	-2.3813308585	1.5091846976
C	7.6310057229	-1.0462579093	0.9293904406

C	8.9562784766	-1.2330725978	0.1737487846
C	8.921061004	-2.7096863168	-0.2479372449
C	8.2904157393	-3.3957834691	0.9736979114
N	6.0455056911	0.4382571306	4.0765727549
P	6.2373147989	1.551530064	5.1482715604
N	5.9205592223	3.125112538	4.6110772899
C	5.432235215	3.4524476187	3.2556111096
C	5.141295033	4.9569345178	3.3387251168
C	6.2106501841	5.4651395215	4.3166078147
C	6.2933262047	4.3370845983	5.3589431051
N	5.3836138622	1.5232485142	6.627175779
C	3.9737999298	1.9916448002	6.6386649567
C	3.395189873	1.4752832857	7.984802947
C	4.5433643227	0.677041331	8.6410351565
C	5.4321843356	0.305048945	7.4510598314
N	7.8263294245	1.4776239319	5.7275307475
C	8.3195639221	1.957480626	7.0328849383
C	9.7349296415	2.472642719	6.7356494864
C	10.1977812628	1.5440012302	5.6034544375
C	8.929844815	1.3781178188	4.7518408645
N	6.8812047402	-3.226668294	3.9919666189
C	8.2353460819	-2.7532854914	4.3643332536
C	8.14726408	-2.4404581826	5.8653387565
C	7.0599938669	-3.4051852332	6.3573629686
C	6.0466746389	-3.3795095023	5.2063634615
N	5.3866520716	-4.1481748247	2.0590570352
C	5.7487178562	-5.512967564	2.4770556515
C	5.2010862382	-6.4002852496	1.3476456173
C	3.9736373747	-5.6181837332	0.8565292501
C	4.4441925305	-4.1577369642	0.9278243026
N	1.4246563341	-1.4684786508	4.2222761631
C	1.7939612467	-2.8092245028	3.6861660174
C	1.7720707058	-3.7549183945	4.9136625065
C	1.1525145464	-2.9173935544	6.0460437689
C	1.5985778301	-1.4993804406	5.6857791245
H	3.4122870716	1.5793814425	5.791243484
H	3.9495893588	3.0831314711	6.5686370806
H	3.0500884054	2.2934324923	8.6252228996
H	2.5326187481	0.8275376017	7.7963158525
H	4.1933655747	-0.1991367923	9.1972630819
H	5.1070214277	1.313336372	9.332752135
H	6.4596965112	0.0661577371	7.7365869539
H	5.0193014685	-0.5638947603	6.9119343332
H	8.3699154098	1.1291389891	7.7576441254
H	7.6624081826	2.7176226895	7.4619375194
H	8.8632655216	2.1770695774	3.9968545177
H	8.8921615179	0.4231850778	4.2205506327
H	4.546780382	2.8642454104	3.0035566133
H	6.1948124169	3.2426778047	2.4918501481
H	7.3000485868	4.2532505067	5.7845672191
H	5.5995003807	4.5202375874	6.1943173156
H	10.3852367768	2.4472489667	7.6165163488
H	9.6936002175	3.5096850732	6.3777056859
H	11.0386936727	1.9428011338	5.0269459695
H	10.5038624488	0.5747041469	6.0166752865
H	5.1824485281	5.4445965469	2.359307741

H	4.1401260276	5.1242816716	3.7562130478
H	5.9696455985	6.4338955959	4.7665719355
H	7.1721454033	5.5667256631	3.7980450045
H	8.5298924336	-1.877941663	3.7806438692
H	8.9745587358	-3.5464448716	4.1735939059
H	9.1076879437	-2.5724096864	6.3760861242
H	7.8195075544	-1.404367118	6.0009160844
H	6.6108145746	-3.105645287	7.3101851103
H	7.471452462	-4.4152349873	6.4813018385
H	5.4488741998	-4.2980029599	5.1569950175
H	5.3584965325	-2.5293447509	5.3053132782
H	5.2696903002	-5.7741470858	3.4334624637
H	6.8276601398	-5.626271818	2.6168116286
H	4.9444898969	-3.8615280697	-0.0084632107
H	3.6260442135	-3.4525005448	1.1001491337
H	7.6945585444	-0.2764789163	1.7046338734
H	6.820968712	-0.7507337677	0.2497658986
H	7.7515621427	-4.3119213962	0.7059293717
H	9.0662638473	-3.6704233639	1.7070343617
H	4.9662401264	-7.413913863	1.689088072
H	5.94015429	-6.4817055149	0.5400966938
H	3.6466877992	-5.9057038647	-0.1480599856
H	3.130680174	-5.774046961	1.5413822747
H	9.0542109578	-0.5426792902	-0.6702231977
H	9.8040352308	-1.0586556808	0.8485496911
H	9.9064118958	-3.1170477609	-0.4971801421
H	8.2715830682	-2.8356813074	-1.1229847069
H	2.7881347402	-2.7953373855	3.2259189035
H	1.0629206723	-3.1092874819	2.9271820116
H	1.2160618522	-4.6774518561	4.7170124286
H	2.7960404824	-4.040730073	5.1788832581
H	1.4856975194	-3.2264119228	7.0426534729
H	0.0579229476	-2.9779342865	6.0186798144
H	0.9974125071	-0.723285591	6.1667028242
H	2.6507216718	-1.3405869504	5.9740384279
H	-0.4290123376	0.7975104554	5.6162224037
H	-0.8818041684	-0.0237400228	4.1221665032
H	1.2342796692	2.6159430698	2.5510007972
H	2.1062264445	2.8656914853	4.0686073627
H	3.3031242647	-0.8007158943	0.7671491867
H	2.4914260296	0.7130269723	0.3374690777
H	-0.6147486885	0.0042168719	1.8332560917
H	-0.2616883268	-1.728534086	1.7883817358
H	-2.0327845007	2.3797188079	4.7497437624
H	-1.5332243283	2.0821657214	3.074660035
H	-0.2929805115	4.1437129336	3.6309439603
H	0.0976277118	3.5156784559	5.2395076555
H	1.7953868086	-0.7245076029	-1.50120525
H	1.6741225152	-2.1347213199	-0.4327229632
H	-0.6722114689	-1.3863475133	-0.5600340204
H	-0.1733867804	0.3084652644	-0.4718035699

36H^+

P	10.8996060909	6.277635745	1.4055999581
N	11.7621358523	4.9841950082	0.9456818081
P	11.5038560601	3.4587355099	0.5942051538

N	10.5638001066	3.0848264612	-0.7406113713
C	9.1215949244	2.7392690466	-0.6361469901
H	8.6636275186	3.1926049625	0.2454425638
H	9.0062941708	1.6500384475	-0.5597300166
C	8.5055911789	3.2705975563	-1.9400395787
H	7.6503310548	2.6712820571	-2.2667963024
H	8.1634566128	4.3006999429	-1.7894085821
C	9.6782521158	3.2417357143	-2.9304303318
H	9.533528863	3.8952855141	-3.7959650973
H	9.8413692939	2.2228312797	-3.3010719324
C	10.8623321752	3.6812847061	-2.0621630501
H	11.8230479948	3.3237086068	-2.4454055547
H	10.910657322	4.7775683721	-1.9927608707
N	12.9831599946	2.7854856563	0.2024678865
C	13.1894029017	1.5986104314	-0.6637181715
H	13.3799685217	1.9149153873	-1.698636051
H	12.3124824907	0.946838318	-0.677546163
C	14.1503924478	3.0023451538	1.0881878329
H	13.9189923742	2.7037283994	2.1217616821
H	14.4292800266	4.0591392485	1.1019034081
N	10.8150623793	2.6304367421	1.8733069558
C	10.2321113242	3.2726679645	3.0742205898
H	9.4930082536	4.0318334013	2.8013900679
H	11.0150409793	3.7608655807	3.6703035256
C	10.8205918723	1.1570333595	1.9949143044
H	11.7904378294	0.7417217035	1.7019329571
H	10.052780023	0.7032836852	1.35178
C	14.4339505671	0.9187070195	-0.0753594637
H	14.9812737268	0.3380587849	-0.823679074
H	14.146893484	0.2352804527	0.7337441428
C	15.2373095108	2.0981071745	0.4910137197
H	15.982960285	1.80016173	1.2337779702
H	15.759519243	2.6215746845	-0.3189808792
C	9.6052821642	2.0991184519	3.8415409597
H	9.552812707	2.2904124831	4.9172948515
H	8.5850076208	1.9173457529	3.4827038553
C	10.509588859	0.9127691091	3.4787731008
H	10.040377597	-0.0600751078	3.6509961177
H	11.4351902931	0.9497458994	4.0653083913
H	10.9126603652	6.2613970469	2.8172381502
N	11.5859816518	7.682415754	0.9770855377
P	13.0343212032	8.2366746014	0.6441367886
N	13.8259191123	7.5945219637	-0.6826998117
C	15.0046639196	6.6987052669	-0.603719646
H	15.0116741878	6.1259231103	0.3265777631
H	15.9305748875	7.2893072952	-0.6433280535
C	14.8771472004	5.7997195605	-1.8427255489
H	15.8503363267	5.4537489661	-2.2040512709
H	14.2673839357	4.9219600384	-1.5993510753
C	14.1384210912	6.6921646428	-2.8509209665
H	13.6608979059	6.1310676814	-3.659827769
H	14.831870227	7.4106836383	-3.3038854852
C	13.1183393489	7.4245028775	-1.970854483
H	12.8206599886	8.3932180597	-2.3866998099
H	12.2117437183	6.8194791334	-1.8331914027
N	12.8771837423	9.8515476858	0.2485889676

C	13.7702085893	10.6018371705	-0.6682613444
H	13.4099281384	10.5173328569	-1.7026083657
H	14.7944150539	10.2200470299	-0.6471460065
C	12.104332535	10.770374676	1.1123598435
H	12.5213329075	10.8004313548	2.1307061828
H	11.0654180556	10.4391745322	1.1878477745
N	14.0827641775	8.0925459303	1.9374542717
C	13.8798733538	7.1723740969	3.0811136328
H	13.6609296345	6.1545345621	2.7438553615
H	13.0375653903	7.5103837858	3.6998836468
C	15.3079140524	8.9029684976	2.103647968
H	15.1168947371	9.9552428512	1.8686709553
H	16.1122486947	8.5508752554	1.4410010323
C	13.6719336239	12.0541372958	-0.1756690449
H	13.8452496642	12.7754565192	-0.9795372889
H	14.4195182002	12.239706348	0.6055350966
C	12.25892785	12.1310021246	0.4207762111
H	12.124585262	12.9678064908	1.1123182291
H	11.5161423696	12.2316722984	-0.3796736851
C	15.2039994911	7.2601769532	3.85433792
H	15.0756504272	7.0449262052	4.9191167868
H	15.924059096	6.5381854482	3.4505041155
C	15.684408504	8.6919615128	3.5774983443
H	16.7538281889	8.833447351	3.7576260455
H	15.1384721534	9.4030309928	4.2086534269
N	9.336235848	6.1799688986	0.9809765241
P	8.1510935657	7.1832114411	0.6499536876
N	8.3117616976	8.1595475549	-0.6997655929
C	8.5573331478	9.6209213251	-0.6489105345
H	9.0307122424	9.9120167449	0.2910186786
H	7.6063361402	10.1658679197	-0.7314398758
C	9.4490862932	9.8935838383	-1.8677848635
H	9.3332830637	10.9139559224	-2.2460968237
H	10.4992656615	9.7422622894	-1.5934278309
C	9.003088134	8.8213741541	-2.8720936178
H	9.7346250762	8.6340102181	-3.6639744069
H	8.0606332536	9.1157299239	-3.3488528542
C	8.7839447392	7.5901202623	-1.982594637
H	8.0467494246	6.8966217679	-2.4019263888
H	9.7222555787	7.0388241557	-1.8337177965
N	6.8006676718	6.2662180528	0.2948782821
C	5.7160254547	6.6517108756	-0.6401999329
H	5.9551162251	6.3165590241	-1.6589144866
H	5.5740548261	7.7349244173	-0.677235485
C	6.3624037464	5.1855592221	1.2047231901
H	6.1521270578	5.5793248416	2.2109344892
H	7.1425887035	4.4260751946	1.3027155268
N	7.8065636815	8.2051756847	1.9275487826
C	8.680799592	8.3911993036	3.1095961504
H	9.6940493006	8.6804309379	2.8149995637
H	8.7500263168	7.4595157528	3.687060223
C	6.519865576	8.9150800185	2.0881007851
H	5.6813878216	8.2768198151	1.7908198652
H	6.4870068005	9.8234312076	1.4686878309
C	4.4825158268	5.9100672471	-0.1030632815
H	3.7578870794	5.6875676676	-0.8917257338

H	3.9742165166	6.5193666628	0.6546081758
C	5.081415287	4.6546618801	0.5475744877
H	4.4110986964	4.1766852949	1.2677578878
H	5.3335219012	3.9143683523	-0.2212867179
C	7.9686798025	9.4881342646	3.9155588011
H	8.1852611839	9.4195614235	4.9855931565
H	8.2910342185	10.4783618769	3.5713878207
C	6.485799234	9.2717235519	3.5809637792
H	5.8619511072	10.1463579607	3.7860669083
H	6.086994732	8.4290231946	4.1580009474

37

C	-1.4523403196	4.5364536573	-0.926036321
N	-1.5521717352	3.4210526061	0.0110181632
H	-0.516818051	5.1008730518	-0.7754735653
H	-2.2914253971	5.2292642824	-0.7860716322
H	-1.4666265865	4.1736344642	-1.9534615652
C	-1.3633620535	3.8280467279	1.3948185541
H	-0.3407992373	4.1855041909	1.5920597865
H	-1.5529044784	2.9837356883	2.0575712269
H	-2.067129599	4.6384693545	1.6207982328
C	-1.0389753514	2.1627133319	-0.3641102353
N	-1.4939945136	1.7369952384	-1.6043580437
C	-2.8557811121	1.9655627729	-2.0536165019
H	-3.4164938476	2.5010412546	-1.2866653304
H	-3.3512881146	1.0026770785	-2.2485318867
H	-2.8889210244	2.5494613958	-2.9868801584
C	-0.6857599645	0.9137567105	-2.4901315825
H	-0.6308217619	1.3987933536	-3.4778758819
H	-1.1133407682	-0.087954067	-2.6031809048
H	0.3177933922	0.7916965909	-2.0825054584
N	-0.2404671796	1.5604689147	0.4527828451
P	0.2178894971	-0.0756545179	0.7176887472
N	1.5667231238	-0.2079148282	-0.3723652855
N	-0.8807906747	-1.0632562644	-0.20504449
C	-1.7272301103	-1.8748769237	0.3338425522
N	-2.3235611597	-1.7883843511	1.5961021948
N	-2.1416085258	-3.0063559844	-0.3856915419
C	-2.5427631568	-0.5159968917	2.2597938123
H	-1.8132992616	-0.3291788893	3.0589838835
H	-3.5508415551	-0.5076343753	2.7017207054
H	-2.4734317914	0.2975524537	1.5373156124
C	-2.4972157935	-2.9547032977	2.4471918994
H	-1.8914206424	-2.8515474055	3.3601478196
H	-2.1752319554	-3.8507291803	1.9162354167
H	-3.5465151122	-3.0824132948	2.7546875495
C	-1.3840959725	-3.348777607	-1.5790443199
H	-0.3249551404	-3.1608589187	-1.4057347307
H	-1.6924776159	-2.7551129612	-2.4539334215
H	-1.5427400877	-4.4103504309	-1.8051586504
C	-3.5607656837	-3.3265673879	-0.4910508251
H	-4.1045228353	-2.9264062129	0.364362083
H	-3.7076236022	-4.4133655813	-0.5254770129
H	-3.9978587821	-2.8969037277	-1.4080490036
C	2.8032464929	-0.2575062829	-0.0079990463
N	3.7407262797	-0.8458625017	-0.8763905539

N	3.3743807718	0.2064786187	1.176972745
C	2.8179140937	1.3159590533	1.9334598109
H	3.6251125056	2.020386565	2.1886176838
H	2.3495147971	0.980097077	2.8683369377
H	2.0629239021	1.8404419515	1.3464720065
C	4.4430042538	-0.5113695495	1.8518577872
H	5.3432430261	0.1113241849	1.9715579356
H	4.7056255555	-1.4039623945	1.2839854899
H	4.1146005152	-0.8163638998	2.8567863699
C	4.9478197077	-0.1028189792	-1.2263050589
H	4.7896434027	0.5160926311	-2.1254724892
H	5.7739345763	-0.7943894761	-1.4325341104
H	5.2394178272	0.5560249394	-0.4085671719
C	3.2066687353	-1.6178547214	-1.9884828799
H	3.9987214665	-2.2717481046	-2.3731675267
H	2.8474673431	-0.9780468474	-2.8092213829
H	2.3664232889	-2.2215082839	-1.6455022821

37H^{+}

C	1.9122172726	4.7063450053	0.9532661808
N	1.6979032476	3.5271289627	0.1117456671
H	1.3215395187	5.5423894957	0.5592010063
H	2.9663972692	5.0067375933	0.9658902928
H	1.5845692563	4.5087821924	1.9732155553
C	1.6395010917	3.8098487735	-1.3187885649
H	0.7011335826	4.308981379	-1.59488858
H	1.7160728293	2.8833124519	-1.8863214687
H	2.4765440007	4.4650970679	-1.5794551038
C	1.1467729279	2.3694718812	0.5976228758
N	1.4849937304	2.0311362244	1.8822129124
C	2.8314749403	2.2381990993	2.4137678415
H	3.4838035797	2.6497388175	1.6438437102
H	3.2397157734	1.2711569979	2.7334808549
H	2.8288869939	2.9115740174	3.2796897699
C	0.6233015512	1.2022921019	2.7196271896
H	0.6497139043	1.5978992095	3.741476809
H	0.9552284627	0.1582849084	2.7278605361
H	-0.4035445896	1.2353732509	2.3559340988
N	0.3310350201	1.7021266809	-0.1946659353
P	-0.1232841423	0.1451074186	-0.2915065656
N	-1.5265452967	-0.1508363344	0.4941861355
N	0.992720729	-0.9108599471	0.2721324693
C	1.6451474623	-1.8658483601	-0.3535910194
N	1.9909172942	-1.8541883462	-1.6903876086
N	2.0353736324	-2.9635821242	0.3624555683
C	2.3299216081	-0.6227749178	-2.393183965
H	1.5792449882	-0.3624153823	-3.1505795005
H	3.2942335627	-0.7483602965	-2.9021067003
H	2.4188730245	0.2002401829	-1.683418392
C	1.9913758278	-3.0581294778	-2.5181069286
H	1.376021214	-2.8801271031	-3.4090732726
H	1.5615629523	-3.8930188851	-1.9643619914
H	3.0005483145	-3.3313605739	-2.85250681
C	1.4315455219	-3.2295802972	1.664533447
H	0.4207112173	-2.8255170903	1.6906198788
H	2.0114618309	-2.7705405754	2.4763421474

H	1.401583015	-4.3122511713	1.8251499919
C	3.3014990688	-3.6572525178	0.1233938945
H	3.8623166391	-3.1628916759	-0.6690538467
H	3.1422850937	-4.70700331	-0.1493351855
H	3.9049314223	-3.6284943644	1.0387301792
C	-2.7428221535	-0.2982980304	0.0126860118
N	-3.6336278803	-1.0443615914	0.7368613146
N	-3.2110776283	0.2304239635	-1.1712245698
C	-2.7736713542	1.5271486992	-1.6803853797
H	-3.651482477	2.1637517793	-1.852067896
H	-2.2388273616	1.423867822	-2.6330357022
H	-2.117956	2.0197760699	-0.9629457475
C	-4.1803501818	-0.4637965692	-2.015481991
H	-5.1518560035	0.0467284644	-2.0402827986
H	-4.3235166361	-1.4846869825	-1.661013952
H	-3.7937837796	-0.5022363338	-3.0415984317
C	-5.0485446889	-0.6854816383	0.8515424415
H	-5.2856630225	-0.4895472255	1.9042963938
H	-5.699614543	-1.4928565577	0.4967759755
H	-5.2601235229	0.2199088361	0.2836202636
C	-3.1422107804	-1.9191402081	1.7971409942
H	-3.8539024593	-2.7400738872	1.9297189822
H	-3.0386776351	-1.3824131248	2.7492737083
H	-2.169103164	-2.3227626901	1.5204440008
H	-0.3328990709	-0.0609577567	-1.6738311916

38

N	-1.6885207515	-1.2072756019	3.9174624061
C	-1.9504055015	-1.6310086522	2.7246374207
N	-2.9278255021	-1.0159443942	1.9324094849
C	-3.0835596382	-1.7159596112	0.7275543104
C	-2.1950176681	-2.7433079034	0.7277358192
N	-1.4749751299	-2.6947044866	1.9450313993
C	-4.0229073849	-1.2995829777	-0.3598634362
C	-1.8968116636	-3.7002518047	-0.3827386386
H	-3.7148606425	-0.3642614258	-0.8440257488
H	-5.0443833979	-1.1540409105	0.0084686923
H	-4.0663819169	-2.0686753328	-1.1349889838
H	-2.6224361356	-3.5685637674	-1.1893528147
H	-1.9486678112	-4.7499557327	-0.0728917531
H	-0.9004348403	-3.5380280763	-0.8138948527
C	-3.7197732642	0.0951392418	2.4787956344
C	-0.64419961	-3.7736194648	2.5112516665
C	0.6148433167	-4.0636850078	1.6829464788
C	-1.462191496	-5.0393568395	2.8109137284
H	-0.2942884225	-3.3695533844	3.4630890069
H	-1.8249040605	-5.5286652794	1.9010539379
H	-2.3253188368	-4.8012227719	3.4399624269
H	-0.8390575416	-5.7622319814	3.3499571439
C	-3.7375045794	1.3320853867	1.571606068
C	-5.1250549688	-0.3568091749	2.9023130812
H	-3.1677640081	0.3472558831	3.3889062072
H	-2.7239403277	1.5944404137	1.2503643782
H	-4.1457399296	2.1821886319	2.130279434
H	-4.3591053121	1.199730009	0.6805014421
H	-5.0578720607	-1.1965441698	3.6010753735

H	-5.7398357969	-0.6653275154	2.04917628
H	-5.64597537	0.4650511371	3.4074473854
H	0.3967750431	-4.5744095103	0.7405426816
H	1.2843115546	-4.7104027933	2.261170006
H	1.1524791598	-3.1366588541	1.4594284829
H	0.0524737972	4.4648667964	7.0851147116
H	2.0808893834	4.8037745357	6.4045842561
H	-1.076850602	3.5512625782	8.0937390731
C	-0.4663685881	3.5048476917	7.184292141
H	1.3500161323	2.1088565851	1.3172724146
H	-1.1390550138	3.385754098	6.3290883522
H	-0.1936001131	1.8035717488	2.1397466623
H	4.312798242	2.0545257614	3.109260374
H	3.4338459927	4.6256445117	5.2921839471
H	3.0940949054	2.9077171586	2.1582616988
C	2.8164340353	4.0902294498	6.0176403965
C	0.6961755175	1.3268481888	1.7163155335
H	0.3784482218	0.6982752406	0.8766605989
C	3.4875437905	2.7757325852	3.1719073229
H	-0.1000088695	1.4089862519	7.3554408891
C	2.1740000297	2.9039680353	5.3715446593
C	0.5029877489	2.3163577699	7.2676908191
C	2.4448479546	2.3549432003	4.1589476526
H	3.4673070575	3.8072162429	6.8545740679
N	1.216660714	2.0967991215	6.0020341708
N	1.6482614576	1.1946916018	4.0228775554
C	0.8649421602	1.0284026293	5.1700917756
H	3.9184705602	3.7335111057	3.4742794498
C	1.3876038963	0.4557747019	2.7769943354
H	3.3880436651	0.407902076	1.8586949523
N	-0.0405809834	0.1702973175	5.5110257285
H	0.8398128429	2.3339458214	9.4015409981
C	1.4362397098	2.3861589867	8.4833543139
H	2.1347612147	1.5429497708	8.4847630088
H	0.6717656232	-0.3119648048	3.0664031074
C	2.6268802743	-0.2756971813	2.2456850934
H	2.3324329863	-0.9384130074	1.4236464719
H	2.0144104326	3.3148327454	8.5227210963
H	3.075807327	-0.8883901802	3.0336164348
P	-0.3555095799	-1.4245250512	4.9845045757
C	-1.3338316639	-1.9540852394	6.5091379278
C	-0.4015826891	-2.0837900615	7.7201429889
H	-2.0458038222	-1.1358522332	6.6827910262
C	-2.1108019165	-3.2513041419	6.2601029173
H	-2.8055217961	-3.1389435453	5.4213411363
H	-2.6928093871	-3.5348868031	7.1471483822
H	-1.434425078	-4.0879764474	6.0364092344
H	0.339028011	-2.8799985547	7.56563797
H	-0.9685255017	-2.3323375014	8.6274730644
H	0.1428964148	-1.1521228344	7.902582565

38H^{+}

H	0.068261497	-0.2601442019	1.7760607046
P	-0.0872838217	-0.1079600675	0.3638422997
N	-0.5585956365	1.37185699	-0.0152589856
C	-1.5229552127	-1.2038308707	0.0145059454

N	1.2544526933	-0.6029550621	-0.385942686
C	0.0254799062	2.5667756602	0.0164907957
C	2.2289608852	-1.4749490861	-0.1689911049
N	-0.2199555488	3.5404247037	0.9464228408
N	3.1354748196	-1.521938665	0.8608230624
C	0.464452205	4.7109087621	0.5843703722
C	4.054803657	-2.5612562555	0.6226534385
C	1.1238050966	4.4487936213	-0.581261526
C	3.7194754425	-3.1336794031	-0.567853196
N	0.8499841897	3.1150683023	-0.9267966454
N	2.586263452	-2.4612377367	-1.0519736054
C	1.1965518655	2.3930172865	-2.1758624632
C	1.8918325675	-2.6249035957	-2.3513815587
C	2.0203583243	5.3564269699	-1.363166824
C	4.3693627489	-4.2927868808	-1.2551187366
C	0.4369546552	5.9824350483	1.372607951
C	5.1650207726	-2.939113059	1.5524161411
C	-1.0921862655	3.2906222173	2.1178152296
C	3.133170537	-0.5333310035	1.9617770422
C	-2.23282087	4.3083025277	2.2350194076
C	4.4741173216	0.1998074868	2.0992149947
H	-1.5384692897	2.3210194486	1.8852468032
H	2.4012907714	0.2079846674	1.6325635835
C	-0.2782320457	3.1450529155	3.4100336284
C	2.6439141681	-1.1387926063	3.2837529147
H	1.2024223078	6.6661999291	1.0000360988
H	5.5698569118	-3.9120980638	1.266430342
H	-0.5270081866	6.4974924979	1.2957849513
H	5.9942516413	-2.2231288733	1.5322552603
H	0.6426459909	5.8174842504	2.434702486
H	4.8248671443	-3.0276052363	2.5885964345
H	1.967042363	6.3684877272	-0.9568661584
H	5.3002180904	-4.5506817231	-0.7460332021
H	3.0685399389	5.0400600108	-1.319378002
H	3.7345883271	-5.1858348688	-1.2518112364
H	1.735322415	5.4192440651	-2.4177069141
H	4.6249209828	-4.0698750998	-2.2956539821
C	0.4080743591	2.9420949491	-3.3717170062
C	2.7426139465	-2.0900954991	-3.5107343194
C	2.7076950341	2.3267201091	-2.4207689895
C	1.3857891088	-4.0529063271	-2.5864548062
H	0.8565588694	1.375572398	-1.9818704895
H	1.0246720779	-1.969823124	-2.246832829
H	-2.9642485618	3.9357979492	2.9596214946
H	4.3365142201	1.0691388177	2.7506354006
H	-1.8938668011	5.2856396888	2.5878948979
H	5.2509240249	-0.4227198568	2.5490970594
H	-2.7446925878	4.4382031958	1.2764302126
H	4.8296528927	0.5568022697	1.1274215471
H	-0.9404017572	2.8384637915	4.2266365341
H	2.5203648131	-0.3447752082	4.0280876039
H	0.5007319942	2.3829566545	3.2994661832
H	1.6796601208	-1.6408826714	3.1568691493
H	0.2006937659	4.0830102802	3.7075313031
H	3.3549548664	-1.8635098868	3.6911250601
H	2.9019344008	1.6069984416	-3.223232838

H	0.6890439204	-4.0461270454	-3.4311195153
H	3.1312558286	3.2841534284	-2.7345461586
H	2.1898943182	-4.7500742297	-2.8350639694
H	3.234518333	1.9843231593	-1.5246173668
H	0.8510821813	-4.4366485593	-1.711613108
H	0.5933587077	2.3130948026	-4.2489749146
H	2.1511754025	-2.1009832233	-4.4324247156
H	-0.666817878	2.9303414118	-3.1665205684
H	3.0570773889	-1.0593372134	-3.3187158924
H	0.6997242711	3.9641660494	-3.6315214965
H	3.6368034549	-2.6961965871	-3.6856625721
C	-1.2295036243	-2.6342640642	0.496588844
C	-2.8216232305	-0.6500053653	0.62090901
H	-1.6098894922	-1.2021795245	-1.0807407761
H	-3.6570257133	-1.3175957705	0.3814797587
H	-2.7565409141	-0.5873821497	1.7145392775
H	-3.0494575892	0.3446873022	0.2311369358
H	-2.0646491298	-3.2932202644	0.2368349123
H	-0.3241432641	-3.0490545152	0.0420172112
H	-1.1121735705	-2.6723573423	1.5867650349

39

N	1.0692060076	0.2142372519	0.2103211877
C	2.3229097354	-0.0175355455	0.4183073882
N	2.7845651982	-0.5050733275	1.6476184625
C	4.1812183775	-0.6282980485	1.6231894067
C	4.6078612171	-0.2632984941	0.3857275385
N	3.4710963397	0.098604244	-0.371295171
C	4.992506296	-1.1344055036	2.773280589
C	6.0090770437	-0.254581875	-0.1381176194
H	4.8169883143	-2.200165166	2.9685383888
H	4.782569734	-0.5954997053	3.7040465749
H	6.0585563731	-1.0153528211	2.5633755773
H	6.7135637455	-0.3816122542	0.6881449364
H	6.2631100369	0.6882119206	-0.63468766
H	6.2048388998	-1.0630010084	-0.8539612298
C	1.8620529477	-0.5924150485	2.7874499048
C	3.4148626293	0.4608993068	-1.7960610461
C	4.0233250777	-0.6108382828	-2.7136147418
C	3.9681949517	1.8665434002	-2.0674421275
H	2.3458241817	0.4906401637	-2.021689186
H	5.0427315122	1.9381584092	-1.867777124
H	3.4427442193	2.597985859	-1.4471574998
H	3.8076106341	2.1312225893	-3.1193102259
C	1.891061637	-1.9591355861	3.4832065534
C	2.0362769638	0.584915478	3.7579900221
H	0.8868508631	-0.4747184197	2.3063133395
H	1.8006173208	-2.7687573128	2.7512579797
H	1.043765179	-2.0314436792	4.1750729616
H	2.8026646423	-2.1222258602	4.0670314735
H	1.9304939203	1.5313630186	3.2184995753
H	3.0113502254	0.5759012722	4.2584033154
H	1.2630318053	0.5475571649	4.5345145533
H	5.1167255692	-0.6299035251	-2.681449057
H	3.7309816316	-0.4037324422	-3.7493999829
H	3.6511174374	-1.6064376153	-2.4496866714

H	-5.1003560189	0.8626515088	2.0886486857
H	-6.2556510784	-0.3389843675	0.5910757504
H	-4.3415209722	2.4286604184	2.4150645867
C	-4.2077971009	1.4702185626	1.8994682377
H	-1.7197421972	-3.7966036436	-0.1016029318
H	-3.3441043127	0.9637207703	2.3413193912
H	-1.0414418074	-2.5073685921	0.9170954799
H	-3.9034765822	-3.0414257282	-2.6642672622
H	-6.3354566483	-1.657063116	-0.5741963732
H	-3.528346495	-3.8188770003	-1.1251039318
C	-5.9374165719	-0.652687033	-0.4097429891
C	-0.9645759992	-3.0054515844	-0.0542194977
H	0.0207781089	-3.4819439367	-0.1122697254
C	-4.0627459371	-2.9777760109	-1.5797239571
H	-3.0484402721	2.2843750252	0.3009344918
C	-4.4489029848	-0.6575091492	-0.5564498933
C	-3.9713530037	1.7062543285	0.3999679552
C	-3.6503043215	-1.6496615151	-1.0280340549
H	-6.4258034124	0.0159253412	-1.1302451426
N	-3.6402352446	0.4596704475	-0.3020714157
N	-2.3226477177	-1.1626971613	-1.0578943909
C	-2.3001346838	0.1577849304	-0.5900414551
H	-5.1285228558	-3.1403997879	-1.3996330707
C	-1.1008440596	-1.9753383963	-1.1863349927
H	-1.6526091892	-3.3793213252	-2.7938407317
N	-1.3541773779	1.01067883	-0.3883506664
H	-5.1412970353	3.5065465218	0.1661313938
C	-5.1051620187	2.4987291965	-0.2639767643
H	-4.9342755208	2.5961755806	-1.3411293544
H	-0.2843503011	-1.2691894799	-1.0418227934
C	-0.9309807148	-2.5835508091	-2.584523665
H	0.0719531436	-3.0175842582	-2.6715522809
H	-6.0893162173	2.0450902138	-0.1102212882
H	-1.0331600936	-1.8096603674	-3.3519953136
P	0.2439470548	1.1331919866	-0.9840552214
N	0.6719718568	2.7003952112	-0.4551514688
C	0.5428459505	3.8783706058	-0.9657241598
N	-0.2277084035	4.4003288773	-2.0103879822
C	0.0218669352	5.7852230934	-2.1395789371
C	0.9076816549	6.1392652001	-1.1725573127
N	1.2430107228	4.9821893728	-0.455077427
C	2.0268590248	4.8458938646	0.7788859072
C	1.5040295321	7.4849926228	-0.906739958
C	-0.5904155048	6.6564064292	-3.1902295428
C	-1.1036974344	3.5615212163	-2.8417869257
H	1.37289669	7.8026150632	0.1341121578
H	2.5795679125	7.5140397375	-1.1219989513
H	1.0240794036	8.2396187149	-1.5346348537
H	-0.0965672128	7.6314673756	-3.1964428112
H	-0.4808580021	6.2352430933	-4.1965975902
H	-1.6603144632	6.8350077126	-3.0267255473
H	2.0500088345	3.7617706767	0.9213786379
C	3.4736711754	5.3373894777	0.6380591785
C	1.3053420223	5.4540738258	1.9912454044
H	-1.2240737017	2.6451246766	-2.2617599234
C	-2.5165838033	4.1376251684	-3.0214854198

C	-0.445962641	3.1845664567	-4.1765839874
H	-1.0962571623	2.4994581447	-4.7333650757
H	-0.2622352269	4.062303241	-4.8070838353
H	0.5057543165	2.6752114954	-3.9983812924
H	-3.1740547747	3.3431840953	-3.3920218122
H	-2.9190585267	4.4902629629	-2.0663152092
H	-2.5596336469	4.9575969534	-3.7435954213
H	1.8571110407	5.2213013235	2.9098720359
H	1.2196988901	6.544607333	1.9239682877
H	0.2993757698	5.0328007785	2.0828719776
H	4.0547839915	5.0065457325	1.5067661625
H	3.9408439639	4.9200065062	-0.2601522036
H	3.5500255877	6.4285070034	0.591652704

39H⁺

H	0.0000000059	-0.000000003	1.6781077803
P	0.0000000059	-0.000000003	0.2487844976
N	0.1916032838	-1.535562284	-0.2227540871
N	1.2340343115	0.9337144436	-0.2227540871
N	-1.4256375775	0.6018478315	-0.2227540871
C	-0.6054474363	-2.5890025105	-0.0912991436
C	2.5448656691	0.7701683852	-0.0912991436
C	-1.9394182149	1.8188341164	-0.0912991436
N	-0.4383018459	-3.606446384	0.8142274778
N	3.3424251152	1.4236426493	0.8142274778
N	-2.9041232515	2.1828037258	0.8142274778
C	-1.3879942419	-4.611038609	0.5713022314
C	4.6872737005	1.1034810211	0.5713022314
C	-3.2992794408	3.5075575789	0.5713022314
C	-2.1370833062	-4.2077652248	-0.4954117838
C	4.7125732373	0.2531141697	-0.4954117838
C	-2.5754899133	3.9546510462	-0.4954117838
N	-1.6494350727	-2.9546425897	-0.9003696855
N	3.3835130845	0.0488686104	-0.9003696855
N	-1.734077994	2.9057739704	-0.9003696855
C	-2.0204879454	-2.1574696875	-2.0931727862
C	2.8786675363	-0.6710590546	-2.0931727862
C	-0.8581795731	2.8285287332	-2.0931727862
C	-3.3023066151	-4.9017474045	-1.1270228269
C	5.8961910891	-0.4090077271	-1.1270228269
C	-2.5938844562	5.3107551227	-1.1270228269
C	-1.5052868678	-5.8702958214	1.3704506458
C	5.8364687493	1.6315312336	1.3704506458
C	-4.3311818637	4.2387645789	1.3704506458
C	0.6078920006	-3.5397977382	1.8571340418
C	2.7616087715	2.2963487748	1.8571340418
C	-3.3695007544	1.2434489545	1.8571340418
C	1.5503763231	-4.7491252398	1.8298043456
C	3.3376749482	3.7172278915	1.8298043456
C	-4.8880512535	1.0318973393	1.8298043456
H	1.1856848971	-2.6612732316	1.5588937887
H	1.7118877828	2.357469848	1.5588937887
H	-2.8975726621	0.3038033748	1.5588937887
C	0.0152277775	-3.2701617849	3.2464509846
C	2.8244292978	1.648268525	3.2464509846
C	-2.8396570575	1.621893251	3.2464509846

H	-2.4205523708	-6.3996005424	1.0975127455
H	6.7524928355	1.1035404173	1.0975127455
H	-4.3319404469	5.2960601162	1.0975127455
H	-0.6678712106	-6.5546833588	1.1938251164
H	6.0104579141	2.6989482349	1.1938251164
H	-5.3425866857	3.8557351149	1.1938251164
H	-1.5547365496	-5.6771888452	2.4468027503
H	5.6939580432	1.4921530648	2.4468027503
H	-4.1392214757	4.1850357715	2.4468027503
H	-3.4072813577	-5.9059264162	-0.7107687541
H	6.8183229945	0.0021709849	-0.7107687541
H	-3.411041619	5.9037554224	-0.7107687541
H	-4.2447499347	-4.3720565326	-0.947429938
H	5.9086869977	-1.4900330194	-0.947429938
H	-1.6639370452	5.8620895431	-0.947429938
H	-3.1866958226	-5.0136532367	-2.2094061743
H	5.9352989863	-0.2529329277	-2.2094061743
H	-2.748603146	5.2665861555	-2.2094061743
C	-1.574711207	-2.8483761069	-3.3885641805
C	3.2541216779	0.060448135	-3.3885641805
C	-1.6794104531	2.787927963	-3.3885641805
C	-3.4957284334	-1.7461348285	-2.0962503471
C	3.2600613429	-2.1543222234	-2.0962503471
C	0.2356671082	3.900457043	-2.0962503471
H	-1.4350791613	-1.245899595	-1.9730635129
H	1.7965202868	-0.6198652222	-1.9730635129
H	-0.3614411077	1.8657648083	-1.9730635129
H	2.4231798641	-4.5334486055	2.4552328822
H	2.7144917334	4.3652596134	2.4552328822
H	-5.1376715797	0.1681889832	2.4552328822
H	1.0855206357	-5.6571015797	2.2229477036
H	4.3564333683	3.7686392271	2.2229477036
H	-5.4419539862	1.8884623437	2.2229477036
H	1.9040156783	-4.9495146362	0.8133183395
H	3.3343975786	4.1236832551	0.8133183395
H	-5.238413239	0.8258313722	0.8133183395
H	0.8259560694	-3.0981388101	3.9628466552
H	2.2700888856	2.264368334	3.9628466552
H	-3.0960449372	0.8337704672	3.9628466552
H	-0.6216072842	-2.3796983519	3.2327641994
H	2.3716828745	0.6515214671	3.2327641994
H	-1.7500755725	1.7281768759	3.2327641994
H	-0.5783304323	-4.1119418753	3.6166942503
H	3.8502113453	1.5551220819	3.6166942503
H	-3.2718808952	2.5568197845	3.6166942503
H	-3.6533756825	-1.006312475	-2.8885397064
H	2.698180015	-2.6607599227	-2.8885397064
H	0.9551956853	3.6670723888	-2.8885397064
H	-4.1723023188	-2.5825252173	-2.2915326585
H	4.3226836098	-2.3220572013	-2.2915326585
H	-0.1503812732	4.9045824097	-2.2915326585
H	-3.7658758247	-1.2814260478	-1.1437594195
H	2.9926854292	-2.6206311173	-1.1437594195
H	0.7731904133	3.9020571563	-1.1437594195
H	-1.7348912099	-2.1713860468	-4.2346811738
H	2.7479210892	-0.4167668467	-4.2346811738

H	-1.0130298616	2.5881528846	-4.2346811738
H	-0.5092013888	-3.0968920438	-3.3486093866
H	2.9365878834	1.1074646739	-3.3486093866
H	-2.4273864768	1.9894273609	-3.3486093866
H	-2.1343702953	-3.7664231572	-3.5926280466
H	4.3290032895	0.0347926723	-3.5926280466
H	-2.1946329765	3.7316304761	-3.5926280466

b) carbon bases and their conjugated acids

Y1

P	-0.0939232402	0.0980051262	0.033282505
C	0.0734692019	-0.045396338	1.860647508
C	1.6767482989	0.0150698124	-0.57399539
C	-0.811845276	-1.4533347488	-0.6358685286
C	2.4176564544	-1.171496752	-0.7000373217
C	3.732266699	-1.1398303043	-1.1689936323
C	4.3222389835	0.0775468022	-1.5179211405
C	3.5916702106	1.2616542256	-1.4038933025
C	2.275240535	1.226887505	-0.9410602544
H	1.965682952	-2.1263126627	-0.4433890645
H	4.2944711066	-2.0652783555	-1.2664734487
H	5.3449191691	0.1007007107	-1.8854122261
H	4.0435787861	2.2099530718	-1.6835554828
H	1.688999565	2.1414025941	-0.8767346149
C	-0.6425151413	-1.7778914207	-1.9905094853
C	-1.2688670788	-2.9020859691	-2.5307491879
C	-2.0570329467	-3.7212972074	-1.7208574487
C	-2.2235301445	-3.4093632372	-0.3701276197
C	-1.6092936082	-2.2787090972	0.1689930998
H	-0.0171924242	-1.155906993	-2.6246342131
H	-1.1362221393	-3.1395823935	-3.5829614564
H	-2.538958772	-4.600461303	-2.1404146945
H	-2.8342628802	-4.0452300529	0.2652885429
H	-1.7526705367	-2.0356871295	1.2174929772
C	-0.9882130892	0.4222292443	2.6517586882
C	-0.9296988512	0.3235270407	4.0423817953
C	0.1839718118	-0.2501421024	4.658211146
C	1.2438990521	-0.7176903432	3.878827512
C	1.1924597854	-0.6131599393	2.4884857375
H	-1.8456881312	0.8684165941	2.1569067246
H	-1.755091533	0.6950927222	4.6440690679
H	0.2281714256	-0.3298052836	5.7413803843
H	2.1168518532	-1.1581740082	4.353322039
H	2.0314249978	-0.9629194745	1.8962231956
C	-1.1029044285	1.3932282297	-0.3315001162
H	-1.5416204777	1.4254834026	-1.3239749802
H	-0.9285461892	2.3431700346	0.1658776857

Y1H^{+}

P	0.0406471817	-0.0695584513	0.0287606558
C	0.0189300924	-0.0380230143	1.8380884127
C	1.7425469232	-0.0426898621	-0.5863599793
C	-0.833990042	-1.5281829475	-0.5896042565
C	2.3042463375	-1.1826317977	-1.1828646739
C	3.6207233808	-1.1449709944	-1.6434615737
C	4.3765179131	0.0207131274	-1.5122512675
C	3.8209725804	1.1568939085	-0.9165337176
C	2.508239521	1.1305089728	-0.4526703025
H	1.7202132458	-2.0904181202	-1.293187607
H	4.0523118421	-2.0268506474	-2.1066048793
H	5.3997846541	0.0465183583	-1.8746708409
H	4.4091006848	2.0637288093	-0.8151379041
H	2.0922097458	2.0190147361	0.0142784504

C	-1.209288867	-1.5922168251	-1.9441924346
C	-1.8659192489	-2.7211696727	-2.427802337
C	-2.1495209472	-3.7881395353	-1.570284388
C	-1.775481841	-3.7290690919	-0.2270934224
C	-1.1183049442	-2.6026930608	0.2684515755
H	-0.9892348148	-0.7740610209	-2.6241786303
H	-2.1575806609	-2.7673786359	-3.4724630252
H	-2.663477165	-4.6654571641	-1.9514054736
H	-1.9963296821	-4.5575600409	0.4387248113
H	-0.8354312738	-2.5610877383	1.3152724836
C	-1.1819912924	0.2506667563	2.5121510588
C	-1.2084872331	0.2623186581	3.9044473304
C	-0.0457866217	-0.0141768687	4.6297356237
C	1.1456101379	-0.3055079259	3.9639768588
C	1.184062347	-0.3187210478	2.569471722
H	-2.0950782867	0.4596407323	1.9618074114
H	-2.135113242	0.4883807593	4.42287366
H	-0.0705700997	-0.0017515368	5.7152428617
H	2.0482514883	-0.5202568163	4.52755231
H	2.114789977	-0.5406155015	2.0576990903
C	-0.8148605868	1.4229870719	-0.5877666236
H	-0.7830278744	1.4451346496	-1.6802048094
H	-1.8581308099	1.4179347744	-0.2620066842
H	-0.3240365194	2.3192940037	-0.1997134857

Y2

P	-0.0142103316	-0.0451164331	-0.2916192496
C	0.0639247745	0.0443520868	1.5831027041
C	1.7833957885	-0.1067740821	-0.7123960583
C	-0.8995326895	-1.6402185082	-0.5529845349
C	2.4160856239	-1.1636393474	-1.3909139585
C	3.7799479363	-1.1209535023	-1.7242060869
C	4.5259232554	0.0081555554	-1.3826870356
C	3.932114933	1.0864452112	-0.7186786402
C	2.5775464472	1.0210897335	-0.399037355
O	1.6361333904	-2.2344237889	-1.6991246601
H	4.2395899847	-1.9520586268	-2.2380736541
O	5.8527877164	0.1574744182	-1.6583714535
H	4.544029211	1.9470883048	-0.4837865231
O	1.9192817314	2.0355886038	0.2265616083
C	-1.5568243502	-1.8393796554	-1.7766823487
C	-2.2684183293	-3.0147487436	-2.0136973883
C	-2.3212728305	-4.0138407063	-1.0386421162
C	-1.6665768944	-3.8247587752	0.1790125328
C	-0.9643341846	-2.6431397431	0.4235547407
H	-1.5013408578	-1.0599122147	-2.5298551989
H	-2.7810970733	-3.1516475728	-2.9626898693
H	-2.8731703166	-4.9317916844	-1.2254229312
H	-1.7085222522	-4.5935159615	0.9465446665
H	-0.4753155265	-2.5036961721	1.3816878762
C	-0.9594803992	0.7485892973	2.2281738387
C	-0.9847261322	0.8569027839	3.6194897578
C	0.0233803924	0.2663641868	4.3842629921
C	1.0555909824	-0.4298864235	3.7512171605
C	1.0759812994	-0.5389553998	2.3589183864
H	-1.7272626387	1.2280830343	1.6242033573

H	-1.7861666526	1.4072903601	4.1064549667
H	0.0091358485	0.353322107	5.4678265238
H	1.8470448349	-0.885618141	4.3416948949
H	1.8893799577	-1.0741245501	1.874842407
C	-0.8073362667	1.1115375739	-1.2231731422
H	-0.3653551103	2.0987999004	-1.299825272
H	-1.889087462	1.0436318431	-1.2880787555
C	2.6446054858	3.1821908081	0.6430534326
H	1.916604169	3.8258227856	1.1390148232
H	3.4405378892	2.9169747178	1.3501590125
H	3.0793098061	3.7136000868	-0.2129425059
C	2.1818342089	-3.3232174227	-2.4232477624
H	1.3540860354	-4.0194736652	-2.5669905818
H	2.5652717328	-3.005661416	-3.4015723276
H	2.9836870299	-3.8223485138	-1.8629648522
C	6.5243464479	-0.8844463199	-2.3485830905
H	7.5559124874	-0.5475840109	-2.4661127035
H	6.5126561036	-1.8212337926	-1.7759637112
H	6.0853317942	-1.0602992253	-3.3393229145

Y2H$^+$

P	0.1463352211	-0.0407323035	0.1450566484
C	0.1566547842	-0.373262822	1.9312192327
C	1.8121695362	0.043776813	-0.5273767349
C	-0.8418082366	-1.2923087788	-0.7379152269
C	2.5241150474	-1.1735999582	-0.6842388296
C	3.7905288403	-1.2200412504	-1.2664983942
C	4.382126698	-0.0195094106	-1.6833763586
C	3.7251855001	1.208695221	-1.5077765359
C	2.4633077479	1.2452832217	-0.9295382835
O	1.8872776651	-2.2690666706	-0.2069782836
H	4.3083584588	-2.1614005516	-1.3746553041
O	5.5964812003	0.0637139858	-2.2544357715
H	4.2364424001	2.1059478105	-1.8284196492
O	1.7874273617	2.3982617412	-0.7137261581
C	-1.1312587706	-1.0734798509	-2.094454108
C	-1.906779227	-1.993506904	-2.7981983486
C	-2.3987232514	-3.1313495737	-2.154759061
C	-2.109684223	-3.3524751811	-0.8070436061
C	-1.3302095122	-2.4390342514	-0.0969423491
H	-0.7491092777	-0.1962597382	-2.609847424
H	-2.1276771345	-1.8200912943	-3.8470656049
H	-3.0060678162	-3.8446560645	-2.7040031278
H	-2.4895269217	-4.2376566459	-0.3055425541
H	-1.1011945898	-2.6224327468	0.9474119079
C	-1.0597580078	-0.4128729709	2.6363654366
C	-1.0578684188	-0.6120536055	4.0153450304
C	0.1510304574	-0.7667413931	4.6989342329
C	1.3595587168	-0.723839834	4.0027787935
C	1.3667427505	-0.528229852	2.6214858306
H	-2.0078670801	-0.2966218476	2.1183889186
H	-1.9992043453	-0.6431944064	4.5555154012
H	0.1490317938	-0.9180820573	5.7742869185
H	2.2996061231	-0.8403559426	4.5334869118
H	2.3092458184	-0.4888893447	2.0858634388
C	-0.7856482633	1.5191264201	-0.0706332839

C	2.3816915595	3.6418467166	-1.1024024733
H	1.6542334044	4.4081210776	-0.8336843393
H	3.3164730369	3.8151031269	-0.5591562656
H	2.5651579264	3.6694680973	-2.1816866424
C	2.445931534	-3.5683703114	-0.4174298591
H	1.7136534334	-4.2676533278	-0.0141781603
H	2.5923110628	-3.762609484	-1.4851610186
H	3.3945117176	-3.6785797608	0.119302948
C	6.3559170084	-1.1264771295	-2.4770338452
H	7.2802623372	-0.7971870003	-2.9512717826
H	6.5886252032	-1.6282420354	-1.530831721
H	5.8250819372	-1.813286448	-3.1459773384
H	-0.7855832336	1.828569217	-1.1170390731
H	-1.8143656764	1.327327059	0.2476708057
H	-0.3478262963	2.3097402403	0.5377480608

Y3

P	0.2356383768	0.0439300504	0.0627346175
C	0.1226468195	-0.0654885933	1.8984948276
C	1.9104832047	-0.3182402605	-0.6232233529
C	-0.7018327814	-1.4913656543	-0.5548058326
C	2.3904715393	-1.6242704799	-0.7867833577
C	3.6545808593	-1.8487111286	-1.332867633
C	4.4458668004	-0.7715246287	-1.7383000404
C	3.9693635998	0.5321333969	-1.5851280735
C	2.7133295525	0.7584330477	-1.0204828824
H	1.7804898852	-2.4678034373	-0.4813162749
H	4.0190781281	-2.8666491642	-1.4489783113
H	5.4272531952	-0.9481061847	-2.1722430593
H	4.5781625049	1.3755779488	-1.9023786639
H	2.3341969083	1.7655945186	-0.873948839
C	-1.3314007328	-1.4142095906	-1.8192339071
C	-2.2012845803	-2.4024344587	-2.2832002178
C	-2.4323939312	-3.5317403019	-1.494076956
C	-1.7816209655	-3.6902077338	-0.2696792389
C	-0.9118481123	-2.6775308738	0.168205937
O	-1.0168330184	-0.3188205399	-2.5653477618
H	-2.6933859928	-2.3384329861	-3.2446941204
O	-3.3010883577	-4.4448227647	-2.0232829928
H	-1.918991806	-4.58626717	0.3181353174
O	-0.181448895	-2.818524772	1.3178609899
C	-1.1483272344	-0.055623128	2.5150291315
C	-1.2987407296	-0.0692481878	3.9016689469
C	-0.1592730825	-0.085337275	4.7111197797
C	1.1162813674	-0.0847072025	4.1469710993
C	1.2416236752	-0.0689328267	2.7474075907
O	-2.2154420502	-0.0303964776	1.6671027537
H	-2.2690468174	-0.0564585001	4.3798693688
O	-0.4010461247	-0.097798586	6.0555598386
H	1.9980979306	-0.0880675911	4.7703172902
O	2.4580055419	-0.0584651558	2.1396894411
C	-0.1662541877	1.6450244893	-0.3063911264
H	-0.1587103539	1.9287105513	-1.3554156822
H	-0.9846884737	2.0566891286	0.2781508586
C	-3.5270043129	-0.0242686287	2.2036171106
H	-4.1967391319	-0.0104508028	1.3421553726

H	-3.7229683534	-0.9231648587	2.8029244283
H	-3.7063490545	0.8662022944	2.8197981491
C	3.639559171	-0.0074642706	2.9167318052
H	4.4588481023	0.020931222	2.1964314034
H	3.6734035961	0.8935403547	3.5433973631
H	3.7466593949	-0.8962712504	3.5533283923
C	0.7061870086	-0.0781642564	6.9407931396
H	0.2844294216	-0.0787647533	7.9477776964
H	1.3416904751	-0.9650619107	6.8147736964
H	1.3167290879	0.8241264966	6.8042740951
C	-1.6724439853	-0.1102435325	-3.8034900426
H	-1.2975850566	0.8435105066	-4.1794253497
H	-1.435703884	-0.9032028461	-4.5251756942
H	-2.7612487118	-0.0492636133	-3.678275291
C	-0.4459201217	-3.9041818513	2.1876300252
H	0.1907068515	-3.7451279263	3.0599821011
H	-1.4965580935	-3.92233613	2.5051456573
H	-0.1915081778	-4.8679794398	1.7258132314
C	-3.5682804336	-5.6265769782	-1.2887586952
H	-4.2844348632	-6.1950398654	-1.8854886128
H	-2.6608061464	-6.228308208	-1.1443754922
H	-4.0106844443	-5.4031022299	-0.3086719536

Y3H^+

P	-0.0202297504	-0.2033116676	0.1419523862
C	0.0264499249	-0.1050181008	1.9458628734
C	1.6609773173	-0.0703582033	-0.556004062
C	-0.8365851535	-1.6844623546	-0.5004614487
C	2.2457356917	-1.1422170352	-1.2393862297
C	3.5182721875	-1.0022484583	-1.7942214325
C	4.2095621839	0.2041432491	-1.6698237021
C	3.6266877271	1.2759339703	-0.989480443
C	2.3544977471	1.1431192669	-0.4351087742
H	1.7117796039	-2.0821283994	-1.3361133796
H	3.967087726	-1.8357169727	-2.3268020132
H	5.1988684042	0.3112548043	-2.1051575435
H	4.1601125294	2.2170900901	-0.8935711259
H	1.9145949362	1.9837658325	0.094705643
C	-1.2025865043	-1.717247782	-1.8761920955
C	-1.8668781201	-2.8016766495	-2.429735046
C	-2.1771703905	-3.9030748693	-1.6178086205
C	-1.8103427737	-3.9258133082	-0.2679619195
C	-1.1353804034	-2.8293750609	0.2810281784
O	-0.8449794282	-0.6224819108	-2.5947902655
H	-2.1539839247	-2.8361341234	-3.4717773615
O	-2.826903041	-4.9050066453	-2.2421457792
H	-2.0328349273	-4.7857467781	0.3453737017
O	-0.725516963	-2.8100345263	1.5687637352
C	-1.1172628319	0.3426025172	2.6594243547
C	-1.0907616382	0.5144061097	4.0367796984
C	0.0770187499	0.1985449495	4.74740975
C	1.1989966765	-0.3197014247	4.0905946003
C	1.1561255665	-0.4960824697	2.7031274615
O	-2.2234396289	0.5739279953	1.9059601557
H	-1.9459701408	0.8742867793	4.592410873
O	0.0088674002	0.4096678276	6.0778234347

H	2.0809421527	-0.6025834934	4.6455130021
O	2.1710446515	-1.0603483053	2.0119772995
C	-0.8779303798	1.3098890112	-0.4659098671
C	-3.4061381317	1.0680893145	2.5380953278
H	-4.1385101323	1.1860537093	1.7389389898
H	-3.7822554852	0.3534997782	3.2782118192
H	-3.2223723346	2.0361835979	3.0168480618
C	3.4053173173	-1.3533583549	2.6638577413
H	4.0665091035	-1.7190990815	1.8779131855
H	3.836769602	-0.4545967802	3.1185688428
H	3.2764313918	-2.1309225679	3.4257272889
C	1.1477073299	0.1305728963	6.891118086
H	0.8560583966	0.4016350908	7.9059238252
H	1.4074240142	-0.9342519758	6.8584211756
H	2.0103922593	0.7335250942	6.584627397
C	-1.1566926589	-0.5682752801	-3.9892298823
H	-0.7695869885	0.3890967311	-4.3387636735
H	-0.6658812549	-1.3833897009	-4.5316168695
H	-2.239127843	-0.6111316338	-4.1514486957
C	-1.0235690535	-3.9082577226	2.4282346714
H	-0.6139834341	-3.631925993	3.400253864
H	-2.1050348954	-4.0617768193	2.5172784338
H	-0.5443199471	-4.8292229044	2.0767648658
C	-3.1897302456	-6.0732472512	-1.506543829
H	-3.7020994536	-6.7197291412	-2.2191192371
H	-2.3029674872	-6.5878609065	-1.1183549145
H	-3.8689530338	-5.8265842333	-0.6822160637
H	-0.4744024745	1.5661957526	-1.4442870677
H	-1.9497376694	1.1331538203	-0.539298154
H	-0.6983330685	2.1229966974	0.2407447722

Y4

P	-0.0240145493	0.223429898	-0.0487121188
C	-0.0437519986	0.0442343242	1.7790414178
C	1.7050050713	0.0762232382	-0.6614501488
C	-0.8221786081	-1.3787055271	-0.608114676
C	2.2721130113	-1.1570413206	-1.0071367793
C	3.6039788848	-1.1954713666	-1.4435433012
C	4.3532973721	-0.0202783263	-1.5334581277
C	3.7757223588	1.2043529166	-1.1912608509
C	2.4471555245	1.2612104027	-0.7467250362
H	1.6787588172	-2.0594596037	-0.9512673637
O	4.2583694672	-2.3368794314	-1.8085452847
H	5.3813615108	-0.058781362	-1.8763886465
O	4.5870920835	2.2934840168	-1.319965065
H	1.9616446189	2.1922755645	-0.4810055908
C	-1.5784595917	-1.3142223615	-1.7811264489
C	-2.1990274807	-2.4749556855	-2.2661712164
C	-2.0750047053	-3.6800464611	-1.5713110622
C	-1.3310161313	-3.7331413382	-0.3897962792
C	-0.6955729925	-2.5819951804	0.0998391198
H	-1.6885960123	-0.356437127	-2.2790706877
O	-2.9540927862	-2.5276030573	-3.4019611118
H	-2.5683435623	-4.572259281	-1.9406366598
O	-1.2857796838	-4.9555584704	0.2189863682
H	-0.1260891208	-2.6094525963	1.0210689289

C	-1.2815942462	-0.0908038574	2.4221607034
C	-1.3192436482	-0.158651486	3.8219801128
C	-0.1357346727	-0.1105197875	4.5625650876
C	1.0919539886	0.0137892337	3.9106618353
C	1.1471565771	0.0936620047	2.5107864078
H	-2.1919206733	-0.147922237	1.8382384604
O	-2.4634724473	-0.2842050122	4.5548178019
H	-0.1715483002	-0.1705047806	5.6445655476
O	2.1875257362	0.0441719575	4.7237620695
H	2.0919880843	0.202231668	1.9944235817
C	-0.6412808614	1.727549449	-0.4843043976
H	-0.4999659527	2.065820164	-1.5068423724
H	-1.5233164807	2.0837616506	0.0388335783
C	3.4675660172	0.184423704	4.1257816573
H	4.1820725292	0.189574613	4.9509638677
H	3.6949772261	-0.6539789199	3.4545618476
H	3.5522004132	1.1250961901	3.5665557192
C	-3.7036703427	-0.3266137774	3.8652109277
H	-4.4703519549	-0.4162169681	4.6369542042
H	-3.8756335828	0.5907391921	3.2873967313
H	-3.7631322133	-1.1924632853	3.1927798038
C	-0.5780869363	-5.0703097253	1.4435122338
H	-0.6889461333	-6.1103330503	1.7560038389
H	0.4889783547	-4.8423062091	1.3179904462
H	-0.9957032997	-4.4117718809	2.2160789649
C	-3.1208017278	-1.3337801967	-4.1523658315
H	-3.7402821663	-1.6051296822	-5.0092207978
H	-3.6298956912	-0.5566051075	-3.5678214035
H	-2.1588532961	-0.9429634072	-4.5084667102
C	4.0493460955	3.5706704932	-1.0064760325
H	4.8547168265	4.2844963734	-1.1890054253
H	3.1923461521	3.8157496087	-1.6464464823
H	3.739681328	3.6320041695	0.0447498285
C	3.5458948149	-3.5638270707	-1.7665124079
H	4.2493127208	-4.3280130751	-2.1022712483
H	3.2108025922	-3.802395614	-0.7483298527
H	2.676258673	-3.5507352057	-2.4359996747

Y4H^+

P	-0.0973605329	-0.1660168505	-0.0784550748
C	-0.1073507766	0.0415586829	1.7199670343
C	1.5794506216	0.0151023959	-0.7360540186
C	-0.7878189934	-1.7764089488	-0.5333676839
C	2.2654164033	-1.1112715111	-1.1965176335
C	3.5708364196	-0.9569461769	-1.6891621204
C	4.174629697	0.3073585383	-1.7185042064
C	3.4693139955	1.4264040429	-1.2487454581
C	2.1655691502	1.2868759835	-0.7543409955
H	1.8241201552	-2.1004233351	-1.1934831804
O	4.1637938556	-2.0950544458	-2.1173732943
H	5.1788325113	0.4212967952	-2.1019486078
O	3.9628388726	2.6859964664	-1.2335516833
H	1.6591338981	2.1747852004	-0.3927323938
C	-1.2096290041	-1.983818809	-1.8521632322
C	-1.7167169339	-3.2392233878	-2.2135644951
C	-1.7976202145	-4.2718863909	-1.2665258619

C	-1.3666019906	-4.044878916	0.0477396109
C	-0.8582094655	-2.7916864418	0.4238486763
H	-1.1546603152	-1.2166966528	-2.6161809408
O	-2.1054374757	-3.3568565287	-3.5040000722
H	-2.1918018799	-5.2377074956	-1.5501041786
O	-1.4027128949	-4.9718368638	1.0324296852
H	-0.5414785185	-2.6447053794	1.4491847199
C	-1.3391964506	0.1670754221	2.3745571918
C	-1.3557814288	0.3184015391	3.7673517127
C	-0.1547398628	0.3436472655	4.4938357685
C	1.0669658481	0.2135649509	3.8201700535
C	1.098456014	0.0612591279	2.4247613226
H	-2.2893083692	0.1398480732	1.8525711726
O	-2.584024513	0.4331659138	4.3230434703
H	-0.1731939214	0.4631893861	5.5679940645
O	2.2767942219	0.2241042995	4.4256111109
H	2.0595317271	-0.0305193918	1.9339318726
C	-1.144689337	1.1394835577	-0.8098286912
C	5.4902312227	-2.0344640571	-2.6374668257
H	5.7453956074	-3.0586880606	-2.91029766
H	5.5393874162	-1.3968826117	-3.5285343439
H	6.1976570133	-1.6716302574	-1.8820039016
C	5.2830156288	2.9227666062	-1.7190406934
H	5.4500899062	3.9938078605	-1.6029439899
H	6.027440565	2.3719107766	-1.1316101589
H	5.3717095947	2.652785533	-2.7782470493
C	2.3439726473	0.3760472226	5.8419808791
H	3.4055489143	0.3546229093	6.0893132101
H	1.9148270288	1.3339002795	6.1600969473
H	1.8335830037	-0.447942776	6.3552378765
C	-2.6970977116	0.5920068936	5.7359901633
H	-3.7661666178	0.65946284	5.9386369062
H	-2.27887968	-0.271138066	6.2677604519
H	-2.2034791749	1.5112387532	6.0736607038
C	-1.9077722332	-6.2737370654	0.7431480716
H	-1.8348430602	-6.830986911	1.6773322049
H	-2.9564063283	-6.2316512068	0.424595988
H	-1.3058251051	-6.7719586527	-0.0264199066
C	-2.6413716687	-4.5975836822	-3.9600371947
H	-2.8764350586	-4.4454740899	-5.0136647436
H	-1.9079170519	-5.4071185578	-3.863454492
H	-3.5572217637	-4.8588978948	-3.4163623982
H	-1.1204678875	1.0702165909	-1.9001500198
H	-2.174860872	1.0233552819	-0.4642848671
H	-0.7742798476	2.1215832267	-0.5057278015

Y5

P	-0.1729177628	0.1181227683	0.144555272
C	-0.0219587791	-0.0597023312	1.9700131088
C	1.5942692846	-0.0290090753	-0.4608759955
C	-0.9551199162	-1.388766094	-0.5534050713
C	2.2794151292	-1.2424960226	-0.5989672192
C	3.599367915	-1.242898862	-1.0633049944
C	4.2364348948	-0.050848203	-1.3897132884
C	3.5512070899	1.1664671675	-1.2593959061
C	2.2288954323	1.1732697946	-0.8024412849

H	1.7900341853	-2.1819856135	-0.3589467353
H	4.1346384376	-2.1826589862	-1.1747923974
H	5.2587918769	-0.0348702115	-1.7547355798
O	4.2550064644	2.2817017911	-1.6121057589
H	1.660284903	2.0950514253	-0.7227836249
C	-0.8144913568	-1.6548857819	-1.9262152783
C	-1.4778268206	-2.7465299884	-2.496226434
C	-2.2674993621	-3.5816725388	-1.6916520123
C	-2.3936556165	-3.3169736936	-0.3337090175
C	-1.7447396028	-2.2210562321	0.2442025569
H	-0.1826833416	-1.017105796	-2.5329391051
O	-1.4129781266	-3.0870210484	-3.8161818424
H	-2.7654182076	-4.4277539985	-2.1550194581
H	-3.0035632979	-3.9704129137	0.284423151
H	-1.8572343587	-2.0175303361	1.3038427294
C	-1.0830148046	0.4413076177	2.7436930405
C	-1.0481201162	0.3222970835	4.1364249388
C	0.041475695	-0.3071173771	4.7570339936
C	1.0862535635	-0.8006533286	3.9851158403
C	1.0669138722	-0.6796706824	2.5919843208
H	-1.9043745872	0.9234329411	2.2259925725
O	-2.0225560612	0.781346274	4.9736814401
H	0.0454890513	-0.3919840663	5.839441558
H	1.9307856984	-1.2807248348	4.472461109
H	1.8980136788	-1.0544251362	2.0056319011
C	-1.1167609206	1.4676316723	-0.2066139414
H	-1.5514141813	1.5363845003	-1.198972533
H	-0.8999669803	2.3999010118	0.3073652861
C	-3.1478869969	1.4309210061	4.3992855131
H	-3.7881092509	1.7131139586	5.2370855076
H	-2.8543461167	2.331547036	3.845007055
H	-3.7006564585	0.7617755598	3.7273711491
C	-0.6269988401	-2.2813103513	-4.6813402736
H	-0.7218599114	-2.7279393155	-5.6727374811
H	-0.9932906014	-1.2467788641	-4.7127675652
H	0.4296867061	-2.2800828713	-4.3832959727
C	3.615853023	3.5436967849	-1.4915126237
H	4.353692454	4.2831387137	-1.8082748481
H	2.7316763812	3.6127572663	-2.1385494803
H	3.31848964	3.7474911815	-0.4546363205

Y5H^+

P	0.0557141484	-0.1064402886	0.0535239485
C	0.094235379	-0.0534331851	1.8626694594
C	1.7374061231	-0.1113292298	-0.6167656913
C	-0.8564958141	-1.5605725236	-0.5217099742
C	2.2833645579	-1.2861584261	-1.1601889558
C	3.5876818442	-1.2540199714	-1.6498647787
C	4.3480254805	-0.0848161518	-1.6094548621
C	3.8007499204	1.0873727097	-1.0641091837
C	2.489267942	1.067569647	-0.5651891018
H	1.7033110766	-2.200664306	-1.2072570942
H	4.0222161636	-2.1535992412	-2.0752576079
H	5.3578737055	-0.0919090901	-2.0023191707
O	4.4386099353	2.274509171	-0.9709270493
H	2.1004857426	1.9887841558	-0.142877276

C	-1.2205648901	-1.6374659003	-1.8706314236
C	-1.9162319327	-2.7587256465	-2.3477941726
C	-2.2389066121	-3.7984246632	-1.4610510654
C	-1.8661596945	-3.7091406789	-0.1196370438
C	-1.1763578466	-2.6002429817	0.367191119
H	-0.9781873584	-0.8567615598	-2.584850927
O	-2.2222244994	-2.7349819647	-3.6634318239
H	-2.7770093654	-4.6726771653	-1.8079928386
H	-2.1221700973	-4.5194947371	0.5562209814
H	-0.9002550245	-2.5440984918	1.4139100303
C	-1.0794637005	0.2605657197	2.5568129468
C	-1.0772763832	0.297375065	3.9596628634
C	0.1092909582	0.0136571768	4.6545233416
C	1.2705109798	-0.3021901101	3.9485708957
C	1.2821688515	-0.3398139494	2.5556373923
H	-2.0155250347	0.4760769939	2.0512870749
O	-2.2585885964	0.6133981878	4.5338413534
H	0.134035529	0.0380693972	5.7375534282
H	2.1817841289	-0.5186835774	4.4978578852
H	2.1946910305	-0.57949172	2.0219456933
C	-0.8017741937	1.3884391218	-0.5524480118
C	-2.3382009072	0.6808058383	5.9588818271
H	-3.3708787378	0.949308026	6.1813936414
H	-2.1055609283	-0.2887108967	6.4147658314
H	-1.6659673208	1.4499551825	6.3568501176
C	-2.9365284697	-3.8365651916	-4.2271833801
H	-3.0649393868	-3.5925312863	-5.2816986372
H	-2.3658504578	-4.7680105902	-4.1339299344
H	-3.9195224672	-3.9536456489	-3.7562233489
C	5.7749197953	2.3825543822	-1.4649978673
H	6.0702035959	3.4162020144	-1.2848660753
H	6.4521152504	1.7093450429	-0.9263466777
H	5.8178324302	2.1716260416	-2.539963912
H	-0.8106524033	1.3927825664	-1.6454280035
H	-1.8318091915	1.3986428572	-0.1873723374
H	-0.287145255	2.2838148761	-0.1947646048

Y6

P	0.0125073922	0.1614966449	0.0239114922
C	0.0337621174	-0.0463169045	1.8440811921
C	1.710970676	-0.051399431	-0.6397921396
C	-0.877970921	-1.3791614694	-0.5444181257
C	2.2500914467	-1.2899893091	-1.0068689781
C	3.5571675866	-1.4019184112	-1.4845778584
C	4.350051702	-0.2539915307	-1.608365577
C	3.8200679319	0.9959937131	-1.2533072339
C	2.5214078932	1.0923516083	-0.7737827439
H	1.6455853312	-2.1882634017	-0.9375507108
H	3.9383271877	-2.3778862523	-1.7630145916
O	5.6345130295	-0.2449253418	-2.0628034928
H	4.4469224207	1.875484423	-1.3648544065
H	2.1019143168	2.0575947408	-0.5060394573
C	-1.7470417592	-1.2497886735	-1.639877687
C	-2.4496264901	-2.3391622874	-2.1371032278
C	-2.3089065726	-3.5986526624	-1.5349333529
C	-1.4581366057	-3.748705269	-0.4318875074

C	-0.7540262631	-2.6415256673	0.0502889413
H	-1.8784342755	-0.2675991983	-2.0892764599
H	-3.1228973604	-2.2417908968	-2.9834558766
O	-3.0453966046	-4.6044733023	-2.0880960769
H	-1.3424178578	-4.7091217974	0.0582101237
H	-0.1081877945	-2.774277473	0.9148298683
C	-1.1245250679	-0.4380174956	2.539034065
C	-1.1418298009	-0.4908020069	3.9265642666
C	0.0088810152	-0.161861248	4.6584003132
C	1.1731822616	0.2231748972	3.9819536783
C	1.1719507319	0.2828900379	2.5872217425
H	-2.0220616794	-0.7073443129	1.9900811642
H	-2.0350171594	-0.7857156001	4.4686210754
O	-0.1073618449	-0.2513998663	6.013674993
H	2.0764555068	0.4798144556	4.523493354
H	2.0779235821	0.5940463996	2.0757298969
C	-0.5372899009	1.7035348855	-0.3686188236
H	-0.4228356847	2.0477582101	-1.3930540651
H	-1.3810625522	2.0937210521	0.1925955083
C	1.016530425	0.0890274227	6.8108733357
H	0.6984440525	-0.0419869083	7.8467329571
H	1.870695579	-0.5704683135	6.6081206764
H	1.3211647137	1.1319659167	6.6538772898
C	-2.9607570557	-5.8983069841	-1.5120883728
H	-3.6258565311	-6.532056773	-2.1015394268
H	-1.9393834016	-6.2981984814	-1.5639823935
H	-3.2939221788	-5.8973256679	-0.4658541228
C	6.2257778138	-1.4782938854	-2.4422873763
H	7.2393398233	-1.2361271467	-2.7668473665
H	6.2728787539	-2.1796486903	-1.5988810084
H	5.6819280715	-1.948132748	-3.2721764741

Y6H^{+}

P	0.0529236913	-0.0936645549	0.0428201092
C	0.0522742604	-0.0470468969	1.8433802849
C	1.7370311966	-0.0754367453	-0.5959304284
C	-0.8407787708	-1.5369794902	-0.5591525829
C	2.2883151911	-1.2081285152	-1.2126392342
C	3.5924069255	-1.1940517986	-1.7023110354
C	4.3711344357	-0.032513079	-1.5780861361
C	3.8250470706	1.1088231639	-0.9555767367
C	2.5298349833	1.0877167483	-0.4733956406
H	1.6978354705	-2.1123035789	-1.320639922
H	3.9892385132	-2.0828079964	-2.1778091019
O	5.6361535685	0.0966435974	-2.0146415138
H	4.4431279609	1.9963414289	-0.8705128547
H	2.137441907	1.9803572184	0.0061827215
C	-1.2086283221	-1.6293485762	-1.920096114
C	-1.8800233286	-2.7416420084	-2.3920637913
C	-2.2064353698	-3.7995497587	-1.5188046534
C	-1.8439234145	-3.7181162152	-0.164883907
C	-1.1666437916	-2.5944192471	0.3030645717
H	-0.9644429974	-0.8328428213	-2.6175736467
H	-2.1706301508	-2.8237714186	-3.4340582726
O	-2.860170499	-4.8316599544	-2.0798240541
H	-2.085840706	-4.516703737	0.5259640551

H	-0.8971593495	-2.5453274342	1.3533082073
C	-1.1436658838	0.2202877474	2.5465704804
C	-1.1535206574	0.2520185288	3.9284127233
C	0.0327213541	0.0178782747	4.6540742473
C	1.2252074351	-0.2535960946	3.9645890144
C	1.2270100623	-0.2847384399	2.5719791868
H	-2.0739115069	0.398351805	2.0140570975
H	-2.0642672615	0.4591048802	4.4803223942
O	-0.0867468493	0.0766495905	5.9918202056
H	2.1491994551	-0.4369693859	4.4993667239
H	2.1578496971	-0.4918331762	2.0538052832
C	-0.8019346461	1.4042260797	-0.5683061739
C	1.0672444849	-0.1373703946	6.8084872652
H	0.719581782	-0.0359452071	7.8363240303
H	1.476682809	-1.1426783481	6.6567674928
H	1.8380769388	0.6147900289	6.6055344185
C	-3.2351236707	-5.9476937874	-1.2685173881
H	-3.7426644771	-6.6391349321	-1.9407711422
H	-2.3532285001	-6.4352748332	-0.8374281078
H	-3.9208957252	-5.6417058479	-0.4702688597
C	6.267715862	-1.0074130794	-2.6677614995
H	7.2667275002	-0.6588894208	-2.928753184
H	6.3434705939	-1.8709667719	-1.9970819375
H	5.7267064606	-1.2876792127	-3.5787038731
H	-0.8231193675	1.4017439604	-1.6612776269
H	-1.8280444209	1.4286399244	-0.1924733458
H	-0.2757749433	2.2988397815	-0.2250727486

Y7

P	0.0561064797	-0.0737383189	0.1406412945
C	0.0084338611	-0.0755225627	2.0065714466
C	1.8562388428	-0.164503367	-0.5030261418
C	-0.7767069421	-1.5840591269	-0.5980881947
C	2.4752618319	-1.3091328523	-1.0756191856
C	3.6821873788	-1.1660759501	-1.7750999025
C	4.331455609	0.0571494008	-1.917318222
C	3.760066452	1.1573107929	-1.2786872473
C	2.5521135548	1.0784139418	-0.5781481215
C	1.9796728949	-2.7386459342	-0.9433372222
H	4.1370193155	-2.0562224933	-2.2062580791
C	5.6073968435	0.1860528417	-2.7134370374
H	4.2739977483	2.1161785629	-1.315491417
C	2.1058810044	2.3563317858	0.109348563
C	-1.1093874931	-1.5316623608	-1.9788855083
C	-1.8395035879	-2.5755495647	-2.5566072944
C	-2.2751157362	-3.679301436	-1.8251234577
C	-1.9106520384	-3.7353677021	-0.4818695294
C	-1.1640376976	-2.7295069646	0.1468453769
C	-0.7177250309	-0.4053239054	-2.9147304259
H	-2.0701200941	-2.5185844426	-3.6189653514
C	-3.1036352492	-4.7677088815	-2.4637557439
H	-2.1976339007	-4.6059702279	0.104759493
C	-0.7981554518	-3.0023174436	1.5943292438
C	-1.2548725554	0.1683884195	2.6233249745
C	-1.312294353	0.3544454123	4.0103848365
C	-0.1866795505	0.298898248	4.8280652564

C	1.0252512023	-0.0166994309	4.2192618336
C	1.1535338165	-0.2202873638	2.838703783
C	-2.6028735705	0.1839020553	1.9197033908
H	-2.2848563317	0.5370074227	4.4631711743
C	-0.2782230843	0.5535806406	6.3129670405
H	1.9128754408	-0.1244468434	4.838972287
C	2.539058048	-0.6355609154	2.3799037105
C	-0.8279990415	1.3084896587	-0.2674226832
H	-0.9469681466	1.6149734226	-1.2972615889
H	-0.8249776826	2.1024519864	0.4707125421
H	-3.3777247778	-0.1253194072	2.6321776051
H	-2.8548104242	1.179944716	1.5481839887
H	-2.6415642624	-0.4772630173	1.0543708692
H	0.4831368613	-0.0078850439	6.8649720023
H	-0.1260862723	1.6172482394	6.540370721
H	-1.2611906204	0.2754316107	6.70781209
H	3.1412387281	-0.9037443652	3.2535226655
H	2.5203520468	-1.5024612857	1.7159678143
H	3.0688063947	0.1581763466	1.8470450289
H	-0.6263106893	-0.7865232768	-3.9369650785
H	-1.4860616078	0.3759950486	-2.9225269001
H	0.2309860064	0.067429139	-2.6517388206
H	-0.9078669957	-4.071424513	1.8019170143
H	0.2293986521	-2.7245800132	1.8379997498
H	-1.4427847608	-2.4653846871	2.2970105424
H	-2.9826137121	-5.7231843738	-1.9423905906
H	-4.1726539144	-4.5167905452	-2.4401862659
H	-2.8303230066	-4.915878514	-3.5142967003
H	2.9489452327	3.0544295724	0.1586190975
H	1.2813427675	2.8375044819	-0.4231019623
H	1.7557967585	2.185751851	1.1315310597
H	2.8178679587	-3.4254139498	-1.1012509081
H	1.5664422814	-2.9536531976	0.0441408236
H	1.2073149068	-2.9894530501	-1.6760419808
H	6.2884969769	0.9181085551	-2.2653039625
H	6.1349444285	-0.7710312408	-2.7844932276
H	5.4022342573	0.5221424161	-3.7388085678

Y7H^+

P	0.1485024975	-0.2576106758	0.0947910952
C	0.0628491227	-0.133940085	1.9334139723
C	1.8640230799	-0.1263473857	-0.5691473155
C	-0.8015243991	-1.6847892173	-0.5850872934
C	2.5269918153	-1.2178319253	-1.1981086812
C	3.7734445435	-0.9877992855	-1.7896897671
C	4.4070628847	0.2552835396	-1.7710275695
C	3.7722302954	1.2884533908	-1.0810042513
C	2.5235547583	1.1330103321	-0.4736055101
C	2.0249229758	-2.6474420571	-1.2515359092
H	4.2738361572	-1.8243457388	-2.2706297893
C	5.7324675331	0.4687510313	-2.4558486269
H	4.2691931678	2.2519144853	-0.9971541922
C	2.0058238791	2.3378930422	0.2926580845
C	-1.1974385972	-1.6379480539	-1.9530448037
C	-2.0330615784	-2.642299312	-2.4480477652
C	-2.4889662704	-3.6989223617	-1.6585822336

C	-2.026272999	-3.7631958275	-0.3438921233
C	-1.1834701856	-2.7974408922	0.2161057917
C	-0.7396947864	-0.6006512192	-2.9637902188
H	-2.3245826514	-2.599988018	-3.4947716224
C	-3.4297525784	-4.7392585473	-2.2096600688
H	-2.3166700396	-4.6090230174	0.2740931367
C	-0.7174600355	-3.0697768372	1.6330209276
C	-1.1809746678	0.2318475893	2.5240395161
C	-1.2329320555	0.4553835781	3.9025091849
C	-0.1162681126	0.3244062152	4.7287227142
C	1.0723479175	-0.1052419869	4.1374116895
C	1.1960914697	-0.3537175282	2.7666421327
C	-2.502339235	0.351672768	1.7848322879
H	-2.1883263332	0.7285164232	4.3440787988
C	-0.1947296498	0.6192405546	6.2046343859
H	1.9415534714	-0.2721751821	4.7683007571
C	2.5405404644	-0.8869065567	2.3113851774
C	-0.7264075016	1.2493380415	-0.5337322466
H	-3.3207622174	0.1102673924	2.4686278969
H	-2.6850270798	1.3708767073	1.4261284293
H	-2.588152991	-0.3286736713	0.9340532193
H	0.5139614022	0.0105510789	6.7743292205
H	0.0478649005	1.6717683368	6.4007005811
H	-1.1996864437	0.438057382	6.5976832985
H	3.09171804	-1.2594633458	3.1787482498
H	2.4546142565	-1.714853287	1.6033321472
H	3.1599087094	-0.1199823725	1.8369273502
H	-0.7147788324	-1.0536528153	-3.9587253074
H	-1.4278634298	0.2500253037	-3.0244789598
H	0.2625600629	-0.2129355329	-2.7659586307
H	-0.8749526038	-4.1261895371	1.8658797071
H	0.3445740757	-2.8609975801	1.7805852129
H	-1.267225927	-2.4867153382	2.3780632508
H	-3.2959069164	-5.7053681248	-1.7137471168
H	-4.4732121028	-4.4348374767	-2.0558629779
H	-3.2874942812	-4.8805341067	-3.2854035151
H	2.8529094456	2.9241444908	0.6594048396
H	1.4129398238	3.006914834	-0.3412918947
H	1.4022078845	2.0713111015	1.16339156
H	2.8571806526	-3.3114056814	-1.4991006949
H	1.6095933851	-2.9907044461	-0.3008741374
H	1.2529313686	-2.7952496145	-2.0124355106
H	6.312349357	1.2601705048	-1.9715232068
H	6.3336196423	-0.4455943871	-2.4619463404
H	5.5822308915	0.7668852188	-3.5016466198
H	-0.2078884086	1.6348739647	-1.4106910854
H	-1.7500897063	0.9951465168	-0.8051773596
H	-0.7413523149	2.016654205	0.2392527296

Y8

P	-0.0274561251	0.195217408	-0.1320081036
C	-0.0962938016	0.1706695639	1.7069741191
C	1.7175143605	-0.0786956913	-0.6733458962
C	-0.8331167622	-1.4362371156	-0.6029482401
C	2.2482179109	-1.344208099	-0.9637876863
C	3.5854044802	-1.4792209532	-1.3408174359

C	4.4043095195	-0.3528027261	-1.437803308
C	3.8834239504	0.911680665	-1.1544161983
C	2.5491080098	1.048300293	-0.7715782254
H	1.6176898714	-2.2249707458	-0.9027835648
H	3.9848607869	-2.464868579	-1.5649168121
H	5.444349184	-0.4598605226	-1.7351829122
H	4.5159265958	1.7921340519	-1.2323128345
H	2.1282279165	2.0250042302	-0.5503771706
C	-1.4842615371	-1.5032962288	-1.8432755833
C	-2.1255718809	-2.6739987553	-2.2501967412
C	-2.1356227433	-3.792089443	-1.4133864423
C	-1.5017869023	-3.7340092062	-0.1706008183
C	-0.8539654837	-2.5640497229	0.2318883938
H	-1.4987395838	-0.6271573099	-2.4871390229
H	-2.623109183	-2.7116037811	-3.2160295564
H	-2.6415374425	-4.7021843936	-1.7250594695
H	-1.5148921762	-4.5985233443	0.4885797966
H	-0.3696385676	-2.5290981478	1.2043503812
C	-1.3435844593	0.2584967128	2.4193926692
C	-1.3139587966	0.2777684671	3.8561363773
C	-0.0683552788	0.2014324516	4.5335402294
C	1.1077190117	0.1059719598	3.8304174368
C	1.0895058756	0.0894634382	2.4168175697
C	-2.6142819315	0.3201777643	1.7817877157
H	-0.0642436586	0.2176335187	5.6209484096
H	2.0588292316	0.0436325955	4.3518803507
H	2.0326591144	0.0179126974	1.8856124953
C	-0.5877470946	1.6827222799	-0.6886659257
C	-0.4706927312	2.1898767318	-2.109478498
H	-1.3225022764	2.1560660789	-0.0435231378
H	-0.2835410437	3.273721499	-2.1223102954
H	0.3669393604	1.724067269	-2.6446323251
H	-1.3694229244	2.0260521578	-2.7285660492
C	-3.7785505548	0.4049693229	2.513316032
H	-2.665118576	0.3119235009	0.6986277633
H	-4.7341998935	0.4503838279	1.9982436073
C	-3.7423792221	0.4330993523	3.9264689259
C	-2.5336669732	0.3695998772	4.5793562556
H	-4.6679976394	0.5030031856	4.4913244026
H	-2.4906739363	0.3886348653	5.6659053216

Y8H^{+}

P	0.1903673493	0.0816113415	0.0367205782
C	0.1407871784	0.1033588862	1.8511377633
C	1.8979585827	-0.1604341769	-0.5354812092
C	-0.8145659373	-1.2787456298	-0.622492601
C	2.3909038408	-1.4672927036	-0.6920980567
C	3.7040866078	-1.6692317495	-1.1136303383
C	4.5292253365	-0.5761006322	-1.3863453439
C	4.0424891226	0.7241745883	-1.237119797
C	2.7318649778	0.9364968166	-0.8118506152
H	1.7522995626	-2.3230807382	-0.4968428293
H	4.078767053	-2.6807670968	-1.2363109156
H	5.5500461761	-0.7372542616	-1.7196067225
H	4.6808488691	1.5752019972	-1.4536420415
H	2.3716833809	1.9538833182	-0.7013373282

C	-1.1863371111	-1.2662175615	-1.9783883331
C	-1.9187161323	-2.3275161891	-2.5059585962
C	-2.2776509651	-3.404421888	-1.6908222362
C	-1.9046421942	-3.4223726028	-0.3458243876
C	-1.174401453	-2.3628164514	0.1932734665
H	-0.9044316968	-0.4404434662	-2.6257380535
H	-2.2075403717	-2.3141611578	-3.5524091653
H	-2.8486468111	-4.2296457267	-2.1056940667
H	-2.1847753167	-4.258310507	0.2877556507
H	-0.8962252242	-2.3768687774	1.2423426458
C	-1.0888606897	0.2958498844	2.5783540886
C	-1.014816727	0.3501992041	4.0124533924
C	0.2411552282	0.2048884706	4.6590898729
C	1.3962424626	0.0126395326	3.9387528414
C	1.3443570194	-0.0347790973	2.529762081
C	-2.3673298965	0.4355672699	1.9746437143
H	0.2733966041	0.2484464804	5.7445325502
H	2.3511769599	-0.099804789	4.4417575007
H	2.2675106541	-0.1801549057	1.9798558408
C	-0.444501523	1.6745005546	-0.667859975
H	-1.5152015216	1.5332514173	-0.8447464347
C	-0.2109601307	2.9240243458	0.1955957465
C	-3.5000996738	0.6285909841	2.737007788
H	-2.4727814212	0.3687434618	0.8970990824
H	-4.4645984611	0.7302002544	2.2483865374
C	-3.4208802597	0.6890610203	4.1463806484
C	-2.2014055067	0.5489536992	4.7672004848
H	-4.3215873414	0.8413555488	4.732730265
H	-2.1258608572	0.5878090011	5.8504342615
H	0.0266024732	1.7801194233	-1.6514820691
H	-0.5850570955	3.8002342651	-0.3425967272
H	-0.7460518967	2.8566606499	1.1457764063
H	0.8466057763	3.0947236932	0.4156906356

Y9

P	-0.0497821851	-0.2655802774	-0.939134072
C	1.4474222803	-0.9380233706	0.0180622605
C	-1.4560461537	-1.2776136177	-0.3086320483
C	-0.2704843988	1.4901576372	-0.325128946
C	2.693258734	-0.083490831	-0.2950658368
H	1.2528137156	-0.8885177214	1.0990750682
C	3.970944952	-0.6621535065	0.3365597739
H	2.8158040999	-0.0324378292	-1.3868277377
H	2.5527669576	0.9479301144	0.0524076462
C	4.1958989541	-2.1238070284	-0.0722312434
H	4.8346466591	-0.0476973699	0.0504934243
H	3.8944297461	-0.6004981692	1.4325189002
C	2.9668609069	-2.9810392057	0.2579630658
H	4.3901828328	-2.171483654	-1.153835167
H	5.0880814286	-2.5258242551	0.4248227479
C	1.6892732395	-2.4094997436	-0.3781071254
H	3.1186678884	-4.0137155445	-0.0822333212
H	2.8406926526	-3.0281583019	1.3501115738
H	1.7710908837	-2.465723112	-1.4734247849
H	0.8269023568	-3.0258907259	-0.0982418408
C	-2.522824857	-1.5426361747	-1.1809347197

C	-3.6253666243	-2.2820453774	-0.7510826644
H	-2.4658112951	-1.1565043693	-2.1945928398
C	-3.6848087799	-2.7561273732	0.5603213013
H	-4.4401491116	-2.4848073534	-1.4414188742
C	-2.6333016654	-2.4922372631	1.4400351697
H	-4.5457942784	-3.3279439164	0.8967495778
C	-1.5255028199	-1.7613944642	1.0083672901
H	-2.6719710784	-2.8587072931	2.4625628518
H	-0.7144688893	-1.5757810295	1.7058535664
C	-1.5584753926	2.1177626093	-0.9001479825
H	0.58733994	2.0308283562	-0.7536012503
C	-1.6981989511	3.5901543614	-0.4831127446
H	-1.5618068708	2.0163280256	-1.9889117677
H	-2.4260880164	1.5522817863	-0.5315300892
C	-1.6466033877	3.7559202493	1.0422706128
H	-2.636619769	3.9985835147	-0.8795523549
H	-0.8862136647	4.1758359845	-0.9396699789
C	-0.3738431186	3.1250073551	1.6231324498
H	-2.5269934843	3.2705498831	1.4889248151
H	-1.702211011	4.8173870514	1.3157953039
C	-0.2240723638	1.6502579981	1.2085648619
H	-0.3732502164	3.2013402572	2.7181696799
H	0.5032536254	3.688059861	1.2709117225
H	0.7117211506	1.2517638403	1.6170633804
H	-1.0401427716	1.0664531214	1.6571633085
C	-0.0637096223	-0.2512581893	-2.6288618951
H	0.4901403055	0.547302023	-3.116390861
H	-0.0795615321	-1.2048639618	-3.149988207

Y9H$^+$

P	-0.0737981365	-0.259996205	-0.7818824844
C	1.4794606203	-0.9639489757	-0.0593877896
C	-1.4918689216	-1.2649751696	-0.2673181712
C	-0.2967474849	1.5103791103	-0.2725708649
C	2.721452216	-0.0881174481	-0.3524029066
H	1.3091232087	-0.960218491	1.0263149377
C	3.9713572022	-0.6857699305	0.3203907924
H	2.8883842395	-0.0362877703	-1.4372615584
H	2.5704737736	0.9390127029	-0.0007675494
C	4.2036533067	-2.1443261271	-0.0958012204
H	4.8413581774	-0.0695164872	0.067552789
H	3.8531657233	-0.6265635707	1.4115960297
C	2.9628688768	-3.0065317997	0.1727488285
H	4.4507295413	-2.1837403059	-1.1662831538
H	5.06852102	-2.552815836	0.438248825
C	1.7083096605	-2.4282308784	-0.5055976383
H	3.1212896762	-4.0305696256	-0.1833125354
H	2.7872339361	-3.0760221221	1.2555799268
H	1.8435466762	-2.4610756477	-1.5952256154
H	0.8338384688	-3.0446969182	-0.270230674
C	-2.5077480324	-1.5949907868	-1.1805593928
C	-3.6072258486	-2.3441285014	-0.762406454
H	-2.4514472145	-1.2788381139	-2.2171605016
C	-3.7029217105	-2.7693154468	0.5633591569
H	-4.3857353026	-2.5971308076	-1.4754421328
C	-2.6965133043	-2.4466306571	1.4765656496

H	-4.559095245	-3.3545768919	0.8848092916
C	-1.5945788631	-1.6980560937	1.0677364147
H	-2.7665180684	-2.7801064269	2.5073060827
H	-0.8196209098	-1.4619693069	1.7908093674
C	-1.5884918833	2.1189695243	-0.8690299969
H	0.567509126	2.027959341	-0.7150445799
C	-1.717439093	3.6033971224	-0.483350206
H	-1.5971132082	2.0251179952	-1.9612419529
H	-2.457773336	1.5656672231	-0.4902229858
C	-1.660886166	3.8035849039	1.0368522336
H	-2.6563345492	3.996511764	-0.8890834724
H	-0.9074721366	4.1720043466	-0.9614445842
C	-0.3888519035	3.1855144566	1.6317337447
H	-2.5436834321	3.3406888829	1.5001977131
H	-1.7066761849	4.8714510663	1.2771066439
C	-0.2499502792	1.6974423509	1.2623848036
H	-0.3865755057	3.2839205636	2.7230721651
H	0.4925713342	3.7310915466	1.2664498025
H	0.6852128441	1.3007305485	1.6758712812
H	-1.0731097664	1.1333496249	1.7210206422
C	0.062277641	-0.3045592107	-2.6022674709
H	-0.8032426732	0.1699255308	-3.0693433339
H	0.9603818477	0.2402045188	-2.9066162059
H	0.1355610431	-1.3370275711	-2.9523796903

Y10

P	-0.1021465685	0.0124545343	-0.099624618
C	-0.0073757759	-0.0516966883	1.775004028
C	1.6702351055	-0.0071721395	-0.5714367586
C	-0.8700031264	-1.612244277	-0.5226635218
C	2.158157348	-0.9035028546	-1.5321058709
C	3.4932139226	-0.8489045868	-1.9344161626
C	4.3519071266	0.1076344308	-1.3897138669
C	3.8736976464	1.0056373216	-0.4338975563
C	2.5428762796	0.9432988747	-0.0186210681
H	1.4946488033	-1.6433980048	-1.9692356266
H	3.8611119633	-1.5530091803	-2.6760413316
H	5.3904427081	0.1515740641	-1.7070644311
H	4.5376529184	1.7515999978	-0.0050856055
H	2.1842530459	1.6330651892	0.7400191764
C	-1.3346268114	-1.7820269016	-1.8373350947
C	-1.9025439458	-2.9916554771	-2.2377494092
C	-2.0130474483	-4.047081383	-1.3301284692
C	-1.5583660333	-3.8862614096	-0.0202682674
C	-0.9923085907	-2.6758837051	0.3834499667
H	-1.2510346522	-0.9492009067	-2.5298041739
H	-2.2612509406	-3.1085193001	-3.2570596901
H	-2.4555711833	-4.9899543019	-1.6411040216
H	-1.6491499697	-4.7019604738	0.6922777429
H	-0.6553753338	-2.5592588491	1.4077654272
C	-0.9569232373	0.6886437643	2.4921479378
C	-0.9409701997	0.7088999367	3.8880128864
C	0.0373715429	-0.002444384	4.5849473404
C	0.9986384358	-0.7330749962	3.8821968596
C	0.9762421931	-0.7580968924	2.4864584028
H	-1.7025122064	1.2631429582	1.9481186406

H	-1.6859043731	1.2854402057	4.4305793869
H	0.0566841724	0.0182741834	5.6715204169
H	1.7682997076	-1.2800399331	4.4212812104
H	1.7341397731	-1.3230812349	1.9493918176
C	-0.8591746902	1.2646575749	-0.9307745797
C	-2.3532448249	1.4917189859	-1.009740468
H	-0.2045408786	2.1088077075	-1.1374152902
H	-2.6387081903	1.8710943047	-2.0020472688
H	-2.9155025457	0.561060396	-0.8596323997
H	-2.7466371662	2.2179864502	-0.2780396901

Y10H$^+$

P	0.0297069225	-0.0952840152	0.0179147477
C	0.0618312135	-0.1021726636	1.8317756745
C	1.7219683443	-0.0324248853	-0.6253564933
C	-0.8095709425	-1.5754447546	-0.6112562607
C	2.3979259861	-1.2160224876	-0.967648082
C	3.7209999946	-1.1578015624	-1.405304825
C	4.3737060188	0.0722427282	-1.5054197554
C	3.7071579279	1.2510134333	-1.1619086005
C	2.3868206825	1.2033583294	-0.719430265
H	1.8964042832	-2.1759914564	-0.8993603268
H	4.238929748	-2.0742510146	-1.6707989766
H	5.4022867269	0.113259955	-1.850967721
H	4.2139083457	2.2080018692	-1.238468391
H	1.886342335	2.1280864069	-0.4485338495
C	-0.7600595702	-1.8750166713	-1.9864637411
C	-1.4445441927	-2.9834483859	-2.4797605196
C	-2.177729496	-3.798358781	-1.6128040266
C	-2.2235631617	-3.5085213966	-0.2486699045
C	-1.5418949831	-2.4012266394	0.2567316207
H	-0.178332162	-1.2611960781	-2.6671751738
H	-1.4018406726	-3.2127511639	-3.5400818682
H	-2.7081405509	-4.6624326206	-2.0016014461
H	-2.7855298207	-4.1459763803	0.4268960995
H	-1.5759310906	-2.1895755914	1.3201309412
C	-1.0830228202	0.2612039273	2.5647110511
C	-1.0519213783	0.238721151	3.9577142017
C	0.1136862817	-0.1428334271	4.6269047102
C	1.2514457538	-0.5038858192	3.9034708804
C	1.2311251805	-0.4847737874	2.5093420282
H	-1.9987391659	0.5583910187	2.0623660267
H	-1.9367586492	0.5222337212	4.5192289769
H	0.1344609554	-0.1559499314	5.7124717814
H	2.1586998528	-0.7971505602	4.4225376671
H	2.1230372761	-0.7585760112	1.9553201376
C	-0.8848113876	1.4159496651	-0.5117146089
C	-0.9924340729	1.6318078478	-2.0272421897
H	-0.3930959535	2.2641176856	-0.0214165189
H	-1.5261177397	2.5679597045	-2.2162995129
H	-0.0091875391	1.7052275148	-2.5010946405
H	-1.5536184426	0.826958265	-2.5093379584
H	-1.8828690371	1.3357578614	-0.066691889

Y11

P	-0.1369969384	0.0690734451	-0.042419441

C	-0.140521605	-0.0038226401	1.8057317842
C	1.705504979	-0.04530342	-0.4954238244
C	-0.8442262404	-1.5202406466	-0.6477725683
C	-2.1777235696	-1.5569940307	-1.0804012343
C	-2.750679614	-2.7543026542	-1.5095443227
C	-2.008075209	-3.9361718658	-1.4896807567
C	-0.6867591941	-3.9148406471	-1.0404249866
C	-0.1083545255	-2.7152203563	-0.6242758628
H	-2.7565110483	-0.6391384254	-1.0650912551
H	-3.7823464949	-2.7644496075	-1.851537906
H	-2.4580601672	-4.8703250095	-1.8154626345
H	-0.1035326511	-4.8315876226	-1.0147691207
H	0.921101039	-2.7193517158	-0.2790074699
C	-1.2807771708	0.468792689	2.4729211048
C	-1.3771138895	0.3871225551	3.8620578833
C	-0.3411015084	-0.1820721554	4.6047097844
C	0.7922576885	-0.6681054679	3.9511086113
C	0.8926172462	-0.5785691795	2.5618599264
H	-2.0868878506	0.8950233964	1.8830806878
H	-2.2643828357	0.7642727397	4.3639248212
H	-0.4175900191	-0.2507752367	5.6867264607
H	1.6016169937	-1.1161889001	4.5216523859
H	1.7840635874	-0.9619606839	2.0758567907
C	-1.1119331007	1.3370948771	-0.5919308842
C	-0.9993799489	2.7734305319	-0.1211118634
H	-1.4871323751	1.1843330185	-1.602796313
C	2.446742524	1.211787602	0.0107936633
H	2.1596656539	-0.9308121982	-0.0263024894
C	3.924804775	1.2143365536	-0.4137176517
H	1.9526065224	2.101757033	-0.4033334175
H	2.3719709145	1.2947532532	1.1011567587
C	4.0755649844	1.0798669366	-1.9350477918
H	4.4093348644	2.1341821782	-0.0613353791
H	4.4460081625	0.3793481094	0.0784729561
C	3.338211567	-0.1616786691	-2.4550764575
H	3.6610096354	1.9762703626	-2.4193898386
H	5.1368384536	1.0371647159	-2.2118428791
C	1.8614704761	-0.1732694787	-2.0253070347
H	3.4043568821	-0.214126738	-3.5496378131
H	3.8344125733	-1.0652644551	-2.0700414802
H	1.3346286704	0.6654460294	-2.5029467807
H	1.3785404872	-1.0883062807	-2.3862606671
H	-1.9785978031	3.2727167312	-0.1626705139
H	-0.6635595228	2.8371480306	0.9217738667
H	-0.3085873977	3.4002922964	-0.7103868475

Y11H^{+}

P	-0.004902684	-0.2235740786	-0.6960667464
C	1.5147457575	-0.9112448469	0.0197530694
C	-1.4220542514	-1.2267017557	-0.1724868246
C	-0.2328886791	1.5436340448	-0.1805935505
C	2.5839588955	-0.0769523895	0.3887915465
C	3.7733474602	-0.6341437473	0.8576407221
H	2.5000209742	1.0026689295	0.3175985785
C	3.9072622665	-2.0201893003	0.9560804529
H	4.5931153655	0.0162677722	1.1465679912

C	2.8507490868	-2.854621348	0.5842182248
H	4.8344444682	-2.4502348099	1.322693498
C	1.657401329	-2.3071007962	0.1169062018
H	2.9532234627	-3.9327799982	0.6597812518
H	0.8395488144	-2.9654800162	-0.1601749022
C	-2.4771305607	-1.5038458072	-1.0590175832
C	-3.583031217	-2.2323490657	-0.6213579946
H	-2.4476953508	-1.164665529	-2.0892564325
C	-3.6476245008	-2.6852789607	0.6971391892
H	-4.391321489	-2.4469594911	-1.3135363652
C	-2.6028581821	-2.4129668027	1.5835290292
H	-4.5097765687	-3.2531930361	1.0335749894
C	-1.492882607	-1.6875772964	1.155509063
H	-2.6490873716	-2.768228835	2.6083663968
H	-0.6805601017	-1.4943047202	1.8490943449
C	-1.4180112985	2.2011040521	-0.9281674241
H	0.6916087547	2.0513044067	-0.4919718221
C	-1.5662514174	3.6774156637	-0.5160213575
H	-1.2785532644	2.1385446115	-2.0137993103
H	-2.3430183117	1.6597408091	-0.68861784
C	-1.7048165416	3.8368358609	1.0035421198
H	-2.4348128141	4.1046989771	-1.0295777275
H	-0.6885304381	4.2382479375	-0.8670768078
C	-0.5351405601	3.1720590551	1.741052238
H	-2.6495426273	3.3823544924	1.3339086117
H	-1.7608429846	4.8991198957	1.265541442
C	-0.3880065145	1.6905428479	1.3512966147
H	-0.6736833947	3.2431479847	2.8257271123
H	0.3986261457	3.7042753714	1.5101344002
H	0.467295441	1.2462975097	1.8726942279
H	-1.2821681074	1.1424632918	1.6750747973
C	0.1455264597	-0.3094445586	-2.5304211008
H	-0.7932650626	0.0471034823	-2.9660765421
C	1.3362082734	0.4786651401	-3.0975081038
H	0.2319347588	-1.3743028083	-2.7754378134
H	1.3822924637	0.3279520574	-4.1799334721
H	1.2379650614	1.5539931554	-2.9192420523
H	2.2857736622	0.1410766487	-2.6726703409

Y12

P	-0.0399874064	-0.2514694121	-0.9465321428
C	1.4542847285	-0.9172672724	0.0318718176
C	-1.4482914424	-1.2689623793	-0.331935131
C	-0.2611988039	1.5005923069	-0.3037974662
C	2.7039291636	-0.064948459	-0.2771701368
H	1.256228652	-0.8596450943	1.1119592747
C	3.9713529797	-0.6330344991	0.3843534274
H	2.8495904846	-0.0319482032	-1.3664028901
H	2.5581710724	0.9721705486	0.0501488389
C	4.2060803536	-2.0983668879	-0.0056961687
H	4.8392578385	-0.0199216468	0.108218212
H	3.8733039347	-0.5599750601	1.4778879428
C	2.9731278978	-2.9553519084	0.3107076797
H	4.4196130533	-2.1558053004	-1.0832250971
H	5.0901734574	-2.4937769291	0.5107344673
C	1.7037351692	-2.3911625745	-0.348529617

H	3.1325367075	-3.9901109671	-0.019698182
H	2.8303394218	-2.9952767648	1.4011235013
H	1.7986574751	-2.4580147929	-1.4421279735
H	0.8389608033	-3.0071043183	-0.0753212633
C	-2.5331740924	-1.5045733694	-1.1908646876
C	-3.6354331298	-2.2432274253	-0.7597870133
H	-2.4931799902	-1.0971137033	-2.1962977433
C	-3.6806528912	-2.743106631	0.542664705
H	-4.4624047665	-2.4232869084	-1.4418361495
C	-2.6140521926	-2.5053378782	1.4109766368
H	-4.5424900421	-3.3125359626	0.8808272903
C	-1.5053267494	-1.7772299248	0.9766781443
H	-2.6408720787	-2.8899909376	2.4271742173
H	-0.683584289	-1.6125594685	1.6662806625
C	-1.5468846674	2.1322282346	-0.8786677133
H	0.59789537	2.0558279878	-0.7087675531
C	-1.7031446775	3.5947861528	-0.4327869842
H	-1.5414363322	2.0572813703	-1.969793913
H	-2.4143060294	1.5526287269	-0.5323986877
C	-1.6692654237	3.7287249898	1.0959286446
H	-2.6405248957	4.004043992	-0.830954065
H	-0.891078497	4.1961780836	-0.8682212275
C	-0.3967329497	3.0972334112	1.6760789305
H	-2.5497567119	3.2258986739	1.5226650369
H	-1.7377812914	4.7836796107	1.390847221
C	-0.2299472996	1.6323656838	1.2334007466
H	-0.4071903573	3.1512221209	2.7724374463
H	0.47851865	3.6751673508	1.3438505993
H	0.706242942	1.2358984905	1.6423478298
H	-1.0435758952	1.0323413378	1.6644938445
C	-0.0534375181	-0.2664148982	-2.6372940252
C	0.6951173954	0.7147441635	-3.5161889603
H	-0.2755853804	-1.2402648228	-3.0715799136
H	0.1365854079	0.9132461832	-4.4431670037
H	0.8280422628	1.6900918687	-3.0288746029
H	1.7019205803	0.3873151115	-3.828600806

Y12H+

P	-0.0523331755	-0.2578586508	-0.801531863
C	1.4917698606	-0.9834099457	-0.0684543008
C	-1.4643771371	-1.2574932728	-0.2340161657
C	-0.2611828758	1.5111748609	-0.2801078649
C	2.7382044652	-0.069352416	-0.1368975269
H	1.2402561487	-1.1114810484	0.9935516683
C	3.9231885609	-0.7369138141	0.5860696947
H	3.0132130767	0.1150018117	-1.1819923814
H	2.5330394116	0.9068969183	0.3163799773
C	4.2229188793	-2.1346137365	0.0264773575
H	4.8038220872	-0.0905796686	0.4999961787
H	3.6937998216	-0.8108424509	1.6586981782
C	2.978713216	-3.031998913	0.067578833
H	4.5748181867	-2.0450859485	-1.0112728949
H	5.0390109807	-2.5974226042	0.5921552862
C	1.7855323293	-2.3845056957	-0.6568661196
H	3.1904567462	-4.0049700763	-0.3897284211
H	2.7003065244	-3.2314946919	1.1119959534

H	2.0232814604	-2.2882509646	-1.7250389896
H	0.9028256995	-3.029891822	-0.5789773631
C	-2.5099096675	-1.5947354182	-1.1113717807
C	-3.5963913309	-2.341744737	-0.6568298255
H	-2.4893498159	-1.287299772	-2.1509632346
C	-3.6541328404	-2.7592008346	0.6730759354
H	-4.3949487867	-2.5981631384	-1.3461258787
C	-2.6213982836	-2.4285021705	1.5527762152
H	-4.5002267221	-3.3424703969	1.0235013643
C	-1.5321344794	-1.6828164362	1.10621381
H	-2.6604299807	-2.7527657211	2.5881195516
H	-0.7422950793	-1.4403351438	1.8099412089
C	-1.5031726757	2.1609233117	-0.9354507243
H	0.6381101725	2.0179478723	-0.6596615929
C	-1.626974092	3.6385549272	-0.5213402671
H	-1.448360752	2.0935156114	-2.0283719591
H	-2.404270678	1.6164518196	-0.6232039157
C	-1.6487217897	3.8055337539	1.0036266543
H	-2.5350875823	4.0588667041	-0.9679230311
H	-0.7821712901	4.202183658	-0.9417451009
C	-0.4216386111	3.1511039334	1.6514282653
H	-2.5627497924	3.3487834599	1.4088781095
H	-1.6894201669	4.8688296932	1.2643246581
C	-0.2967313853	1.6679973818	1.258905294
H	-0.4751775191	3.2281164899	2.7432323529
H	0.4884201075	3.6853914967	1.3441039166
H	0.6028195306	1.2377287256	1.7157742186
H	-1.1578915819	1.1167403753	1.6583126872
C	-0.0196217673	-0.3385122817	-2.6429648455
H	-0.9961786619	0.0210211634	-2.9821338173
C	1.0932867898	0.4557385607	-3.3457841636
H	0.0322925679	-1.3996783032	-2.9094886139
H	0.9322229828	0.406767838	-4.4268365563
H	1.0933168087	1.5125098025	-3.0622818047
H	2.0846811058	0.0439979042	-3.1433293665

Y13

C	-1.8312111964	-0.0157721136	-0.6317215977
C	-0.4552725637	-0.4002242646	-0.9025919935
P	0.9489034147	-0.1621651467	0.002288961
N	1.0366142683	1.396535511	0.6688732053
C	0.542082866	2.5326958802	-0.1135809768
N	1.2846450139	-1.116165559	1.4153264957
C	1.3387464441	-2.5620921627	1.1421058412
N	2.2750327095	-0.5939164117	-0.970807272
C	2.3062566591	-0.2164724799	-2.3848407983
C	0.3991499602	-0.8696263389	2.5663164017
C	3.6205426044	-0.7290974189	-0.4126415517
C	2.1202606117	1.7796855301	1.5759847344
H	-0.3079729007	-1.111916006	-1.7098012642
H	-0.6298959738	-1.2146512275	2.3834941283
H	0.3667971378	0.1914203919	2.8138414028
H	0.7954419182	-1.4146910552	3.4311090283
H	0.3494449011	-2.9725298179	0.8859230035
H	2.020935962	-2.7754889461	0.3185069811
H	1.7052713146	-3.0727178372	2.0397712554

H	3.5741308603	-0.9673119305	0.6494711566
H	4.2059890253	0.195274484	-0.5420592886
H	4.1550793299	-1.5362789931	-0.9316744878
H	1.2972175544	-0.0477558148	-2.7583338219
H	2.8959099458	0.701050856	-2.5430771692
H	2.7723772991	-1.0206511983	-2.9695401727
H	2.9528849067	2.2526724478	1.0314671736
H	1.747076347	2.5055529465	2.3105009011
H	2.5032335628	0.9091614173	2.1084123555
H	1.3432361806	2.9840339404	-0.720354863
H	0.1597835918	3.3012328995	0.5696068197
H	-0.2672888099	2.218623294	-0.7708845447
C	-2.2650016002	0.7643026361	0.4689218569
C	-3.606864474	1.0948762479	0.6478167844
C	-4.5828802804	0.6656131447	-0.2531296865
C	-4.180664501	-0.1087184903	-1.3456104889
C	-2.842893259	-0.4387003681	-1.5310445104
H	-1.5388210349	1.109857132	1.1987433155
H	-3.8911611978	1.6951579981	1.5100901563
H	-5.6278982642	0.9250306093	-0.108313262
H	-4.9183392677	-0.4598731658	-2.0644618194
H	-2.5565230659	-1.04070762	-2.3913563892

Y13H$^+$

C	-1.813405557	-0.0818322076	-0.7891310448
C	-0.4455083688	-0.4404310992	-1.3372468751
P	0.9446369813	-0.1259878335	-0.1633839483
N	1.0970873231	1.5050116187	0.0909030011
C	1.1502366744	2.4323694017	-1.0506785903
N	0.6827766674	-0.7913867137	1.3360338503
C	1.1972070315	-2.1215349455	1.6939169522
N	2.310246888	-0.7630575925	-0.867003093
C	2.3576607266	-1.8708126483	-1.8346724012
C	-0.3608546444	-0.3015923654	2.2576162245
C	3.6312431292	-0.4271185944	-0.3075756025
C	1.5951610251	2.1083884403	1.3383481497
H	-0.4176330064	-1.5136040624	-1.5499286976
H	-1.1832149694	-1.0215173608	2.3224666759
H	-0.7680335237	0.6495601685	1.9155250684
H	0.0729570645	-0.1675550934	3.2554580401
H	0.4000482309	-2.8748993942	1.6611363666
H	1.9978816056	-2.4265824248	1.0198554988
H	1.5998116719	-2.0879308992	2.7122038225
H	3.565749759	0.4400399366	0.3510520042
H	4.3162217386	-0.1851251313	-1.1269493674
H	4.049143364	-1.2670002467	0.2615487151
H	1.4044558104	-2.0036467354	-2.3439402945
H	3.1111180515	-1.6379455668	-2.5941227982
H	2.6354721782	-2.8160925726	-1.3506316242
H	2.5744077085	2.5730997901	1.1681201114
H	0.8980472848	2.8833623493	1.6755169514
H	1.6969964435	1.3584701561	2.1211270278
H	2.1804713252	2.7620339	-1.2329633465
H	0.533737333	3.3112015699	-0.8346186622
H	0.7751414831	1.9674746993	-1.9628590355
C	-2.6326327807	-1.0886394972	-0.2600599226

C	-3.9000863036	-0.7853615715	0.2378885137
C	-4.3640761229	0.5309654527	0.2119102944
C	-3.5590022396	1.5391103274	-0.3218922745
C	-2.2916806364	1.2352548382	-0.8204572682
H	-2.2847407333	-2.1194634196	-0.2517112441
H	-4.5276346256	-1.5779588681	0.634636375
H	-5.3527570549	0.7680411928	0.5931319251
H	-3.9213429866	2.5621808805	-0.3608111767
H	-1.6847864588	2.0261935369	-1.252206642
H	-0.2342094872	0.0721655852	-2.2821826589

Y14

C	-2.6582250837	-1.753329148	-1.9599713725
C	-1.7644023458	-1.3838091699	-0.9193265314
C	-2.2707948383	-1.5020714801	0.4021203688
C	-3.5650622197	-1.9513225248	0.6522903113
C	-4.4240041255	-2.307236202	-0.3906636705
C	-3.9488143552	-2.2024360638	-1.7018076136
C	-0.4375719303	-0.9145083323	-1.2583884493
P	0.7006579472	-0.1795015563	-0.2391943561
N	2.0879999566	0.1222183107	-1.1957261724
C	2.6995230361	-1.0014692136	-1.9048318209
N	0.4504467404	1.2816070411	0.5441663712
C	-0.5635440395	2.0901295074	0.3754818629
N	-1.2283150056	2.3605427873	-0.8043479602
C	-0.5735080837	2.296186571	-2.1018507347
N	-1.0348283783	2.7901095223	1.46939709
C	-0.6669378475	2.3564993097	2.8082507763
C	-1.4839332529	4.1737389556	1.3759213156
C	-2.6834276609	2.4610761815	-0.8616920089
N	1.0095472591	-1.173321516	1.1137280644
C	1.0224704675	-2.6273211035	0.9705803268
C	1.793882338	-0.7103546757	2.2546022923
C	3.0727530083	1.1233338275	-0.7928551016
H	-0.8199690451	4.8224541702	1.964806032
H	-2.5052315427	4.2939402114	1.7601895347
H	-1.4566694704	4.5064566987	0.3385114263
H	0.2851721559	2.798215162	3.1372233328
H	-0.5713554847	1.2717712698	2.8298119055
H	-1.4529642319	2.664636955	3.5072321959
H	-3.1062583108	2.3366216468	0.1350611846
H	-3.0754717394	1.659494255	-1.5003710977
H	-3.0070939549	3.4278394961	-1.2723200034
H	-0.7628821229	3.2319629514	-2.6480413912
H	-0.9502138695	1.4570036449	-2.6958383846
H	0.5007858042	2.169142583	-1.9746822985
H	3.5612340211	1.5323121289	-1.687744715
H	2.5858252823	1.9365928463	-0.2542174128
H	3.8612186757	0.6991057631	-0.1481453386
H	0.4161359731	-2.9229426493	0.1144233412
H	2.045265824	-3.0183386088	0.8395332389
H	0.6013713955	-3.0861858899	1.8749960682
H	2.8505820093	-1.0164798822	2.1756961786
H	1.7416432518	0.3753034605	2.3252794994
H	1.393836292	-1.1455361502	3.1813372015
H	-0.2180647465	-0.7812450406	-2.313445896

H	-1.6304014522	-1.2426700369	1.2408662956
H	-3.9065550567	-2.0257418154	1.6834734266
H	-5.432040196	-2.6588033418	-0.188630995
H	-4.5922346462	-2.4749377907	-2.5365318501
H	-2.3129830404	-1.6855401524	-2.9904032679
H	3.1738698433	-0.63795079	-2.8259830256
H	3.4764758694	-1.5008085874	-1.3014823895
H	1.9386959264	-1.7360975356	-2.1718467836

Y14H⁺

C	-1.2784772697	0.8765024425	3.1372944688
C	-0.2149661432	0.073498738	2.7041952883
C	0.9742827641	0.0728227995	3.447297141
C	1.1011945757	0.8672404063	4.5870284885
C	0.0376331706	1.6699496945	5.0051775013
C	-1.1550409796	1.6683774564	4.2803632386
C	-0.3620838958	-0.794047397	1.4719088592
P	0.5041506173	-0.1020629986	-0.0135633118
N	0.3089526454	-1.2564146819	-1.2099440648
C	-0.6132810911	-2.3935234662	-1.1998191132
N	0.0758058487	1.4222171928	-0.2946979472
C	-1.0421435204	2.1315909888	-0.2967233324
N	-2.2379366354	1.6996205829	-0.8104743412
C	-2.3024524945	0.7261590363	-1.8940694008
N	-1.0093188743	3.3976179802	0.2010057524
C	0.0769069377	3.8102293079	1.0886298829
C	-1.7676922297	4.5035685042	-0.3925993846
C	-3.5342155416	2.1113020502	-0.269692666
N	2.155977655	-0.0225436597	0.1926841536
C	2.9398435916	-1.2494418468	0.3625866446
C	2.9019417571	1.2024712945	0.4967937698
C	1.0082406989	-1.0854102978	-2.4901322913
H	-1.0685309335	5.3059355008	-0.6524926478
H	-2.5123800499	4.9069664781	0.3025530418
H	-2.2642138258	4.1781869759	-1.3060990276
H	0.9353544866	4.188333327	0.5192596723
H	0.3926145894	2.973493449	1.7092591011
H	-0.2929861782	4.6103050253	1.7364644607
H	-3.3969707514	2.6246235837	0.6820387323
H	-4.1420939274	1.2152718337	-0.0968781798
H	-4.0834576068	2.7660644032	-0.9567123137
H	-3.0302277377	1.0702150374	-2.6377653339
H	-2.6241472766	-0.2610634835	-1.5388728184
H	-1.3291674262	0.6401228997	-2.3746618019
H	-1.3841110541	-2.2805417906	-1.9736387573
H	-0.0606507128	-3.3186623073	-1.4068367759
H	-1.1089302926	-2.5075325334	-0.236663192
H	0.2933400027	-0.9113826798	-3.3051944435
H	1.6982887743	-0.2415179011	-2.4424763983
H	1.582946364	-1.9895392693	-2.7255000433
H	3.82407633	-1.2141985354	-0.2847935503
H	3.2821250189	-1.3625693697	1.4002245015
H	2.3531794414	-2.1280679508	0.0883549924
H	2.2994226778	2.0715285718	0.244449338
H	3.1817232338	1.2453439827	1.5573026577
H	3.8170590696	1.2182790634	-0.1066800797

H	-1.4219249948	-0.9173396656	1.224051064
H	1.7987263659	-0.5688381928	3.147288452
H	2.0259000776	0.8480723931	5.1565156998
H	0.1333147344	2.280498257	5.8981014729
H	-1.9945474843	2.2735166576	4.6107237946
H	-2.2189548797	0.8649304882	2.5908019297
H	0.0487393789	-1.7952813751	1.6494781166

Y15

C	1.0915274148	3.4842378129	-0.7619102385
C	0.5190830917	2.1056954686	-1.1261882686
N	0.6076741344	1.3302198518	0.1403922187
C	1.3711810384	2.0917627082	1.1559677984
C	2.1069968177	3.163819809	0.3440004934
P	0.3816749045	-0.3369611887	0.0747870015
N	1.7012320208	-1.2073280892	-0.5246592987
C	1.7524030363	-1.777154388	-1.8896472314
C	3.2151018915	-2.2206368725	-2.0520952128
C	3.9861504891	-1.2250885055	-1.1760619927
C	3.059555857	-1.055442685	0.0358965612
C	-0.9481627668	-0.7340003388	-0.88637121
C	-2.2530796807	-0.1043611167	-0.9838897949
N	0.4959850579	-0.7261541244	1.749498316
C	-0.5031368056	-0.117003676	2.6642270507
C	-0.4252381051	-0.9958384777	3.9138483541
C	-0.2838349386	-2.3993505273	3.312115369
C	0.6066226902	-2.1799506812	2.0731479406
H	-0.256044765	0.9271432295	2.8703069329
H	-1.5176343818	-0.1503223823	2.2396934427
H	0.463830393	-0.7354570461	4.5012968666
H	-1.3038903579	-0.8848910968	4.5573259355
H	0.144420675	-3.1299018361	4.0057707137
H	-1.2683922533	-2.7701417479	3.0042249544
H	1.65418436	-2.4240215019	2.2860643839
H	0.2831871912	-2.8099000178	1.2365115233
H	1.0625568783	-2.6220188045	-1.9917185713
H	1.4693066964	-1.0324475009	-2.6443816649
H	3.3461794397	-3.239032175	-1.6661466558
H	3.5323532857	-2.2173966943	-3.0994630871
H	4.9851830707	-1.5729821401	-0.8948957203
H	4.0960083874	-0.2677077443	-1.7004613961
H	3.1878707732	-0.0830179738	0.519633419
H	3.262869879	-1.8282269464	0.7916989689
H	1.1241765549	1.631650269	-1.9104995897
H	-0.5140856339	2.1544336415	-1.4797663313
H	1.5303952919	3.9903569003	-1.6272807433
H	0.298209554	4.1291284466	-0.3642968832
H	3.0248889563	2.7477283215	-0.0912409563
H	2.3831154519	4.032750273	0.9501437669
H	0.6833444245	2.5758777402	1.8652022417
H	2.0292427836	1.4380366045	1.7320468515
C	-3.221901144	-0.6806034729	-1.8455604626
C	-4.4892860127	-0.1324503056	-2.0066285799
C	-4.8624535274	1.0258934794	-1.3180251507
C	-3.9289420187	1.6142896188	-0.4627494528
C	-2.6583249383	1.0671427832	-0.2942343949

H	-2.9572967783	-1.5812835965	-2.3964767929
H	-5.1948806547	-0.6160648392	-2.679494751
H	-5.8527553268	1.4549463439	-1.4431732057
H	-4.1923687645	2.5141008422	0.0903797587
H	-1.9653729674	1.5490235196	0.3895188229
H	-0.8411386695	-1.6803431707	-1.4072300489

Y15H+

C	1.5794053364	3.7079791258	-0.4433755955
C	1.0283461396	2.4350226956	-1.1045977319
N	0.7355261979	1.5254970141	0.0454388928
C	1.270839358	2.1067760685	1.3128867844
C	2.2443966179	3.1904160782	0.8390796534
P	0.4565742985	-0.084456229	-0.2223098332
N	1.7883973516	-0.8864798321	-0.7958466146
C	1.8225805214	-1.9034205577	-1.8890715474
C	3.2503462703	-2.4696859026	-1.8173515288
C	4.0758357221	-1.3168618176	-1.2297341489
C	3.1320794089	-0.7214754686	-0.1790965364
C	-0.8066547986	-0.2356857438	-1.556545523
C	-2.2231260577	0.0460403369	-1.0982837226
N	0.003850373	-0.7373566044	1.22689632
C	-1.0116949661	-0.1718664291	2.1662153463
C	-0.9330207185	-1.1211408423	3.3661049887
C	-0.6683848784	-2.4829126129	2.7098517803
C	0.3215478994	-2.1556184329	1.5818717934
H	-0.7677355474	0.8599773037	2.424441082
H	-2.0075372635	-0.1897982595	1.7135528284
H	-0.0985847928	-0.8404175023	4.0192427798
H	-1.8498927417	-1.1016226806	3.9610996958
H	-0.26308933	-3.2267093487	3.4008625858
H	-1.5973018871	-2.884014909	2.2892700617
H	1.3585879078	-2.2257624251	1.9296907154
H	0.2047778139	-2.826971832	0.7255010914
H	1.0698741286	-2.6844407798	-1.7392144593
H	1.6324679902	-1.4234647328	-2.8558462657
H	3.2777519554	-3.3344680858	-1.1450480435
H	3.6037416012	-2.798085262	-2.7981553122
H	5.0226412477	-1.6452631114	-0.7933314228
H	4.2977920282	-0.5701803751	-2.0005632399
H	3.3333212484	0.3342607567	0.0251352876
H	3.1969975064	-1.2736477894	0.7679957325
H	1.773881845	1.9816198877	-1.7709830164
H	0.1245882574	2.6325089198	-1.6881378911
H	2.2660415506	4.2428785492	-1.1044532849
H	0.7564905449	4.3870696115	-0.1944249053
H	3.2253505604	2.7539490038	0.6149961553
H	2.3887499612	3.9686477693	1.5933300192
H	0.4476536364	2.5551338443	1.8817166558
H	1.7377982259	1.3395736845	1.9347485552
C	-3.075157186	-1.0205958419	-0.779350964
C	-4.38006223	-0.7839047576	-0.3458631229
C	-4.8503030215	0.5255498398	-0.2266347754
C	-4.0123885246	1.5938750833	-0.5511654156
C	-2.7075254061	1.3558283514	-0.9844435288
H	-2.7210161517	-2.0440633308	-0.8828366475

H	-5.0310661921	-1.6212557949	-0.1118921573
H	-5.8673627753	0.7116486255	0.1052635165
H	-4.3764573607	2.6142919905	-0.474757935
H	-2.0705932281	2.1966901931	-1.2453222015
H	-0.7376730078	-1.2598336597	-1.936305713
H	-0.5016794393	0.4227572187	-2.3782142369

Y16

C	-2.6118609412	-1.9286645658	-1.9552363251
C	-1.7650225909	-1.4346978271	-0.9270188108
C	-2.3108284081	-1.4546900924	0.3847675935
C	-3.5914066388	-1.939846434	0.6393119356
C	-4.4010813814	-2.4228943655	-0.3916994945
C	-3.8904065027	-2.4083125156	-1.6939750816
C	-0.4490720462	-0.9397397714	-1.2638156767
P	0.6762193081	-0.1920363487	-0.2405977725
N	2.0582197441	0.1328436856	-1.1692842909
C	2.7372358395	-0.9548057096	-1.9054398039
N	0.4343052322	1.2658067104	0.5651254636
C	-0.5503002981	2.1037586784	0.3938476972
N	-1.2333153594	2.3732118108	-0.7791599358
C	-0.6077457513	2.2802760538	-2.0885363893
N	-0.9851246117	2.8422074069	1.4821874807
C	-0.5890038436	2.430968742	2.8194528396
C	-1.3578531652	4.2467062089	1.3636462328
C	-2.6855072421	2.5088069799	-0.808085469
N	0.9968813562	-1.1962177907	1.0828429032
C	0.8732232245	-2.6744484444	1.0117632929
C	1.8133700467	-0.7938796983	2.242123771
C	3.0059611596	1.2316636512	-0.8665803439
H	-0.6279189348	4.8775448601	1.8918033569
H	-2.3481743485	4.4365859335	1.797526371
H	-1.3703513199	4.5430055842	0.3150738853
H	0.4060034298	2.8121880095	3.0926322484
H	-0.5695873859	1.3430393403	2.8782032452
H	-1.3205780909	2.8181504595	3.5379095376
H	-3.0882644068	2.4230818229	0.2009827859
H	-3.1136766951	1.7027525531	-1.4178588016
H	-2.9935776024	3.4731208427	-1.2368633689
H	-0.7977842458	3.2100182525	-2.6454026711
H	-1.003707842	1.4357402155	-2.6623593339
H	0.467674857	2.1457965101	-1.9809418707
H	2.7691635635	2.1199948505	-1.472605418
H	2.9510884419	1.5357559598	0.1813492375
C	4.3802825874	0.6670020951	-1.2569308243
H	1.1949928768	-3.0651173246	0.0425030074
C	1.7486886974	-3.1851622721	2.1698624618
H	-0.1762358865	-2.9646555143	1.1423129265
H	2.8584168673	-0.5940418127	1.949893691
H	1.4195650622	0.1186498201	2.6930340957
C	1.74897517	-2.0157085309	3.1654067291
H	-0.2166292533	-0.8309275316	-2.3191989626
H	-1.7154446449	-1.0776033046	1.2131384052
H	-3.9633671721	-1.9349689685	1.6626252903
H	-5.3999314456	-2.7987660875	-0.1878189681
H	-4.4971774035	-2.7770623748	-2.519171863

H	-2.2383144538	-1.9333051024	-2.9778668381
H	1.367927578	-4.1190882766	2.5953043742
H	2.7716425634	-3.3723378317	1.8188103309
H	2.5793776669	-2.0492335946	3.8785409965
H	0.8117747419	-2.0029078881	3.7356223425
H	5.0867369361	1.4502048509	-1.5503303809
H	4.8162174141	0.1209636104	-0.410680214
C	4.042775695	-0.3104707791	-2.391286003
H	2.9524525445	-1.8109161869	-1.247776685
H	2.1160242663	-1.321719661	-2.7280170947
H	4.8272080169	-1.0499723404	-2.5809312207
H	3.8661050249	0.2399544475	-3.3237476162

Y16H$^+$

C	-1.414230796	0.7679366248	3.0927237028
C	-0.2698814644	0.0734864836	2.6786390962
C	0.9108891005	0.2174021476	3.4219442946
C	0.9476970339	1.0449768679	4.5447333006
C	-0.1975866683	1.736736285	4.945474145
C	-1.3804270838	1.5924016821	4.2189496932
C	-0.3168665851	-0.8288067841	1.4651420051
P	0.5282193244	-0.1214879707	-0.0216891584
N	0.2509052492	-1.2556026491	-1.2110108907
C	-0.5834358749	-2.48068023	-1.1280234506
N	0.1227433471	1.4148999443	-0.2869236455
C	-0.9836531688	2.1399420633	-0.2508318156
N	-2.200353266	1.7412073437	-0.7439639247
C	-2.3123560505	0.7833987732	-1.8365637748
N	-0.9175238241	3.3956121572	0.2703470794
C	0.195889351	3.7632979077	1.1435930414
C	-1.6606146571	4.5275822234	-0.2911378182
C	-3.4731537072	2.1663398922	-0.1607726277
N	2.1726312565	-0.0574413874	0.169438072
C	3.0099528257	-1.2739765879	0.2716635069
C	2.987150943	1.1770093779	0.3452944723
C	0.871695707	-1.1131372175	-2.5549446414
H	-0.9485201051	5.316283837	-0.558293001
H	-2.3789893977	4.9401923618	0.4261794451
H	-2.1868620376	4.2261559184	-1.1962694538
H	1.0712428891	4.0814489057	0.564214105
H	0.468328481	2.9191932389	1.7754405025
H	-0.1277123138	4.5910971188	1.7809045406
H	-3.3000971066	2.6589052041	0.7960131367
H	-4.0933857629	1.2787085558	0.012485287
H	-4.028577994	2.8437629904	-0.8205542077
H	-3.0538659679	1.1496308032	-2.555959776
H	-2.6408945353	-0.2030204011	-1.4849459906
H	-1.3541178797	0.6845788765	-2.3431977616
H	-1.6539864409	-2.2323614948	-1.1319484536
C	-0.1991945916	-3.2613078663	-2.3937387621
H	-0.3741958405	-3.0486320665	-0.216934118
C	0.1376352882	-2.1578799675	-3.4066012172
H	0.7541998748	-0.0933922123	-2.9376429057
H	1.9476478257	-1.3246499718	-2.5043744467
C	4.4267669425	-0.7465555619	0.0193833825
H	2.9356431227	-1.7223461614	1.2740955952

H	2.6951530742	-2.0277628155	-0.4558050375
H	2.9782299787	1.7607444652	-0.5807223659
H	2.5884395785	1.8053951664	1.1444372379
C	4.3927925681	0.6431107601	0.6723953926
H	-1.3536655062	-1.0501221277	1.1875236868
H	1.8014967618	-0.3344917538	3.1323956048
H	1.8674382414	1.1384154426	5.1151081482
H	-0.1712992516	2.3719422042	5.8259192819
H	-2.2812267393	2.1106056909	4.5355913471
H	-2.3459396494	0.6433232747	2.5452736223
H	0.1669688837	-1.790774641	1.6756695233
H	-1.0030980712	-3.9240131321	-2.7251963038
H	0.68503042	-3.8785179604	-2.1974075713
H	0.749243867	-2.5106533963	-4.2411415875
H	-0.7818966306	-1.7301505314	-3.8232586257
H	5.1944453527	-1.4032110919	0.4375853378
H	4.6050212306	-0.6592212993	-1.0586803129
H	5.1770038046	1.3105609642	0.3048846318
H	4.5183216446	0.5500067271	1.7576084277

Y17

P	-0.1134712838	0.0269294375	-0.1166709038
C	-0.1336620674	0.0423136905	1.7264684233
C	1.7314387229	-0.0533405015	-0.5328071874
C	-0.8427608864	-1.5492014209	-0.7015916698
C	-2.2105650956	-1.5940294523	-1.0161083323
C	-2.7987008765	-2.7866647256	-1.4356979917
C	-2.038657324	-3.9549141773	-1.5215097704
C	-0.6829261533	-3.9242591135	-1.1920429163
C	-0.0872938051	-2.7291099153	-0.7870158082
H	-2.7962117204	-0.6850099437	-0.9229934141
H	-3.8561334858	-2.8048542086	-1.6860590513
H	-2.501565772	-4.8861736096	-1.8371404785
H	-0.0853457658	-4.8300012031	-1.2519611048
H	0.9698416903	-2.7248165083	-0.5395560966
C	-1.1113509849	0.7984164263	2.3859569085
C	-1.181043815	0.8027182768	3.7790869847
C	-0.2879297559	0.0357424272	4.5294469207
C	0.6795390341	-0.7338209909	3.8808257294
C	0.7596361678	-0.7268800862	2.4879191422
H	-1.8191832277	1.3669277957	1.7919333007
H	-1.9376409193	1.4021507358	4.2786916539
H	-0.3477882071	0.0332152348	5.6146756688
H	1.3741135685	-1.3384169761	4.4581361063
H	1.5219723818	-1.3288615206	2.0017843654
C	-1.1107489141	1.2744366807	-0.7414019813
O	-0.6851064637	2.5956499861	-0.4163795805
H	-1.3814788428	1.1398630282	-1.7950914321
C	2.4297845376	1.2133365876	0.0157272222
H	2.1893577111	-0.9352424063	-0.0597551848
C	3.9155702773	1.2551438264	-0.3756355324
H	1.9161633001	2.0996500661	-0.3798153812
H	2.3318135428	1.2605169787	1.1060443535
C	4.1071515859	1.1454529856	-1.8945656437
H	4.3693485146	2.1811519427	0.0004339937
H	4.445229449	0.4258430405	0.117523942

C	3.4153016293	-0.1075938134	-2.4483334251
H	3.6808325783	2.0366863611	-2.3777837216
H	5.1755779503	1.1330781667	-2.1457412733
C	1.9280147702	-0.1561617767	-2.0600786106
H	3.5113387022	-0.1460426363	-3.5412029916
H	3.9221729152	-1.003789421	-2.0597523143
H	1.3988020453	0.6798695178	-2.538517205
H	1.4720703092	-1.0742244891	-2.4482916694
C	-1.7745927431	3.4814570487	-0.2695301639
H	-1.3635424663	4.454478862	0.0217887675
H	-2.3295508378	3.6009256089	-1.2144319864
H	-2.48657597	3.1379241841	0.49673534

Y17H^{+}

P	-0.0481126404	-0.2781270577	-0.6840604213
C	1.4859095469	-0.9667075801	-0.0109057437
C	-1.4656830953	-1.2776507414	-0.1665501058
C	-0.2487638397	1.4991813887	-0.2083769109
C	2.6215712253	-0.1485628062	0.1240446106
C	3.8206235341	-0.6952085147	0.5773583931
H	2.5817351116	0.9056927783	-0.1279586203
C	3.8990923887	-2.0539853748	0.8901138847
H	4.6933382229	-0.058547398	0.6848956254
C	2.7774682147	-2.8727480233	0.7454559406
H	4.8351705669	-2.4749150616	1.244688863
C	1.572115699	-2.3361279583	0.2955461658
H	2.8377018275	-3.9299957147	0.9846655867
H	0.7057851773	-2.9812968612	0.1912191636
C	-2.4766138107	-1.6310974279	-1.0772159642
C	-3.5838808292	-2.3593626625	-0.6428934132
H	-2.4135682626	-1.3500018179	-2.1232052611
C	-3.6951454132	-2.7343324989	0.6966610906
H	-4.3573943806	-2.6330486414	-1.3536602328
C	-2.6952025774	-2.3852055445	1.607655394
H	-4.5590772033	-3.3007546844	1.0309706996
C	-1.583243081	-1.661109522	1.1832141268
H	-2.7783371771	-2.6790035213	2.6494522905
H	-0.8057261714	-1.4089403344	1.8975235887
C	-1.4566513511	2.1413711671	-0.929739528
H	0.664308596	1.9778250583	-0.5866445656
C	-1.5760966886	3.6304158503	-0.5560547465
H	-1.3469278629	2.047114773	-2.0158765124
H	-2.3780132678	1.6170393411	-0.6425972239
C	-1.6460657448	3.8379744218	0.9626835437
H	-2.4631983209	4.0499394069	-1.0438610271
H	-0.7101694171	4.1717018594	-0.9621395609
C	-0.4491700367	3.1869920444	1.6686473292
H	-2.578589277	3.4030504227	1.3496463603
H	-1.682230189	4.9084586287	1.1932336204
C	-0.3309839583	1.6919075677	1.32252566
H	-0.5363613645	3.2964827986	2.7554723305
H	0.4772346852	3.7005566892	1.3751823382
H	0.5475243693	1.2585490436	1.814290917
H	-1.2127524226	1.1632538055	1.7080966367
C	0.0875893936	-0.3281405343	-2.5347808288
H	-0.8763597141	-0.0721856865	-3.0004954981

O	1.0874395908	0.6057497067	-2.8428113132
H	0.3539689067	-1.3546351023	-2.833038704
C	1.4590757285	0.595808179	-4.2236161641
H	2.2302048672	1.358388091	-4.340199474
H	1.8628467748	-0.3832439268	-4.5133649607
H	0.6008126703	0.8408579747	-4.863389379

Y18

P	0.0438854986	0.2052973736	-0.0420846239
C	0.1311543106	0.1605430022	1.778847351
C	1.8069480849	-0.0325903527	-0.6089726875
C	-0.7758114713	-1.2974601013	-0.6884671614
C	2.3949496068	-1.2784240107	-0.8577475248
C	3.7161293923	-1.3853507521	-1.3013096569
C	4.4700749164	-0.2234093211	-1.5098296173
C	3.8909162142	1.0334621228	-1.2741877128
C	2.5773466903	1.1207814071	-0.8358347925
H	1.8193377053	-2.18843328	-0.7113554037
H	4.1387094234	-2.3667505737	-1.4862997051
O	5.7621677149	-0.2055613819	-1.945038272
H	4.4887876575	1.9215545927	-1.4552906279
H	2.1249139077	2.0977707314	-0.6796974495
C	-1.0452487991	-1.4079672225	-2.0673033029
C	-1.6960431618	-2.5232501286	-2.5736938234
C	-2.0880459372	-3.5654666698	-1.7179768728
C	-1.8236901778	-3.4717004187	-0.3475820193
C	-1.1731843797	-2.3398986036	0.1537004378
H	-0.7585260483	-0.6047384744	-2.7382460958
H	-1.9133696749	-2.6145183777	-3.6335314884
O	-2.7141054631	-4.6180770603	-2.3182710468
H	-2.1174791405	-4.2605899771	0.335502638
H	-0.9830235557	-2.2780215072	1.2212726118
C	-0.982092551	0.5938284714	2.5265012957
C	-0.9551686779	0.5816528943	3.9129697137
C	0.1830562432	0.121040727	4.5938728939
C	1.2946677044	-0.3211354514	3.865472533
C	1.260289177	-0.2950915471	2.4699834501
H	-1.8566484206	0.9543010547	1.9945369981
H	-1.8055789977	0.9218036033	4.4958359744
O	0.1069833687	0.1421697278	5.9532295554
H	2.1875257388	-0.6757319761	4.367716239
H	2.1349255943	-0.6267013788	1.9198078094
C	-0.8674662016	1.5927940393	-0.4376552179
O	-1.1604534195	1.7820400871	-1.8123528452
H	-0.6232316379	2.5062090248	0.1128179992
C	1.2288172804	-0.3047167071	6.7001923191
H	0.9496567325	-0.1987207866	7.7499170627
H	1.4594236401	-1.3573764336	6.4903776092
H	2.117463964	0.3069642219	6.497315587
C	-3.1418576038	-5.6991955067	-1.5052784025
H	-3.6112964797	-6.4151660471	-2.1823563416
H	-2.2964031289	-6.1818381506	-0.9971131501
H	-3.8752865014	-5.3752182852	-0.7551491665
C	6.3962618571	-1.4429890228	-2.2277073388
H	7.4042092702	-1.1924332646	-2.5634193087
H	6.4596923254	-2.0782921937	-1.3342872943

H	5.8748446231	-1.99133775	-3.0232242077
C	-2.545179966	1.9967722779	-2.0314232011
H	-2.6857938133	2.109022741	-3.112977955
H	-3.15052394	1.1562024352	-1.6643125028
H	-2.892528493	2.914130179	-1.5312272615

Y18H^{+}

P	0.0558432369	-0.1451924643	0.0550053521
C	0.0498960306	-0.122138968	1.8564553508
C	1.7276856426	-0.1237861522	-0.6052822695
C	-0.8782875345	-1.5537691666	-0.5596044926
C	2.2897103155	-1.2884714646	-1.1476706997
C	3.5887707535	-1.289143341	-1.6492045526
C	4.348427919	-0.1088396615	-1.6168404146
C	3.7883676126	1.0660245743	-1.0750242297
C	2.4977690644	1.059309424	-0.5800036182
H	1.7104288686	-2.205431096	-1.1898070897
H	3.995302147	-2.2025416096	-2.0661166262
O	5.6072504464	0.008784322	-2.0756872207
H	4.3899994152	1.9687373162	-1.0666686926
H	2.0892224085	1.9800546558	-0.1735942514
C	-1.3966122042	-1.5479239977	-1.8746450795
C	-2.0826603378	-2.6474880038	-2.356375166
C	-2.2664804082	-3.7865650754	-1.5471737051
C	-1.7497437454	-3.8020554295	-0.2410905243
C	-1.0630104027	-2.6913295539	0.24242561
H	-1.2640571795	-0.6767447341	-2.5070136433
H	-2.492720246	-2.6571543496	-3.3608240438
O	-2.9466523568	-4.7981663557	-2.1158997154
H	-1.8809017455	-4.6651909162	0.4003183869
H	-0.6761741525	-2.7159923826	1.2564283876
C	-1.1781753679	-0.0881208667	2.5555904705
C	-1.1977266403	-0.0321122844	3.9366600208
C	0.0095270936	-0.009960937	4.6648954918
C	1.234178096	-0.0480616925	3.979473105
C	1.2465819264	-0.1025194258	2.5876213028
H	-2.1224295286	-0.1204040395	2.018557063
H	-2.1328433528	-0.0066420403	4.4861369784
O	-0.1224629557	0.0441454072	6.0019297143
H	2.174222661	-0.0370185831	4.5177157714
H	2.2015387055	-0.1309703363	2.0726193219
C	-0.7866418295	1.4255936485	-0.4679844412
O	-0.6779752467	1.5531476233	-1.8586793681
H	-0.305725191	2.2598299842	0.0693896344
C	1.0496455037	0.0698332948	6.8204666219
H	0.6871756234	0.1141246191	7.8472462471
H	1.6475039431	-0.8380175745	6.6806112904
H	1.6591988958	0.9553899082	6.6074848953
C	-3.1798695105	-5.9932583304	-1.3676548536
H	-3.7395239172	-6.6498701289	-2.0335495518
H	-2.2354311889	-6.4736611238	-1.0871499871
H	-3.7747658495	-5.7871791976	-0.4704318008
C	6.2485713568	-1.1284306882	-2.6578473309
H	7.2383661639	-0.7838258659	-2.9566266605
H	6.3472569495	-1.9413588457	-1.9292636772
H	5.7007533533	-1.4811249434	-3.5391108816

C	-1.3006096042	2.737986254	-2.3575368128
H	-1.1411325417	2.7398706264	-3.4367044934
H	-2.3782988584	2.7385523375	-2.1446471536
H	-0.8441920861	3.6355896064	-1.918368097
H	-1.8331001507	1.3736650244	-0.1248298718

Y19

P	-0.2466279349	-0.003464148	0.1999319839
C	-0.191625275	-0.4607686279	2.0127004278
C	1.5409497438	0.0371682054	-0.2939734399
C	-0.9206162181	-1.4881151358	-0.6474818329
C	2.4876798967	-0.8735029779	0.2008157973
C	3.7913074493	-0.8795365327	-0.2961325425
C	4.1649158322	0.0216699906	-1.2956121331
C	3.2253035896	0.9183965237	-1.8061879402
C	1.9186763132	0.9176064677	-1.3157008891
H	2.2116554819	-1.5815803474	0.9764583365
H	4.5149412545	-1.588717792	0.0973692326
H	5.1814534489	0.0173012139	-1.6803041599
H	3.5061568485	1.6142121236	-2.5925431905
H	1.1676172649	1.5867228102	-1.7262792755
C	-1.5367657283	-1.3484133172	-1.9012530777
C	-2.0058176557	-2.467926912	-2.5876272543
C	-1.8476355761	-3.7444036339	-2.0445253017
C	-1.2152583155	-3.8951543794	-0.8100335277
C	-0.7519959004	-2.7760242815	-0.1167040003
H	-1.6384876603	-0.3595162853	-2.3351778172
H	-2.4883337618	-2.3415075404	-3.553176518
H	-2.2078581115	-4.6165485557	-2.5836883125
H	-1.0814810907	-4.8850696151	-0.3818668759
H	-0.2684439659	-2.9095736235	0.8457047459
C	-1.3459164322	-1.0178316781	2.5924728716
C	-1.4373196438	-1.2056996531	3.9698725785
C	-0.385151104	-0.8153637041	4.8033722021
C	0.754093026	-0.2354643747	4.2467671961
C	0.8500847527	-0.0590384191	2.8639721133
H	-2.1810156966	-1.3052958137	1.9588014007
H	-2.33297827	-1.6515559584	4.3948068685
H	-0.4588216741	-0.9552538521	5.8785284711
H	1.5734567173	0.0815621476	4.8869304498
H	1.7451148164	0.3952433577	2.4501529901
C	-1.1491000199	1.3335262551	-0.3183171474
H	-2.1272722651	1.0077316987	-0.6729131994
C	-1.1458847591	2.7677503741	0.2183461123
C	-1.4467066405	3.7227335227	-0.9608741356
C	-2.2580696053	2.9487501178	1.2817027204
C	0.196122882	3.1830141799	0.8521592
H	-2.3182050844	3.9897682911	1.6301630978
H	-3.2374623086	2.677580968	0.8685549807
H	-2.0751883222	2.3080424015	2.1516406255
H	0.1455955868	4.2226084461	1.199435155
H	0.4407298851	2.5632242182	1.7232564823
H	1.0245116356	3.1109083488	0.1401457047
H	-1.5230850231	4.7651987109	-0.622007916
H	-0.6598888467	3.6650367307	-1.7215001453
H	-2.3949265353	3.458292055	-1.444993112

Y19H$^+$

P	-0.1608998992	-0.0868683504	0.1410839516
C	-0.3138467437	-0.376222048	1.9262297624
C	1.5931053616	-0.0012154355	-0.3332550967
C	-0.9088328727	-1.5060760678	-0.725131632
C	2.5106918077	-0.8879789893	0.2586620223
C	3.8362077469	-0.9113649092	-0.1701585811
C	4.257183317	-0.0625785818	-1.1961215864
C	3.3480094494	0.8077925677	-1.7985768085
C	2.0198844907	0.8382700009	-1.3747255005
H	2.199595991	-1.5561463077	1.0554354137
H	4.5388343794	-1.5942455238	0.2974850696
H	5.2908304542	-0.0830196391	-1.5279167321
H	3.6693542054	1.4654392463	-2.600311629
H	1.3309977202	1.5184928005	-1.862972182
C	-1.6285482674	-1.330338936	-1.9180998297
C	-2.1241741421	-2.4393626322	-2.6038807076
C	-1.9011207084	-3.7258358469	-2.1119142357
C	-1.1781317789	-3.9072382427	-0.9305603898
C	-0.6802505269	-2.8053511993	-0.2386311063
H	-1.809097791	-0.3403468682	-2.3231751463
H	-2.684661072	-2.2945261899	-3.522291851
H	-2.2892724106	-4.5866159711	-2.6480125016
H	-1.0032083923	-4.9069849337	-0.5449928538
H	-0.1283307998	-2.9606802101	0.6833365405
C	-1.492876864	-0.9621222865	2.4205465618
C	-1.6742571501	-1.1076525426	3.7945676845
C	-0.6873618672	-0.6725830836	4.6821633302
C	0.4848905673	-0.0897381134	4.1962000665
C	0.6745145348	0.0637476866	2.8232591963
H	-2.2639898229	-1.3090581971	1.7397239254
H	-2.5853879827	-1.5627465828	4.1705190303
H	-0.8312621266	-0.7893568531	5.7521104256
H	1.2541466313	0.2464504304	4.884585569
H	1.588899503	0.5190681188	2.458003517
C	-1.0924769723	1.4149991643	-0.408778069
H	-2.1350278604	1.0761315452	-0.4456575
C	-1.0426383901	2.7801622472	0.3535303906
C	-1.746152819	3.7927130821	-0.5785315093
C	-1.8298510763	2.7070145706	1.6765246592
C	0.3928138099	3.2649901457	0.6227751489
H	-1.8929665015	3.7057981151	2.1212958031
H	-2.8553085753	2.3556455617	1.5112912193
H	-1.3573935352	2.050143016	2.4107839407
H	0.3639446701	4.2532040695	1.0939108061
H	0.9358576891	2.5997753276	1.3028609967
H	0.9763649002	3.3594390939	-0.2992189662
H	-1.7905084562	4.7760238596	-0.0986897472
H	-1.209760752	3.9096486046	-1.5278588053
H	-2.7746676454	3.487060772	-0.8036267478
H	-0.7895444269	1.5705065151	-1.4513443164

Y20

P	0.0224379319	-0.3541177106	-0.1703185828
C	-0.1542549824	-0.2006615415	1.6637917142

C	1.7787059766	-0.3537597142	-0.7200824588
C	-0.7394717007	-1.9950938694	-0.5734739558
C	2.4224102962	-1.4103613573	-1.4021623622
C	3.684998093	-1.2474638858	-1.9947578205
C	4.3394261953	-0.0197305885	-1.8871763377
C	3.7621516111	1.0327554023	-1.1724971106
C	2.5107620891	0.8574484075	-0.5887684185
O	1.7719369681	-2.6004592176	-1.4645735319
H	4.1460592745	-2.0718661453	-2.5175583386
O	5.5611856549	0.2425530725	-2.4245963818
H	4.3096231427	1.9619539527	-1.0852094623
O	1.9367632504	1.8247319169	0.167024413
C	-1.4357464324	-2.1727378216	-1.7930097765
C	-2.2176431717	-3.3031345079	-2.0354255285
C	-2.2710548945	-4.3177683295	-1.0763881577
C	-1.5252096946	-4.2265370502	0.1013643476
C	-0.749281194	-3.0789672838	0.3240543167
O	-1.2649888755	-1.196968316	-2.7143418663
H	-2.7688150202	-3.4339025545	-2.9571993793
O	-3.0622916436	-5.381492625	-1.3974844684
H	-1.5188673213	-5.034655348	0.8181970674
O	0.0633259632	-2.9685883367	1.4111905882
C	-1.4540201885	-0.0856591802	2.2153549638
C	-1.6652261053	0.1409455634	3.5743531134
C	-0.5654949372	0.2388520014	4.4309810786
C	0.730479431	0.0794910304	3.9412815369
C	0.9220300496	-0.1609653681	2.5705083102
O	-2.4852595689	-0.2189054199	1.335953017
H	-2.6563750462	0.2450989183	3.9947937029
O	-0.8662440711	0.4742918807	5.7395873208
H	1.5809482704	0.1180062316	4.6058359367
O	2.1586616997	-0.4045147173	2.0670532074
C	0.199233948	0.6095928059	6.6670237957
H	-0.2688884844	0.815519855	7.6312403798
H	0.7898203085	-0.3131788469	6.7418926012
H	0.8622118696	1.4426333983	6.3999250079
C	6.194754789	-0.7620468659	-3.202752392
H	7.1290613005	-0.3208089891	-3.5538571456
H	6.418752457	-1.6550622997	-2.6043813108
H	5.580822527	-1.0474166064	-4.0662926806
C	-3.1619868208	-6.456012341	-0.4773422187
H	-3.8537258385	-7.1708622381	-0.9267657128
H	-2.1911972403	-6.9442563337	-0.3181944712
H	-3.5613982529	-6.1238201555	0.4898765379
C	-0.7989572019	1.037796121	-0.8018790839
C	-0.6270516661	1.7613889913	-2.0010568008
H	-1.524608096	1.4255979384	-0.0974478847
C	-1.3755321409	2.9767001573	-2.173613511
C	-1.2684019542	3.7658777787	-3.295454127
C	-0.3961143431	3.3919463398	-4.3346466037
C	0.3529672894	2.2096468436	-4.2158879277
C	0.24130683	1.4180380799	-3.0907008087
H	-2.0516891859	3.2795778527	-1.3770619355
H	-1.8442080254	4.6782883348	-3.3989059911
N	-0.276129059	4.2060444045	-5.5039003458
H	1.0161387718	1.9322933464	-5.0273870913

H	0.819700883	0.5040421944	-3.0424092891
O	0.5019938974	3.8426320385	-6.4053673772
O	-0.9544567433	5.2470022445	-5.5748677574
C	3.3071239545	-0.1336185745	2.8510532779
H	4.1573247482	-0.2716897444	2.1807731094
H	3.303176004	0.8971331358	3.2267160172
H	3.3967568798	-0.8299769548	3.6957444612
C	-3.8201173793	-0.0682583091	1.7950130019
H	-4.4482725752	-0.195106574	0.9121648984
H	-4.0769073653	-0.8324316934	2.5396226719
H	-3.9871325403	0.927664428	2.2236188628
C	2.3210316888	3.1810081756	-0.0364914759
H	1.5837957896	3.7781100913	0.5021232745
H	3.3198137526	3.3824999324	0.3723119653
H	2.2946424507	3.4388809585	-1.1002460467
C	2.2797320836	-3.6476739799	-2.2738666933
H	1.5361865171	-4.4444724527	-2.2161044603
H	2.3952916358	-3.332266445	-3.3184069924
H	3.2408583569	-4.0217957034	-1.8970525236
C	-2.0754129214	-1.1746330252	-3.8823634238
H	-1.8120129266	-0.2500818263	-4.3960901197
H	-1.8622722394	-2.0335959168	-4.5316896942
H	-3.1413412596	-1.1673813126	-3.62515744
C	-0.052165776	-3.8993303571	2.4732794537
H	0.5962490487	-3.5217105601	3.2658176294
H	-1.0824596423	-3.9611884943	2.845739951
H	0.2872988469	-4.8999533354	2.1738517426

Y20H^{+}

P	-0.4197759115	-0.0715406849	-0.3531666303
C	-2.1665086569	-0.3391404233	-0.8230078123
C	-0.0737477761	1.633460915	0.1901643655
C	-0.0526482016	-1.3564037523	0.8957860678
C	1.1489111644	1.9289156027	0.8530550917
C	1.4629998565	3.2177675732	1.2656215514
C	0.567059932	4.263338976	1.0054864328
C	-0.6257067212	4.0246915907	0.3175861723
C	-0.9324790311	2.7218147244	-0.0959374229
O	1.9811713629	0.8779166197	1.0427373242
H	2.390184254	3.4515844601	1.7708585035
O	0.9580849991	5.4772922552	1.4494677938
H	-1.3002279453	4.8363965323	0.0908217816
O	-2.0498301041	2.4479268881	-0.8125273552
C	-0.0099263578	-2.7067385133	0.4506606812
C	0.2469280028	-3.7627447166	1.3145824689
C	0.447120592	-3.5024018018	2.6758107444
C	0.3616775786	-2.197379563	3.1674364506
C	0.0916394276	-1.1395149962	2.2889470386
O	-0.2318465905	-2.8989898914	-0.8807220422
H	0.2964660686	-4.7884291255	0.9758238459
O	0.7007626314	-4.5881991498	3.4372571272
H	0.4756049315	-2.004371655	4.2234526077
O	-0.0740485994	0.1240258744	2.7439492645
C	-2.6580048746	-0.6434167393	-2.1198141256
C	-4.0077255219	-0.9138329856	-2.3306364446
C	-4.9113396475	-0.8717762579	-1.2623923459

C	-4.475450842	-0.5363135379	0.0228950919
C	-3.1196248002	-0.2627843976	0.2188213016
O	-1.7627954098	-0.6571377189	-3.1410506591
H	-4.3945511712	-1.1615621238	-3.3093201803
O	-6.1920798107	-1.1610952137	-1.5808795349
H	-5.1699919169	-0.4760859319	0.847301441
O	-2.6198816103	0.1168064779	1.4196952285
C	0.6481174687	-0.2819828927	-1.8792913618
C	2.147415081	-0.1954960737	-1.7310684965
H	0.2872026307	0.5089994391	-2.5408873742
C	-2.2143684371	-0.9341287334	-4.466497621
H	-1.3251794783	-0.8754296956	-5.0955421916
H	-2.6486053718	-1.937721014	-4.5377250499
H	-2.9477149427	-0.1909745357	-4.7986295847
C	-3.4720432396	0.1806013113	2.5575405782
H	-2.8291068035	0.4860011945	3.3825248819
H	-4.2653540171	0.9241970209	2.4176070373
H	-3.9159902453	-0.7972410626	2.777751188
C	-2.9682394131	3.4901539579	-1.1284923965
H	-3.7721955407	3.0066473057	-1.6845291898
H	-3.3764854209	3.9512777621	-0.2215856828
H	-2.4999710226	4.2581345238	-1.7551661848
C	3.2413632572	1.0858247092	1.6767673141
H	3.7184248886	0.1061228228	1.7032088574
H	3.861909827	1.7814390064	1.1020206928
H	3.1122458034	1.4633670879	2.6976888568
C	0.2412607323	0.4383511808	4.096108765
H	0.1392062112	1.5217201386	4.172176909
H	-0.4537849192	-0.0436244162	4.7946505463
H	1.2684367661	0.1484902354	4.3463531508
C	-0.3562949507	-4.2305128883	-1.3845053065
H	-0.6041326498	-4.1202802518	-2.4406768274
H	0.5844698902	-4.7832492728	-1.2858178058
H	-1.1590182964	-4.7694033403	-0.8702687676
C	2.8236144795	0.9858203832	-2.070335993
C	4.2128186786	1.0585117746	-2.0144781946
C	4.9242416917	-0.0705133446	-1.6157164056
C	4.2853340218	-1.2642075885	-1.2851338959
C	2.8966787263	-1.3188871941	-1.3485492617
H	2.2585767801	1.8571983263	-2.3891271082
H	4.7478090461	1.9621114593	-2.2802582588
N	6.3942691855	-0.0023978802	-1.5449105037
H	4.8775459753	-2.1242755517	-0.9971607383
H	2.3884675346	-2.2472820582	-1.1102645857
H	0.3598380376	-1.2315310141	-2.3211032073
C	-7.1822721608	-1.1593136282	-0.5550163172
H	-8.1147141872	-1.4384024513	-1.0463218112
H	-6.9468545177	-1.892717127	0.2254350106
H	-7.2905419453	-0.1638382475	-0.1075338846
C	0.9282605876	-4.4228167138	4.8352019259
H	1.1252446466	-5.4233520169	5.220825123
H	1.7975661849	-3.7813088858	5.0224397988
H	0.0451085918	-4.0069059614	5.334913146
C	0.1214739	6.6084511444	1.2161702459
H	0.6407540837	7.455113766	1.6657815041
H	-0.0108649519	6.790039519	0.1429154687

H	-0.8573123909	6.4840367762	1.6947681485
O	6.9928961186	-1.0167340085	-1.191049028
O	6.9276237754	1.066743693	-1.8358079377

Y21

C	-1.8119582912	-0.0151848439	-0.6416554558
C	-0.4532871725	-0.3771796931	-0.9264044533
P	0.9568507543	-0.1474819016	0.0007358777
N	1.0408789632	1.4074998638	0.662164311
C	0.560036783	2.5498324994	-0.1235877783
N	1.2401002728	-1.1158834041	1.3965305353
C	1.2937326991	-2.5632586788	1.1251445525
N	2.2742210245	-0.5878861351	-0.9700824857
C	2.3171289617	-0.2029599222	-2.3837885664
C	0.3738702849	-0.8591574397	2.5606936161
C	3.6168164187	-0.7428896639	-0.4048979839
C	2.1175846559	1.7844433388	1.5836968791
H	-0.3003811453	-1.0655964095	-1.7520066074
H	-0.6556583488	-1.2106297892	2.3980495354
H	0.3426063217	0.2037895549	2.7985945799
H	0.7876391732	-1.3945559094	3.4227316425
H	0.2990594444	-2.9771350188	0.9006683571
H	1.9526638138	-2.7749648651	0.2826928711
H	1.6885917251	-3.0684696529	2.0133233198
H	3.5626043092	-0.992333104	0.6543240905
H	4.2115050501	0.1762179098	-0.5223961584
H	4.1424766562	-1.5510351194	-0.9298977688
H	1.3135037106	-0.0231253587	-2.7666897863
H	2.9158215497	0.7092788942	-2.5319794313
H	2.7790729754	-1.0092386658	-2.9676178971
H	2.9564058336	2.2546867459	1.0480875371
H	1.7369405457	2.5093302979	2.3142544692
H	2.4915296909	0.9122870167	2.119882356
H	1.3737958732	3.0047175327	-0.7089635538
H	0.1616302934	3.3119258972	0.5566073524
H	-0.233788745	2.2401456375	-0.8017755379
C	-2.2412669087	0.775737786	0.4642091873
C	-3.5732143928	1.0885633291	0.6669233757
C	-4.5442778975	0.625363356	-0.2279051749
C	-4.169314496	-0.1566514038	-1.3291251894
C	-2.8382163295	-0.4644453001	-1.5254810159
H	-1.5081202273	1.1412306105	1.1753917634
H	-3.8845286448	1.6886020066	1.5139242918
N	-5.9339586254	0.9517707994	-0.0146702219
H	-4.9347256558	-0.5075241794	-2.0111243168
H	-2.5566338947	-1.0711722787	-2.3826513671
O	-6.767143024	0.5240596177	-0.8257270145
O	-6.229240985	1.6445580431	0.969664264

Y21H$^+$

C	-1.8334461035	-0.1096791227	-0.8314545196
C	-0.4407396115	-0.3958007019	-1.3525380287
P	0.9490651825	-0.1635346011	-0.1545172932
N	1.1622999536	1.4581642454	0.1075524242
C	1.2111541849	2.4331172541	-0.9951101564
N	0.6380576514	-0.868378391	1.3127913457

C	1.4111344058	-1.9964046856	1.8536105598
N	2.2858397361	-0.8335136868	-0.8772934063
C	2.3004054951	-2.1358788978	-1.5671472587
C	-0.4712111233	-0.4371422372	2.1844308809
C	3.6352222458	-0.2896734094	-0.6460241129
C	1.6340509399	2.0144021618	1.3876455376
H	-0.2126794882	0.2156202555	-2.2320026009
H	-1.2089575392	-1.2391986399	2.2888559767
H	-0.9695670544	0.442312339	1.7766319206
H	-0.0778790161	-0.1888480865	3.1773206575
H	0.7713661867	-2.8799113166	1.9616399099
H	2.2475513515	-2.2456682015	1.2016279063
H	1.808293899	-1.7314934327	2.8404441233
H	3.5815020311	0.7143257008	-0.2246511911
H	4.1648636747	-0.2339391514	-1.6028414833
H	4.2098674913	-0.9295054119	0.0352173321
H	1.309346004	-2.5890431187	-1.6012312547
H	2.6622425094	-2.0077806725	-2.5931170417
H	2.9685111931	-2.8326295775	-1.0467257858
H	2.6395884323	2.4373297269	1.2727487518
H	0.9560923662	2.8115227011	1.7111096474
H	1.6656508167	1.2433828143	2.1561518358
H	2.2319699873	2.8124464238	-1.1250621538
H	0.5531557404	3.2787441151	-0.7675622247
H	0.8936847534	1.9909065487	-1.9397867046
C	-2.6634538108	-1.1692402087	-0.4369588171
C	-3.9524078853	-0.9301356607	0.0333757087
C	-4.4014004974	0.3850957138	0.1042742216
C	-3.6093068834	1.4590246306	-0.2918768481
C	-2.3234672062	1.203256001	-0.7618336478
H	-2.3073069331	-2.193662396	-0.5082794545
H	-4.6088428921	-1.7371685919	0.3354966808
N	-5.7654505502	0.6501130033	0.6095944036
H	-4.0069702522	2.4650647431	-0.2357109988
H	-1.7072270438	2.0349313079	-1.0902726353
O	-6.126991606	1.8223302959	0.6597042966
O	-6.436496417	-0.3204735686	0.948001711
H	-0.3810963186	-1.4363362136	-1.6837472136

Y22

C	0.7342959484	3.624841663	-0.1259481489
C	0.2757345702	2.2890527163	-0.7298863002
N	0.44498194	1.3097378332	0.3811788511
C	1.1590413387	1.9449283865	1.5168555083
C	1.787079394	3.2035233978	0.9084293432
P	0.4031636348	-0.3290588724	0.0153273748
N	1.7871069761	-0.9392696278	-0.7246746196
C	1.901621993	-1.1954220849	-2.1811414205
C	3.3982055828	-1.4753264781	-2.3918566207
C	4.0751374396	-0.6425353027	-1.2957067033
C	3.1254603139	-0.819743141	-0.1040576358
C	-0.894916585	-0.6777930194	-1.0337243867
C	-2.2407676479	-0.1890508275	-1.0172319179
N	0.5238014176	-1.0208977355	1.5721907918
C	-0.4999004421	-0.6628752666	2.5902190467
C	-0.3605392977	-1.7675327406	3.6400028747

C	-0.1226827774	-3.0114196695	2.7751245095
C	0.7692603346	-2.4940535644	1.6321434707
H	-0.3082326236	0.3312019968	3.000455845
H	-1.5139593228	-0.6679155063	2.1656104214
H	0.5079286192	-1.5698353568	4.2800549591
H	-1.2449592056	-1.8448205552	4.2797179745
H	0.3427529261	-3.8387688461	3.3195451279
H	-1.0767674424	-3.3708118854	2.3725468214
H	1.8304999712	-2.6738305045	1.8413605325
H	0.5294401267	-2.9847663889	0.6827796962
H	1.2863044051	-2.0498457646	-2.4816260206
H	1.5680169518	-0.3287694987	-2.7650318991
H	3.6077540094	-2.5405321339	-2.2366914464
H	3.7256127689	-1.2146695254	-3.4027326494
H	5.0952922937	-0.9673802402	-1.0692141114
H	4.1136397057	0.4131359679	-1.5914159386
H	3.1707623486	0.0215040294	0.5927954319
H	3.3741641428	-1.7292897976	0.4608354783
H	0.9075706472	2.0139054376	-1.5844688039
H	-0.7623387011	2.3124158189	-1.0707082713
H	1.1183891113	4.3092625814	-0.8881956369
H	-0.1057233242	4.1208095211	0.3751467173
H	2.7328994491	2.9540473779	0.4103991229
H	1.992814974	3.9720390762	1.6601267034
H	0.4409027326	2.2303254458	2.298728012
H	1.8817320576	1.2611618281	1.9668480658
C	-3.1666389716	-0.7013866235	-1.9763106258
C	-4.4785447672	-0.2785255145	-2.0390916987
C	-4.9392718437	0.6909849764	-1.1366034469
C	-4.0693109841	1.223090273	-0.1773392252
C	-2.7563730675	0.7933686212	-0.1187809873
H	-2.8192670987	-1.4518862273	-2.6822802737
H	-5.164867571	-0.6810328422	-2.7747568402
N	-6.3081739061	1.1382029929	-1.19380477
H	-4.44538963	1.967889321	0.5142914825
H	-2.1039431882	1.2143502275	0.6392828111
H	-0.6873178094	-1.4810976888	-1.7331890344
O	-6.6803229765	1.9970069003	-0.3800718
O	-7.051566941	0.6443908401	-2.0542347405

Y22H+

C	1.3166183158	3.8281028199	0.2962282949
C	0.8731202336	2.6837687803	-0.6295231738
N	0.5887712367	1.5490321663	0.3037340713
C	1.0254575534	1.9004016606	1.6898903721
C	1.9512467753	3.1039625752	1.491069399
P	0.4487796962	0.0077730664	-0.2825489525
N	1.8535876052	-0.5671085714	-0.9420573017
C	2.0004418496	-1.371738069	-2.1930772259
C	3.4654990982	-1.8380299023	-2.1574604344
C	4.1798039337	-0.7489121997	-1.3449029732
C	3.1624834609	-0.4182777429	-0.2481271688
C	-0.7513208417	0.042825353	-1.6853521747
C	-2.2025747463	0.1401066565	-1.2638539912
N	-0.0093335046	-0.9515021862	0.9808274946
C	-1.0989334462	-0.6585812544	1.9603610318

C	-0.9517127342	-1.7881868376	2.9848817319
C	-0.53130245	-2.9835921027	2.1189775588
C	0.446406229	-2.3723480047	1.1038434093
H	-0.9726679954	0.3334569326	2.39712443
H	-2.0770576818	-0.6986354751	1.4710042775
H	-0.1672486521	-1.5433736415	3.7101993375
H	-1.8799421007	-1.9612583757	3.535534605
H	-0.0672706642	-3.7914878106	2.6905055297
H	-1.4029411655	-3.3966743024	1.5991467194
H	1.4763425576	-2.3981290668	1.476978001
H	0.4201009711	-2.8934841506	0.1420350197
H	1.3068780433	-2.2188242355	-2.2133102186
H	1.803183541	-0.7430668741	-3.0687873223
H	3.5388910616	-2.8022533728	-1.6424439547
H	3.8732403052	-1.9641657062	-3.1635879182
H	5.1361070573	-1.0795404907	-0.9316714483
H	4.3648089882	0.1354910096	-1.9649015662
H	3.2734856212	0.5960159093	0.1460745746
H	3.242808699	-1.1240696233	0.5894549163
H	1.6710791924	2.409557188	-1.3316982095
H	-0.0161926598	2.946329285	-1.2102294707
H	2.0002537469	4.5150328702	-0.2089396924
H	0.4442479821	4.4041664348	0.6235763064
H	2.9671047693	2.7715556422	1.2453386043
H	2.0106113882	3.7260663041	2.3880848099
H	0.1486677756	2.1859447894	2.2827745623
H	1.5079717168	1.0513738876	2.1792701697
C	-2.993235052	-1.0181759974	-1.2192776029
C	-4.3285929761	-0.9584376688	-0.8290512052
C	-4.8660908765	0.2782858375	-0.4832272149
C	-4.1144663823	1.448719895	-0.5293965895
C	-2.7803840244	1.3712920711	-0.9213616298
H	-2.5667090792	-1.9767427462	-1.5031709984
H	-4.9541802878	-1.8421311637	-0.7956605161
N	-6.2799519344	0.3512997569	-0.0595703109
H	-4.577779199	2.3917266208	-0.2659887794
H	-2.1929495626	2.2830968488	-0.9690840976
H	-0.5932797111	-0.8770408648	-2.2563497899
O	-6.7218707899	1.4584038595	0.2351267449
O	-6.9114922184	-0.7013074804	-0.0260989335
H	-0.4658606681	0.8721426969	-2.3431111067

Y23

C	1.1269474439	0.357167334	2.5434860718
C	0.005314946	-0.0378215385	1.8005431033
C	-1.1497081713	-0.4506704502	2.4829032683
C	-1.1847696528	-0.4550000403	3.8785653449
C	-0.061305616	-0.0642325007	4.6082550208
C	1.0956542617	0.338011003	3.9380247582
P	0.0187000237	0.0816740862	-0.0361348155
C	-0.5543060612	1.5903386062	-0.539103625
C	1.7474617158	-0.1639524049	-0.6367915427
C	2.2503404627	-1.4038148103	-1.0569468015
C	3.5804682822	-1.527531805	-1.4625377356
C	4.42350633	-0.4156413204	-1.4574357596
C	3.9310850149	0.8251401039	-1.0473091786

C	2.6034128754	0.9502967256	-0.6389603726
C	-0.8233439285	-1.5349651635	-0.5192898517
C	-1.6968068796	-1.4851599294	-1.6137450642
C	-2.3781721832	-2.6275698584	-2.0391485393
C	-2.20147484	-3.8361921727	-1.3642602806
C	-1.3417156116	-3.8986709473	-0.2643802535
C	-0.6578283473	-2.7563977072	0.1552208028
H	1.6039836586	-2.2741466481	-1.078870233
H	3.9549584743	-2.4943478889	-1.7886172733
H	5.4582282843	-0.514318887	-1.775308065
H	4.5797473675	1.6972209499	-1.047897093
H	2.2065248431	1.9133147854	-0.3313883131
H	-1.848565973	-0.5381048457	-2.1260952713
H	-3.0507841248	-2.5722443113	-2.8913352388
H	-2.7356108755	-4.7255876296	-1.6884856009
H	-1.2067906778	-4.8367809953	0.2685525134
H	0.0025640463	-2.8166860044	1.0167301484
H	-2.023654127	-0.7725981114	1.9247200073
H	-2.0894977859	-0.7669295494	4.3936188797
H	-0.0864061994	-0.0742018521	5.694753905
H	1.9754587191	0.6389635395	4.5005417468
H	2.0288722407	0.6783976737	2.0320373209
C	-0.3147612374	2.0897838102	-1.951789085
C	-1.6685802361	2.2992694724	0.202017821
H	-1.4751808047	3.383992183	0.2179262248
H	-2.6616482586	2.1744976925	-0.266306927
H	-1.7639556784	1.9794628438	1.2452687075
H	-0.0715427778	3.1650458734	-1.9400574085
H	0.5135299678	1.5811417888	-2.458105384
H	-1.1992289103	1.9898709004	-2.6068169316

Y23H^{+}

C	1.2241230072	-0.0592746071	2.6462739658
C	0.0351478896	-0.0335933991	1.8988559759
C	-1.2039726175	-0.04115431	2.5696632739
C	-1.2442645446	-0.0467776721	3.961790428
C	-0.0571147456	-0.0525143105	4.6988418281
C	1.1725377908	-0.0657083108	4.0406589106
P	0.0576383389	-0.0301009019	0.0810173638
C	-0.8098576061	1.4748698983	-0.6141893043
C	1.7698185286	-0.0685387161	-0.5366822129
C	2.169555681	-1.0966941581	-1.4068427828
C	3.4701254648	-1.1199096107	-1.9113951178
C	4.3810468458	-0.1268489344	-1.5520522512
C	3.9939280583	0.8941512618	-0.6802970003
C	2.697003324	0.9283849884	-0.1727396807
C	-0.8172601009	-1.5055191817	-0.5179420887
C	-1.3102297071	-1.5498516941	-1.835242714
C	-1.9433273958	-2.6992574718	-2.3039462027
C	-2.0871205157	-3.8103443791	-1.4689237573
C	-1.5929944939	-3.7743366741	-0.1642594309
C	-0.9585662571	-2.6279191061	0.3145013682
H	1.4741549617	-1.8785853183	-1.6903053008
H	3.7687132311	-1.9186799304	-2.583261386
H	5.3926706385	-0.1485275857	-1.9460024065
H	4.7021286454	1.665257611	-0.3929169192

H	2.4210638395	1.721357037	0.5137480623
H	-1.2005041959	-0.6992752614	-2.5013100366
H	-2.3250732141	-2.7257669042	-3.3199215645
H	-2.5833336087	-4.7033414213	-1.8369045562
H	-1.7013836476	-4.6375809555	0.4851042039
H	-0.5806999697	-2.6096184809	1.3312830498
H	-2.1347434902	-0.068075149	2.0139152369
H	-2.2034821173	-0.0527758763	4.4702945434
H	-0.0927745628	-0.0573782072	5.7840991259
H	2.0970583629	-0.0867368834	4.609384665
H	2.1875973089	-0.0865256663	2.1509300634
H	-0.6810660089	1.3449065178	-1.6972209886
C	-2.3162653824	1.5025324199	-0.2959076343
C	-0.1362145367	2.7955268486	-0.2033424389
H	-2.7832680666	2.2818381505	-0.9071506795
H	-2.8209122462	0.558526072	-0.5195765402
H	-2.495880159	1.7610988963	0.7516306558
H	-0.7186384375	3.6252309094	-0.6171517146
H	-0.1160257604	2.9188520007	0.884883735
H	0.8802024715	2.8857404657	-0.5918127463

Y24

P	-0.0271568618	0.0089090679	-0.2205794675
C	0.1164623702	0.1118941585	1.6644519075
C	1.752757139	-0.0316120755	-0.7003303308
C	-0.8747150258	-1.6389125178	-0.3728835999
C	2.3062283035	-1.0044454601	-1.555694029
C	3.6721201697	-1.0219260052	-1.8797156675
C	4.5040070796	-0.0296318535	-1.3586193761
C	3.9905648258	0.9749905064	-0.5329128411
C	2.6314162845	0.9726748734	-0.2224659647
O	1.4440048604	-1.9297618234	-2.0498245288
H	4.0657857582	-1.7879968116	-2.5310196077
O	5.8407956343	0.0519390508	-1.6085154975
H	4.6653370068	1.7396534502	-0.1720622543
O	2.0576514982	1.9376824763	0.5422812668
C	-1.522062723	-1.9334382087	-1.5832318242
C	-2.1869391998	-3.1451177549	-1.7586362533
C	-2.2017865587	-4.0929515505	-0.7309339716
C	-1.5556979838	-3.813742339	0.4723900945
C	-0.9022749562	-2.5915148273	0.6537808761
H	-1.4922907422	-1.1949387284	-2.3783479272
H	-2.6944792273	-3.3515565572	-2.6978892843
H	-2.7172958009	-5.0403721902	-0.8679797409
H	-1.5664295862	-4.541697141	1.2797846175
H	-0.4232084924	-2.3838613803	1.6039136232
C	-0.8736991497	0.8360566919	2.3397275516
C	-0.8478708574	0.9610608183	3.7306422566
C	0.1774603887	0.3660564177	4.4671336973
C	1.1753148971	-0.3541076506	3.8053547355
C	1.1435405276	-0.4798600248	2.4152072518
H	-1.6651508168	1.3161383151	1.7695111082
H	-1.6254998939	1.5268815648	4.2381326858
H	0.2022153271	0.4647849915	5.5494669916
H	1.9791506683	-0.8177930234	4.3728138215
H	1.9293592675	-1.0374703177	1.9107402456

C	-0.8721816669	1.1404116743	-1.1550816463
C	2.8763244304	2.9191243729	1.159759385
H	2.1990211118	3.5287199787	1.7593962437
H	3.6291877698	2.4589181061	1.8115722843
H	3.3749457213	3.5515858815	0.414549346
C	1.9206069829	-2.9739441895	-2.8808337433
H	1.043547965	-3.5791154118	-3.1158717308
H	2.3546630642	-2.5834226699	-3.8104498066
H	2.6635769828	-3.594871568	-2.3636935475
C	6.4344952798	-0.9211791657	-2.4541371138
H	7.4926764205	-0.6601002667	-2.5117121901
H	6.3326653397	-1.9322766067	-2.0387747369
H	5.9998334396	-0.8980078192	-3.4618853279
C	-0.2015715764	2.3711697901	-1.7239038527
C	-2.3851452877	1.2359580581	-1.0777439645
H	-0.6602557166	2.6190436001	-2.6948068353
H	-0.3081921377	3.2738175202	-1.0953321806
H	0.8681368035	2.2308842733	-1.9066262705
H	-2.8118323706	1.4096053868	-2.0797823568
H	-2.8600761897	0.3313555476	-0.6813530361
H	-2.7354554966	2.0792913661	-0.4543464842

Y24H^+

P	0.049219677	-0.1256589372	-0.0031232727
C	0.1624326355	-0.4197866241	1.792819311
C	1.7242098487	0.0569047603	-0.6709396556
C	-0.8216258731	-1.4976005149	-0.829502559
C	2.5350244096	-1.1050961587	-0.770793622
C	3.8408105733	-1.0587010908	-1.2609711552
C	4.3839360288	0.1813668761	-1.6187696637
C	3.6400351452	1.3604755586	-1.4661350502
C	2.3409030153	1.3037604185	-0.9793911548
O	1.9551055166	-2.2549393819	-0.3484309411
H	4.4272692436	-1.9618449396	-1.338474963
O	5.6280576675	0.3512932434	-2.101481658
H	4.1175212535	2.2962780163	-1.7214198927
O	1.6001790051	2.4193511801	-0.7603597849
C	-0.5177245575	-1.8115561976	-2.1638295479
C	-1.2571303494	-2.7807023065	-2.839411223
C	-2.3034197586	-3.4432513434	-2.1932648379
C	-2.6133938052	-3.1312537634	-0.8692783922
C	-1.8807612882	-2.1585692174	-0.1867486502
H	0.2978244547	-1.306378899	-2.6721646428
H	-1.0144122453	-3.018761071	-3.8707707488
H	-2.8763354793	-4.1994726507	-2.7216772734
H	-3.4268336485	-3.6421978334	-0.3629688603
H	-2.1341736977	-1.9262995254	0.8423262165
C	0.2766125763	0.6871093654	2.6519290482
C	0.4402274986	0.4953833314	4.0226426273
C	0.4961280079	-0.7975600938	4.5471763788
C	0.3952246159	-1.9001366603	3.6974135657
C	0.234063441	-1.7173410232	2.3240852818
H	0.2444658854	1.6991960675	2.2598094351
H	0.5226504261	1.3560430963	4.6794262581
H	0.6204846433	-0.9447195083	5.6158896308
H	0.4425280339	-2.9067013327	4.1022508019

H	0.1662044144	-2.5794825638	1.6710095947
C	-1.0356281389	1.3706500657	-0.2859490195
C	2.1630103157	3.7031610387	-1.0521492186
H	1.3879612408	4.425680866	-0.7951844419
H	3.0546215831	3.8866596839	-0.4436087262
H	2.4105872684	3.7921261506	-2.1150784115
C	2.6591534721	-3.4932632418	-0.465767945
H	1.9719836123	-4.2549941044	-0.0970703887
H	2.9118819767	-3.7039823949	-1.5104286345
H	3.5667848044	-3.4883864105	0.1473870346
C	6.4779032983	-0.7840264389	-2.2754915824
H	7.4110097207	-0.3903607876	-2.6782204353
H	6.6731482303	-1.2806697286	-1.3181290413
H	6.0439654349	-1.4966979285	-2.9862364854
C	-1.2281568789	1.6412771933	-1.7885002597
C	-2.3933781454	1.2098954077	0.4222879908
H	-0.4929073717	2.2087712962	0.1552348273
H	-1.8075361075	2.5636208042	-1.9043357549
H	-0.2803729574	1.7748665282	-2.3141842229
H	-1.7897060446	0.8353864811	-2.2706597071
H	-2.9660285879	2.1318765739	0.2745172919
H	-2.9780922205	0.3894124983	-0.0040284058
H	-2.2968748193	1.0478101705	1.4992599354

Y25

C	1.1215261759	0.4209756833	2.5376962455
C	0.0227956093	-0.0123781789	1.7880184086
C	-1.1076373907	-0.4830319021	2.4809238099
C	-1.1386387138	-0.5068112339	3.869095783
C	-0.027542584	-0.0707819549	4.6059660877
C	1.1106366668	0.3913487678	3.9334106709
P	0.0300661944	0.1317608102	-0.0420191931
C	-0.5489901983	1.6469715903	-0.5244984287
C	1.7508862045	-0.0983985345	-0.6540790143
C	2.2695959671	-1.3301345136	-1.0918573434
C	3.5891888722	-1.4442898718	-1.5076479205
C	4.4322545571	-0.3228127262	-1.5023270187
C	3.9325693164	0.9146987006	-1.0772781747
C	2.6047688745	1.0132266727	-0.6586730091
C	-0.8149287471	-1.468358085	-0.5430077629
C	-1.7213814426	-1.4022788327	-1.6049944691
C	-2.4146352912	-2.5298112958	-2.054597347
C	-2.2081292238	-3.7606402171	-1.4218942062
C	-1.3095269112	-3.8464685774	-0.3455154796
C	-0.6268127434	-2.7160521321	0.0837468968
H	1.6342416513	-2.2084530677	-1.1202215613
H	3.9909334609	-2.3929100983	-1.8503932523
O	5.7076897446	-0.539588903	-1.9292233749
H	4.5587977893	1.7996548684	-1.0718218607
H	2.2083361628	1.9741979514	-0.3432982052
H	-1.8987711257	-0.4441967769	-2.0877064505
H	-3.1083581728	-2.4355290883	-2.8824833282
O	-2.8291302837	-4.9251680653	-1.7651157824
H	-1.1709524539	-4.809335924	0.1373666275
H	0.0563749373	-2.8040806171	0.9248412476
H	-1.973304615	-0.8361799772	1.9290074004

H	-2.0123967626	-0.8607081985	4.4074124511
O	-0.1532007615	-0.1396247208	5.9611653244
H	1.9849996781	0.7278629072	4.4788040892
H	2.0095080175	0.7868484495	2.0313675643
C	-0.3360869557	2.1562812855	-1.9375482049
C	-1.6536456746	2.3458524604	0.2399253121
H	-1.4649559426	3.4316839774	0.2641172731
H	-2.654925662	2.2230341991	-0.212467432
H	-1.7310961867	2.0145962391	1.2812918552
H	-0.0977091405	3.233066315	-1.924821442
H	0.4866677271	1.6541408857	-2.4595433691
H	-1.2299543311	2.056953505	-2.5809329296
C	-3.753987816	-4.9021972656	-2.8415618863
H	-4.1225148185	-5.9248168004	-2.9406903263
H	-4.5977210602	-4.2309087773	-2.6343760873
H	-3.2731771201	-4.5970204094	-3.7802154848
C	6.603878307	0.5610209719	-1.9640132801
H	7.5496238093	0.1640060559	-2.3370952704
H	6.2485497037	1.348924322	-2.6407807644
H	6.7586917475	0.9870797644	-0.9640812708
C	0.9315450088	0.3032315308	6.7624680531
H	0.612696943	0.1681036189	7.7975334178
H	1.8366106302	-0.2914046753	6.5815552799
H	1.1554983728	1.3635698885	6.5871171324

Y25H^{+}

P	0.1382563212	-0.0671646051	-0.0115363688
C	0.1118654363	0.021938947	1.7960681723
C	1.8456212616	-0.0685934809	-0.5991510046
C	-0.7017308698	-1.5744672068	-0.5590464549
C	2.1859800559	-0.6897084095	-1.8131536838
C	3.4759187777	-0.6024962218	-2.3299036927
C	4.4578514568	0.1247746266	-1.6370088606
C	4.1229956855	0.7621492777	-0.4265773345
C	2.8390068601	0.6710612043	0.0807121642
H	1.4431240789	-1.2565214124	-2.3652515106
H	3.7064295002	-1.099400246	-3.2645132636
O	5.7326711875	0.2718430843	-2.0382700822
H	4.8933499398	1.3220089196	0.0931651624
H	2.6072508849	1.1703471288	1.0158608779
C	-2.0739686035	-1.5662983564	-0.8853125543
C	-2.734971651	-2.7416279354	-1.1968862942
C	-2.0477786775	-3.9699296966	-1.1819010508
C	-0.6845485143	-3.9943490841	-0.8437496202
C	-0.0287083609	-2.8067934275	-0.5316845847
H	-2.6383411084	-0.6394449491	-0.898044694
H	-3.7883810703	-2.7413463932	-1.4561272261
O	-2.7813399402	-5.051268544	-1.5021050709
H	-0.1328504233	-4.9264079376	-0.819561888
H	1.0243238308	-2.8501088277	-0.2700497134
C	-0.9542688047	0.651190216	2.4729806881
C	-1.0287409365	0.6263280052	3.8544333242
C	-0.0431248387	-0.0388851917	4.608258235
C	1.0154469968	-0.6820591177	3.9461890034
C	1.0815639604	-0.6517089486	2.5558197829
H	-1.7359477502	1.1671300186	1.924054069

H	-1.8413871522	1.1145893511	4.3817899875
O	-0.2066496074	-0.0004378256	5.9430144814
H	1.7854231178	-1.2038567074	4.5015884815
H	1.9086259981	-1.1566836278	2.0660117113
C	-0.7409920546	1.4183538622	-0.7132909524
C	-0.7698819007	1.3705464938	-2.2519589207
C	-0.1071901759	2.7284910977	-0.2106955369
C	0.7462211418	-0.6543628502	6.783842776
H	0.4005123574	-0.4845814506	7.803354602
H	0.7774885852	-1.7311870935	6.5815918962
H	1.7454048465	-0.2214892007	6.6584519136
C	-2.1650821939	-6.3406440056	-1.5012450843
H	-2.9514119712	-7.0399725086	-1.7847562137
H	-1.3501799329	-6.3891681257	-2.2327737541
H	-1.7880801625	-6.5970780828	-0.5045038305
C	6.1585761258	-0.3398858431	-3.2584061593
H	7.2120842337	-0.0809404019	-3.3618734134
H	6.0512824333	-1.4296536208	-3.211209092
H	5.59805247	0.0542370105	-4.113791278
H	-1.7703214605	1.3568798925	-0.3372594864
H	-1.3404259774	2.229150041	-2.6211217219
H	0.2404491129	1.4405200371	-2.6671910675
H	-1.2404995147	0.4634724627	-2.6403137296
H	-0.6855218399	3.5724066583	-0.6009635227
H	-0.0976538476	2.8037659832	0.8797678526
H	0.9200096839	2.8353450183	-0.5737424652

Y26

P	0.0050661015	0.2561586811	-0.1000999808
C	-0.0351388123	0.1106322274	1.7367458577
C	1.7424298809	0.0098916779	-0.6785655961
C	-0.8394137753	-1.3501603866	-0.6197039347
C	2.2224164607	-1.2357524685	-1.0984808605
C	3.5662060465	-1.3572704337	-1.4827022211
C	4.4148026635	-0.249381995	-1.4490681863
C	3.9248108847	0.9918820763	-1.0364781978
C	2.5861291503	1.1291129552	-0.6439841195
H	1.5559285532	-2.0860310086	-1.1339308073
O	4.137481253	-2.5207117975	-1.9102761877
H	5.4509327185	-0.3505001488	-1.7525215329
O	4.8293080596	2.0135882983	-1.0488095716
H	2.1696255853	2.0803039095	-0.3357881186
C	-1.650741786	-1.2677603555	-1.7551974841
C	-2.3190878704	-2.4134702569	-2.2142907982
C	-2.1866535206	-3.6236701653	-1.5311203007
C	-1.3848518904	-3.6975206246	-0.3887246398
C	-0.7030615335	-2.5620755686	0.0734766492
H	-1.7603026678	-0.3114186863	-2.2538157034
O	-3.128292932	-2.4448334556	-3.3128463437
H	-2.7144081371	-4.5043419665	-1.8800123747
O	-1.3329097482	-4.9246666323	0.2105292663
H	-0.0862062524	-2.6087270184	0.9627789201
C	-1.2299637439	-0.2508495576	2.3742368428
C	-1.2762794039	-0.2864826069	3.7758069613
C	-0.1383918349	0.0158570449	4.5260434578
C	1.0500299873	0.3645447066	3.8816751452

C	1.1089362019	0.4177302629	2.4816219299
H	-2.1005726154	-0.5059892753	1.784124574
O	-2.3862802634	-0.6139720548	4.4989324171
H	-0.1774989542	-0.0212719776	5.6089721608
O	2.105880998	0.6328868377	4.7040916424
H	2.0233534717	0.6927686743	1.9726808578
C	-0.5571553737	1.770711902	-0.6018450183
C	-0.2960429257	2.2770976891	-2.0082527381
C	-1.6839236061	2.4768332604	0.1226287185
C	3.3416939762	1.004922866	4.1133173731
H	4.02907387	1.1758003363	4.9438500128
H	3.739396284	0.2088457173	3.4705746577
H	3.2464748744	1.9272759059	3.5254605364
C	-3.5830796898	-0.917282177	3.7976735952
H	-4.3302866394	-1.1315336432	4.5639564805
H	-3.9205503042	-0.0686300498	3.1887590024
H	-3.4629479534	-1.797731336	3.1527537725
C	-0.5612795084	-5.0623651742	1.393306695
H	-0.6760874585	-6.102056394	1.7054501441
H	0.5016759285	-4.8547849462	1.2105700473
H	-0.9218976664	-4.400859656	2.1917794561
C	-3.3043136448	-1.2455126148	-4.0525489434
H	-3.9682975962	-1.5016164695	-4.8802325781
H	-3.7697817098	-0.459512295	-3.4439243069
H	-2.352027484	-0.8737754416	-4.4521749637
C	4.382188197	3.3110828294	-0.6842105505
H	5.2516535204	3.9636156452	-0.7825899964
H	3.5835980855	3.6646023783	-1.3486206519
H	4.023129889	3.3390486421	0.3530686423
C	3.3225994153	-3.6802494496	-1.9944793418
H	3.9750857466	-4.4765009142	-2.3573054332
H	2.9174034582	-3.962049757	-1.0136591087
H	2.4920831775	-3.5412620667	-2.6983803955
H	-0.0524800729	3.3525974137	-1.9887072348
H	0.5389779413	1.7698000233	-2.5043379206
H	-1.1714680715	2.1836828573	-2.6774872024
H	-1.4934790947	3.5620939395	0.1433252877
H	-2.6697911767	2.3515646379	-0.3619203805
H	-1.7946466625	2.15511443	1.1634836203

Y26H$^+$

P	0.0107544932	0.0216546144	0.1583568441
C	-0.0537744858	-0.1693439786	1.9618874592
C	1.7293819248	-0.0770768704	-0.4294650907
C	-0.9393249268	-1.2999032338	-0.6532548786
C	2.5950718374	-1.0094546481	0.1521159797
C	3.8874116595	-1.1622360244	-0.3706155365
C	4.3079777833	-0.3952219816	-1.4667136825
C	3.4236834026	0.5260466818	-2.0442723733
C	2.1287168072	0.6880808692	-1.5303271812
H	2.309820526	-1.6193403593	1.0009656646
O	4.659118478	-2.0796648304	0.2571524024
H	5.3059815568	-0.514876955	-1.8644030517
O	3.7251161602	1.311731895	-3.1044528258
H	1.4819617607	1.4074810213	-2.01756189
C	-1.5371526801	-1.0471901422	-1.8928362776

C	-2.175376272	-2.0948248089	-2.572105212
C	-2.2114553622	-3.3826587956	-2.0193312101
C	-1.5979461887	-3.620340164	-0.7811237689
C	-0.9556901027	-2.5807112334	-0.0922821383
H	-1.5282174321	-0.0721549338	-2.3651887926
O	-2.7301905478	-1.7584356805	-3.7599378626
H	-2.7074422464	-4.1866458323	-2.5447659069
O	-1.5726129503	-4.8239266973	-0.162907398
H	-0.4964944634	-2.8044103113	0.8629494091
C	-1.2276443698	-0.6476885766	2.55763641
C	-1.3180979833	-0.6851031896	3.9569264881
C	-0.2510208352	-0.24230453	4.7514885794
C	0.9128548725	0.2428839435	4.1386067706
C	1.0170808417	0.2861235596	2.7402511861
H	-2.0690268915	-1.0085497058	1.9789026045
O	-2.4828188202	-1.1697361132	4.4465899834
H	-0.3244115205	-0.2763617541	5.8294331222
O	1.9967135697	0.6938710704	4.8117752086
H	1.9354154656	0.6592989374	2.3041463518
C	-0.711324888	1.6794113759	-0.2809244188
C	-2.199439072	1.7617813454	0.1059200763
C	0.0980090994	2.8238290261	0.3565841234
C	1.9834604215	0.6825142776	6.2377675273
H	2.9500177117	1.0837593412	6.5432748661
H	1.1829855621	1.3193528629	6.6334452696
H	1.8724994353	-0.3370641312	6.6262828495
C	-2.6621058986	-1.2539097118	5.8587890839
H	-3.6589747213	-1.6709123446	6.0035032938
H	-1.9189571302	-1.9189998223	6.3150505604
H	-2.6103671925	-0.2632527739	6.3265563618
C	-2.1994237774	-5.940375701	-0.7902064423
H	-2.0460576059	-6.7814350132	-0.1136359864
H	-3.274847658	-5.7704477716	-0.9224070156
H	-1.7371909567	-6.1633737339	-1.7594708839
C	-3.3980760266	-2.7603448226	-4.5239702742
H	-3.7479162064	-2.2570453423	-5.4255954965
H	-2.7137510139	-3.571190003	-4.8013467188
H	-4.2571277089	-3.1698043217	-3.9788356213
C	5.0203702028	1.2163461834	-3.693346952
H	5.0224972763	1.9326384524	-4.5152509157
H	5.8045470071	1.4838506499	-2.9748266378
H	5.2051132797	0.2094346424	-4.0866676162
C	5.9899563425	-2.3014168298	-0.2042938621
H	6.4016711652	-3.0724263604	0.4473855192
H	5.9976931826	-2.6585656568	-1.2412518143
H	6.5969433406	-1.3917059673	-0.1209778651
H	-2.5925818076	2.7297084148	-0.2221467474
H	-2.7995380354	0.9779765263	-0.363461018
H	-2.3306835496	1.6985701145	1.1905632602
H	-0.3213733288	3.7782739572	0.0215921737
H	0.0309146662	2.794025664	1.4484713159
H	1.1538163737	2.8027461548	0.0742597669
H	-0.6266165496	1.7487400764	-1.3725081482

Y27

C	-1.5651179946	0.302727759	-0.839730494

C	-0.2476025888	-0.4276842439	-0.9880182175
P	1.1144374877	0.0237417868	-0.1327294385
N	2.2212848087	1.1802256739	-0.7702347709
C	2.2473623425	1.4305694865	-2.2024704097
N	0.7908388179	0.8040385648	1.3567812732
C	0.2464002271	0.0155867972	2.4558879805
N	2.0731453726	-1.3598256692	0.1223541922
C	1.4834125269	-2.6282278878	0.5382577806
C	0.4066796036	2.2116788097	1.4239497431
C	3.5286822583	-1.391614832	0.1035429947
C	3.2660850604	1.8698643101	-0.0253390491
C	-0.1911478703	-1.4204456077	-2.131270068
H	-0.6831417086	2.3386730931	1.5178390261
H	0.7343501578	2.7347500014	0.5247679893
H	0.8751875376	2.6889865192	2.2971560683
H	0.6695352192	-0.9901991008	2.4510250121
H	0.5177819437	0.488391241	3.4093844762
H	-0.8514096547	-0.0684957959	2.4173641003
H	3.9265023681	-0.5306791416	-0.4302317571
H	3.8674429892	-2.2968759685	-0.4197156891
H	3.9617025899	-1.4111210356	1.1167282493
H	0.3956913519	-2.5496076589	0.5244062937
H	1.7817879594	-3.4320139296	-0.1505497695
H	1.814703067	-2.9159289249	1.5493164163
H	4.2723619029	1.6183086722	-0.398140011
H	3.1495981585	2.9614694059	-0.1130954208
H	3.2129557186	1.6044769013	1.032162571
H	3.1566962957	1.028588997	-2.6787219326
H	2.2153950483	2.5118393608	-2.4047005013
H	1.3749967455	0.9685284772	-2.670567448
H	-2.3980256585	-0.4138699643	-0.7474414767
H	-1.6041236431	0.9455639865	0.0458408699
H	-1.8090469663	0.9405987705	-1.7078826061
H	-0.9590887306	-2.2041993792	-2.0162063302
H	-0.3843932173	-0.9517812937	-3.1127639357
H	0.7772034741	-1.9278241803	-2.2060827111

Y27H^+

P	0.9246697579	-0.0966719662	-0.1251858682
N	1.6042211318	1.4135852968	0.1081659186
C	0.7816746826	2.6354784092	0.1688682462
N	0.3485554941	-0.9025669048	1.2223371639
C	1.2817131833	-1.3376830557	2.2802272739
N	2.1631823775	-1.0430956883	-0.6986718333
C	2.1180628965	-2.5162143899	-0.6623899481
C	-0.472520192	0.0859055508	-1.3249171077
C	-1.1067175504	-1.2867169062	-1.6363014293
C	-1.002275703	-0.6481503842	1.755545455
C	3.2002651382	-0.5267674238	-1.6104323921
C	2.8400253762	1.5467194103	0.9053313359
C	0.0185798989	0.7493836406	-2.6300769785
H	-1.3781917766	-1.5700485729	2.2109271503
H	-1.6984174751	-0.3553791455	0.9705031443
H	-0.987518428	0.1354039176	2.5243426349
H	0.9249242128	-2.2846774273	2.6985755412
H	2.2828745579	-1.4945870181	1.8787091187

H	1.3350460136	-0.5983115253	3.0895548578
H	3.1568160302	0.5594233629	-1.6754812462
H	3.066853271	-0.94640897	-2.6146892679
H	4.1891323882	-0.8222547386	-1.2418629554
H	1.3005890153	-2.8661128684	-0.0337293606
H	1.9838379383	-2.918385825	-1.6737429686
H	3.0633596047	-2.8995984239	-0.2618434959
H	3.4484200176	2.3535084826	0.4835049374
H	2.6121568842	1.7931013713	1.9503359424
H	3.4234652853	0.6262228565	0.8787375602
H	1.3746770594	3.4744216998	-0.209309688
H	0.4791244048	2.8638797571	1.1991448502
H	-0.111499236	2.5531728764	-0.4490682834
H	-1.9500758275	-1.1347473226	-2.3173145606
H	-0.393416397	-1.9450169825	-2.1415481715
H	-1.4803099952	-1.8044995706	-0.7500653326
H	-0.8359119376	0.8686587166	-3.3035503226
H	0.4644585308	1.7347688716	-2.4766971107
H	0.7523085455	0.118526232	-3.1413860394
H	-1.2325771783	0.7298966578	-0.8675807702

Y28

P	0.0229510828	-0.0314971015	-0.0520496648
C	-0.2677673889	0.0287466665	1.782313602
C	1.8400506975	-0.2581355762	-0.4669661678
C	-0.8519060253	-1.6511386844	-0.4309143708
C	2.3970800425	-1.2319478325	-1.3247096076
C	3.7363143827	-1.1630380547	-1.7402009911
C	4.5367783895	-0.1110652482	-1.3151563009
C	4.0227225461	0.8833823488	-0.4888931407
C	2.6870232392	0.8094492838	-0.0802016759
O	1.5717627894	-2.2293038995	-1.7537138914
H	4.1478854659	-1.9194567634	-2.3965880983
H	5.5726515934	-0.0592655908	-1.6397655682
H	4.6526830032	1.7051046348	-0.1706600311
O	2.1317653444	1.7546578287	0.7294318151
C	-1.9990170982	-1.7461512012	-1.248848011
C	-2.6307272381	-2.9850497894	-1.4608919754
C	-2.1443798015	-4.1253814735	-0.8352338252
C	-1.0366197062	-4.0644617831	0.0074245521
C	-0.4060051305	-2.8312954646	0.2025228617
O	-2.4674400713	-0.6032304392	-1.801916498
H	-3.5043703386	-3.0489905148	-2.0978801062
H	-2.6416529269	-5.0784231031	-0.9973510675
H	-0.6754405951	-4.9603344144	0.497673348
O	0.6831554364	-2.6797544824	1.0044624784
C	-1.6110726604	0.1967690932	2.2029345755
C	-1.9542990588	0.299096648	3.557037093
C	-0.9545190946	0.196962581	4.5176329862
C	0.3657207032	-0.030785999	4.1502381832
C	0.7053984129	-0.141481283	2.7924372516
O	-2.5344711315	0.2559802963	1.2108118309
H	-2.9837608941	0.4510382675	3.8560567172
H	-1.2114611304	0.2792766222	5.5704645239
H	1.1248629596	-0.1461454891	4.9138996461
O	1.969858389	-0.4504252879	2.4062240954

C	-0.5018870207	1.4265388278	-0.8038251868
C	-0.4243120865	1.5940561519	-2.2379939135
C	-0.9657169464	2.5940884499	0.0382158619
C	-3.8975136241	0.4852201544	1.5243761482
H	-4.4127884726	0.5046217767	0.5633895679
H	-4.3103531975	-0.3199952811	2.1461382604
H	-4.0354931275	1.4475098571	2.0334846892
C	3.0363410071	-0.2922218401	3.3257640132
H	3.9488412068	-0.4183644354	2.7396090368
H	3.0292428015	0.7054142785	3.7824158555
H	3.0093966556	-1.0517594679	4.1187319954
C	2.7656674306	3.0182875871	0.8511717799
H	2.054066204	3.6547569238	1.3797061805
H	3.6940001677	2.953924365	1.4352104602
H	2.9839534814	3.4509441732	-0.1322206972
C	2.0291630622	-3.1457034443	-2.7331656566
H	1.176099695	-3.7929096801	-2.9446219262
H	2.3389911063	-2.6328531907	-3.6523861091
H	2.8626332652	-3.7566345976	-2.3613341666
C	1.2624502796	-3.8139044614	1.6198965332
H	2.1259330905	-3.4370601525	2.1699431832
H	0.5665860556	-4.2960742087	2.3192400385
H	1.5942270799	-4.5496592436	0.8756475863
C	-3.3788344325	-0.6677587028	-2.8865674276
H	-3.4507122519	0.3531895	-3.2632491697
H	-3.0060284638	-1.321897689	-3.6842113644
H	-4.3710795753	-1.0137077458	-2.5647990409
C	-0.775830889	2.8361984967	-2.8427340993
C	-0.705952674	3.0411659166	-4.2185153511
C	-0.2882518186	2.0279207971	-5.0834930195
C	0.0478071794	0.79077812	-4.5226736756
C	-0.0224093154	0.5755120705	-3.1516947825
H	-1.1156802707	3.6556397752	-2.2189142301
H	-0.9865393919	4.0144856375	-4.6186819718
H	-0.2325171954	2.1914233168	-6.1564196572
H	0.3659387612	-0.0275449587	-5.1675080938
H	0.2228200544	-0.415675447	-2.7836573312
H	-0.372056484	3.4972806036	-0.1759718186
H	-2.0202848841	2.8622180821	-0.1442903755
H	-0.8604066494	2.4104078903	1.108904306

Y28H⁺

P	-0.1533446234	-0.1120129973	-0.0660122358
C	-0.3500028831	0.0650903716	1.7584406901
C	1.5903475321	-0.0559232101	-0.6322485816
C	-0.9341133705	-1.7582930378	-0.3808903978
C	2.1285393002	-0.9619378534	-1.5762436962
C	3.4678233033	-0.8587991639	-1.9774160639
C	4.2614891903	0.16327218	-1.4720760474
C	3.7451005298	1.1109452381	-0.5925990832
C	2.4085383978	1.0122727128	-0.2000088481
O	1.2909256708	-1.9028995582	-2.0716738336
H	3.8834539963	-1.5619609583	-2.6872664691
H	5.2978189349	0.2386024212	-1.7883115856
H	4.3678896446	1.9250297165	-0.2447335191
O	1.8001756956	1.9368955329	0.5906427194

C	-2.14423827	-2.0144263585	-1.0691953819
C	-2.6579396599	-3.3165937156	-1.1500887155
C	-1.9836793831	-4.3706355553	-0.5464757787
C	-0.7965756777	-4.15900826	0.1455263881
C	-0.2830146901	-2.862608615	0.2251919158
O	-2.7923291535	-0.9631552105	-1.6442898723
H	-3.5827938414	-3.5076442773	-1.6776504993
H	-2.3933739605	-5.3741503488	-0.6140046209
H	-0.2837673662	-4.9886519431	0.6147550795
O	0.8669517087	-2.5629767618	0.8751676004
C	-1.6862006647	0.1872800165	2.2130104312
C	-1.997660983	0.2935691136	3.5704152679
C	-0.9633034762	0.2516380842	4.4990835225
C	0.3573964769	0.0866961773	4.0987791959
C	0.6724267098	-0.0270248631	2.7366866007
O	-2.6318458438	0.1908470163	1.2333303193
H	-3.0227868839	0.401451037	3.9003065725
H	-1.1920620917	0.3362128246	5.5573517324
H	1.1376444006	0.0318701683	4.8465693713
O	1.937815985	-0.2487842362	2.3106865821
C	-1.0224268515	1.3153830255	-1.0070509132
C	-0.353485205	1.5422278717	-2.3607081352
C	-1.1797237685	2.5930811438	-0.1640444987
C	-4.0126068277	0.2491218129	1.5890993085
H	-4.5567247872	0.2063354156	0.6455396777
H	-4.2931309912	-0.6038579137	2.2166713917
H	-4.2497936927	1.1854962912	2.1063633646
C	2.9998034025	-0.2944291755	3.2594390109
H	3.9067028065	-0.4367842013	2.6700907653
H	3.074193424	0.6413032195	3.8259986322
H	2.8796261962	-1.1319443476	3.9569456777
C	2.58175085	2.9702756602	1.1860469386
H	1.8941796392	3.5312401338	1.8201624671
H	3.3883657946	2.5518879845	1.7982782216
H	3.0057115316	3.6368441753	0.4264421567
C	1.7517919359	-2.7943389027	-3.0836569322
H	0.897339588	-3.4299546724	-3.3187977473
H	2.0631177256	-2.2465190326	-3.98019641
H	2.5794925669	-3.4165892174	-2.7238983035
C	1.6123122552	-3.5997452523	1.505875831
H	2.4857078101	-3.1081297483	1.9334650526
H	1.0304758428	-4.0794863207	2.30133114
H	1.9350219075	-4.3535457185	0.7787058192
C	-3.9942905054	-1.1903326989	-2.3822007367
H	-4.2927860071	-0.2109644039	-2.7581878182
H	-3.8224673478	-1.8676167077	-3.2259207117
H	-4.7847534572	-1.5944428859	-1.7398263314
C	0.5282610092	2.611287261	-2.5780405518
C	1.0988148692	2.8275557243	-3.8327902433
C	0.802901078	1.9764177532	-4.8976589405
C	-0.0721338123	0.9082845619	-4.6963329951
C	-0.6443673942	0.6951737842	-3.442126201
H	0.7691638092	3.2913876136	-1.7683273784
H	1.7722471963	3.6677574152	-3.9777912031
H	1.2427344438	2.1490631058	-5.8757892295
H	-0.321866384	0.2454910879	-5.5206796134

H	-1.3316260148	-0.1316240063	-3.3010180419
H	-0.2286041996	3.0013538598	0.1735237586
H	-1.6779964741	3.3447927086	-0.7856587937
H	-1.8057203189	2.4117341324	0.7095779506
H	-2.0146772968	0.9079847769	-1.1790231942

Y29

P	0.0327754199	0.0104505425	-0.0722509685
C	-0.2197621488	0.0504387683	1.7634696558
C	1.8268993139	-0.2734032371	-0.5217624771
C	-0.9044534786	-1.5574460459	-0.4765660241
C	2.3517883075	-1.2583844659	-1.3924596895
C	3.682481499	-1.2357977441	-1.8180655846
C	4.5306274413	-0.2128116896	-1.3954494017
C	4.0568589095	0.7940693433	-0.5545626413
C	2.7190410809	0.7537247991	-0.140511776
O	1.493804796	-2.2216237541	-1.8306736525
H	4.0830386656	-1.9845658965	-2.4878944169
O	5.8123924949	-0.283695559	-1.8609685711
H	4.7032852198	1.5932552762	-0.222550249
O	2.2145090732	1.7116569636	0.6863346159
C	-2.0637978191	-1.5970077076	-1.275568526
C	-2.7425743229	-2.8036418439	-1.5340930116
C	-2.2814960986	-3.9849279982	-0.9588404901
C	-1.1589365112	-3.9812924544	-0.1237927737
C	-0.4952054559	-2.7792853319	0.108070015
O	-2.5208373154	-0.4237897901	-1.7736379439
H	-3.6254372481	-2.7962439697	-2.1563895947
O	-2.8668171769	-5.2059143117	-1.1394711768
H	-0.8399768833	-4.9165476796	0.3171737802
O	0.6013586756	-2.69675829	0.908234424
C	-1.5412606284	0.2585702035	2.2210165857
C	-1.8675343162	0.3515263869	3.5817429969
C	-0.8530145429	0.1955772661	4.5253704675
C	0.4544103125	-0.0772050788	4.12073142
C	0.760752593	-0.1733225833	2.760963336
O	-2.4869728315	0.3680253289	1.2539119513
H	-2.888022806	0.5334349294	3.8843999449
O	-1.0431731316	0.2718786953	5.8741112746
H	1.2020367424	-0.2337329012	4.8866138226
O	2.0077855995	-0.5159478917	2.3498676364
C	-0.4564881639	1.5014953575	-0.7898001017
C	-0.4072942365	1.6881513005	-2.2210289712
C	-0.8754739459	2.6659720479	0.078800968
C	-3.8297491695	0.6602086672	1.5947108048
H	-4.3596545565	0.7199846858	0.6433061068
H	-4.2736217991	-0.1327958764	2.2114614619
H	-3.9115338112	1.6207852828	2.1194148156
C	3.080739293	-0.4763924626	3.2735548194
H	3.9818703535	-0.6459556685	2.6809125141
H	3.1469493101	0.4990950273	3.7717896554
H	2.9947137987	-1.2634526099	4.0347760968
C	2.8862630828	2.9545483932	0.8060472029
H	2.2023898774	3.6070874768	1.351304759
H	3.8216746817	2.8603230942	1.3752611346
H	3.1002081953	3.3889731994	-0.1776209357

C	1.9267732568	-3.1574467883	-2.8029540552
H	1.0562790651	-3.7823652544	-3.0102413295
H	2.2520586307	-2.6593595787	-3.7248671416
H	2.7431057265	-3.7874078132	-2.4254280809
C	1.1422261864	-3.8745346071	1.4752666726
H	2.0203307262	-3.5511552607	2.0362062078
H	0.4308314052	-4.359161159	2.1568654213
H	1.4446136737	-4.5908547827	0.7003980319
C	-3.4085722557	-0.4192933672	-2.8790769668
H	-3.4499375274	0.6188238008	-3.2109667285
H	-3.0313052648	-1.0441039059	-3.6976135947
H	-4.4160744198	-0.7555555442	-2.5948906051
C	-0.750717444	2.9451280513	-2.8020334553
C	-0.7067853811	3.1690123979	-4.1758825056
C	-0.3251918249	2.1620497897	-5.0648984029
C	0.0012747921	0.9112478918	-4.5284311203
C	-0.0434008038	0.6763661307	-3.159728377
H	-1.0641527961	3.7609149255	-2.1597079899
H	-0.979639128	4.1528383903	-4.5556493585
H	-0.2896544853	2.3403415567	-6.1363768862
H	0.2922729279	0.0971689802	-5.1913854475
H	0.1932189277	-0.3242671217	-2.8123175277
H	-0.2742033893	3.5623354553	-0.1439375562
H	-1.9308787564	2.9557923615	-0.0656084201
H	-0.7381647378	2.4674708284	1.1434968121
C	6.711717074	0.7585509436	-1.5216276084
H	7.6540539003	0.5131229883	-2.0153168965
H	6.3561993577	1.7324804644	-1.882586075
H	6.8770411054	0.8145679083	-0.4370912389
C	-2.3500927929	0.5354976674	6.3587075603
H	-2.2658354785	0.5599790946	7.4468134722
H	-2.7251375655	1.5039920532	6.0026640329
H	-3.055769722	-0.253978475	6.0682550959
C	-4.0113604653	-5.2881690467	-1.972381805
H	-4.3011139418	-6.3408058319	-1.9798389178
H	-4.8420392232	-4.6871251733	-1.5791608728
H	-3.7892126912	-4.9660221639	-2.9982946311

Y29H$^+$

P	0.2161509547	0.0677010866	-0.2204837898
C	2.0393573001	0.3104469014	-0.2723824743
C	-0.2876716395	-1.6656437408	-0.5111256653
C	-0.238018155	0.6948774594	1.4419274504
C	-1.6069856787	-2.0714490106	-0.1733340012
C	-2.0560693161	-3.3657523192	-0.4116094419
C	-1.2025593036	-4.2904334096	-1.0259223676
C	0.0853025264	-3.9178467092	-1.4199325015
C	0.5269649672	-2.6104892613	-1.1755013361
O	-2.3901081601	-1.1179887716	0.3809848824
H	-3.0564623658	-3.6840573136	-0.1515091032
O	-1.7272322346	-5.523221237	-1.2106680706
H	0.7311648529	-4.6216637707	-1.9226173385
O	1.7457168277	-2.1824042372	-1.5909515427
C	-0.0890335969	2.0912547954	1.6578348534
C	-0.3565803057	2.6901466502	2.8810178331
C	-0.7600162977	1.8912395001	3.9571591744

C	-0.8636723009	0.5064486459	3.8101289567
C	-0.5830041322	-0.0881010113	2.571439704
O	0.3353418741	2.8039469706	0.5771148624
H	-0.2616793999	3.7559835142	3.0373517467
O	-1.008442867	2.5564068734	5.1073976126
H	-1.1307585128	-0.1116795038	4.6537972566
O	-0.5976428685	-1.4337446198	2.4257437066
C	2.7538282005	1.2752925223	-1.0309722747
C	4.137420741	1.4005416693	-0.9175122306
C	4.851962295	0.5837665432	-0.0359135313
C	4.1871105313	-0.3662572627	0.7440084766
C	2.8008737614	-0.4835939444	0.6179325307
O	2.0437562433	2.078975982	-1.8674635538
H	4.6928664575	2.1307348507	-1.4893513716
O	6.1874458907	0.7948179047	-0.0067631021
H	4.728238619	-0.9965086788	1.4338950677
O	2.0824740925	-1.3762320189	1.3411078261
C	-0.612145577	1.0121750801	-1.656463313
C	-2.1229417571	1.1932176233	-1.5501851768
C	-0.1720425335	0.3484189923	-2.9755158529
C	2.7342794717	2.9927625173	-2.7184604513
H	1.9599975099	3.4746823181	-3.3169308347
H	3.2728492739	3.7501142033	-2.1374518393
H	3.4351042648	2.4696914493	-3.3785102934
C	2.7333664914	-2.2244357681	2.278116227
H	1.9416713279	-2.8313703995	2.7168703925
H	3.4636008724	-2.876074163	1.7838074689
H	3.230294189	-1.6431812063	3.0638814329
C	2.6075360961	-3.0672286379	-2.2986031601
H	3.5012123167	-2.4833093131	-2.5220715459
H	2.8823873729	-3.9346464879	-1.6866266889
H	2.1487738018	-3.4074675876	-3.2344246768
C	-3.7474902683	-1.4138904276	0.695046466
H	-4.1650804624	-0.4826142963	1.0771664114
H	-4.3010309978	-1.7194245355	-0.1987429854
H	-3.813647672	-2.197153393	1.4597569926
C	-1.1152194208	-2.2543266593	3.4660785688
H	-1.1333696936	-3.2647888749	3.054883236
H	-0.4721420409	-2.2335650723	4.3547302482
H	-2.1323532029	-1.9553823805	3.7457996067
C	0.661307628	4.1846039937	0.7324308884
H	1.039849165	4.5038532981	-0.2387420558
H	-0.2237971511	4.7758846226	0.9926807221
H	1.4350178462	4.32054035	1.4957933573
C	-3.020211686	0.3952287794	-2.2759555969
C	-4.3934237622	0.6422571461	-2.2391918392
C	-4.9012556992	1.6909120455	-1.4711248209
C	-4.0203040925	2.4944653455	-0.7456064927
C	-2.6477721768	2.2503012636	-0.7911909958
H	-2.654988289	-0.4228817606	-2.887152559
H	-5.0654587696	0.0187472918	-2.8229914848
H	-5.9693033254	1.8885473289	-1.4497255983
H	-4.3990736053	3.324927977	-0.1558903248
H	-1.972371753	2.9000310859	-0.2452993267
H	-0.553816989	-0.6697383903	-3.0787533639
H	-0.5622019098	0.9412055407	-3.8095543417

H	0.9141012301	0.3125765037	-3.0619156574
H	-0.1512705973	1.9945257119	-1.5971339684
C	-0.9407352561	-6.524972841	-1.8500470504
H	-1.5677360952	-7.4165566777	-1.8800449427
H	-0.6764280018	-6.2310093875	-2.8730462045
H	-0.0286518996	-6.7395502519	-1.2798888408
C	-1.4292333212	1.8263686796	6.2567191787
H	-1.580542121	2.5709541234	7.0387487486
H	-2.371371346	1.2973510802	6.0691272256
H	-0.6612945996	1.112169148	6.5780401517
C	6.9927418886	0.0185477986	0.8763114696
H	8.0145072905	0.3688755952	0.7274325534
H	6.7020106468	0.1756262003	1.9220393494
H	6.934707389	-1.0494116311	0.6330353436

Y30

P	0.0175695136	-0.2564390522	-1.3656691077
N	-0.1970946411	1.3600268862	-0.9296881085
N	-1.2967953307	-1.062522712	-0.6293298958
N	1.3496008016	-0.8897398346	-0.5363199548
C	-2.5662261005	-1.1806384563	-1.3436886256
C	1.8401159748	-2.238022285	-0.7975654879
C	0.5960949728	2.4129618795	-1.5674506002
H	1.1480348859	1.9868292133	-2.4064371156
H	-0.062717029	3.2006487999	-1.9534682458
H	1.3092104273	2.8707963135	-0.865702267
H	-2.4382076283	-0.8709361461	-2.3830067326
H	-2.9087607778	-2.2238496116	-1.3477173392
H	-3.3600372657	-0.5624090791	-0.8965020352
H	1.1624553337	-2.7598109643	-1.4775712239
H	2.838130998	-2.2195710173	-1.2589114851
H	1.9050934188	-2.8285134948	0.1282608937
H	-0.324718716	-2.3642735342	0.7093094778
H	-2.0761966671	-2.3649934256	0.7908024467
H	3.0395080069	-0.5092734856	0.6224106825
H	2.0992805173	0.9165420223	0.2248812133
H	-1.9895599557	1.2414031977	0.1463991889
H	-1.2838828326	2.84833572	0.0394713013
H	0.4974704163	2.1909134097	1.667524089
H	-1.1248058186	1.9933418971	2.3251850229
H	1.8878694417	0.3915275361	2.6085862
H	1.3310222266	-1.2427483209	2.2633094142
H	-2.0946943701	-0.2334372404	2.0066006303
H	-1.0553604014	-1.411512431	2.8002472296
N	-0.0823881993	0.2165058486	1.8952451567
C	-1.1433239891	-0.7696616704	1.9062809749
C	-0.4157839457	1.5902528254	1.5808258529
C	-1.0424597234	1.7876303606	0.1865876379
C	2.0119570013	-0.1335852213	0.5221294784
C	-1.2029302168	-1.7088839965	0.681681768
C	1.3012677428	-0.2103188522	1.8932357958
C	0.2468624175	-0.4251367276	-3.0299863077
C	-0.497500579	0.135997216	-4.1407619109
H	1.0392671786	-1.126138196	-3.279280626
C	-0.1508552093	-0.2449745046	-5.4632067373
C	-0.8045209352	0.2702292271	-6.5764564759

C	-1.8443112839	1.1947731105	-6.4348967803
C	-2.2059000476	1.5885914847	-5.1451608721
C	-1.5531380513	1.0773733361	-4.0252512059
H	0.6543755297	-0.9638145447	-5.6027024325
H	-0.4992485434	-0.056285824	-7.568790336
H	-2.3568748301	1.5956833701	-7.3048790483
H	-3.0100778597	2.3084386345	-5.0035298668
H	-1.8594088567	1.4194853393	-3.0401436299

Y30H+

P	0.2321812388	-0.0599002583	-1.2936644533
N	-0.0736316058	1.5252195164	-0.8762350426
N	-1.0965409004	-1.0319695603	-1.0336086819
N	1.5911727045	-0.6522921537	-0.5237245687
C	-2.3957469835	-0.7987940738	-1.6993278398
C	2.2048890722	-1.942819229	-0.8875817435
C	0.8603159333	2.6187189802	-1.196557766
H	1.6696930108	2.2715180226	-1.840723427
H	0.3302554969	3.4262212334	-1.7127744942
H	1.3137455256	3.0268103293	-0.2853329214
H	-2.3323839271	0.0240216014	-2.410062013
H	-2.7053458631	-1.6976179357	-2.2411908344
H	-3.1626557995	-0.5542573973	-0.9547998006
H	1.6525160722	-2.4336024883	-1.6911169116
H	3.2401705099	-1.7963614271	-1.2142794672
H	2.2024222634	-2.6235050775	-0.0284187377
H	-0.2583520226	-2.481680947	0.2466737018
H	-1.9987427818	-2.568265486	0.0482673093
H	3.0291686623	-0.4747746568	0.9764341543
H	2.1326149888	0.9996412605	0.6654360582
H	-2.034790158	1.3721960334	-0.1263853077
H	-1.25449334	2.9218593732	0.126695347
H	0.183857781	1.9631301546	1.9112329351
H	-1.4985118219	1.5814827976	2.2605458198
H	1.3962608768	0.0995563246	2.8303849503
H	1.0663110615	-1.4799697562	2.1259248265
H	-2.3054884109	-0.5479031287	1.4713003468
H	-1.3149320971	-1.7187007507	2.3349213653
N	-0.2987704978	-0.0238323644	1.555417946
C	-1.327441845	-1.0411815698	1.4674421509
C	-0.6726316505	1.3763248195	1.5621331231
C	-1.0866764199	1.8402784942	0.1518389404
C	2.0280289058	-0.0846642492	0.7667474223
C	-1.163896025	-1.8722084725	0.1794041129
C	1.0523182986	-0.4050905331	1.9145421751
C	0.678365953	-0.0919470468	-3.0890563212
C	-0.4064771338	0.1793569548	-4.1101583312
H	1.1128267473	-1.08071171	-3.2649185033
C	-0.948224121	-0.8782713209	-4.8531758935
C	-1.9384957937	-0.6433289823	-5.807754874
C	-2.3999881801	0.6546213599	-6.032614464
C	-1.8588243635	1.7168690964	-5.3053326403
C	-0.8666761537	1.4815588759	-4.3529241582
H	-0.587569407	-1.891649041	-4.6917489004
H	-2.3425378403	-1.4731432187	-6.3802383719
H	-3.1676516165	0.8396178377	-6.7780121197

H	-2.2012887298	2.7315607455	-5.4867361841
H	-0.4411670075	2.3194310328	-3.807255107
H	1.5039683941	0.6210619911	-3.1961778058

Y31

C	-1.2714317962	1.5974856785	-0.2594765281
C	-2.3274551567	2.3814192788	-0.7531469174
H	-3.2687794402	1.8960801506	-0.9930122369
C	-2.2554521626	3.7764889284	-0.8740624
H	-3.1166085254	4.3247757292	-1.2513933825
C	-1.1298490139	4.4434251469	-0.4567751277
H	-1.0786189365	5.5293788951	-0.470878694
C	0.0000618766	3.7146891445	0.0000545556
C	1.1299863483	4.4433956315	0.4568973885
H	1.0787728308	5.529349784	0.4710314817
C	2.2555881392	3.7764311061	0.8741414068
H	3.1167606057	4.3246943601	1.2514695279
C	2.327567936	2.3813631967	0.753183541
H	3.268900206	1.8960134637	0.9929973527
C	1.2715216295	1.59745407	0.2595214685
C	0.0000503503	2.2673633642	0.0000345961
C	-1.4995930165	0.1874894757	0.0508748897
H	-0.7565169686	-0.3325081817	0.6437124908
C	-2.1863590606	-3.2044468018	-0.7695612411
H	-1.092368026	-3.140693281	-0.8827810159
H	-2.6497952995	-2.9324791395	-1.719719572
H	-2.4460334058	-4.2482313074	-0.5504307238
C	-2.197420221	-2.725321829	1.6194223072
H	-1.0998652192	-2.6563403114	1.6893226623
H	-2.4876374156	-3.7608037667	1.8417860425
H	-2.6312751373	-2.0729042927	2.3798687328
C	-2.8713135448	-0.3886714605	-2.7627104822
H	-3.1685143227	0.5315331831	-3.2833996722
H	-3.0133903953	-1.2351494932	-3.4523778622
H	-1.815951071	-0.3098516495	-2.5007957493
C	-5.0976334085	-0.6648935903	-1.8014735832
H	-5.6627851274	-0.5775863306	-0.8749482903
H	-5.3614377312	-1.6176386783	-2.28649684
H	-5.4153713621	0.1495995784	-2.4670745853
C	-5.2345120654	-1.2720340606	1.4943206819
H	-5.2046575081	-1.4762432493	2.5756020878
H	-5.1332560241	-2.2203828409	0.9646852951
H	-6.2231637655	-0.841285355	1.2695345589
C	-4.1957766528	0.9219140123	1.8087357111
H	-4.2376384799	0.7656259321	2.8965977432
H	-5.0812059526	1.5066238966	1.5168630357
H	-3.3035238467	1.5037408768	1.5765004248
C	1.4996581278	0.1874648004	-0.0508812705
H	0.756592105	-0.332484068	-0.6437731176
C	2.8713254271	-0.3889755384	2.7627119457
H	3.0134613585	-1.2354622937	3.4523546644
H	1.8159566651	-0.3102058973	2.5008083768
H	3.1684744451	0.5312350074	3.2834204769
C	5.0976518859	-0.6650939136	1.801460829
H	5.4154017551	0.1494684708	2.4669743928
H	5.6627855421	-0.5778837511	0.8749119903

H	5.3614648306	-1.617790148	2.2865729561
C	4.1959952387	0.9220363405	-1.8085066721
H	3.3037614794	1.5038792575	-1.5762397379
H	4.2378922019	0.7658741958	-2.8963856431
H	5.0814421992	1.5066683527	-1.5165314637
C	5.2346731635	-1.2719594169	-1.4942332541
H	6.2233034189	-0.8412304567	-1.269314291
H	5.204916808	-1.476065225	-2.5755371524
H	5.133367313	-2.2203573939	-0.9646956038
C	2.1863163929	-3.2045472795	0.7692420841
H	2.6497402548	-2.9326915364	1.7194384106
H	2.4459500692	-4.2483211985	0.5500135618
H	1.0923247194	-3.1407565005	0.8824372707
C	2.1974502227	-2.7251706471	-1.6196906185
H	1.0999003982	-2.6561198287	-1.6896021516
H	2.4876146998	-3.7606450618	-1.842157053
H	2.6313529693	-2.0726966221	-2.3800607188
N	-2.6970770149	-2.3512776042	0.2976210222
N	-3.6668177427	-0.5598758908	-1.5492920296
N	-4.1547343834	-0.3657664193	1.1223812961
N	3.6668326321	-0.5601238494	1.5492866778
N	4.1548658262	-0.3657227856	-1.1223053032
N	2.6971040721	-2.3512923763	-0.2978398694
P	-2.9406611609	-0.6906658651	-0.0268193703
P	2.9407152176	-0.6907171213	0.0267802872

Y31H^{+}

C	0.9347675853	3.2205862248	-0.0359492169
C	0.5474977292	1.8728888171	0.3069966062
C	-0.7306654921	1.3704878105	-0.1943220147
C	-1.2898647137	2.0730976361	-1.274939443
H	-2.2055653385	1.7029319711	-1.7252349515
C	-0.8043432957	3.3193246105	-1.7053216915
H	-1.3117254987	3.8296805195	-2.5201611047
C	0.24417021	3.9282701282	-1.052045254
H	0.563881296	4.933754778	-1.3096343023
C	2.0321260718	3.8312589794	0.6326975666
H	2.2668909013	4.8655941045	0.3942307157
C	2.7782469904	3.1428572986	1.5583701276
H	3.5934332578	3.6275169964	2.0877520553
C	2.521716713	1.7706131403	1.7588815761
H	3.1890903811	1.196663827	2.3968730854
C	1.4696750397	1.1277788759	1.1228621957
C	-1.4225425909	0.2701495744	0.4564346374
C	1.4698105378	-0.3901224018	1.150224525
N	-3.5629501612	-1.1899165297	1.5457015734
N	-4.112141281	1.0385303138	0.0349799825
N	4.2101639338	-1.0479396855	0.5450350116
N	2.1920184801	-2.7238433622	-0.2230626305
N	2.7243201257	-0.3769656682	-1.5285727184
P	-3.0442641217	-0.2473200233	0.2357784484
P	2.6614899715	-1.1310989444	-0.0456507561
N	-3.2690236837	-1.3156283247	-1.0506587032
H	0.4957764594	-0.7840245764	0.8585370458
H	1.6962572847	-0.7909202513	2.1439871157
H	-1.1149400366	0.0303616145	1.4702369934

C	-2.4237413309	-1.2337413255	-2.2370024462
H	-1.5137119903	-0.676827625	-2.0069589825
H	-2.1420801172	-2.2462003779	-2.5569889271
H	-2.9366107027	-0.7450387309	-3.0781207998
C	-4.4064969915	-2.2257871523	-1.1854710004
H	-5.0460619275	-1.9540628104	-2.0372990731
H	-4.0446063947	-3.2494639499	-1.35446261
H	-5.0071047747	-2.2189397518	-0.2752641349
C	-5.4105414208	0.9312702894	-0.6304922938
H	-6.2336357666	0.7991575388	0.0866678581
H	-5.5989338533	1.8504481393	-1.1980666349
H	-5.4198028335	0.0980060331	-1.3317940622
C	-3.9290927018	2.3023316984	0.7551348855
H	-3.9684137245	3.1400859233	0.0490117094
H	-4.7166270634	2.4486629098	1.5079323247
H	-2.9583495364	2.3155178076	1.2514105562
C	-4.1189845715	-0.5371081925	2.7329069439
H	-4.770257354	-1.246962068	3.2552910955
H	-3.3408967399	-0.2039885756	3.4368759812
H	-4.7226303516	0.3240236417	2.4446450829
C	-2.8553779645	-2.434055496	1.8516881803
H	-2.5172860563	-2.908516796	0.9282855126
H	-1.9850257077	-2.2698688372	2.5068876599
H	-3.538183814	-3.1227926398	2.3637451466
C	0.7997756963	-3.1923335002	-0.3034498529
H	0.6164614039	-3.6591315782	-1.2800691695
H	0.6156550391	-3.9424519379	0.4747724836
H	0.08390265	-2.3777068355	-0.1848599431
C	3.1633591702	-3.7452558475	-0.6395878133
H	4.1827296291	-3.4110546543	-0.4452123494
H	2.987610942	-4.6601163339	-0.0630032859
H	3.0659856872	-3.9829412179	-1.7072839977
C	5.4094351753	-0.9561672534	-0.3017655563
H	5.9997304315	-0.0779944873	-0.0156257355
H	6.0329329183	-1.8495801452	-0.1698903933
H	5.1361333023	-0.8735662771	-1.3523351507
C	3.2384609628	0.9953539681	-1.704063744
H	3.9293609317	1.0090349988	-2.5556903584
H	2.4172586355	1.6916604094	-1.8978991566
H	3.7661197859	1.3319758877	-0.8123458096
C	1.8803793127	-0.816012516	-2.647824963
H	0.9647579469	-0.2142736186	-2.7113487749
H	2.443643973	-0.6945866755	-3.5796009785
H	1.6158838977	-1.8689922586	-2.547772475
C	4.5257360677	-1.3552300382	1.9463730418
H	5.0732949596	-2.3036094047	2.0160818458
H	5.1516357289	-0.5602807546	2.3671899282
H	3.6251786871	-1.439936035	2.5539367614

References

[1] Disscussion on PA and GB of phosphine **8** is based on the measured PA(8) = 249 kcal mol^{-1}. Liu, M.; Yang, I.; Buckley, B.; Lee, J.K. Proton Affinities of Phosphines versus N-Heterocyclic Carbenes. *Org. Lett*. **2010**, *12*, 4764–4767.

[2] Klamt, A.; Eckert, F.; Diedenhofen, M.; Beck, M.E. First Principles Calculations of Aqueous pK_a Values for Organic and Inorganic Acids Using COSMO-RS Reveal an Inconsistency in the Slope of the pK_a Scale. *J. Phys. Chem. A* **2003**, *107*, 9380–9386.

[3] Eckert, F.; Leito, I.; Kaljurand, I.; Kütt, A.; Klamt, A.; Diedenhofen, M. Prediction of acidity in acetonitrile solution with COSMO-RS. *J. Comput. Chem.* **2009**, *30*, 799–810.

[4] Marenich, A.V.; Cramer, C.J.; Truhlar, D.G. Universal Solvation Model Based on Solute Electron Density and on a Continuum Model of the Solvent Defined by the Bulk Dielectric Constant and Atomic Surface Tensions. *J. Phys. Chem. B* **2009**, *113*, 6378–6396.

[5] Peräkyllä, M. Ab Initio Quantum Mechanical Study on the Origin of the pK(a) Differences of the Proton Sponges 1,8-Bis(dimethylamino)naphthalene, 1,8-Bis(dimethylamino)-2,7-dimethoxynaphthalene, 1,6-Dimethyl-1,6-diazacyclodecane, and 1,6-Diazabicyclo[4.4.4]tetradecane. *J. Org. Chem*. **1996**, *61*, 7420–7425.

[6] Kovačević, B.; Maksić, Z.B. Basicity of Some Organic Superbases in Acetonitrile. *Org. Lett.* **2001**, *3*, 1523–1526.

[7] Glasovac, Z.; Eckert-Maksić, M.; Maksić, Z.B. Basicity of Organic Bases and Superbases in Acetonitrile by the Polarized Continuum Model and DFT Calculations. *New J. Chem*. **2009**, *33*, 588–597.

[8] Liptak, M.D.; Shields, G.C. Accurate pK_a Calculations for Carboxylic Acids Using Complete Basis Set and Gaussian-n Models Combined with CPCM Continuum Solvation Methods. *J. Am. Chem. Soc*. **2001**, *123*, 7314–7319.
